# Bis(diiminate)-Supported Bimetallic Complexes: Tri-Coordinated Zinc for Nitrile and Carbodiimide Hydroboration

**DOI:** 10.1021/acsomega.4c08068

**Published:** 2025-01-10

**Authors:** Darakshan Parveen, Rahul Kumar Yadav, Felipe Fantuzzi, Dipak Kumar Roy

**Affiliations:** † Department of Chemistry, Indian Institute of Technology Indore, Khandwa Road, Simrol, Indore 453552, India; ‡ School of Chemistry and Forensic Science, University of Kent, Park Wood Rd, Canterbury CT2 7NH, U.K.

## Abstract

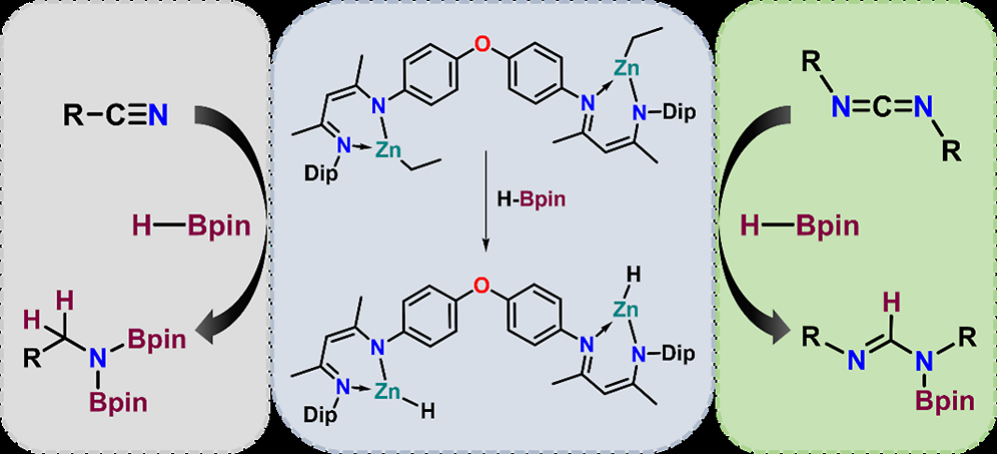

We report the synthesis and characterization of bis(diiminate)-supported tricoordinated zinc complexes (**1–4**) and demonstrate the catalytic activity of one representative compound in the hydroboration of nitriles and carbodiimides using pinacolborane (HBpin). Experimental and theoretical studies were performed to elucidate the reaction mechanism. Our findings indicate that the hydroboration reaction initiates with the formation of a tricoordinated zinc hydride intermediate, followed by the subsequent attack of nitriles and carbodiimides. This leads to the formation of a four-membered metallacycle before the release of the diborylated amine. This work provides access to new types of zinc complexes and highlights its effectiveness in the hydroboration of nitriles and carbodiimides, offering a milder alternative to existing reduction methods.

## Introduction

One of the environmentally favorable methods for reducing nitriles or nitro compounds is direct hydrogenation. However, this process usually requires harsh conditions, including elevated temperatures and pressures, Lewis acid additives, and precious metal catalysts, which frequently lead to poor product selectivity.^1^ In contrast, the hydroboration of nitriles, due to its milder reaction conditions, has recently gained significant attention from many research groups, as it allows for better selectivity of the products. Functionalizing the C≡N bond in nitriles can produce a range of value-added nitrogen-containing molecules, making nitriles an attractive substrate for hydroboration studies. Additionally, 1,1′-diborylamines, produced through the hydroboration of nitriles, are finding utility in the synthesis of imines and amides, and can also act as reagents in Pd-catalyzed C–N cross-coupling reactions.^2^ Since the first report of molybdenum-catalyzed nitrile hydroboration in 2012,^3^ several other catalytic systems have been developed.^4,5^ Newly discovered transition metal catalysts for nitrile hydroboration primarily utilize Ru^6^ and Co,^7^ with a limited number of examples involving Ti,^8^ Mn,^9^ Fe,^10^ and Ni.^11^ Furthermore, a few rare earth metal-based catalysts have been reported in the dihydroboration of nitriles.^12,13^ Notably, main group elements are emerging as promising alternatives to transition metals in catalysis, with Li, Mg, B, and Al compounds showing potential for the hydroboration of nitriles.^14,15^

Organometallic zinc compounds, which have found various applications in organic synthesis,^16,17^ including cross-coupling reactions^17b^ and asymmetric synthesis,^17c^ are valued for their versatile ability to participate in a wide range of reactions. Due to their high abundance, low toxicity, and biocompatibility, zinc catalysts are becoming suitable alternatives to precious and toxic metal complexes. β-diketiminato ligands have been instrumental in many areas of bioinorganic, main-group, and transition-metal chemistry due to their ability to fine-tune electronic and steric properties, through the introduction of different substituents on the nitrogen atoms or ligand backbone.^18^ By bridging two diketiminato centers with a spacer, it is possible to facilitate the cooperative action of two metal centers, similar to the spatial arrangement observed in natural metalloenzymes, where metals are positioned by a carefully designed ligand system. For example, in 2005, Ding and colleagues reported the copolymerization of cyclohexene oxide with CO_2_ using a dinuclear zinc complex.^19^ Later that year, Park introduced bimetallic anilido-aldimine zinc complexes for epoxide/CO_2_ copolymerization.^20^ In 2008, Harder and co-workers demonstrated the superiority of bimetallic zinc complexes over the monomeric zinc systems for epoxide/CO_2_ copolymerization.^21^

**Scheme 1:**
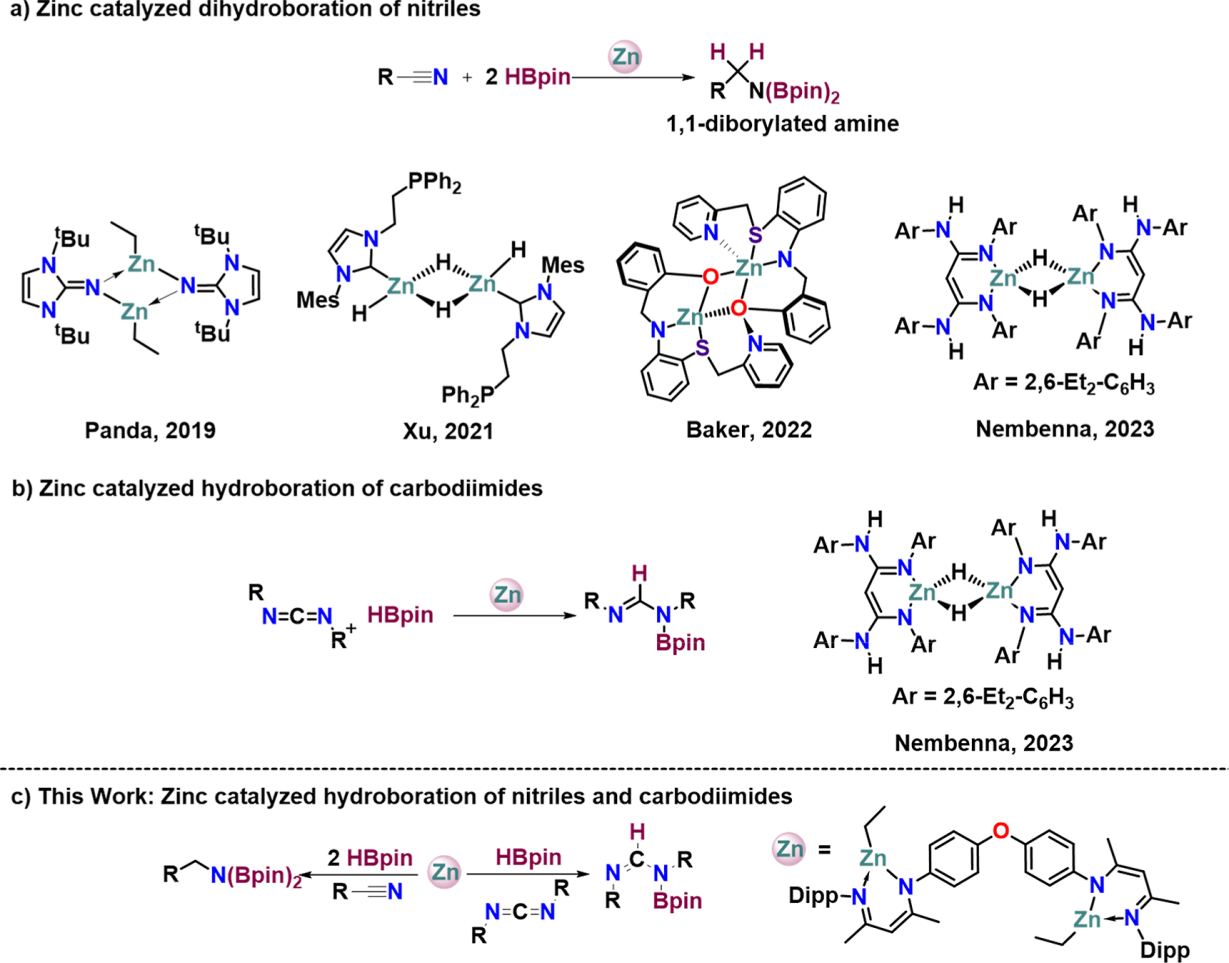
(a,b) Previously Reported Zinc Catalysts Employed for Nitrile and Carbodiimide Hydroboration; (c) This Work

Although numerous zinc catalysts have been reported, there are only a few instances in the literature specifically detailing the use of zinc catalysts for nitrile hydroboration (Scheme 1). The first report came from the Panda group,^22^ who used a zinc(II) imidazolin-2-iminato complex as a catalyst. Subsequently, Xu^23^ and Tom Baker^24^ reported the dihydroboration of nitriles using either N-heterocyclic carbene (NHC)–zinc dihydride or a pyridine-thioether-anilido-aryloxide-stabilized zinc complex, Zn(NSNO), respectively. Very recently, Nembenna and co-workers reported that a conjugated bis-guanidinate (CBG)-stabilized zinc hydride acted as a catalyst for the hydroboration of nitriles, resulting in the formation of 1,1′-diborylamines.^25^ In two of the reported procedures,^23,25^ zinc hydrides were prepared and used as catalysts. In the other two,^22,24^ in situ zinc hydride was generated; however, only a very limited scope was attained. In addition, while catalytic hydroboration of carbodiimides has been periodically reported, only one zinc-catalyzed example has been described.^26^ Therefore, there is a need to develop zinc catalysts for the hydroboration of nitriles and carbodiimides at lower temperatures with a wide substrate scope and broad functional group tolerance. In this study, we report a series of bis(diiminate) ligands and their bimetallic zinc complexes. In all these complexes, zinc exhibits a coordination number of three. Unlike the previously reported zinc catalysts for nitrile hydroboration, the zinc centers in our present study are separated by a spacer, keeping them far from each other. Among these zinc complexes, we have tested complex **1** as an effective catalyst for the hydroboration of nitriles and carbodiimides.

## Results and Discussion

### Synthesis and Characterization of **1**

**Scheme 2:**
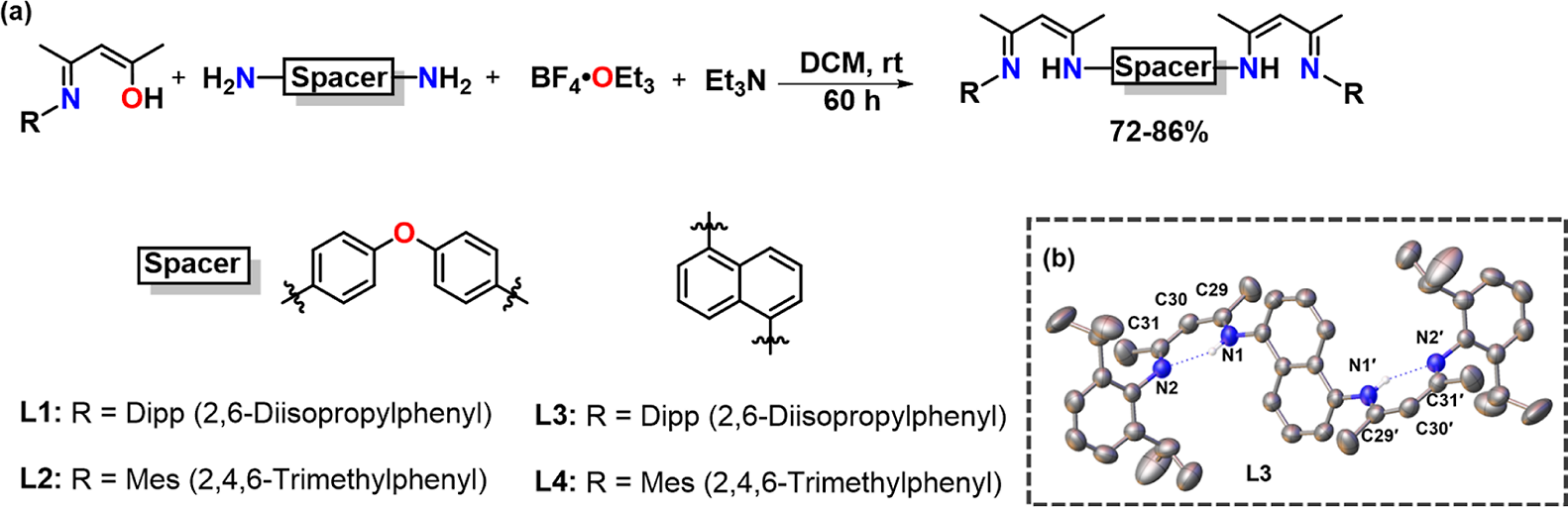
(a) Synthesis of *para*-Oxydiphenyl and 1,5-Naphthalene Bridged Ligands (**L1–L4**); (b) Molecular Structure of **L3** Thermal ellipsoids are shown at 50% probability level. Hydrogen atoms are omitted (except at N1) for clarity. Selected bond lengths (Å) and bond angles (deg): N2–C31 1.322(3), C31–C30 1.391(3), C30–C29 1.401(3), C29–N1 1.313(3); N2–C31–C30 121.3(2), C31–C30–C29 126.24(19), C30–C29–N1 120.24(19). Symmetry operations used to generate equivalent atoms: 1 – *X*, 2 – *Y*, 1 – *Z*.

Our investigations initiated with the synthesis of *para*-oxidiphenyl and 1,5-naphthalene spacer ligands (**L1–L4**) by modification of a previously reported procedure (Scheme 2a).^27^ The ligands were characterized by ^1^H and ^13^C NMR spectroscopy, high-resolution mass spectrometry (HR-MS), and single-crystal X-ray diffraction analysis. As depicted in Scheme 2b, hydrogen bonding is present between N–H···N, which is supported by the downfield shift of the N–H proton in ^1^H NMR (**L1**: 12.62 ppm; **L2**: 12.45 ppm; **L3**: 12.62 ppm; **L4**: 12.41 ppm). The N2–C31 bond length is 1.322(3) Å, which falls in between the lengths of a C–N single and double bond.

**Scheme 3:**
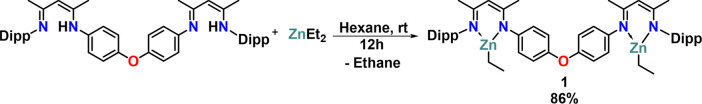
Synthesis of *para*-Oxydiphenyl-Bridged Zinc Complex **1**

Complex **1** was synthesized by treating ligand **L1** with 3 equiv of diethyl zinc in hexane, resulting in 86% yield of the corresponding complex as a yellow solid (Scheme 3). The ^1^H NMR spectrum exhibits a resonance at ∼5 ppm as a singlet for the γ-methine proton, similar to that observed in the ethylene-bridged bis(β-diketiminate)-supported zinc complex^28^ and zinc complexes with bridged β-diketiminate ligands.^21^ Furthermore, a triplet is observed at 1.08 ppm corresponding to the methyl (CH_3_) group and a quartet at 0.41 ppm for the methylene (CH_2_) protons of the ethyl groups attached to the zinc centers. This suggests that the CH_2_ protons are more shielded in comparison to the CH_3_ protons. The ^1^H NMR pattern for the ethyl group attached to Zn is similar to that of the earlier reported imidazolin-2-iminato zinc(II) complexes^22^ and the ethylene-bridged bis(β-diketiminate)-supported zinc complex.^28^

### Catalytic Studies: Nitrile Hydroboration

Despite numerous studies on the zinc-catalyzed hydroboration of unsaturated organic substrates, there have been only four reports on the hydroboration of nitriles catalyzed by zinc complexes to yield 1,1′-diborylated amines.^22−25^ This motivated us to investigate the utility of zinc complex as a catalyst in nitrile and carbodiimide hydroboration. Initial data showed that when 0.4 mmol of benzonitrile and 0.8 mmol of HBpin was heated at 60 °C for 12 h in C_6_D_6_ in the presence of 5 mol % loading of **1**, the corresponding 1,1′-diborylated amine, **1d** was obtained in 95% yield. The ^1^H NMR spectrum of **1d** displayed a new singlet at 4.28 ppm, corresponding to the methylene (2H) group of the product. We then tested the reaction by keeping the loading of **1** at the same 5 mol % and increased the temperature to 80 °C. At 6 and 12 h, the reaction resulted in 65% and 92% product formation, respectively, indicating that higher temperatures do not significantly improve the yield of the product. When we reduced the mol % of **1** to 3 mol % at 60 °C for 12 h in C_6_D_6_, the product yield was cut down to 79%. Under solvent-free conditions with 3 mol % of **1** at 60 °C for 12 h, the yield increased to 88%. However, at room temperature, the yield dropped to 46% even after 24 h. Reducing the catalyst loading to 1 mol % under solvent-free conditions at room temperature for 24 h yielded only 11% of the product, whereas heating at 60 °C for 12 h improved the yield to 69%. Control experiments without a catalyst yielded no conversion, confirming that the Zn complex **1** is essential for the transformation. Under standard conditions, using ZnEt_2_ as a catalyst, only 22% of the product formation was achieved. These results underscore the crucial role of zinc and the bis(diiminate) ligand in these reactions (Table 1). Additionally, the corresponding monomeric zinc complex was synthesized following the reported procedure^29^ to compare and validate the necessity of preparing the dimeric zinc catalyst (see the Supporting Information, Scheme S3 and Figures S25 and S26). Subsequently, 5 and 10 mol % of the monomeric zinc complex (Table 1, entries 11 and 12) were employed as catalysts for the hydroboration of benzonitrile under standard reaction conditions, resulting in 44% and 56% of product formation, respectively.

**Table 1:** Optimization Table for the Hydroboration of Nitriles Catalyzed by Complex **1**^a^

entry	Cat. (mol %)	solvent	*T* (°C)	*t* (h)	NMR yield (%)	^1^H NMR figure
1	0	C_6_D_6_	60	12	0	S27
2	1	neat	rt	24	11	S28
3	1	neat	60	12	69	S29
4	3	neat	rt	24	46	S30
5	3	neat	60	12	89	S31
6	3	C_6_D_6_	60	12	79	S32
**7**	**5**	**C**_**6**_**D**_**6**_	**60**	**12**	**95**	S33
8	5	C_6_D_6_	80	6	65	S34
9	5	C_6_D_6_	80	12	92	S35
10	Et_2_Zn	C_6_D_6_	60	12	22	S36
11	5^b^	C_6_D_6_	60	12	44	S37
12	10^b^	C_6_D_6_	60	12	56	S38

a Reactions were conducted with benzonitrile (2 equiv), HBpin (4 equiv), and complex **1** in a J Young NMR tube. Yields were determined by ^1^H NMR spectroscopy using 1,3,5-trimethoxybenzene as an internal standard.

b Monomeric Zn complex.

**Scheme 4:**
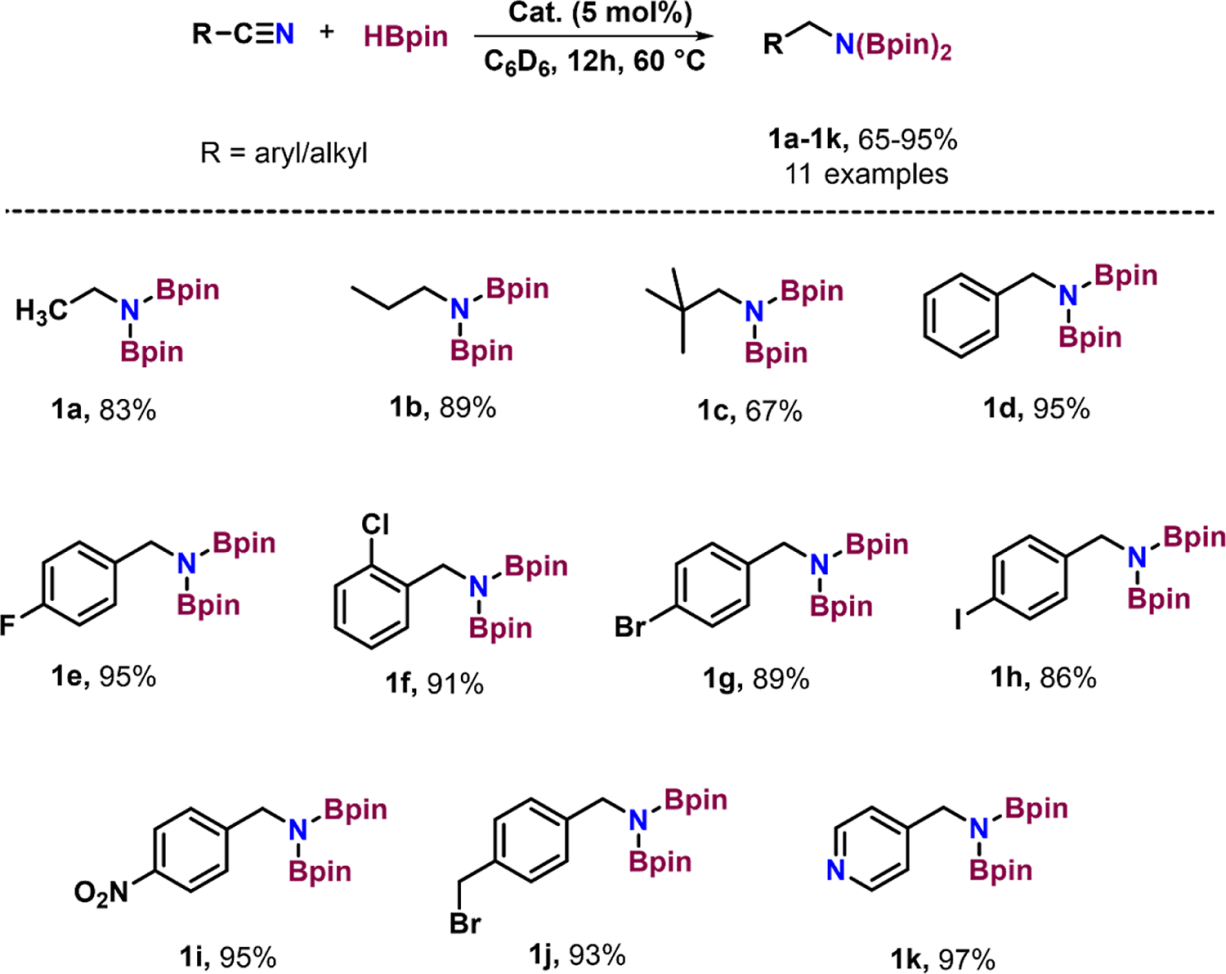
Hydroboration of Nitriles Catalyzed by Complex **1** Reactions were conducted with nitrile (0.4 mmol, 2.0 equiv), HBpin (0.8 mmol, 4.0 equiv), and complex **1** (5 mol %) in C_6_D_6_, in a J Young NMR tube at 60 °C for 12 h. Product formation was examined by ^1^H ^13^C{^1^H} and ^11^B{^1^H} NMR spectroscopy based on the appearance of a characteristic new proton resonance for the (C*H*_2_N(Bpin)_2_) moiety of products **1a–1k**.

**Scheme 5:**
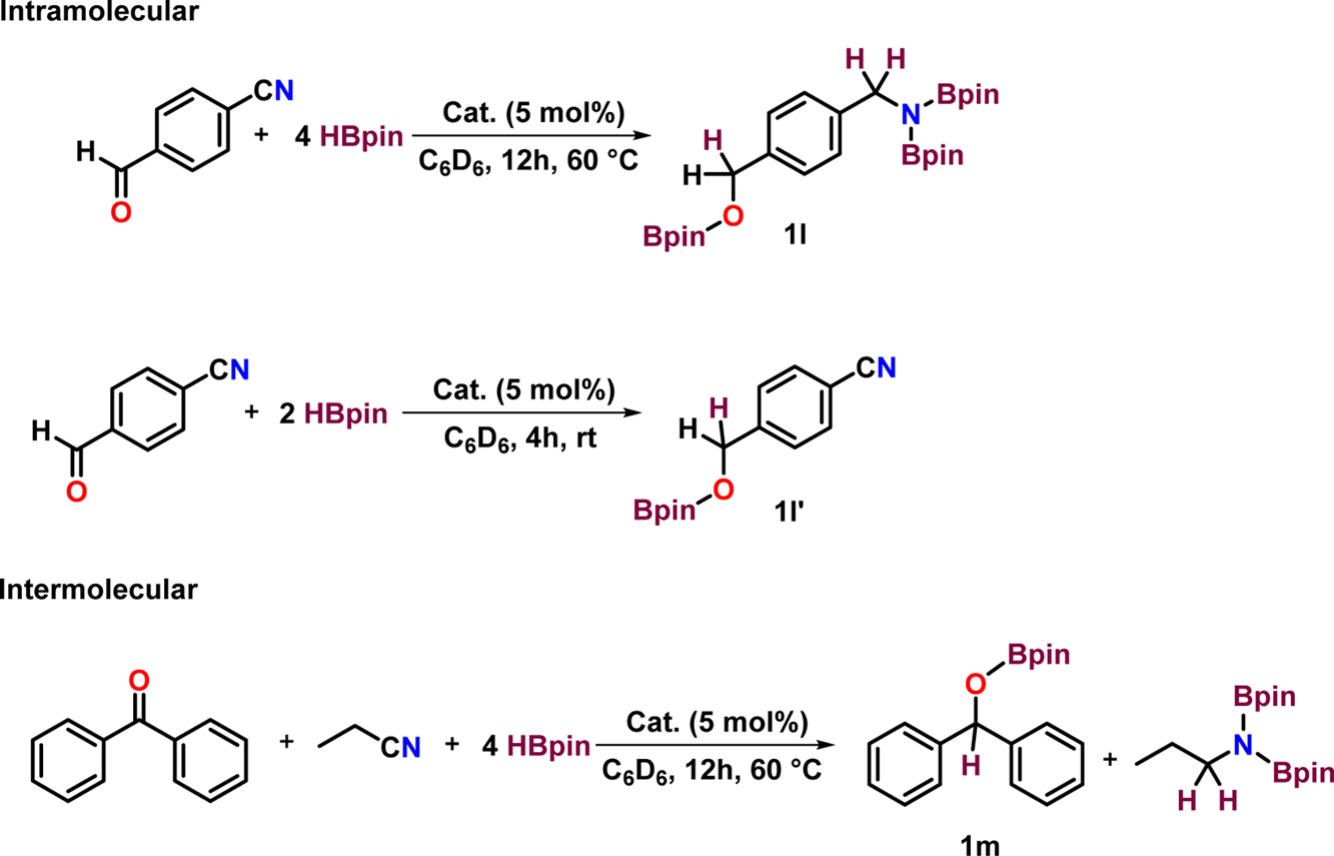
Competition Reaction Catalyzed by Complex **1**

Encouraged by the outcome of zinc-catalyzed hydroboration of benzonitrile, we were prompted to further investigate the potential of nitriles as substrates. Using the standard reaction conditions, we subjected various nitriles to 5 mol % of complex **1** and 4 equiv of HBpin. This resulted in the formation of the corresponding 1,1′-diborylamines (**1a–1k**) with 65–97% yields within 12 h at 60 °C (Scheme 4). During our substrate scope investigation, we attempted the hydroboration of 4-cyanobenzaldehyde using a 5 mol % catalyst loading in C_6_D_6_ at 60 °C for 12 h. Alongside the hydroboration of nitrile group to 1,1′-diborylated amine product, the −CHO functional group is also hydroborated obtaining **1l** (Scheme 5). To further comprehend the chemo selectivity between the nitrile and formyl group, we carried out a controlled hydroboration of 4-cyanobenzaldehyde in C_6_D_6_ at room temperature for 4 h with 2 equiv of HBpin and 5 mol % of **1**. To our delight, instead of forming the 1,1′-diborylated amine, the ^1^H NMR spectrum revealed a new singlet peak at 4.42 ppm, corresponding to the C*H*_2_ group of the hydroborated product of the carbonyl group (**1l′**). This suggests that hydroboration occurred at the carbonyl moiety rather than the nitrile functional group at room temperature. This led us to perform a competition reaction between benzophenone and propionitrile, using 0.4 mmol of each. The result showed hydroboration of both the benzophenone and propionitrile after 12 h of heating at 60 °C (Scheme 5). To investigate the reaction’s selectivity, we conducted a controlled experiment under standard reaction conditions. A reaction between benzophenone and propionitrile was carried out, and the ^1^H and ^11^B{^1^H} NMR spectra were recorded at different time intervals after heating at 60 °C. After 1 h, the ^1^H NMR revealed a new peak at 6.36 ppm, while the ^11^B{^1^H} NMR showed a peak at 22.7 ppm, indicating that hydroboration occurred at the benzophenone before the propionitrile (see the Supporting Information, Figures S82 and S83). After 3 h, ^1^H and ^11^B{^1^H} NMR spectra were recorded again, showing the peak at 6.36 ppm, along with a new triplet at 3.33 ppm in the ^1^H NMR. Additionally, the ^11^B{^1^H} NMR displayed a new peak at 26 ppm alongside the existing 22.7 ppm peak (see the Supporting Information, Figures S84 and S85). This indicates that the hydroboration of propionitrile had begun. After 5 h, ^1^H and ^11^B{^1^H} NMR spectra were recorded once more, showing an increase in the intensity of the peak at 3.3 ppm (see the Supporting Information, Figures S86 and S87). Using the hydroborated product of benzophenone as a reference for 100% yield, the hydroboration of propionitrile reached a yield of 18% and 30% after 3 h and after 5 h of heating at 60 °C, respectively. This indicates that the hydroboration selectively occurred at the benzophenone before proceeding to the propionitrile. The outcome of the reaction resembled that of intermolecular nitriles and ketones hydroboration using HBpin with aluminum-^30^ and zinc-based^31^ catalysts. Notably, this protocol is also effective for gram-scale synthesis of 1,1′-diborylated amine under optimized reaction conditions (Scheme S6).

### Carbodiimide Hydroboration

Building on the successful hydroboration of nitriles using the zinc complex, we aimed to explore the catalytic activity of complex **1** for the hydroboration of carbodiimides. To our knowledge, there has been only one report on the zinc-catalyzed version of this reaction.^26^ Therefore, it is essential to investigate a broader range of catalytic processes to expand the scope of carbodiimide hydroboration.

**Scheme 6:**
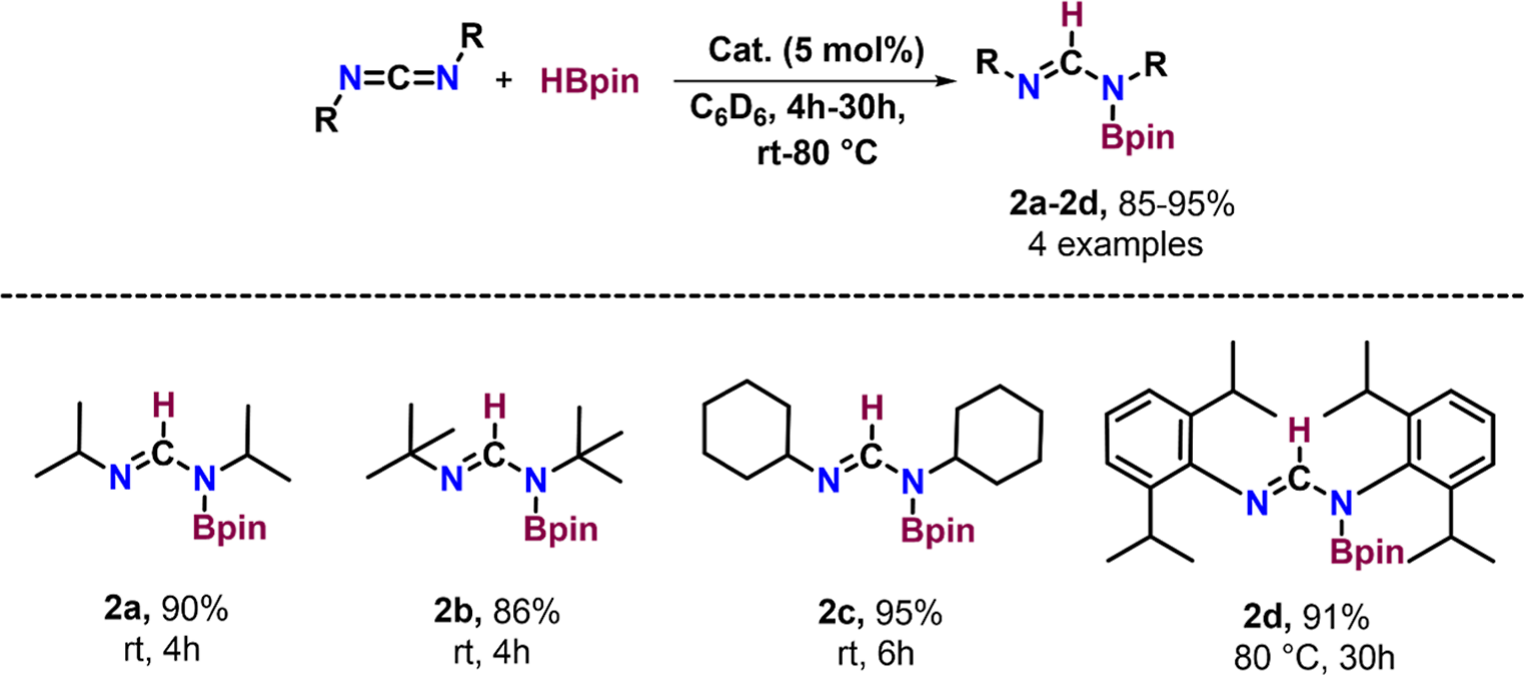
Hydroboration of Carbodiimides Catalyzed by Complex **1** Reactions were conducted with carbodiimides (0.14 mmol, 2.0 equiv), HBpin (0.28 mmol, 4.0 equiv), and complex **1** (5 mol %) in a J Young NMR tube.

The addition of 5 mol % of complex **1**, 4 equiv of HBpin, and 2 equiv of carbodiimides in C_6_D_6_ afforded the corresponding *N*-boryl formamidine derivatives **2a–2d** in 85–95% isolated yield (Scheme 6). Compounds **2a–2d** were well characterized using ^1^H, ^11^B{^1^H}, and ^13^C{^1^H} NMR spectroscopy, as well as LC–MS spectrometry. In the ^1^H NMR spectra, we observed a new peak at ∼8 ppm, which corresponds to the NC*H* protons of the *N*-boryl formamidine derivatives **2a–2d**. Furthermore, a resonance at ∼150 ppm in the ^13^C{^1^H} NMR spectrum indicates the presence of the N*C*N carbon atom in these derivatives.

### Reaction Mechanism

**Scheme 7:**
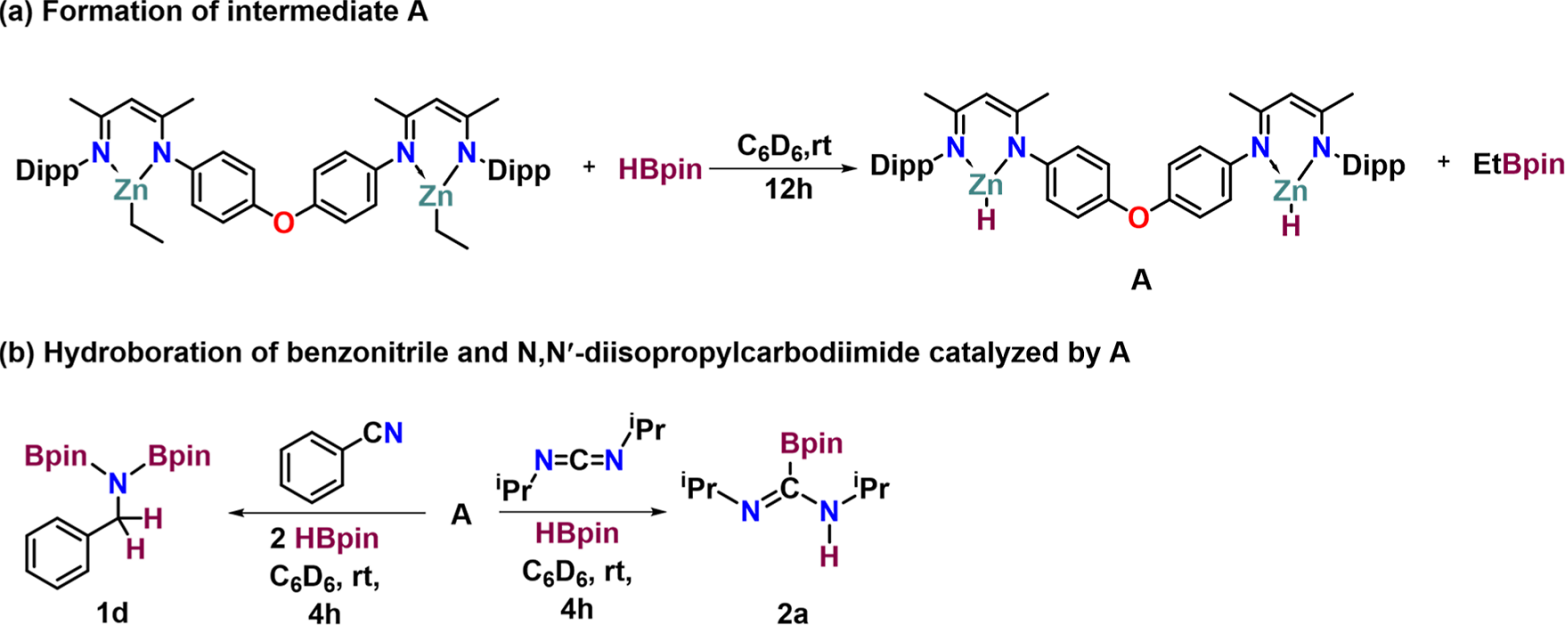
Stepwise Addition of HBPin and Substrate for the Mechanistic Study of Hydroboration

To shed more light into the mechanism of the hydroboration of nitriles and carbodiimides catalyzed by complex **1**, a stoichiometric reaction was performed to explore the potential catalytic pathway. Initially, complex **1** was treated with HBpin, resulting in the formation of a zinc hydride intermediate (**A**) and EtBpin in situ (Scheme 7a). **A** was characterized by ^1^H NMR spectroscopy, exhibiting a chemical shift at δ = 3.5 ppm corresponding to Zn–H, while EtBpin was identified by ^11^B{^1^H} NMR spectroscopy with a resonance at 34.6 ppm (see the Supporting Information, Figures S39 and S40). Next, 1 equiv of benzonitrile was added to the reaction mixture containing Zn–H, followed by heating for 12 h at 60 °C. Upon recording the ^1^H NMR spectrum, the Zn–H peak at 3.5 ppm had disappeared, and a new peak at 4.3 ppm corresponding to PC*H*_2_N(Bpin)_2_ was observed, along with unreacted benzonitrile. Subsequently, an additional equiv of HBpin was added to the same NMR tube, and the mixture was heated again for 12 h at 60 °C. The ^1^H NMR spectrum revealed complete consumption of the remaining benzonitrile, with only product (**1d**) peaks detected (Scheme 7b). Similarly, *N*,*N*′-diisopropylcarbodiimides (1 equiv) and HBpin (1 equiv) were added to **A**, leading to the formation of product **2a** (Scheme 7b). The formation of **2a** was confirmed by ^1^H NMR and ^11^B{^1^H} NMR spectroscopy. The ^1^H NMR spectrum displayed a new resonance at 8 ppm corresponding to the NC*H*N proton while a broad singlet appeared at 25.3 ppm in the ^11^B{^1^H} NMR spectrum.

### Theoretical Study

Inspired by the mechanistic proposal suggested by Panda and co-workers,^22^ the reaction mechanism of the hydroboration of nitriles and carbodiimides mediated by complex **1** was further investigated through density functional theory (DFT) calculations. Energies were computed at the PBE0-D3(BJ)/def2-TZVPP + SMD(benzene) level of theory using geometries optimized at the PBE0-D3(BJ)/def2-SVP level.^32^ The choice of the PBE0 functional was based on its reliable accuracy and effectiveness, as demonstrated in previous studies on metal-catalyzed hydroboration reactions.^33^ Additionally, we applied a concentration correction of Δ*G*^0→*^ = *RT* ln(24.46) = 1.89 kcal mol^–1^ to the free energies of all calculated species. This adjustment converts the 1 atm gas-phase values (Δ*G*^0^) to a condensed phase standard state concentration of 1 M (ΔG*), providing a more accurate representation of associative and dissociative steps.^34^ For more details, see the Supporting Information.

**Figure 1:**
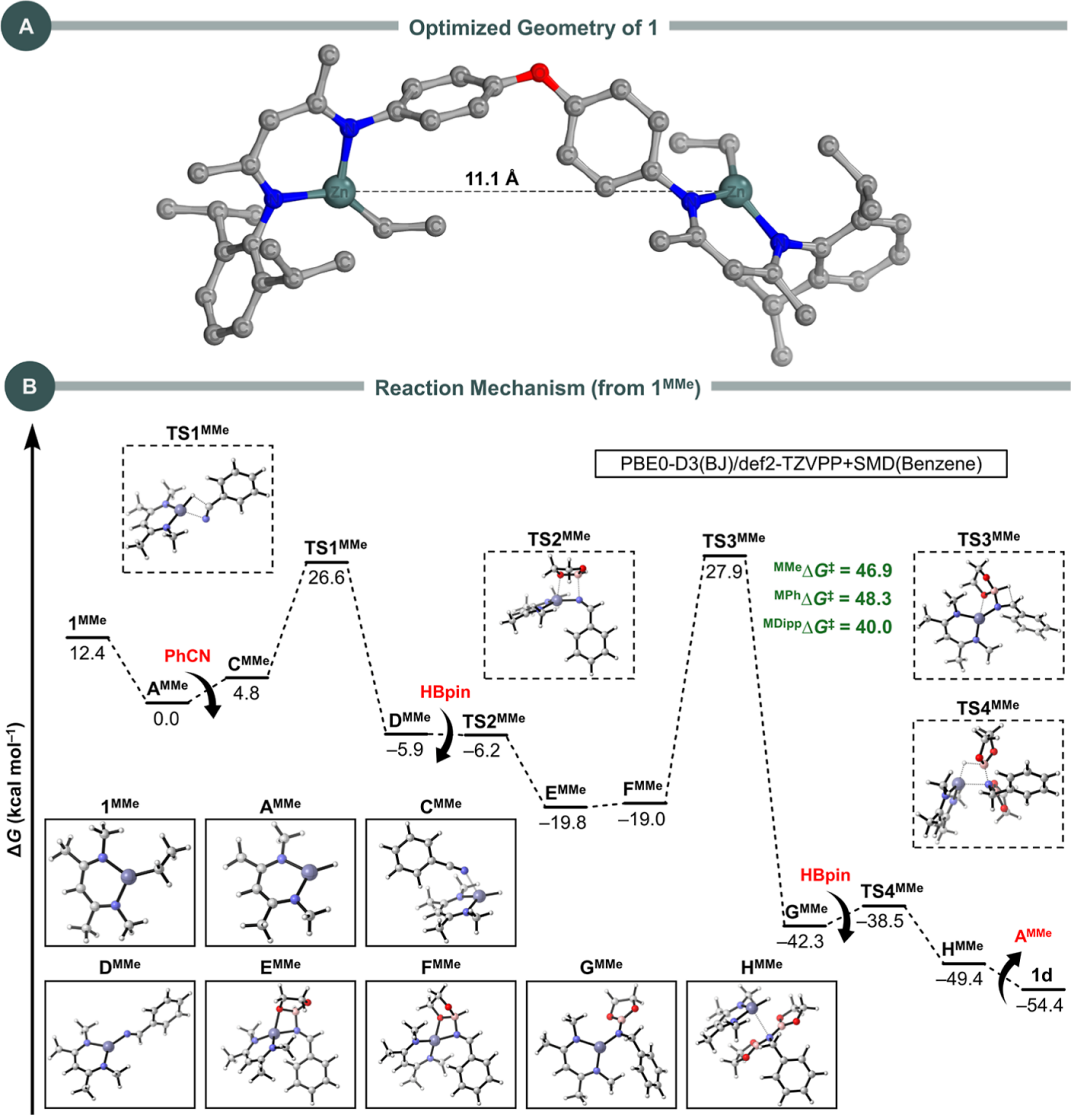
(A) 3D representation of the optimized structure of **1** at the PBE0-D3(BJ)/def2-SVP level of theory. (B) Computed Gibbs free energies in kcal mol^–1^ for the hydroboration of PhCN to **1d** using **1**^**MMe**^ as the precatalyst. Energies are at the PBE0-D3(BJ)/def2-TZVPP + SMD(benzene) level of theory, from optimized structures at PBE0-D3(BJ)/def2-SVP. The 3D representation of all optimized structures is shown. The activation free energies for the transformation **F** → **G** in the monomeric systems with Me, Ph, and Dipp substituents (^MMe^Δ*G*^⧧^, ^MPh^Δ*G*^⧧^, and ^MDipp^Δ*G*^⧧^), as determined through the corresponding **F** and **TS3** structures, are also presented.

The computation of the mechanism starting from **1** is challenging due to the absence of its experimental crystal structure. To address this, we examined the relative energies of different conformers of **1**, considering various orientations of the Et groups relative to the oxidiphenyl spacer. The most stable one (Figure 1A) was found to have the Et groups oriented toward the Ph groups of the linker. This structure positions the Zn atoms orthogonally due to the geometry of the linker, spatially separating the two metal centers by 11.1 Å. Although the two zinc centers in our bimetallic catalyst act independently and do not seem to exhibit cooperative interactions, the presence of two active sites allows the catalyst to process more substrate molecules simultaneously. However, since the activity per zinc center is comparable to that of monometallic catalysts, the overall advantage of the bimetallic system is not inherent but may be beneficial in specific contexts where catalyst loading per molecule is a consideration.

**Scheme 8:**
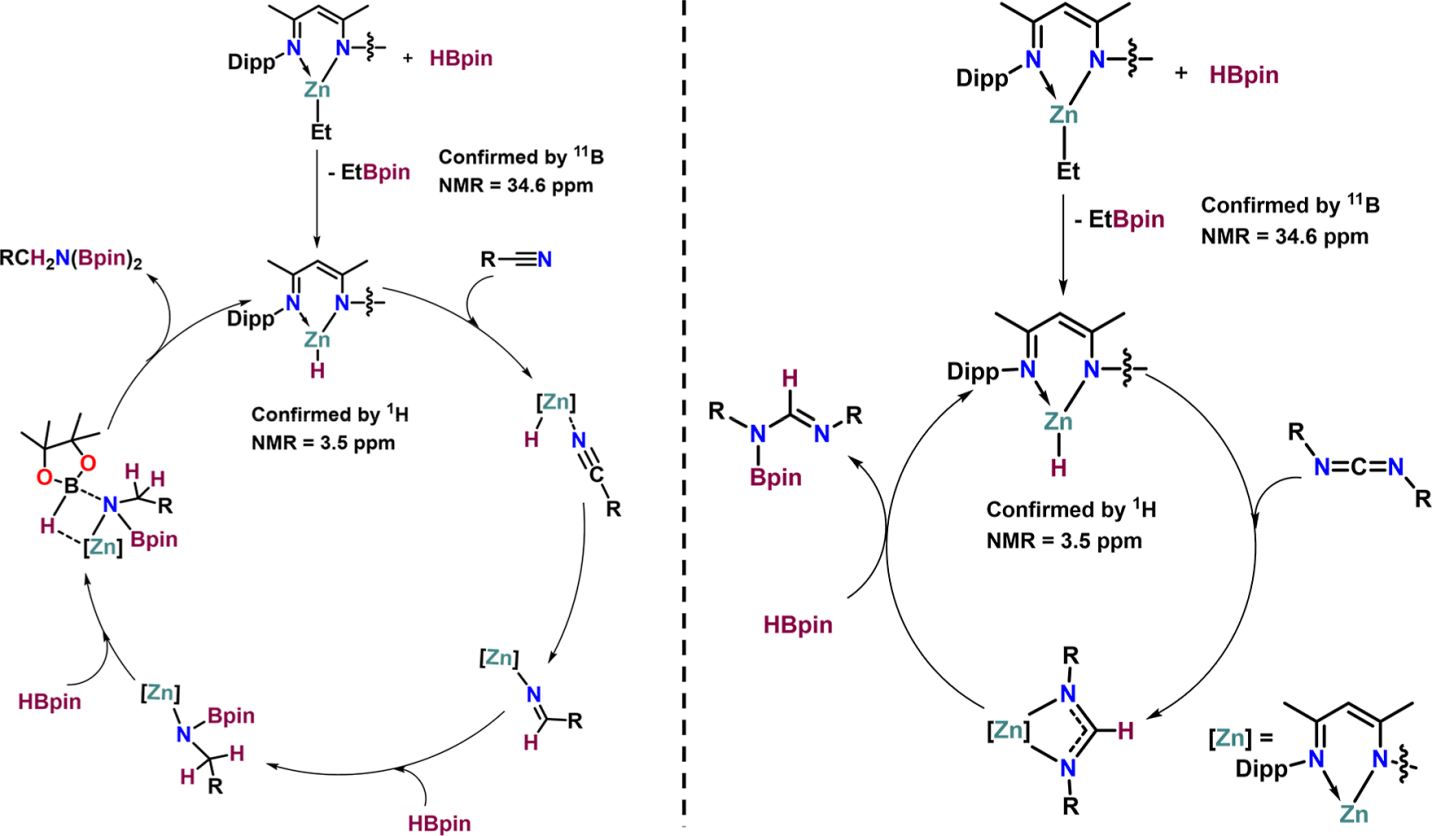
Proposed Mechanism (Adapted from Panda and Co-workers^22^) for the Hydroboration of Nitriles (Left) and Carbodiimides (Right) Catalyzed by Complex **1**

Charge analyses using the Hirshfeld, Mulliken, and Löwdin population methods^35^ confirmed that the Zn charge remains identical in both complex **1** and monomeric model units where the oxidiphenyl linker and terminal Dipp ligands were replaced either by Ph or Me groups. These simplified structures, referred to as **1**^**MPh**^ and **1**^**MMe**^, respectively, were subsequently employed for further mechanistic investigations into the hydroboration of nitriles, specifically PhCN, catalyzed by the tricoordinate zinc center. The rationale for selecting these model systems is 2-fold. First, it allows for more efficient computational calculations, which would be considerably more demanding with the full bimetallic system and bulky Dipp groups. Second, although the models may not account for all stabilizing effects—such as indirect contributions from the neighboring metal center—they still provide mechanistic insights into key catalytic steps. Notably, the energy differences observed between Me and Ph substituents could reveal important information about the influence of sterics and dispersion interactions throughout the reaction mechanism. This approach balances computational feasibility with a reasonable representation of the catalytic behavior, while recognizing that the full system may exhibit additional effects not accounted for in the simplified models. A plausible reaction mechanism derived from our DFT calculations is shown in Figure 1B (data for Me substituents; for Ph substituents, see Figure S108 in the Supporting Information), while our proposed catalytic cycle for the hydroboration of nitriles and carbodiimides mediated by **1**, which closely follows that of Panda and co-workers,^22^ is illustrated in Scheme 8.

Starting from **1**^**MMe**^, the initial step involves generating the intermediate **A**^**MMe**^, which is the tricoordinated zinc hydride analog of **1**^**MMe**^ and serves as the active catalyst. The formation of **A**^**MMe**^ is exergonic, with a free energy change of Δ*G* = −12.4 kcal mol^–1^. This zinc hydride intermediate is confirmed by ^1^H NMR data, showing a chemical shift at δ = 3.5 ppm (Figure S39 in the Supporting Information). Since the hydride is the active catalytic species, all other species in our mechanistic study have Δ*G* values relative to **A**^**MMe**^. The next step involves the insertion of PhCN into **A**^**MMe**^, which occurs without a kinetic barrier and results in **C**^**MMe**^ with an endergonic free energy value of Δ*G* = +4.8 kcal mol^–1^. The subsequent hydride transfer to the nitrile carbon atom occurs through the transition state **TS1**^**MMe**^, with an activation barrier of Δ*G*^#^ = 21.8 kcal mol^–1^, leading to **D**^**MMe**^ in an exergonic step, with **D**^**MMe**^ being at Δ*G* = −5.9 kcal mol^–1^ relative to **A**^**MMe**^.

Next, the attack of HBpin on the Zn–N bond of **D**^**MMe**^ forms the four-membered ZnNBO ring **E**^**MMe**^ through the transition state **TS2**^**MMe**^, which is located −6.2 kcal mol^–1^ below **A**^**MMe**^. This transformation is exergonic, with a free energy value of Δ*G* = −13.6 kcal mol^–1^.

Following a conformational change to **F**^**MMe**^, the hydride from the attached HBpin is transferred to the carbon atom of the imine group via the transition state **TS3**^**MMe**^. This structure represents the highest barrier in the mechanism (Δ*G*^⧧^ = 46.9 kcal mol^–1^), partially submerged in the reaction pathway and situated 27.9 kcal mol^–1^ above **A**^**MMe**^. Even with its partial submersion, this barrier remains substantial and could still impede the reaction in the monomeric system. When considering Ph substituents, a similar scenario is observed, with the barrier increasing to Δ*G*^⧧^ = 48.3 kcal mol^–1^ and **TS3**^**MPh**^ located 31.3 kcal mol^–1^ above **A**^**MPh**^ (see the Supporting Information, Figure S108).

To further evaluate the reliability of these findings, we extended our analysis by recalculating the energy profile using different levels of theory, including single-point calculations using the DFT functional ωB97X-D^36^ and the domain-based local pair natural orbital coupled cluster method with singles, doubles and perturbative triples, DLPNO-CCSD(T),^37^ both employing def2-TZVPP basis sets. These calculations corroborated the presence of a high barrier, reinforcing the validity of our original results and suggesting that the chosen methods are not responsible for the elevated energy values observed.

We then conducted an examination of this critical reaction step for the monomeric system with Dipp substituents. We observed a reduction in the activation free energy by 6.9 and 8.3 kcal mol^–1^ compared to the Me and Ph substituted models, respectively, lowering the barrier to 40.0 kcal mol^–1^. Although this decrease suggests that bulkier substituents can modulate the energetic landscape, particularly at this step, the resulting barrier remains significantly higher than what would be ideal for efficient catalysis under the given reaction conditions. This persistent high barrier underscores a critical limitation in the current mechanistic model and indicates that intrinsic factors within the monomeric system, such as steric or electronic effects, may be affecting the process. This finding points to the necessity for further investigation into other influences that could potentially lower the activation energy, such as solvent interactions, intermolecular effects, or conformational flexibility. Although such a thorough investigation lies beyond the scope of the current study, it offers promising avenues for future research to deepen our understanding and refine the reaction mechanism of organozinc complexes as catalysts for the hydroboration of organic nitriles.

Overcoming the hydrogen migration step associated with **TS3**^**MMe**^ results in the singly hydroborated intermediate **G**^**MMe**^, which is exergonic by −23.3 kcal mol^–1^ relative to **F**^**MMe**^ and −42.3 kcal mol^–1^ below **A**^**MMe**^. The addition of a second HBpin to **G**^**MMe**^ leads to the doubly hydroborated intermediate **H**^**MMe**^ through a minor energy barrier of Δ*G*^#^ = +3.8 kcal mol^–1^, associated with **TS4**^**MMe**^. This transition state is situated at Δ*G* = −38.5 kcal mol^–1^ below **A**^**MMe**^, while **H**^**MMe**^ forms exergonically by −7.1 kcal mol^–1^, placing it at Δ*G* = −49.4 kcal mol^–1^ relative to **A**^**MMe**^. Finally, the breaking of the Zn–N bond in **H**^**MMe**^ results in the formation of the product **1d** at Δ*G* = −54.4 kcal mol^–1^ and the regeneration of the zinc hydride catalyst.

**Figure 2:**
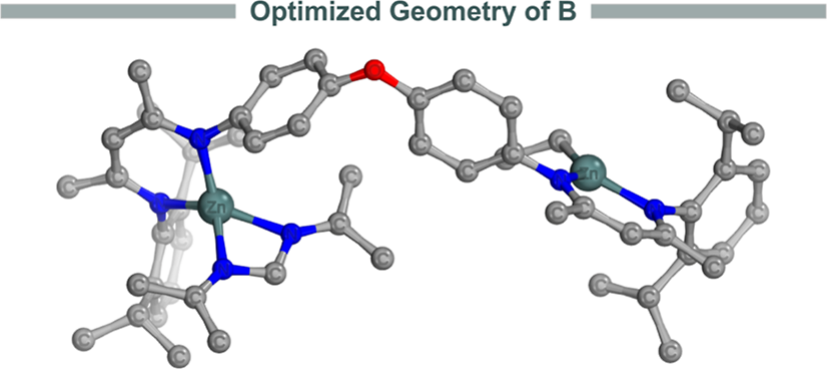
3D representation of the optimized structure of the spiro zinc intermediate **B** at the PBE0-D3(BJ)/def2-SVP level of theory. Hydrogen atoms are omitted for clarity.

Scheme 8 (left) presents a simplified version of the mechanistic proposal for the hydroboration of nitriles, using complex **1** as the precatalyst. The right panel of Scheme 8 illustrates the proposed mechanism for carbodiimides. Although we did not investigate the full pathway for the hydroboration of carbodiimides using a monomeric version of **1**, we have optimized the structure of the spiro zinc intermediate, as shown in Figure 2. Our results provide a rationale for the zinc-mediated hydroboration mechanisms of nitriles and carbodiimides, and underscore the potential relevance of bis(diiminate) ligands in zinc organometallic chemistry.

Based on previous reports published by Panda and co-workers,^22^ as well as theoretical calculations and experimental results, the proposed mechanism is depicted in Scheme 8. The reaction starts with zinc complex **1** reacting with HBpin to form a Zn-hydride species, which then coordinates with the nitrile group. This interaction facilitates nitrile reduction, resulting in the formation of a Zn-iminium intermediate. The iminium group undergoes σ-metathesis with HBpin, leading to the formation of a monoborate ester. The monoborate ester subsequently reacts with another HBpin molecule, generating a four-membered Zn species. This species rearranges, producing the 1,1'-diborylamine product and regenerating the active Zn-hydride species for further catalytic cycles. Similarly, the zinc hydride species reacts with carbodiimides, transferring the hydride from zinc to the carbon atoms of the carbodiimide, forming a four-membered spiro zinc complex. This spiro complex reacts with HBpin to yield the *N*-boryl formamidine product, while regenerating the active Zn-hydride species for continuous catalysis.

Having a series of ligands in our hands, we have synthesized complexes **2–4** by following a similar approach, obtaining them as yellow solids in good yields (see Scheme S2 in the Supporting Information). These complexes were characterized with ^1^H, ^13^C NMR spectroscopy and mass spectrometry. The ^1^H NMR spectra revealed triplets at 1.45 and 0.79 ppm for the methyl protons of complexes **2** and **4**. For complex **3**, two distinct triplet signals were observed at 0.70 and 0.67 ppm. Quartet signals at 0.75 and 0.15 ppm were assigned to the methylene protons of the Et groups attached to the zinc ion in complexes **2** and **4**. In the case of complex **3**, two separate quartet signals were evident at 0.15 and 0.11 ppm, corresponding to two methylene protons of the two Et groups. Additionally, complex **2**, exhibited a singlet at 5.27 ppm corresponding to the γ-methine protons. Complexes **3** and **4** showed doublets at 5.02 and 5.01 ppm, respectively, for their respective γ-methine protons. With these complexes in hand, we are now poised to investigate a diverse range of Zn-mediated catalytic reactions, which we plan to undertake in due course.

## Conclusions

To summarize, we synthesized a series of bis(diiminate) ligands and prepared the corresponding tricoordinated bimetallic zinc complexes **1–4** in good yields, which were characterized by NMR spectroscopy and mass spectrometry. We demonstrated that **1** serves as an efficient catalyst for the hydroboration of nitriles and carbodiimides. Our experimental and theoretical findings unveil the formation of a tricoordinated zinc hydride during the catalytic cycle. A plausible catalytic cycle has been proposed based on the in situ isolation of the active catalyst and supporting computational studies. Furthermore, the synthesized complexes **2**, **3**, and **4**, along with their corresponding ligands, hold potential for supporting zinc in diverse catalytic reactions. These studies are currently underway.

## Experimental Section

### Synthesis of Zinc Complexes (**1–4**)

#### General Synthesis of Complexes (**1–4**)

To a solution of ligands (**L1–L4**) in hexane, diethyl zinc (1 M solution in toluene) was added dropwise at room temperature and further stirred for 12 h. Volatiles were removed in vacuo to give corresponding zinc ethyl complexes **1–4** as yellow solids.

#### Oxydiphenyl Spacer Zinc Complexes (**1** and **2**)

**1**: Ligand **L1** (150 mg, 0.22 mmol) and diethyl zinc (0.66 mL, 0.66 mmol). Yield: 86%. ^1^H NMR (C_6_D_6_, 500 MHz): δ 7.11 (m, 6H, C*H*_Aryl_), 6.90 (d, *J* = 8.5 Hz, 4H, C*H*_Aryl_), 6.72 (d, *J* = 8.5 Hz, 4H, C*H*_Aryl_), 4.95 (s, 2H, C*H*_pentene_), 3.15 (m, 4H, C*H*_Dip_), 1.79 (s, 6H, C*H*_3_), 1.68 (s, 6H, C*H*_3_), 1.20 (d, *J* = 7 Hz, 12H, C*H*_3Dipp_), 1.14 (d, *J* = 7 Hz, 12H, C*H*_3Dipp_), 1.08 (t, 6H, C*H*_3ethyl_), 0.41 (q, 4H, C*H*_2ethyl_). ^13^C{^1^H} NMR (C_6_D_6_, 126 MHz): 166.91, 166.02, 153.99, 145.10, 144.18, 140.89, 125.46, 125.35, 123.14, 118.69, 95.70, 27.73, 23.61, 22.75, 22.70, 22.66, 11.67, −2.57. HR-MS (ESI): calculated for C_50_H_66_N_4_O_1_Zn_2_, 867.3892 [M + H]^+^; found, 867.3866.

**2**: Ligand **L2** (150 mg, 0.25 mmol) and diethyl zinc (0.75 mL, 0.75 mmol). Yield: 81%. ^1^H NMR (C_6_D_6_, 500 MHz): δ 6.90 (d, *J* = 8.5 Hz, 4H, C*H*_Aryl_), 6.82 (s, 4H, C*H*_Aryl_), 6.69 (d, *J* = 8.5 Hz, 4H, C*H*_Aryl_), 4.93 (s, 2H, C*H*_pentene_), 2.15 (s, 6H, C*H*_3Mes_), 2.11 (s, 12H, C*H*_3Mes_), 1.81 (s, 6H, C*H*_3_), 1.62 (s, 6H, C*H*_3_), 1.11 (t, 6H, C*H*_3ethyl_), 0.41 (q, 4H, C*H*_2ethyl_). ^13^C{^1^H} NMR (C_6_D_6_, 126 MHz): 167.22, 166.51, 154.65, 145.85, 145.54, 133.90, 130.79, 129.46, 126.17, 119.36, 96.50, 23.43, 22.48, 20.93, 18.78, 12.40, −2.26. HR-MS (ESI): calculated for C_44_H_54_N_4_O_1_Zn_2_, 783.2953 [M + H]^+^; found, 783.2910.

#### Naphthalene Spacer Complexes (**3** and **4**)

**3**: Ligand **L3** (150 mg, 0.23 mmol) and diethyl zinc (0.70 mL, 0.70 mmol). Yield: 86%. ^1^H NMR (C_6_D_6_, 500 MHz): δ 7.95–7.89 (m, 2H, C*H*_Aryl_), 7.32–7.26 (m, 2H, C*H*_Aryl_), 7.11 (s, 6H, C*H*_Ary_l), 7.0 (m, 2H, C*H*_Aryl_), 5.04 (s, 1H, C*H*_pentene_), 5.01 (s, 1H, C*H*_pentene_), 3.27–3.21 (m, 4H, C*H*), 1.76–1.68 (m, 12H, C*H*_3_), 1.25–1.20 (m, 24H, C*H*_3_), 0.72–0.66 (m, 6H, C*H*_3ethyl_), 0.19–0.15 (m, 4H, C*H*_2ethyl_). ^13^C{^1^H} NMR (C_6_D_6_, 126 MHz): 167.89, 167.59, 167.05, 147.52, 144.92, 144.86, 141.73, 141.66, 141.58, 141.54, 131.27, 126.35, 126.09, 123.97, 123.81, 121.45, 121.31, 119.90, 96.27, 96.17, 28.59, 28.52, 24.60, 24.51, 24.30, 24.25, 23.63, 23.46, 23.34, 23.30, 23.16, 22.97, 12.01, 11.87, −2.04, −2.11. HR-MS (ESI): calculated for C_48_H_64_N_4_Zn_2_, 825.3787 [M + H]^+^; found, 825.3795.

**4**: Ligand **L4** (150 mg, 0.27 mmol) and diethyl zinc (0.81 mL, 0.81 mmol). Yield: 79%. ^1^H NMR (C_6_D_6_, 500 MHz): δ 7.93 (m, 2H, C*H*_Aryl_), 7.27 (m, 2H, C*H*_Aryl_), 6.96 (d, *J* = 7 Hz, 2H, C*H*_Aryl_), 6.80 (d, *J* = 8 Hz, 4H, C*H*_Aryl_), 5.02 (s, 1H, C*H*_pentene_), 5.00 (s, 1H, C*H*_pentene_), 2.18 (s, 6H, C*H*_3Mes_), 2.13 (s, 12H, C*H*_3Mes_), 1.78 (s, 3H, C*H*_3_), 1.70 (s, 3H, C*H*_3_), 1.66 (s, 6H, C*H*_3_), 0.79 (t, 6H, C*H*_3ethyl_), 0.20 (q, 4H, C*H*_2ethyl_). ^13^C{^1^H} NMR (C_6_D_6_, 126 MHz): 167.56, 167.51, 166.97, 147.62, 145.52, 145.47, 133.89, 131.22, 130.80, 129.47, 126.37, 121.37, 121.26, 119.95, 96.29, 96.18, 22.99, 22.55, 20.93, 18.79, 18.76, 11.99, −2.64, −2.69. LC–MS (ESI): calculated for C_42_H_52_N_4_Zn_2_, 781.3035 [M + CH_3_CN]^+^; found, 781.2104.

### Catalytic Studies

#### General Procedure for the Hydroboration of Nitriles

10 mg (0.01 mmol, i.e. 5 mol %) of complex **1** was dissolved in 0.5 mL of C_6_D_6_. 116.08 μL (0.8 mmol) of pinacolborane was then added followed by 0.4 mmol of nitrile. This mixture was then transferred to a J Young NMR tube and the reaction was kept in an oil bath at 60 °C. The progress of the reaction was regularly monitored by ^1^H and ^11^B NMR spectroscopy until complete conversion was observed. The corresponding 1,1′-diborylamines **1a–1k** were isolated in 67–97% yields.

#### General Procedure for the Hydroboration of Carbodiimides

6 mg (0.01 mmol, i.e. 5 mol %) of complex **1** was dissolved in 0.5 mL of C_6_D_6_. 81 μL (0.8 mmol) of pinacolborane was then added followed by 0.14 mmol of carbodiimide. This mixture was transferred to a J Young NMR tube and kept at room temperature for 4 h, except for dipp-carbodiimides which was heated at 60 °C for 30 h. The progress of the reaction was regularly monitored by ^1^H and ^11^B NMR spectroscopy until complete conversion of the starting material was observed. The corresponding *N*-boryl formamidine derivatives **2a–2d** were isolated in 85–95% yields.

## Supporting Information Available

Crystallographic data of **L3** (CIF)
All experimental procedures, details of the DFT calculations, and additional data can be found in the Supporting Information (PDF)

## Supplementary Material

ao4c08068_si_001.cif
