# Supplementary material for: Bis(diiminate)-Supported Bimetallic Complexes: Tri-Coordinated Zinc for Nitrile and Carbodiimide Hydroboration
Source: ACS Omega. 2025 Jan 10;10(2):2033–43. doi: 10.1021/acsomega.4c08068 (PMC11755180; doi:10.1021/acsomega.4c08068)
Supplement: Supplementary file 2 — ao4c08068_si_002.pdf [file ao4c08068_si_002.pdf]

Supplementary Information

for

Bis(diiminate)-Supported Bimetallic Complexes: Tri-Coordinated Zinc for Nitrile and Carbodiimide Hydroboration

Darakshan Parveen,[a] Rahul Kumar Yadav,[a] Felipe Fantuzzi,[b] Dipak Kumar Roy*[a]*

[a] Department of Chemistry, Indian Institute of Technology Indore, Khandwa Road, Simrol, 453552, Indore, India.

[b] School of Chemistry and Forensic Science, University of Kent, Park Wood Rd, Canterbury CT2 7NH, UK.

Table of Contents

General Methods S3

Synthetic Details S4

General synthesis of ligands (L1-L4) S4

Oxydiphenyl spacer ligands (L1 and L2) S4

Naphthalene spacer ligands (L3 and L4) S5

Synthesis of mononuclear zinc complex S6

Mass and NMR spectra of ligands and complexes S7

General procedure for optimization of hydroboration of nitriles using benzonitrile as substrate. S210

NMR data of optimized hydroboration reaction of benzonitrile with 1,3,5-trimethoxybenzene as an internal standard (IS) S210

General procedure for hydroboration of nitriles S27

General procedure for hydroboration of carbodiimides S33

Experimental data to propose mechanistic pathway of hydroboration of benzonitrile S35

NMR spectra for hydroboration of nitriles S38

NMR spectra for hydroboration of carbodiimide S62
Crystallographic Details S72
Computational Details S73
Cartesian Coordinates S78
References S140

General Methods

The reactions were performed either in an Argon-filled Glove box or using standard Schlenk techniques. Chemicals were purchased from Spectrochem, Sigma-Aldrich, and TCI and used as received. All the solvents were purified by distillation using the appropriate drying agents, deoxygenated using three freeze–pump–thaw cycles, and stored over molecular sieves under argon prior to use. The deuterated solvents used for NMR spectroscopy were deoxygenated by freeze–pump–thaw cycles and stored under an argon atmosphere over molecular sieves. NMR chemical shifts are reported in ppm and coupling constants in Hz. ^{1}H, ^{11}B, and ^{13}C NMR spectroscopy data were obtained at ambient temperature using a Bruker 500 NMR spectrometer (operating at 500 MHz for ^{1}H, 126 MHz for ^{13}C and 160 MHz for ^{11}B). ^{1}H NMR spectra were referenced via residual proton resonances of $CDCl_3$ (^{1}H, 7.26 ppm) and C_6D_6 (^{1}H, 7.16 ppm). ^{13}C NMR spectra were referenced to $CDCl_3$ (^{13}C, 77.16) and C_6D_6 (^{13}C, 128.06 ppm) . HR-MS spectra were obtained by Bruker microTOF-Q II Daltonik. The X-ray diffraction data collections were performed on a CCD Agilent Technologies (Oxford Diffraction) SUPER NOVA (Mo at home/near, Eos) diffractometer. Data for the compounds were collected at 293 K using graphite-monochromated MoK_{α} radiation. DFT calculations were computed at the PBE0-D3(BJ)/def2-TZVPP+SMD(Benzene) level of theory using geometries optimized at the PBE0-D3(BJ)/def2-SVP level.

Synthetic Details

General synthesis of ligands (L1-L4)

To a solution of 2-hydroxy-4-imino-2-pentene in DCM (10 mL) the solution of triethyloxonium tetrafluoroborate in dichloromethane was added under an argon atmosphere. The mixture was stirred at room temperature for 12 h. An equimolar portion of triethylamine was slowly added to the resulting solution. After being stirred for another 20 min, the resulting mixture was added to a solution of diamines in triethylamine (10 mL). The resulting mixture was stirred at room temperature for an additional 48 h. The volatiles were removed under vacuum, resulting in an oily solid. Toluene was added to the resulting residue and filtered. Toluene was removed and the obtained solid was recrystallized from methanol at -20 °C (Scheme S1).

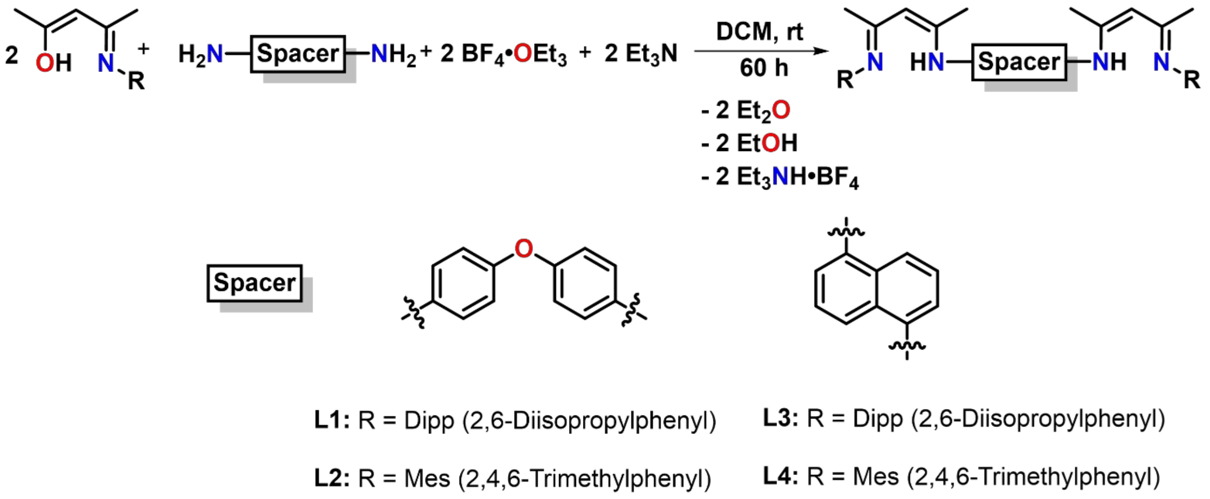

Scheme S1: Synthesis of oxy-diphenyl and naphthalene spacer ligands (**L1-L4**).

Oxydiphenyl spacer ligands (L1 and L2)

L1: 2-hydroxy-4-(2,6-diisopropylphenyl)imino-2-pentene (1 g, 3.86 mmol), triethyloxonium tetrafluoroborate (0.73 g, 3.86 mmol), triethylamine (0.391 g, 3.86 mmol) and 4,4-oxidianiline (0.386 g, 1.93 mmol). Yield: 73%

^{1}**H NMR** ($CDCl_3$, 500 MHz): δ 12.62 (s, 2H, N*H*), 7.12 (m, 6H, CH_{Aryl}), 6.91-6.87 (m, 8H, CH_{Aryl}), 4.86 (s, 2H, C$H_{pentene}$), 2.99 (m, 4H, C*H*), 2.02 (s, 6H, CH_3), 1.69 (s, 6H, CH_3), 1.20 (d, J = 7 Hz, 12H, CH_3), 1.13 (d, J = 6.5 Hz, 12H, CH_3). ^{13}**C{^{1}H} NMR** ($CDCl_3$, 126 MHz): 162.81, 157.95, 153.63, 142.44, 141.18,140.07, 124.65, 124.34, 123.08, 119.14, 95.54, 28.42, 24.32, 22.81, 21.14, 20.79. **HR-MS (ESI):** Calculated for $C_{46}H_{58}O_1N_4$: 683.4683 $[M+H]^+$, found: 683.4699.

L2: 2-hydroxy-4-(2,4,6-trimethylphenyl)imino-2-pentene (1 g, 4.6 mmol), triethyloxonium tetrafluoroborate (0.87 g, 4.6 mmol), triethylamine (0.46 g, 4.6 mmol), and 4,4-oxidianiline (0.46 g, 2.3 mmol). Yield: 82%

^{1}**H NMR** ($CDCl_3$, 500 MHz): δ 12.45 (s, 2H, N*H*), 6.92-6.88 (m, 8H, CH_{Aryl}), 6.86 (s, 4H, CH_{Aryl}), 4.85 (s, 2H, C$H_{pentene}$), 2.26 (s, 6H, C$H_{3\ Mes}$), 2.10 (s, 12H, C$H_{3\ Mes}$), 2.01 (s, 6H, CH_3), 1.67 (s, 6H, CH_3). ^{13}**C{^{1}H} NMR** ($CDCl_3$, 126 MHz): 162.20, 158.39, 153.56, 142.14, 140.55, 133.41, 131.10, 128.57, 124.23, 119.06, 95.70, 20.91, 20.78, 20.64, 18.47. **HR-MS (ESI):** Calculated for $C_{40}H_{46}O_1N_4$: 599.3744 $[M+H]^+$, found: 599.3757.

Naphthalene spacer ligands (L3 and L4)

L3: 2-hydroxy-4-(2,6-diisopropylphenyl)imino-2-pentene (1 g, 3.86 mmol), triethyloxonium tetrafluoroborate (0.73 g, 3.86 mmol), triethylamine (0.39 g, 3.86 mmol) and 1,5-naphthalenediamine (0.31 g, 1.93 mmol). Yield: 76%

^{1}**H NMR** ($CDCl_3$, 500 MHz): δ 12.62 (s, 2H, N*H*), 7.74 (d, J = 8.5 Hz, 2H, CH_{Aryl}), 7.32 (t, 2H, CH_{Aryl}), 7.12 (m, 6H CH_{Aryl}), 6.97 (d, J = 7.5 Hz, 2H, CH_{Aryl}), 4.96 (s, 2H, C$H_{Pentene}$), 3.17(sept, 4H, CH_{Dipp}), 1.90 (s, 6H, CH_3), 1.74 (s, 6H, CH_3), 1.24 (d, J = 6.5 Hz, 12H, CH_{3Dipp}), 1.13 (d, J = 6.5 Hz, 12H, CH_{3Dipp}). ^{13}**C{^{1}H} NMR** ($CDCl_3$, 126 MHz): 162.27, 160.46, 143.75, 143.39, 139.53, 129.57, 125.79, 125.35, 123.16, 119.38, 118.61, 95.07, 28.5, 24.56, 22.98, 21.03, 20.67. **HR-MS (ESI):** Calculated for $C_{40}H_{54}N_4$: 641.4578 $[M+H]^+$, found: 641.4578.

L4: 2-hydroxy-4-(2,4,6-trimethyl phenyl)imino-2-pentene (1.2 g, 5.52 mmol), triethyloxonium tetrafluoroborate (1.05 g, 5.52 mmol), triethylamine (0.56 g, 5.52 mmol) and 1,5-naphthalenediamine (0.44 g, 2.76 mmol). Yield: 72%

^{1}H NMR ($CDCl_3$, 500 MHz): δ 12.41 (s, 2H, N*H*), 7.73 (d, J = 8.5 Hz, 2H, CH_{Aryl}), 7.34 (t, 2H, CH_{Aryl}), 6.97 (d, J = 7 Hz, 2H, CH_{Aryl}), 6.86 (s, 4H, CH_{Aryl}), 4.96 (s, 2H, $CH_{pentene}$), 2.25 (s, 6H, CH_{3Mes}), 2.20 (s, 12H, CH_{3Mes}), 1.89 (s, 6H, CH_3), 1.73 (s, 6H, CH_3). **^{13}C{^{1}H} NMR** ($CDCl_3$, 126 MHz): 162.7, 159.7, 144.35, 139.30, 134.50, 133.21, 129.44, 128.59, 125.26, 119.19, 118.23, 95.14, 21.0, 20.92, 20.21, 18.50. **HR-MS (ESI):** Calculated for $C_{38}H_{44}N_4$: 557.3639 $[M+H]^+$, found: 557.3653.

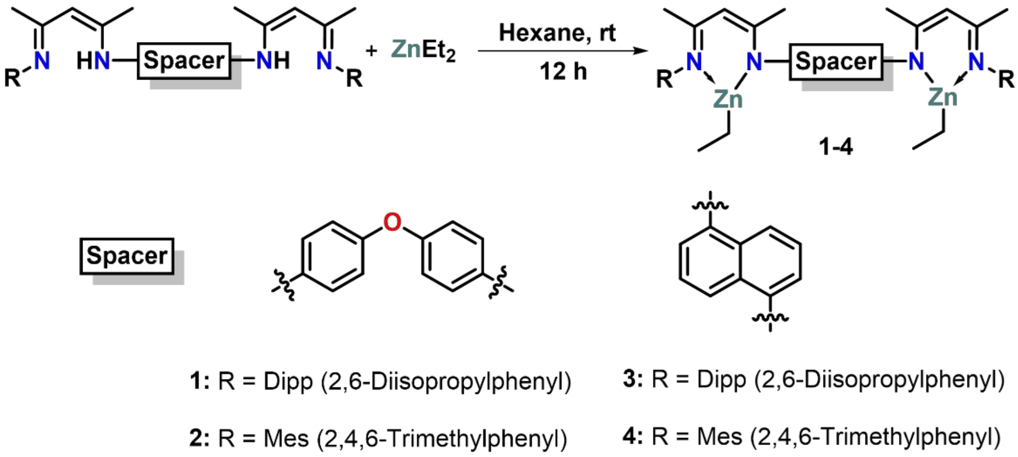

Scheme S2. Synthesis of oxy-diphenyl and naphthalene spacer complexes **1-4**.

Synthesis of monomeric zinc complex:

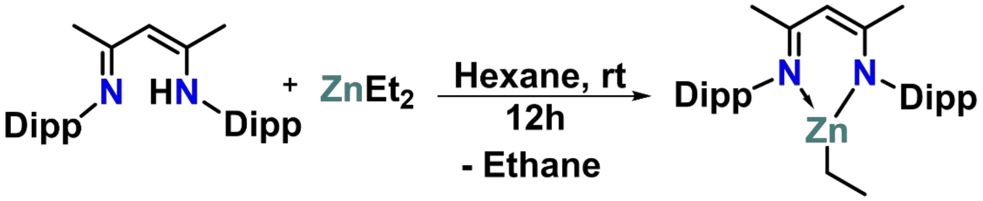

Scheme S3. Synthesis of monomeric zinc complex.

Zinc complex synthesized by modifying the reported procedure.[1] To a solution of ligand in hexane, diethyl zinc (1M solution in toluene) was added dropwise at room temperature and further stirred for 12 h. Volatiles were removed in vacuo to give corresponding zinc ethyl complex. The obtained product was confirmed by matching the ^{1}H and ^{13}C NMR spectra with the reported data.[1]

^{1}H NMR (C_6D_6, 500 MHz): δ 7.05 (d, J = 7.62 Hz, 4H, CH_{Aryl}), 6.89 (t, 2H, CH_{Aryl}), 4.90 (s, 1H, C$H_{pentene}$), 2.64 (m, 4H, C*H*), 1.45 (s, 6H, CH_3), 1.14 (d, J = 6.81 Hz, 24H, CH_3), 1.04 (t, 3H, CH_2CH_{3ethyl}), 0.58 (q, 2H, CH_2CH_{3ethyl}). **^{13}C{^{1}H} NMR** (C_6D_6, 126 MHz): 172.79, 141.21, 129.49, 123.96, 98.04, 28.39, 24.09, 23.37, 12.25, -1.60.

Mass and NMR spectra of ligands and complexes

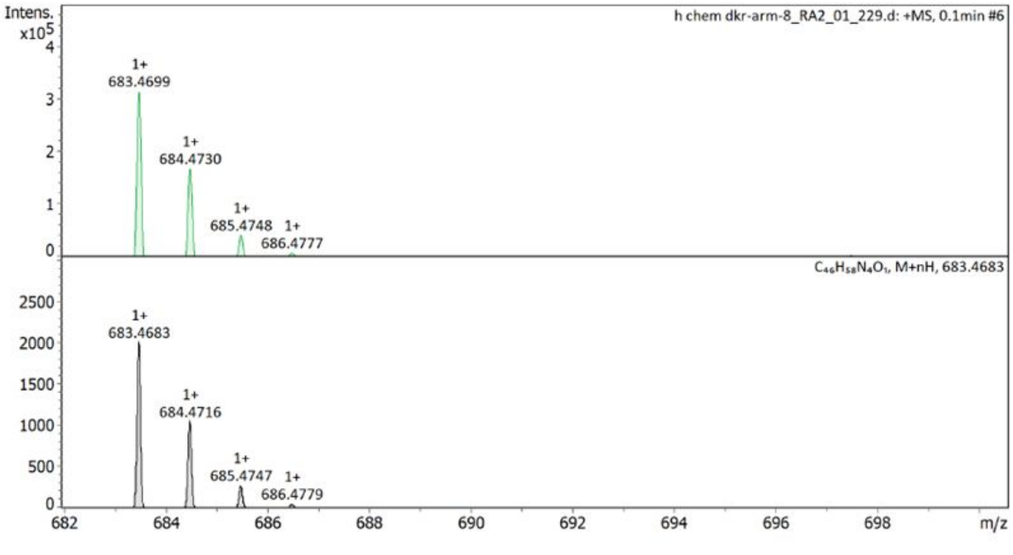

Figure S1. High-resolution mass spectrum of ligand **L1**. Calculated isotope pattern for $[M+H]^+$.

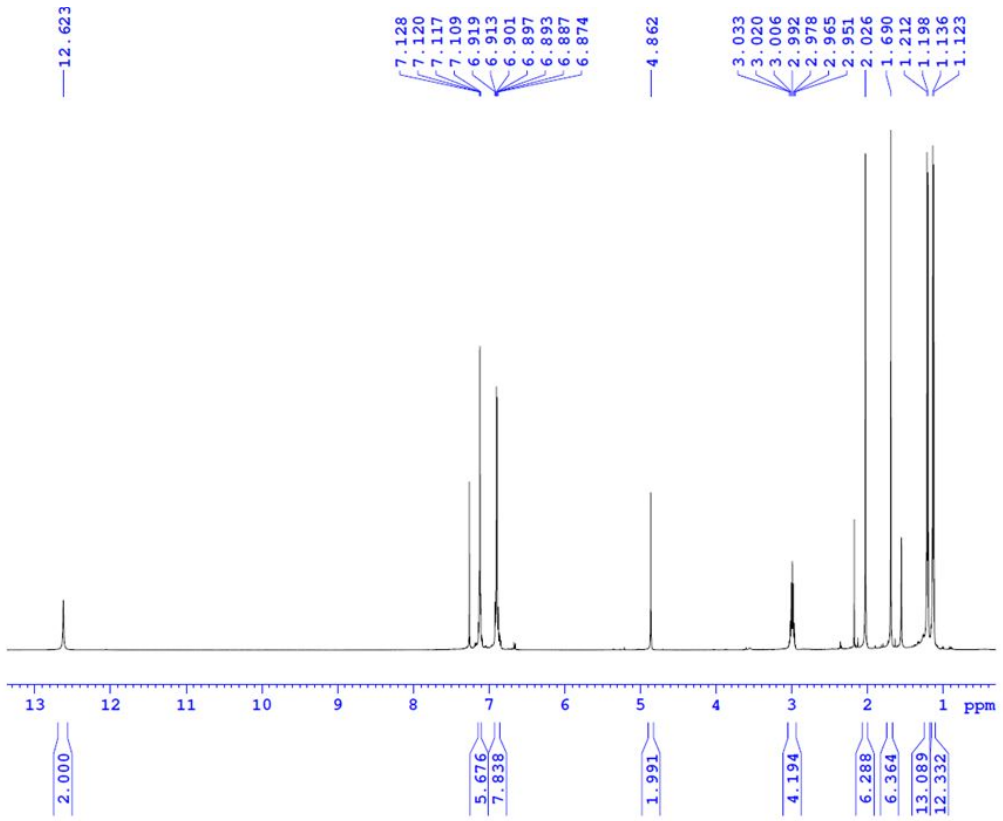

Figure S2. ^{1}H NMR spectrum of ligand **L1**.

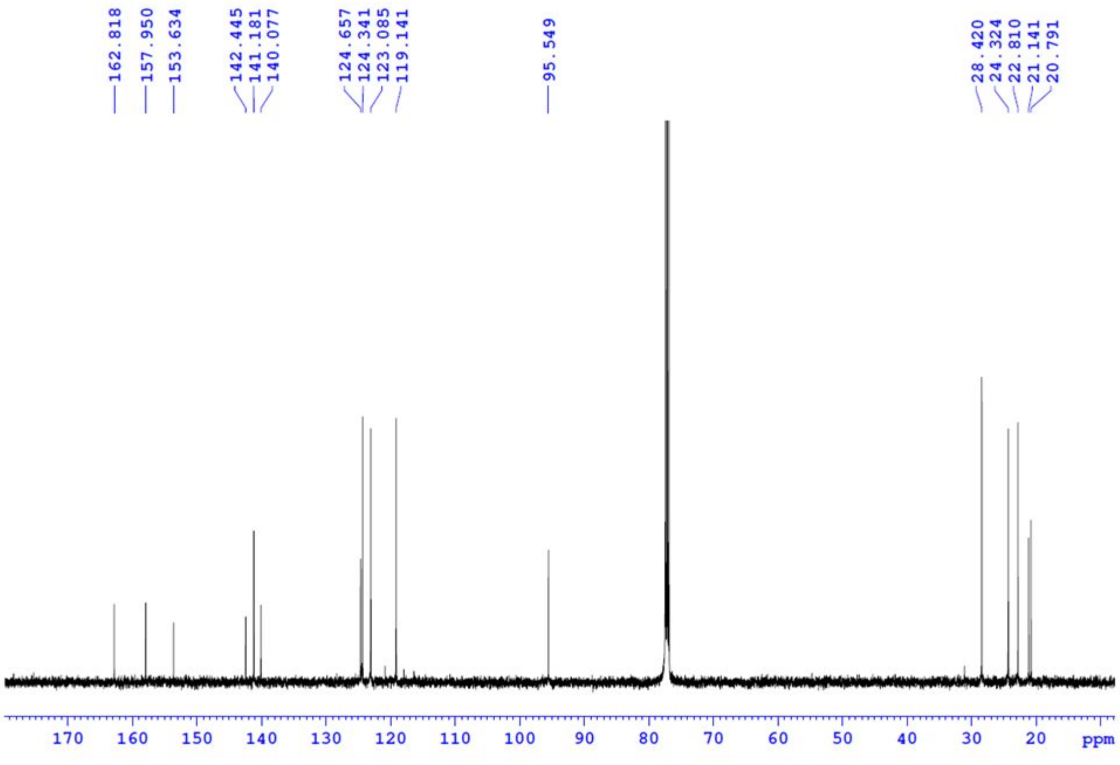

Figure S3. $^{13}C\{^{1}H\}$ NMR spectrum of ligand **L1**.

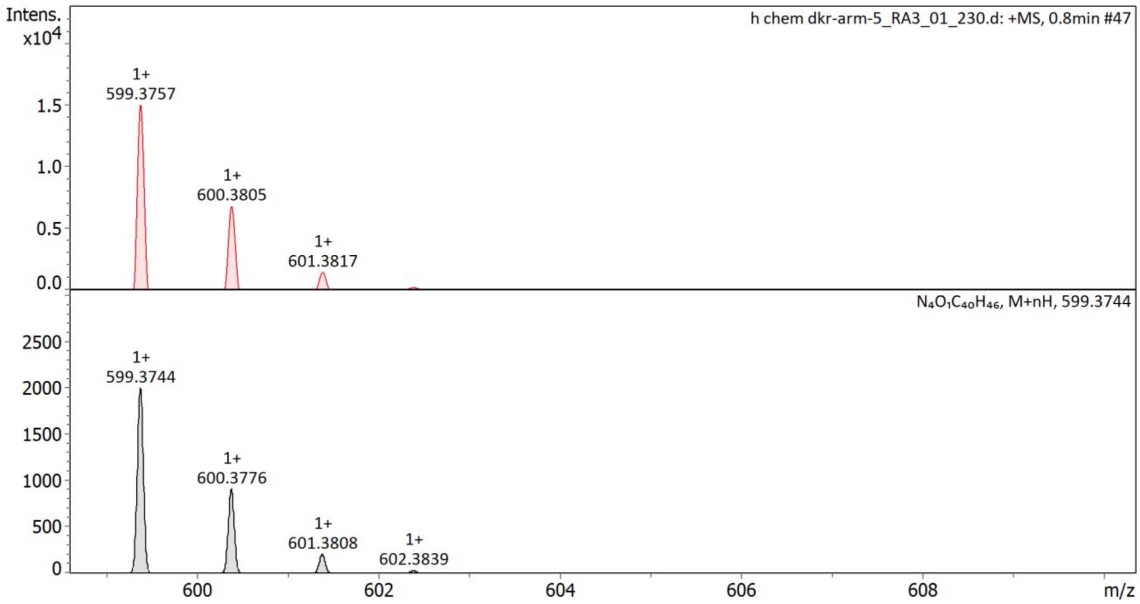

Figure S4. High-resolution mass spectrum of ligand **L2**. Calculated isotope pattern for $[M+H]^{+}$.

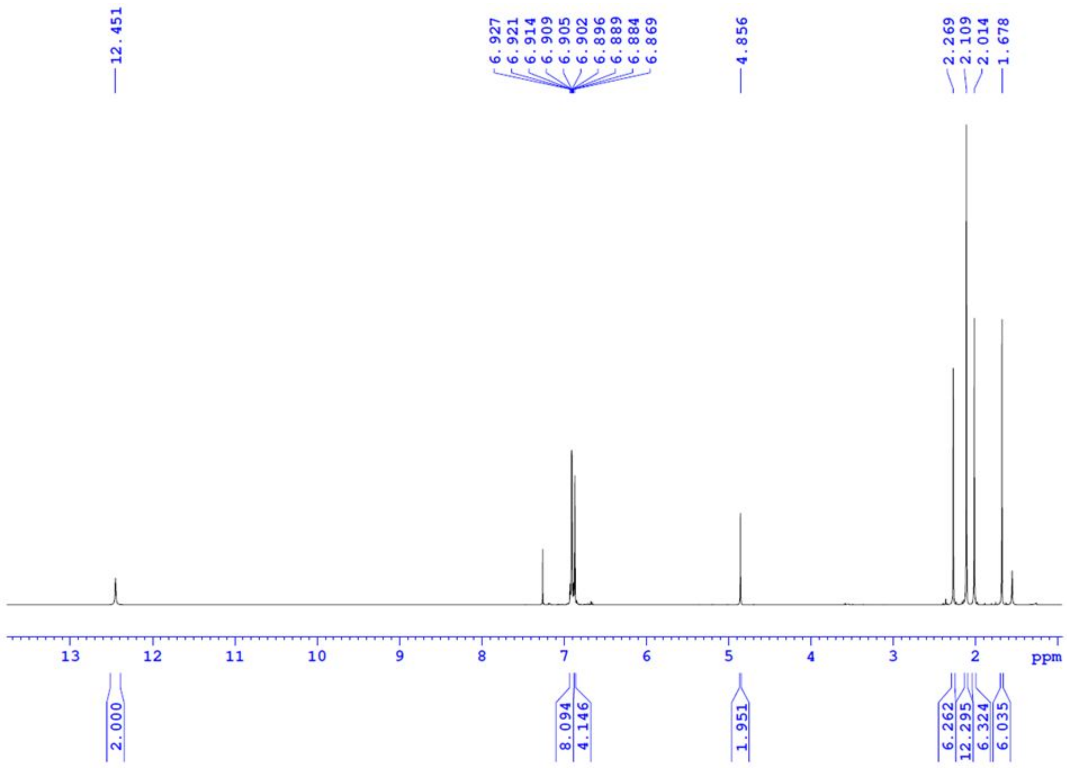

Figure S5. ^{1}H NMR spectrum of ligand **L2.**

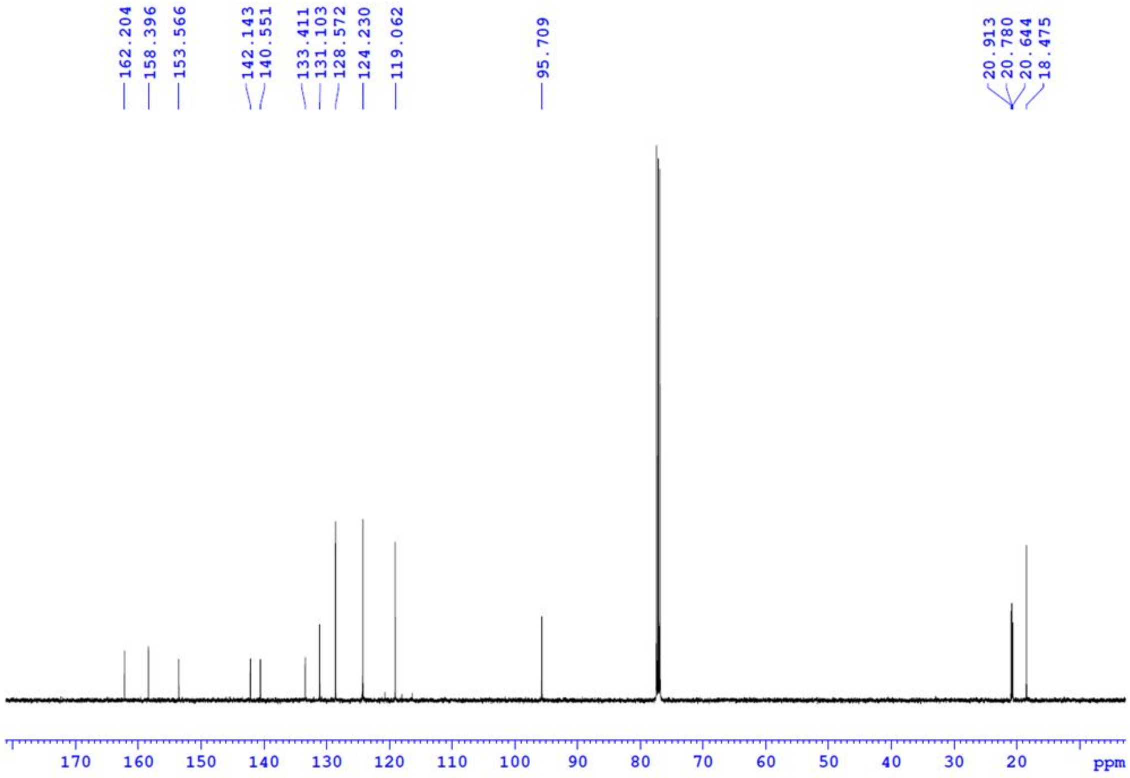

Figure S6. $^{13}C\{^{1}H\}$ NMR spectrum of ligand **L2**.

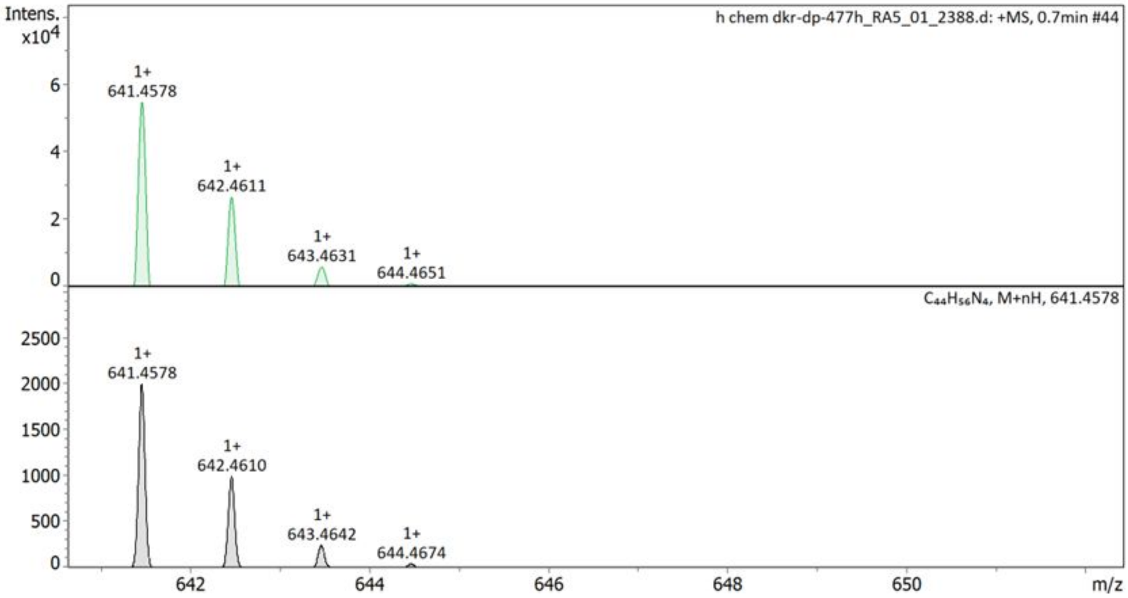

Figure S7. High-resolution mass spectrum of ligand **L3**. Calculated isotope pattern for $[M+H]^+$.

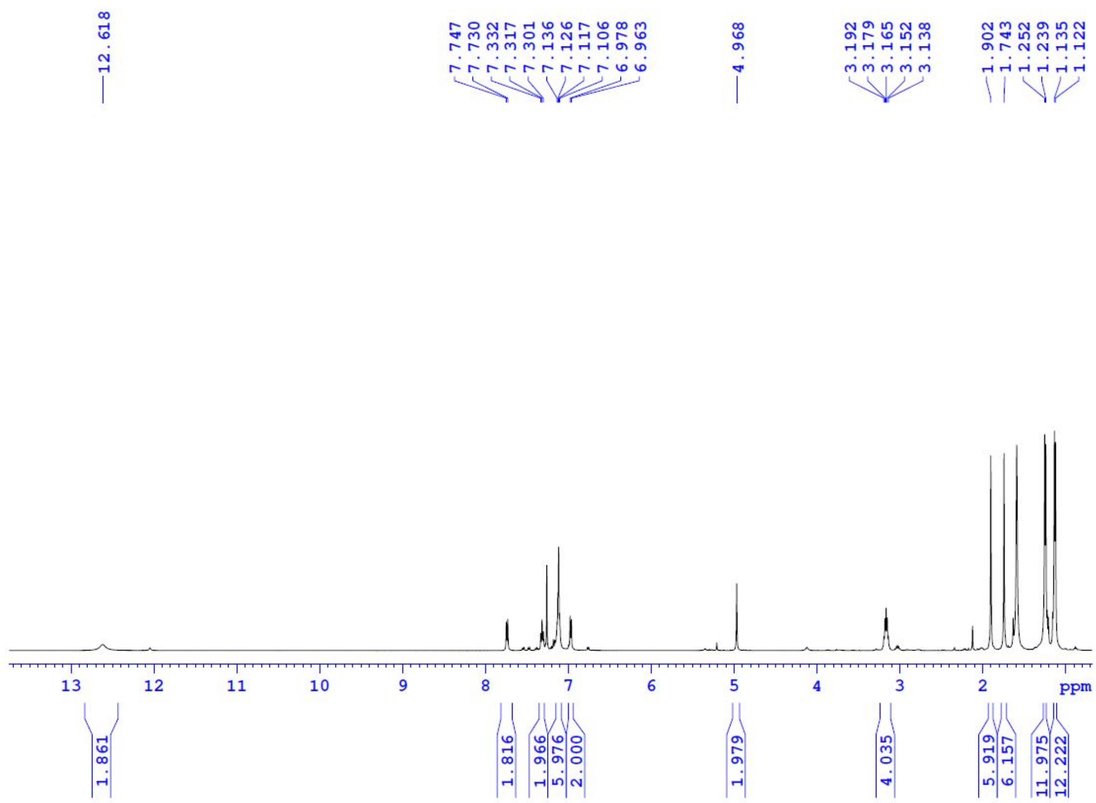

Figure S8. ^{1}H NMR spectrum of ligand **L3.**

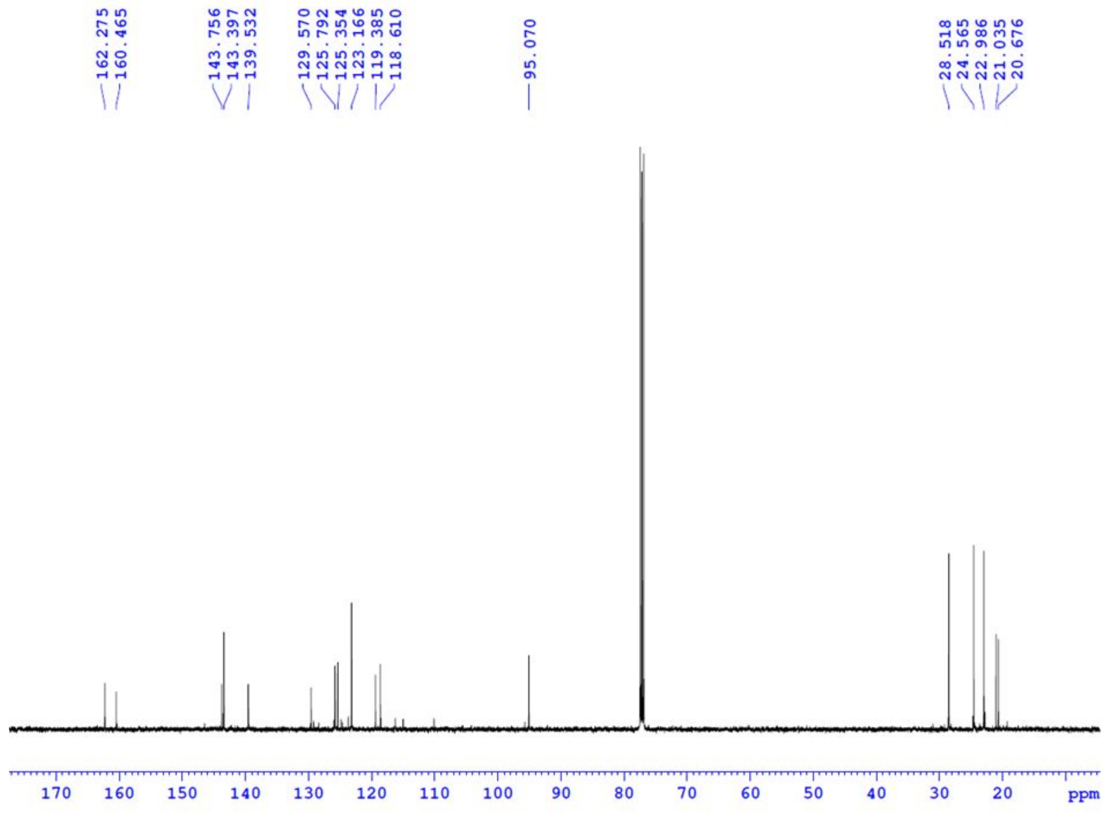

Figure S9. $^{13}C\{^{1}H\}$ NMR spectrum of ligand **L3**.

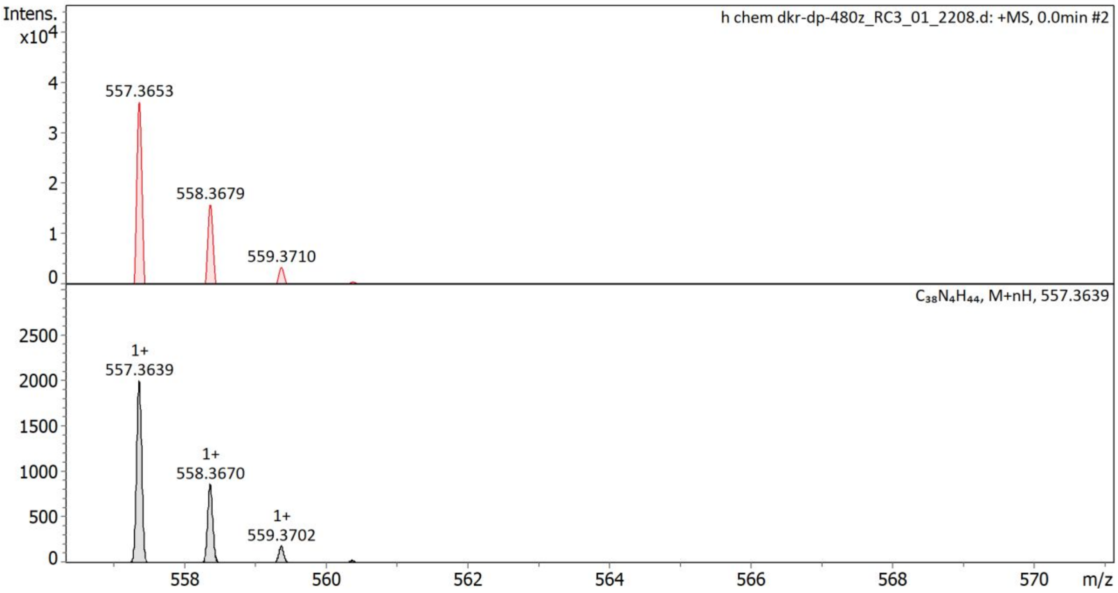

Figure S10. High-resolution mass spectrum of ligand **L4**. Calculated isotope pattern for $[M+H]^+$.

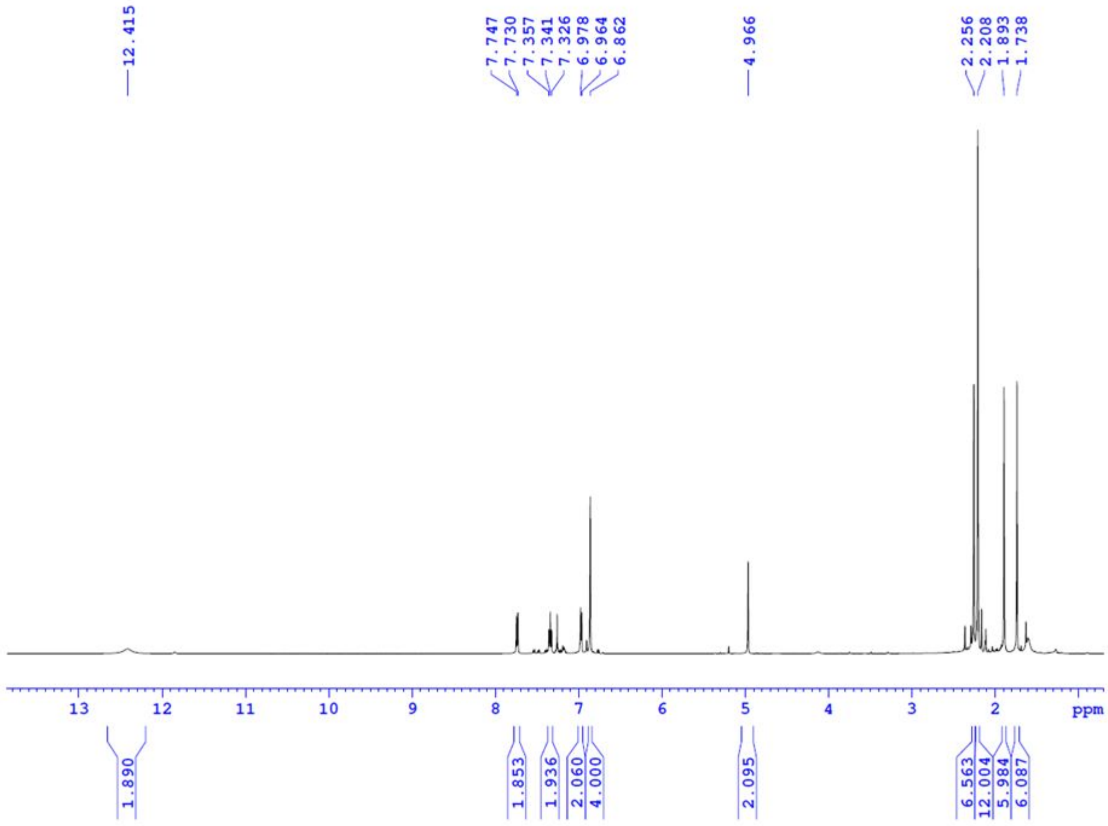

Figure S11. ^{1}H NMR spectrum of ligand **L4.**

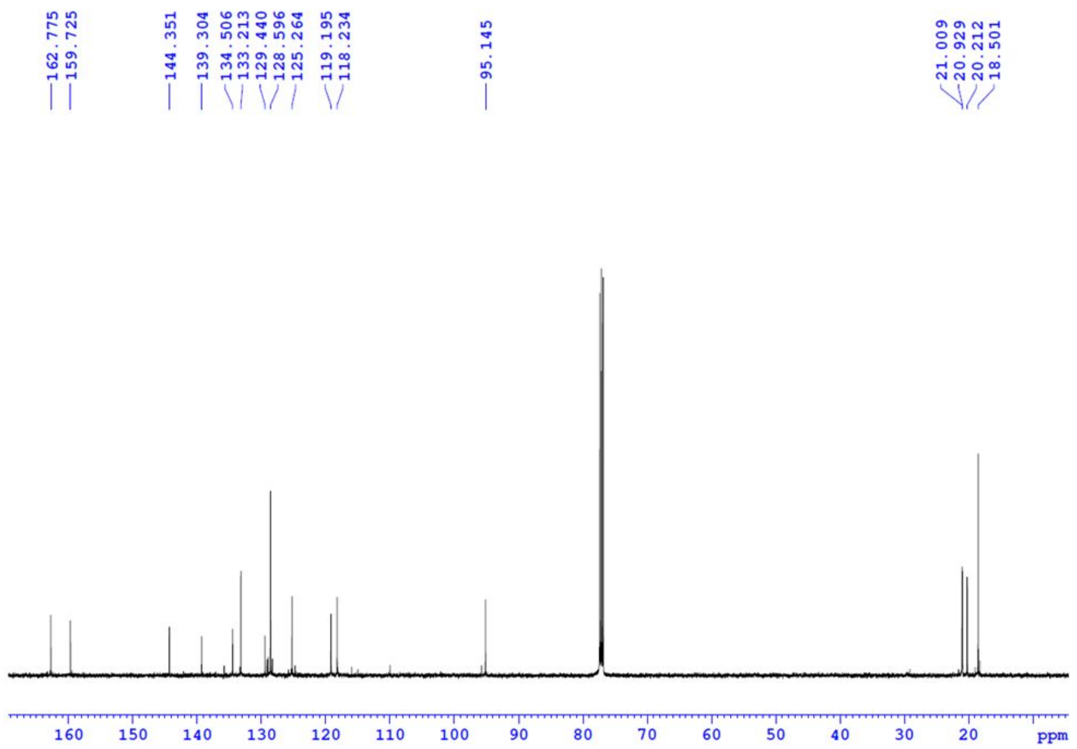

Figure S12. $^{13}C\{^1H\}$ NMR spectrum of ligand **L4.**

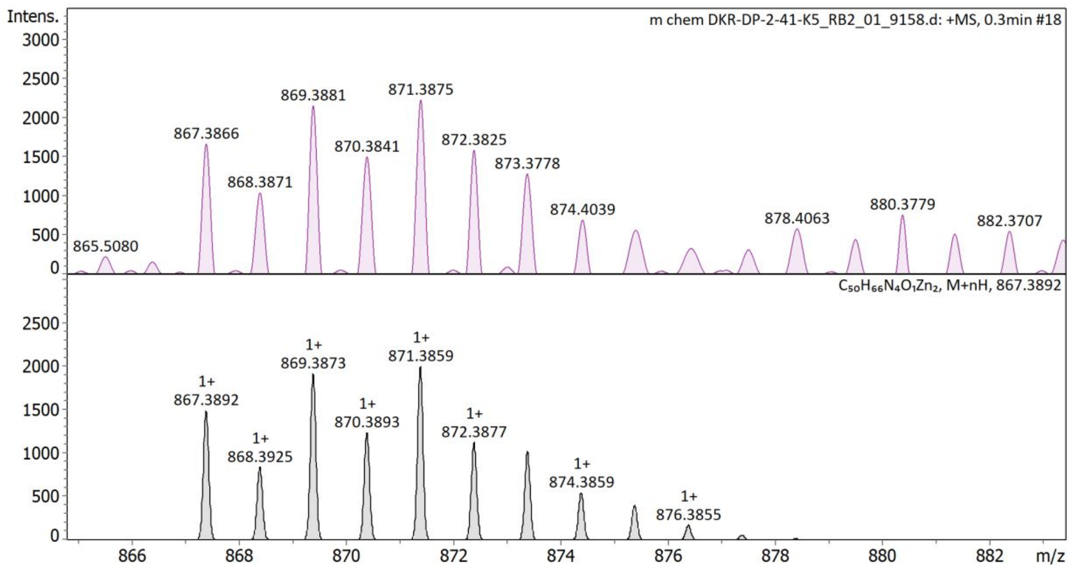

Figure S13. High-resolution mass spectrum of complex **1**. Calculated isotope pattern for $[M+H]^+$.

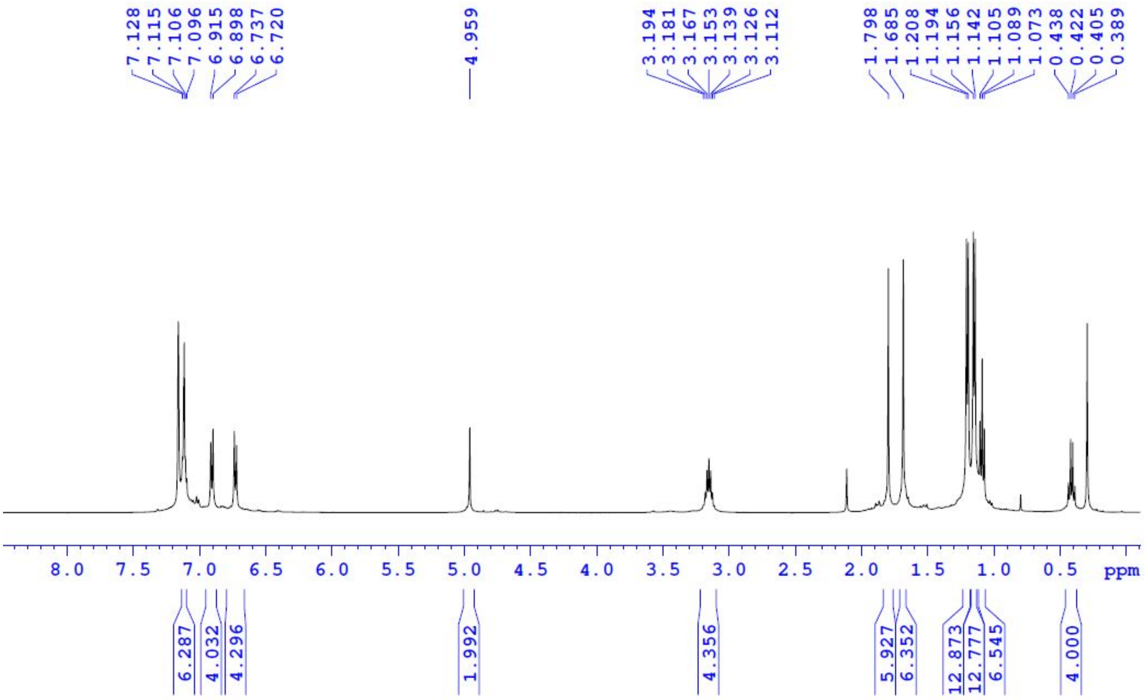

Figure S14. ^{1}H NMR spectrum of **1.**

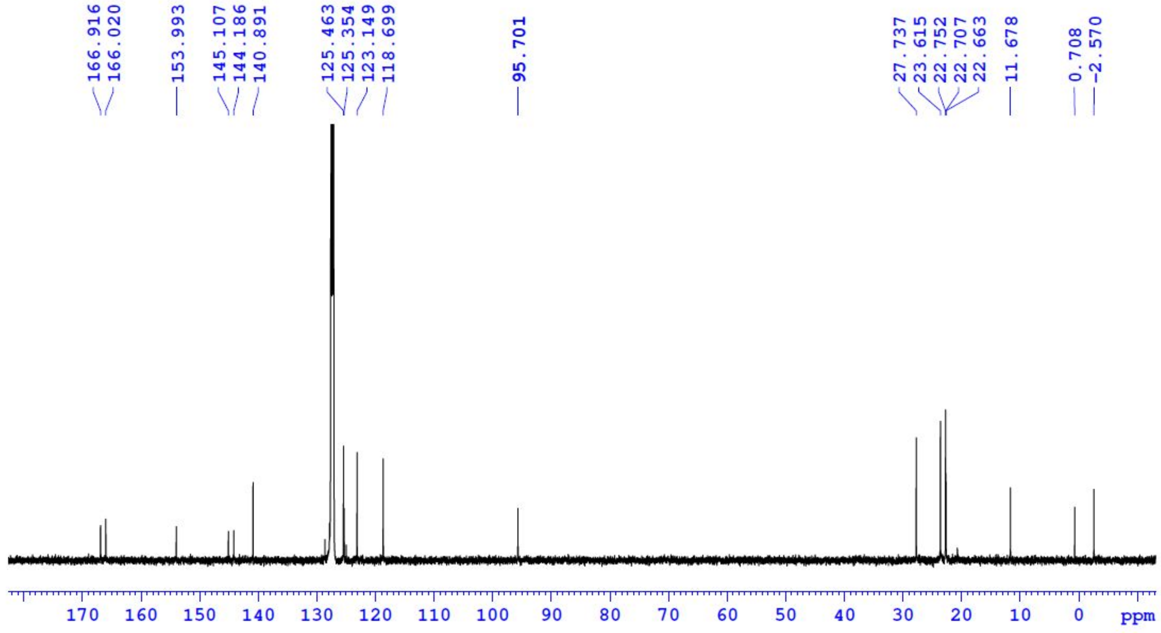

Figure S15. $^{13}C\{^{1}H\}$ NMR spectrum of **1.**

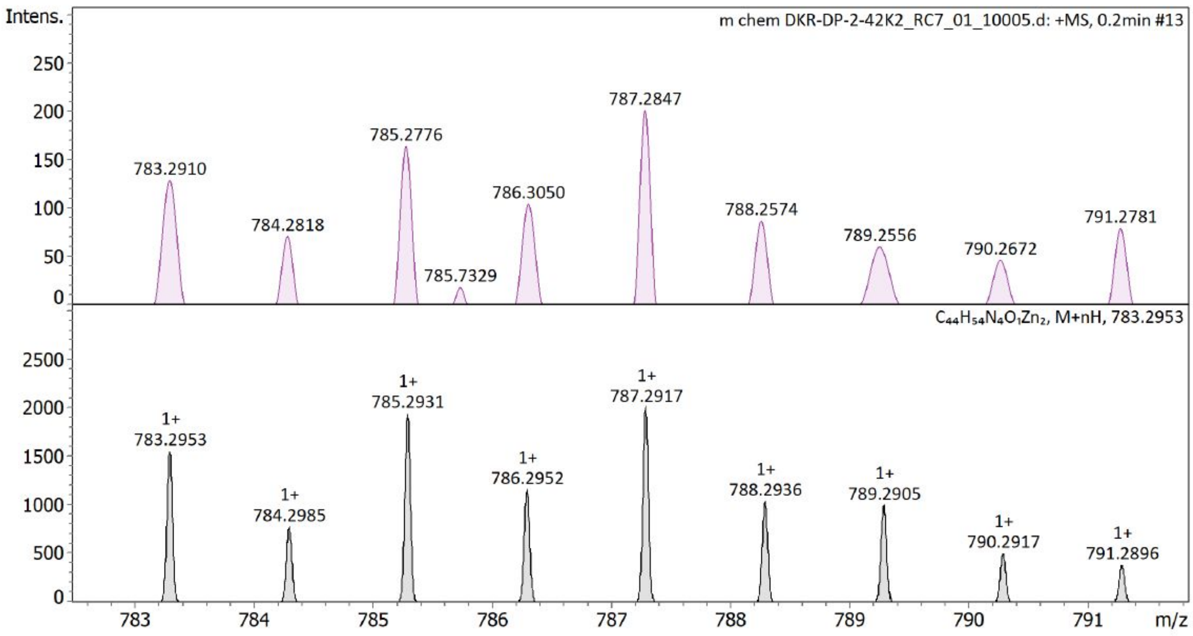

Figure S16. High-resolution mass spectrum of complex **2**. Calculated isotope pattern for $[M+H]^{+}$.

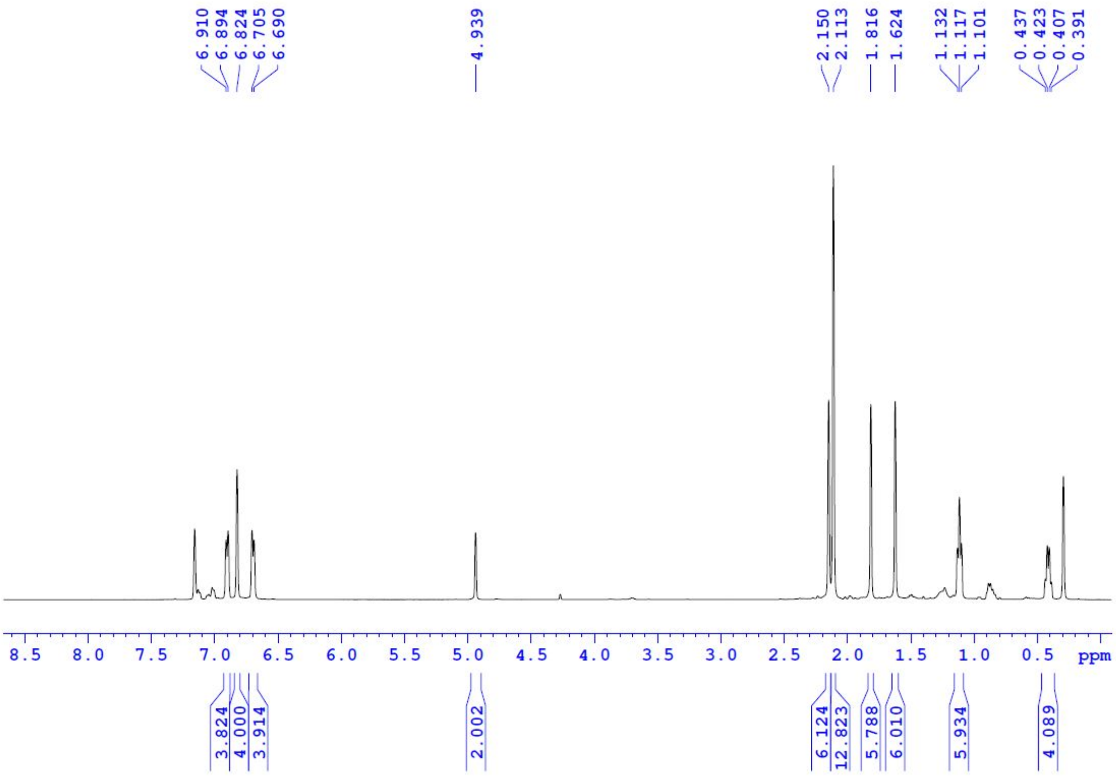

Figure S17. ^{1}H NMR spectrum **2.**

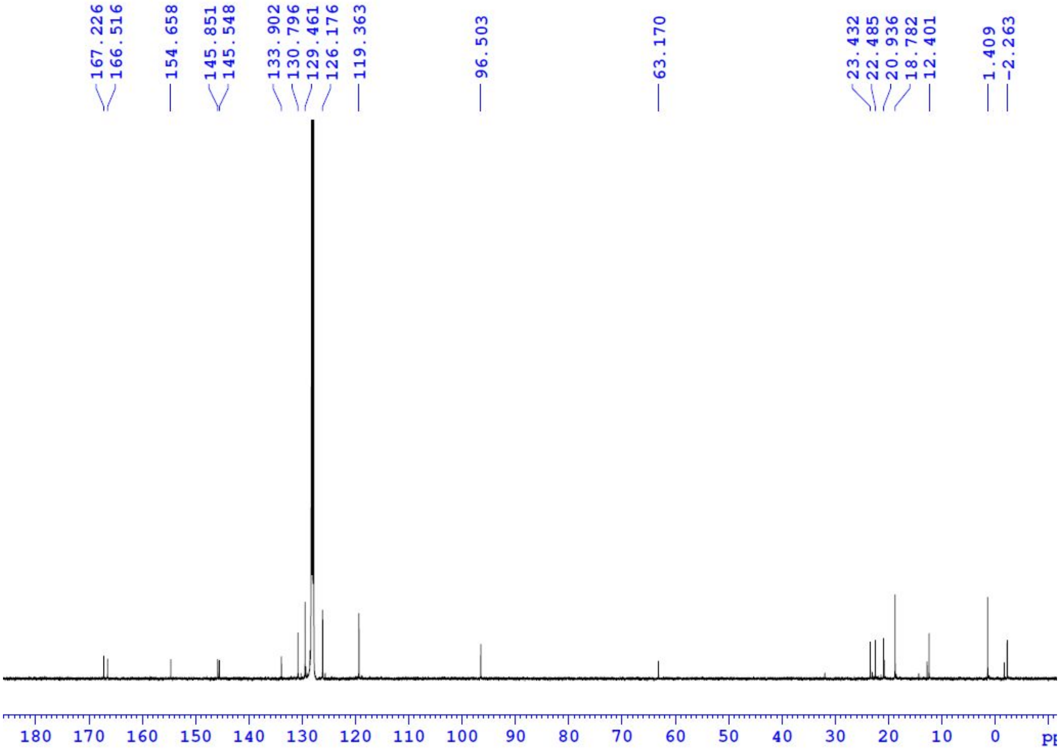

Figure S18. $^{13}C\{^{1}H\}$ NMR spectrum of **2**.

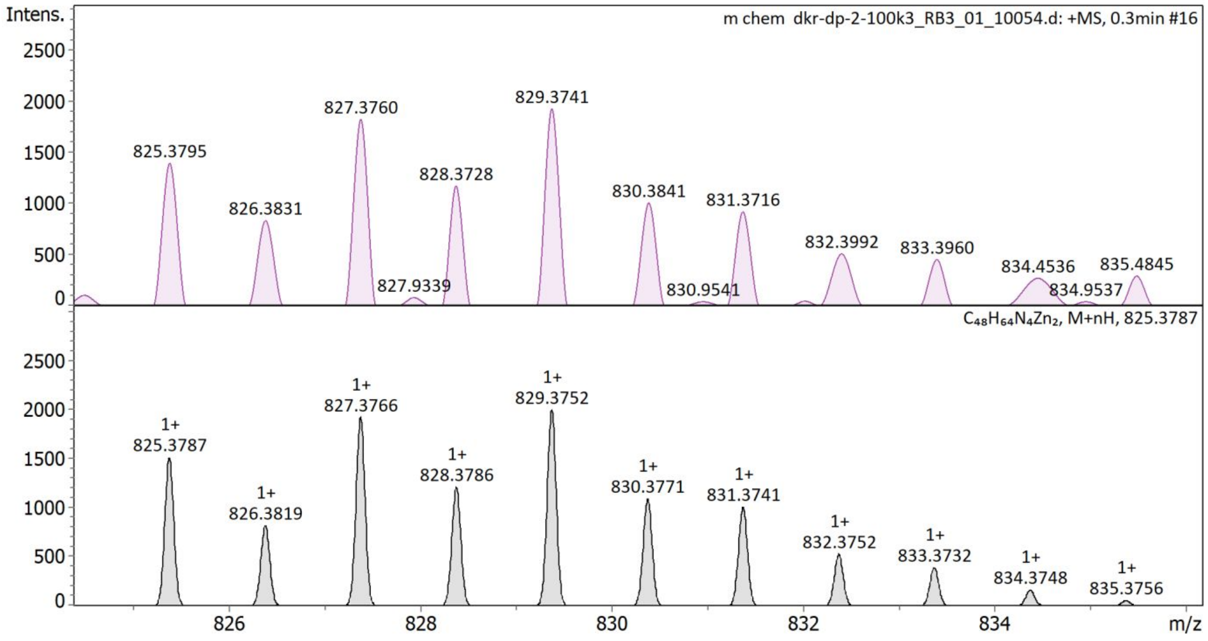

Figure S19. High-resolution mass spectrum of complex **3**. Calculated isotope pattern for $[M+H]^+$.

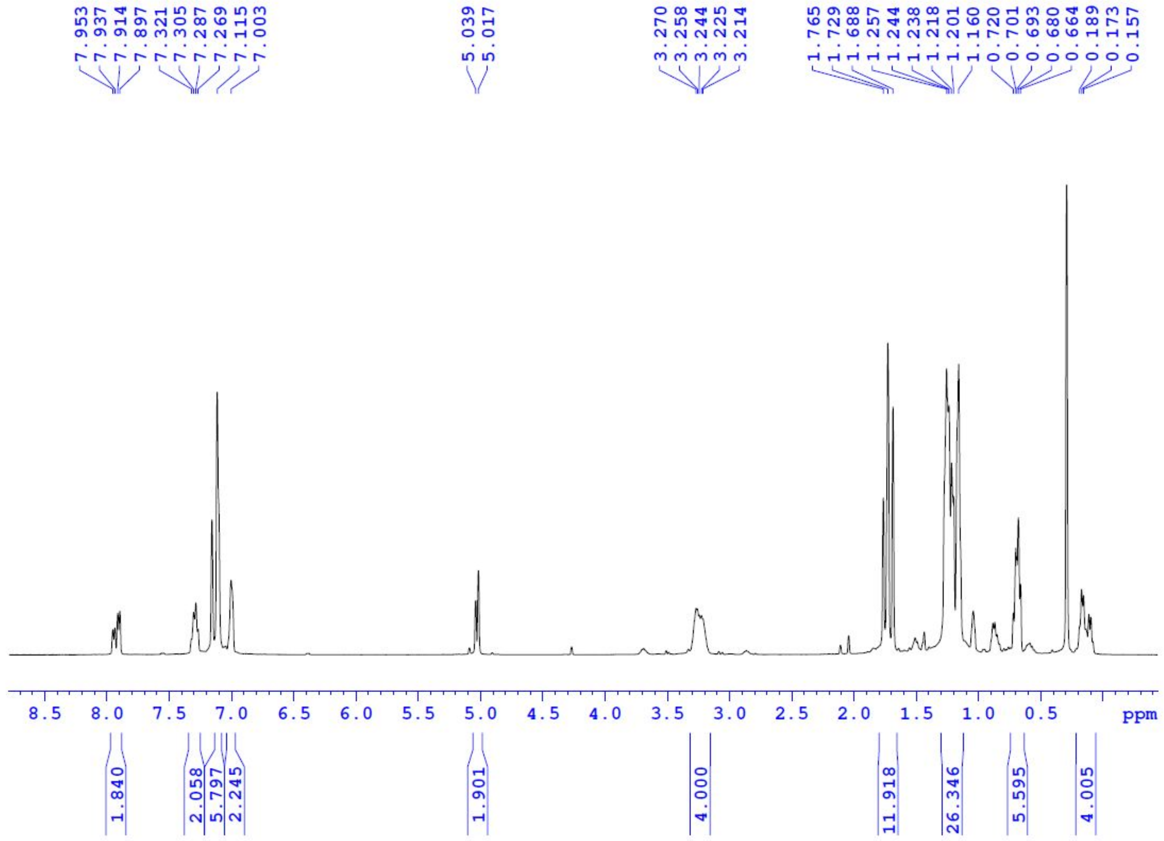

Figure S20. ^1H NMR spectrum of **3**.

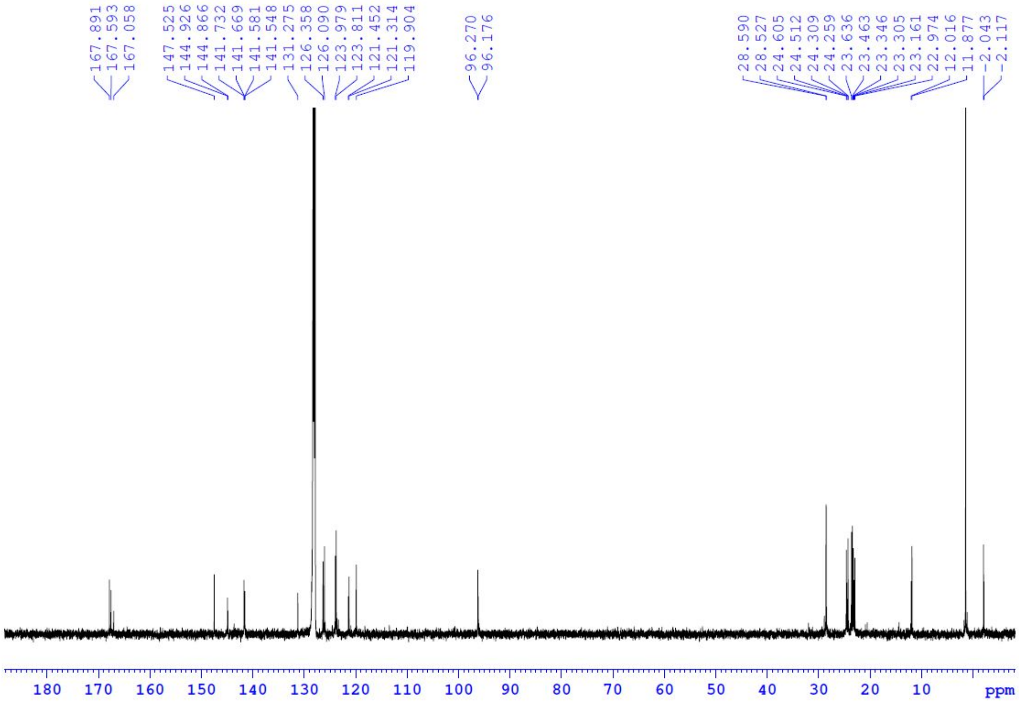

Figure S21. $^{13}C\{^{1}H\}$ NMR spectrum of **3**.

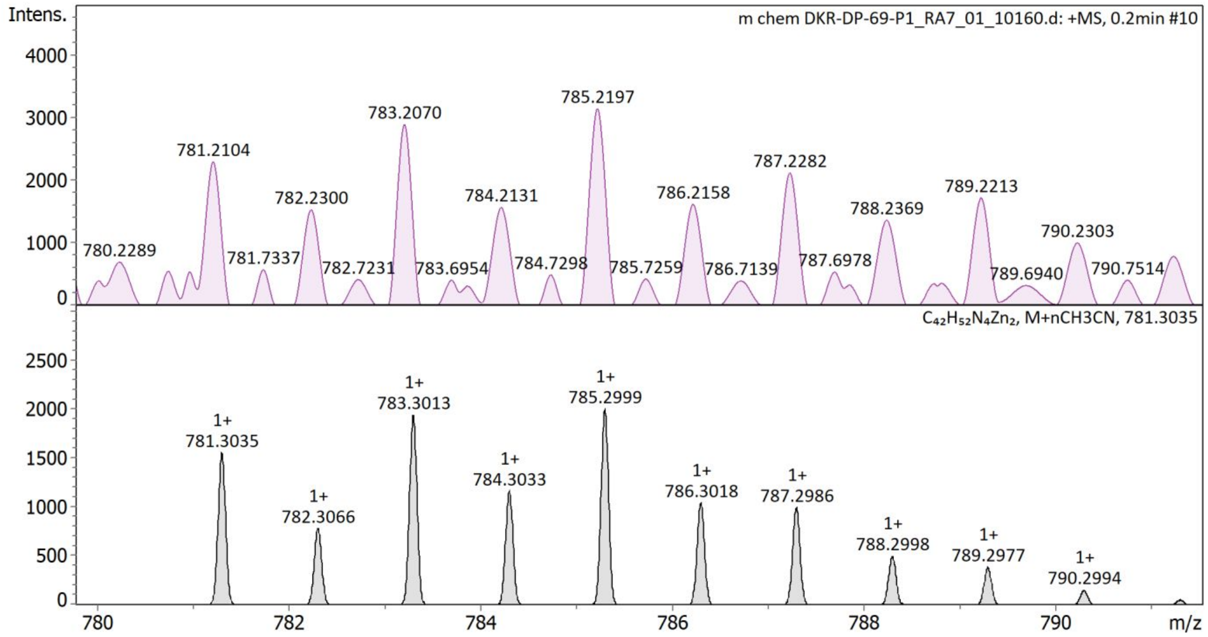

Figure S22. LC-MS spectrometry of complex **4**. Calculated isotope pattern for $[M+CH_3CN]^+$.

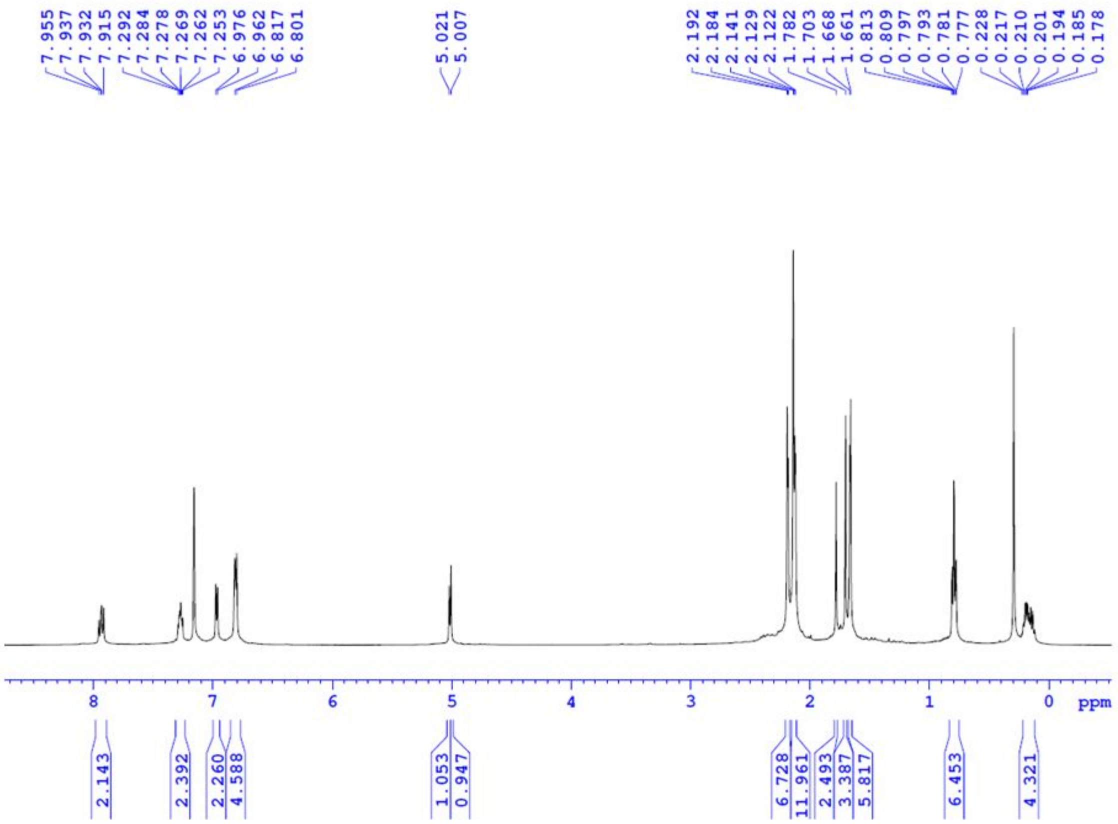

Figure S23. ^{1}H NMR spectrum of **4**.

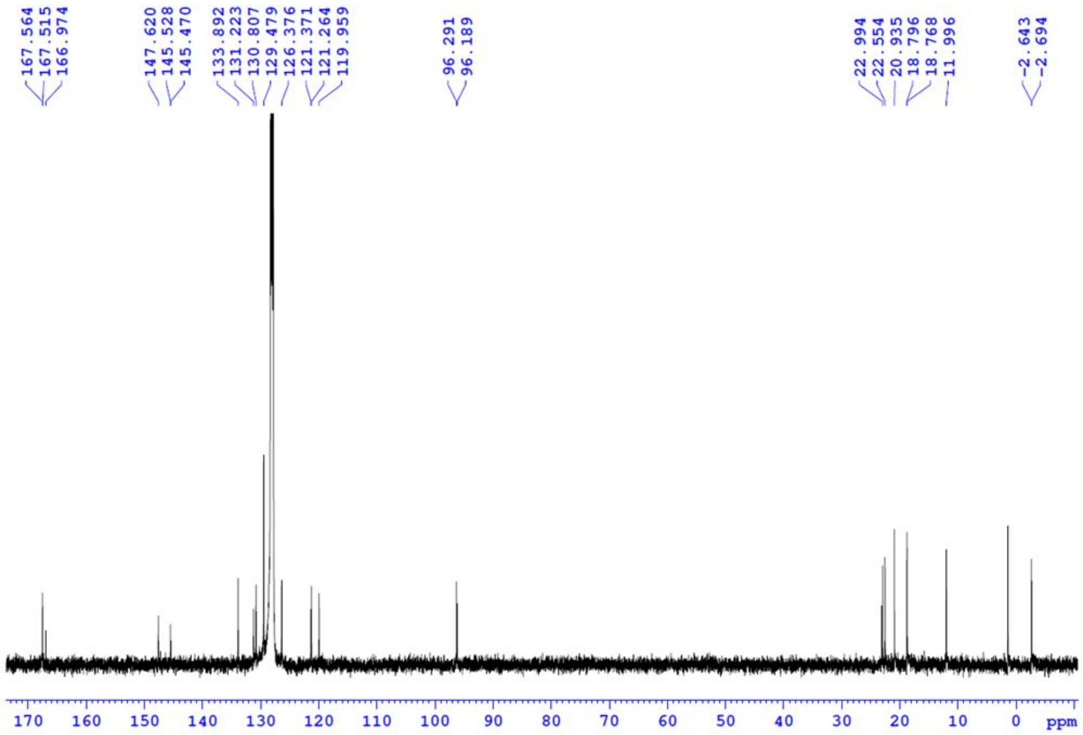

Figure S24. ^{13}C{^{1}H} NMR spectrum of **4**.

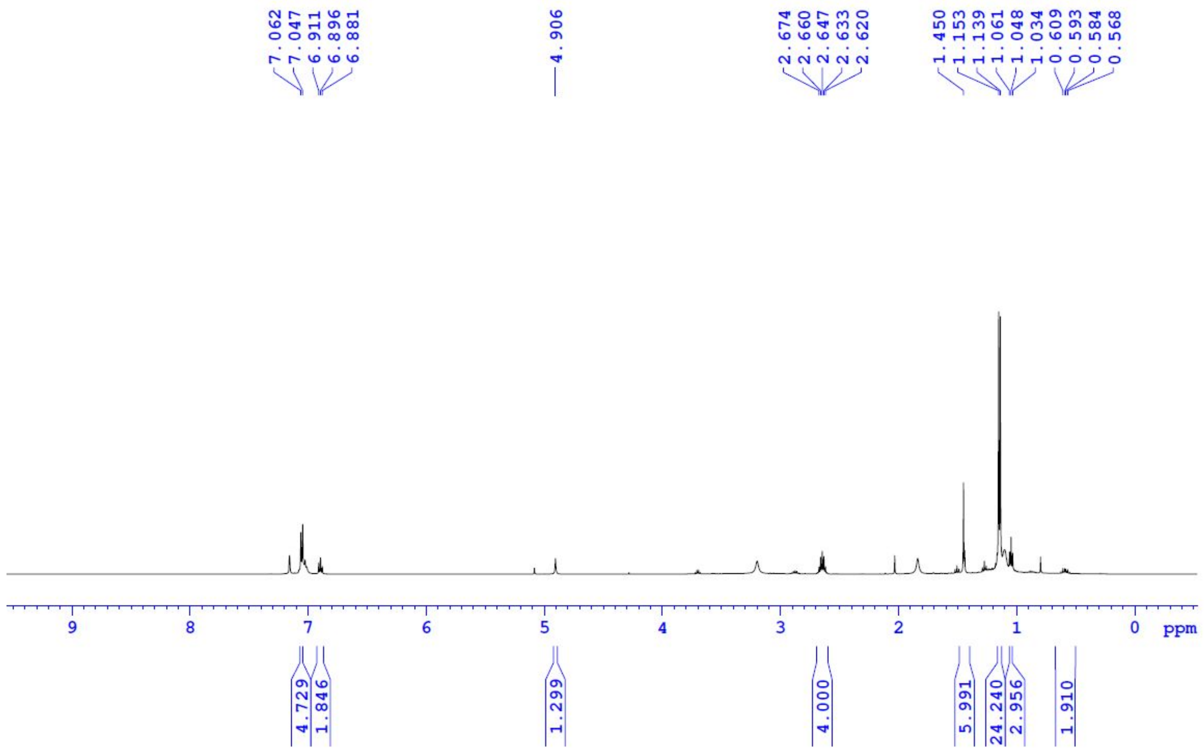

Figure S25. ^{1}H NMR spectrum of monomeric zinc complex.

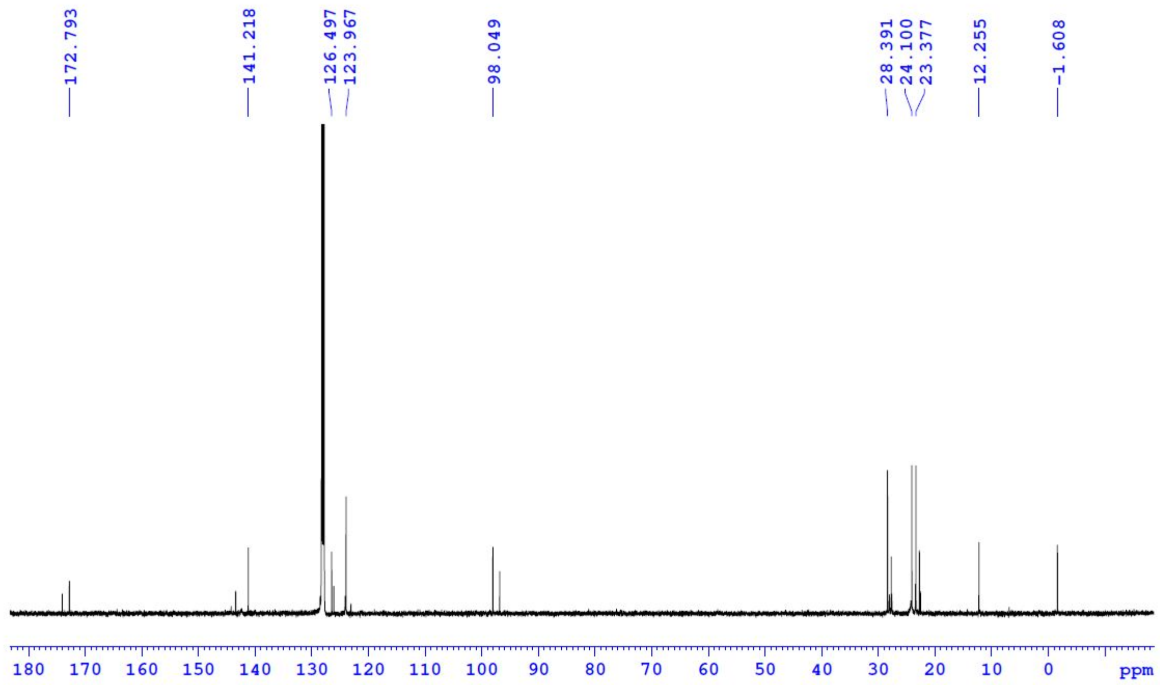

Figure S26. ^{13}C{^{1}H} NMR spectrum of monomeric zinc complex.

Catalytic Reactions

General procedure for optimization of hydroboration of nitriles using benzonitrile as substrate.

The optimization of hydroboration reactions of benzonitrile was conducted in a J. Young NMR tube. Complex **1** was dissolved in 0.5 mL of C_6D_6, followed by adding 4 equiv of HBpin and 2 equiv benzonitrile.

NMR data of optimized hydroboration reaction of benzonitrile with 1,3,5-trimethoxybenzene as an internal standard (IS)

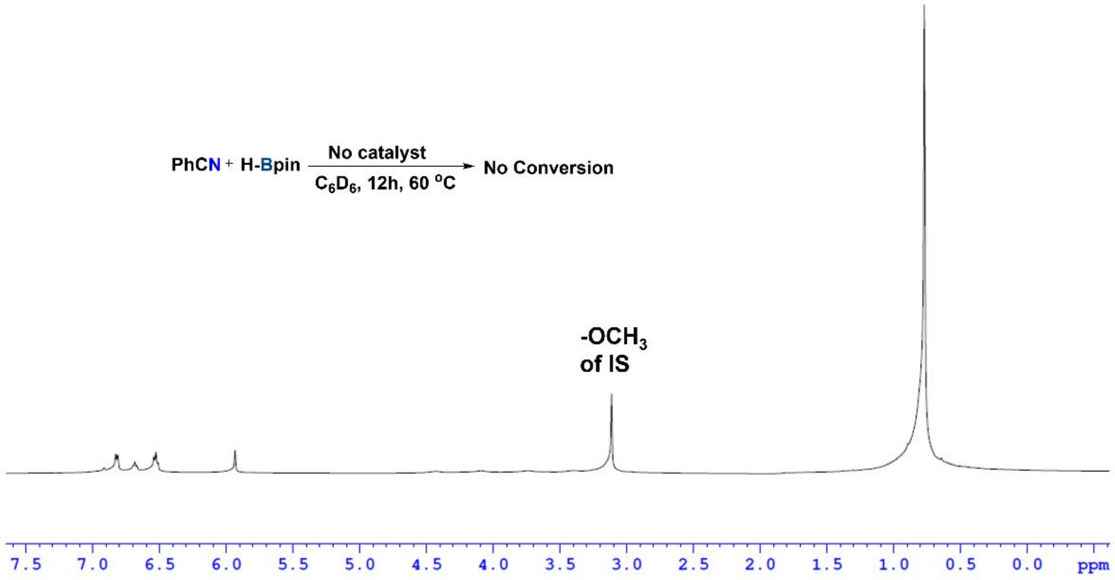

Figure S27. ^{1}H NMR of reaction mixture of HBpin, and benzonitrile with 0.05 mmol of 1,3,5-trimethoxybenzene as an internal standard.

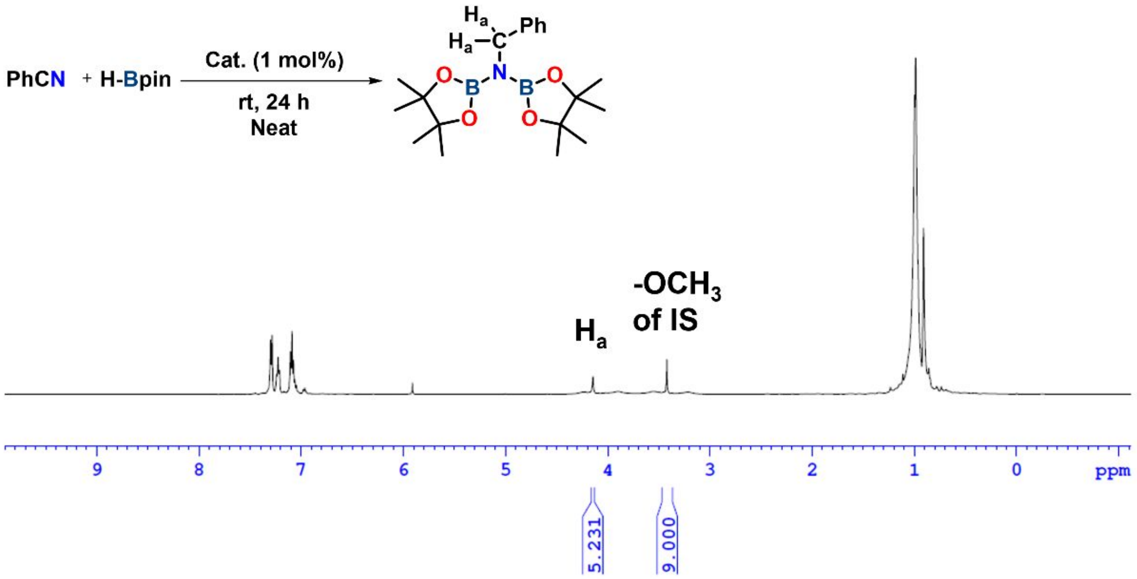

Figure S28. ^{1}H NMR of reaction mixture of **cat. (1 mol%)**, HBpin, and benzonitrile with 0.05 mmol of 1,3,5-trimethoxybenzene as an internal standard.

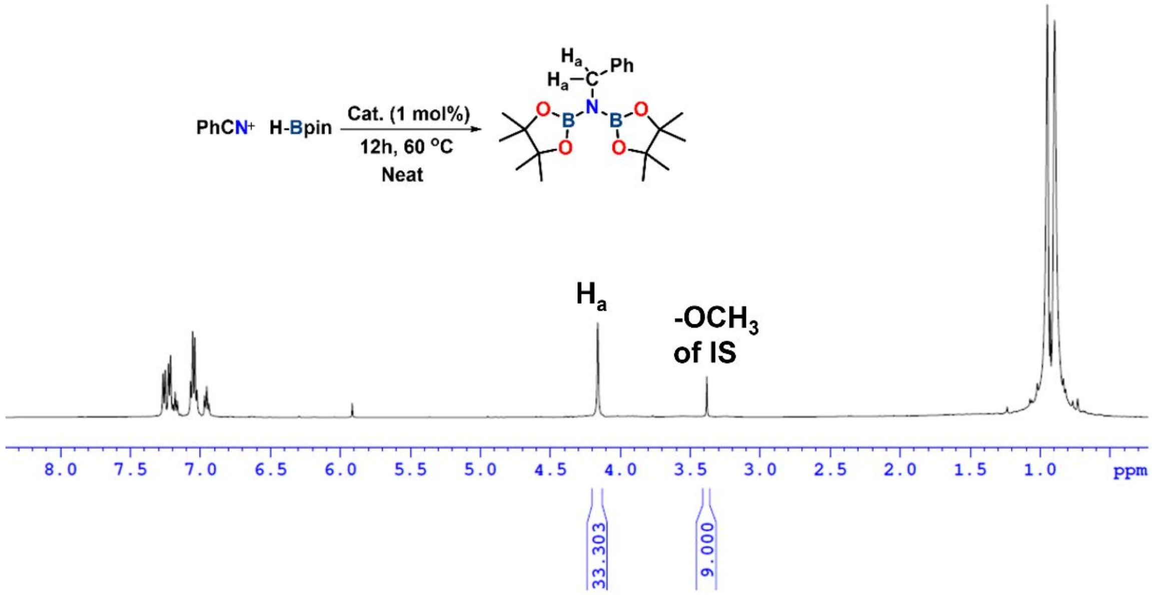

Figure S29. ^{1}H NMR of reaction mixture of **cat. (1 mol%)**, HBpin, and benzonitrile with 0.05 mmol of 1,3,5-trimethoxybenzene as an internal standard.

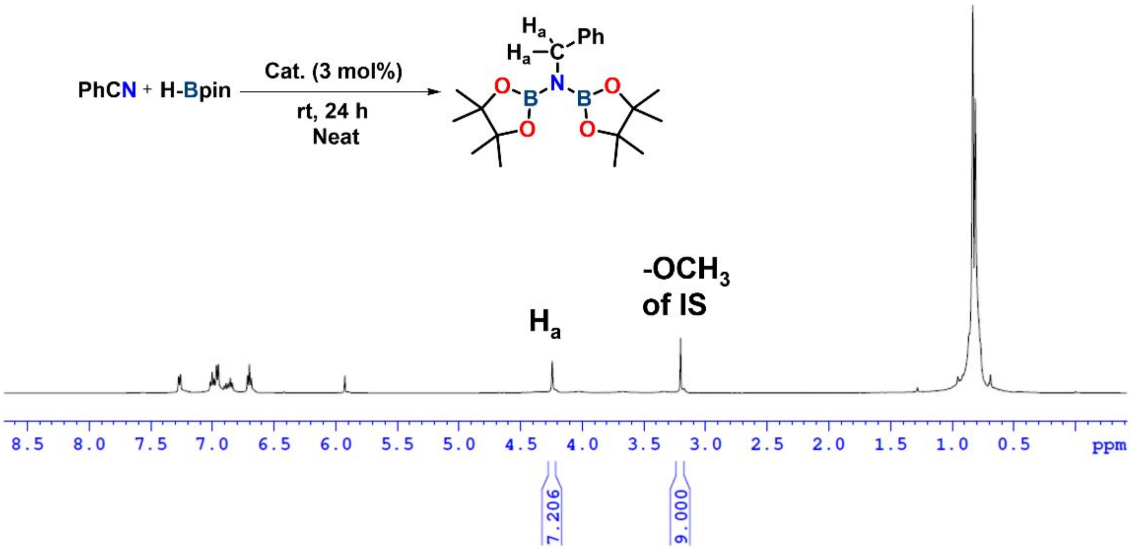

Figure S30. ^{1}H NMR of reaction mixture of **cat. (3 mol%)**, HBpin, and benzonitrile with 0.05 mmol of 1,3,5-trimethoxybenzene as an internal standard.

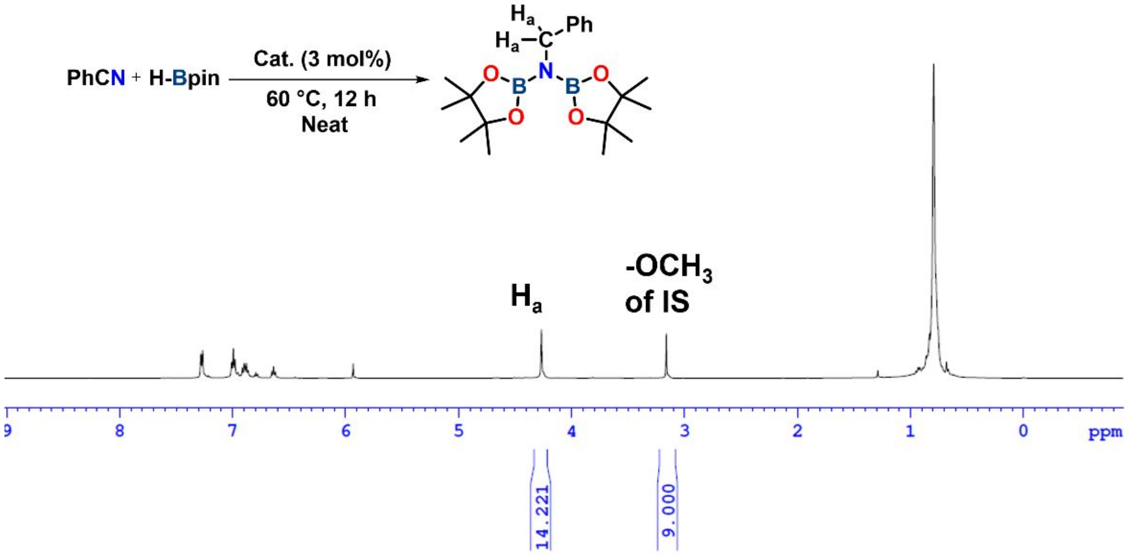

Figure S31. ^{1}H NMR of reaction mixture of **cat. (3 mol%)**, HBpin, and benzonitrile with 0.05 mmol of 1,3,5-trimethoxybenzene as an internal standard.

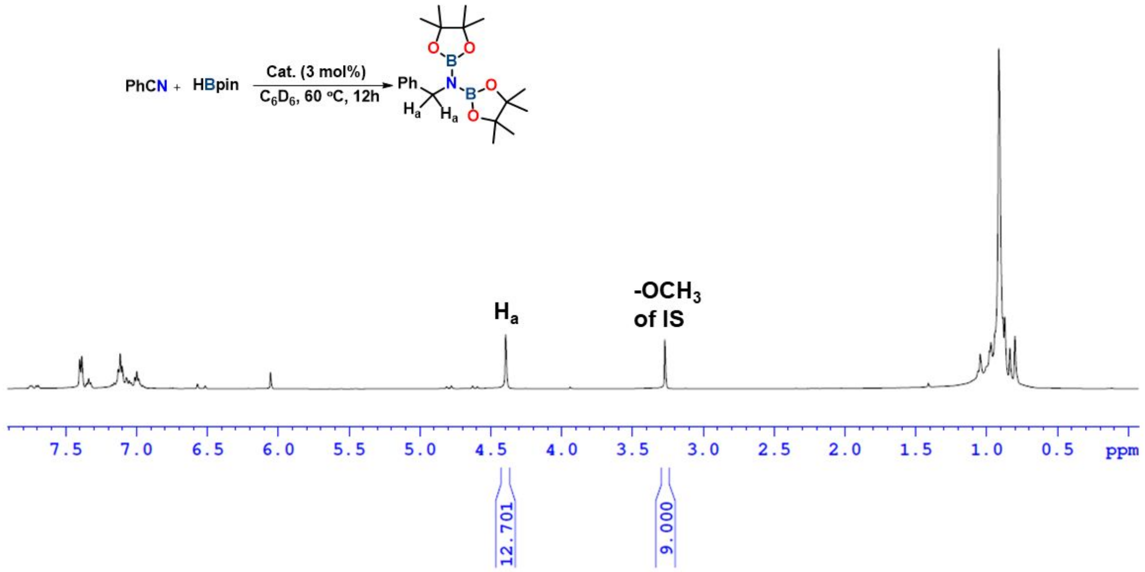

Figure S32. ^{1}H NMR of reaction mixture of **cat. (3 mol%)**, HBpin, and benzonitrile with 0.05 mmol of 1,3,5-trimethoxybenzene as an internal standard.

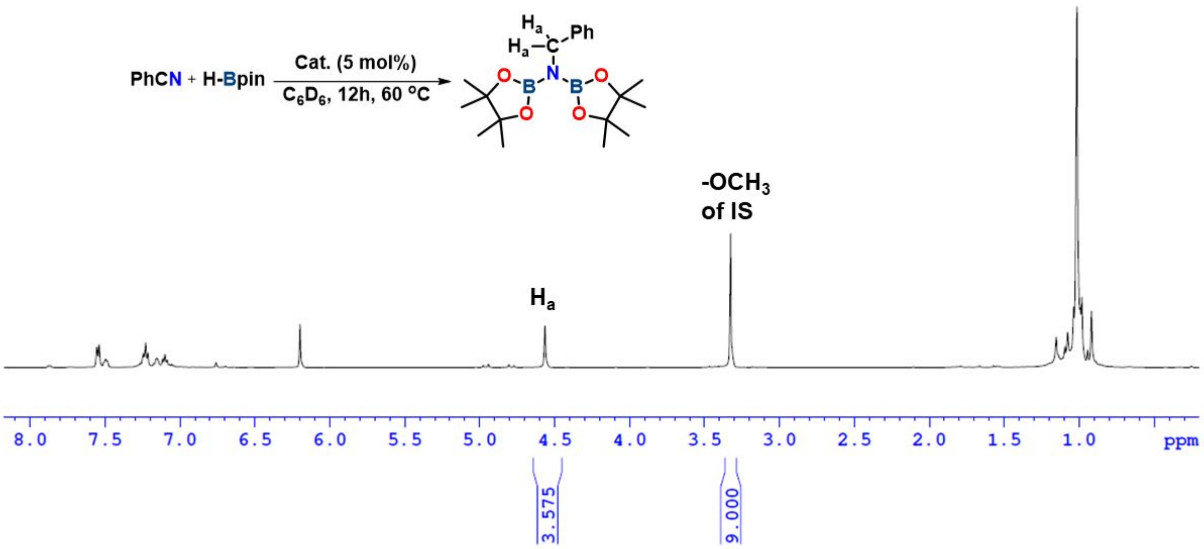

Figure S33. ^{1}H NMR of reaction mixture of **cat. (5 mol%)**, HBpin, and benzonitrile with 0.107 mmol of 1,3,5-trimethoxybenzene as an internal standard.

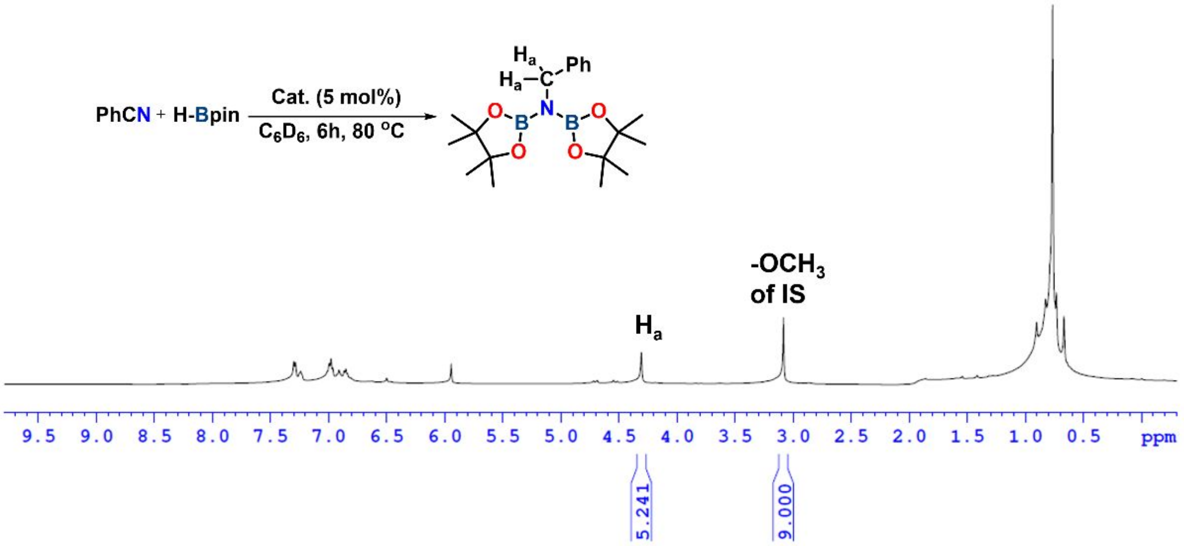

Figure S34. ^{1}H NMR of reaction mixture of **cat. (5 mol%)**, HBpin, and benzonitrile with 0.107 mmol of 1,3,5-trimethoxybenzene as an internal standard.

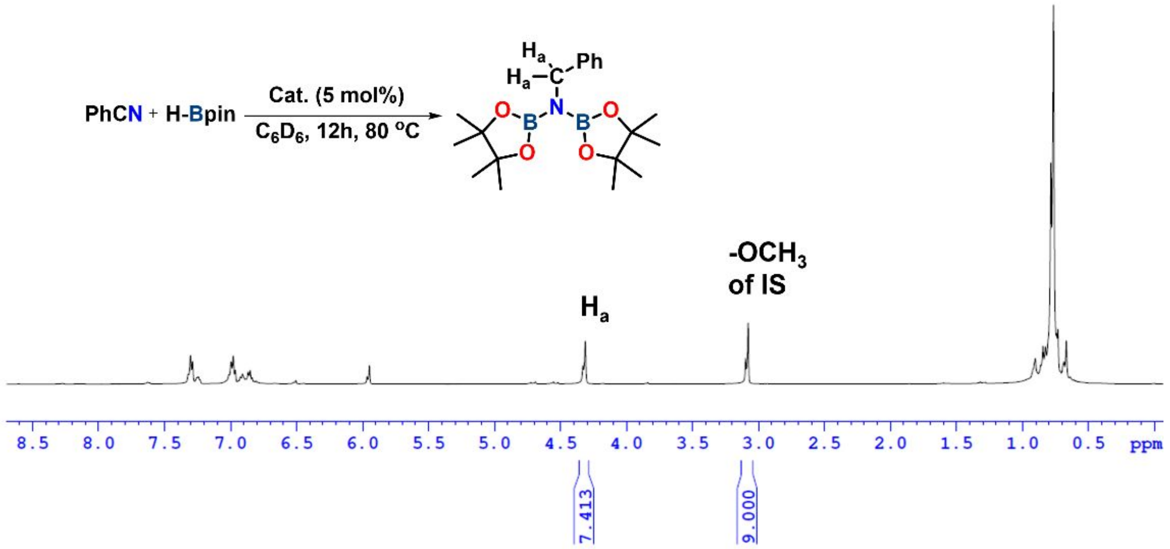

Figure S35. ^{1}H NMR of reaction mixture of **cat. (5 mol%)**, HBpin, and benzonitrile with 0.107 mmol of 1,3,5-trimethoxybenzene as an internal standard.

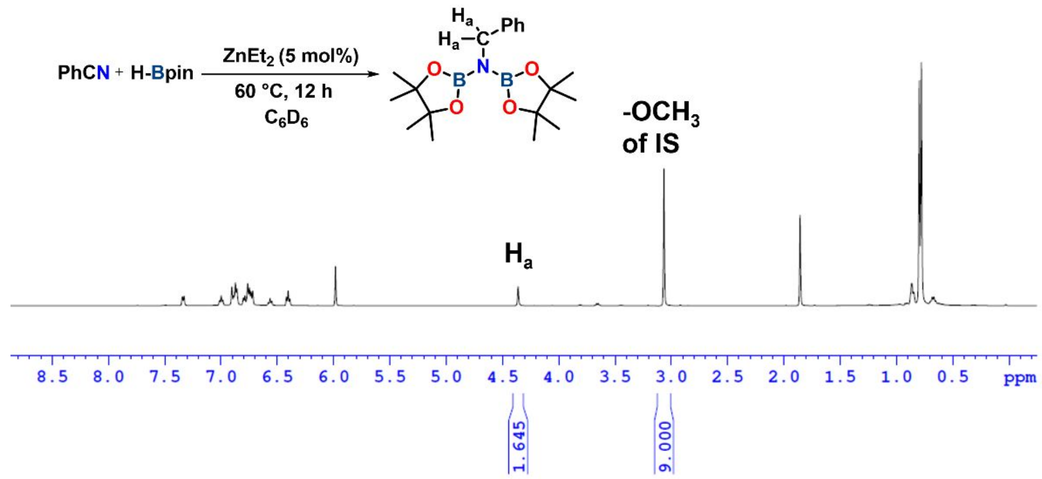

Figure S36. ^{1}H NMR of reaction mixture of **$ZnEt_2$ (5 mol%)**, HBpin, and benzonitrile with 0.05 mmol of 1,3,5-trimethoxybenzene as an internal standard.

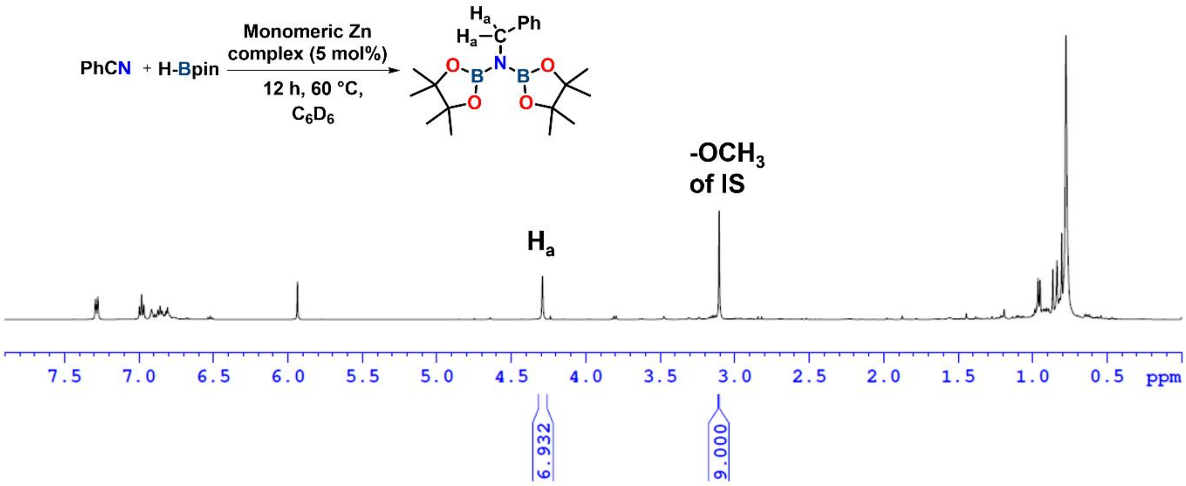

Figure S37. ^{1}H NMR of reaction mixture of monomeric Zn complex **(5 mol%)**, HBpin, and benzonitrile with 0.05 mmol of 1,3,5-trimethoxybenzene as an internal standard.

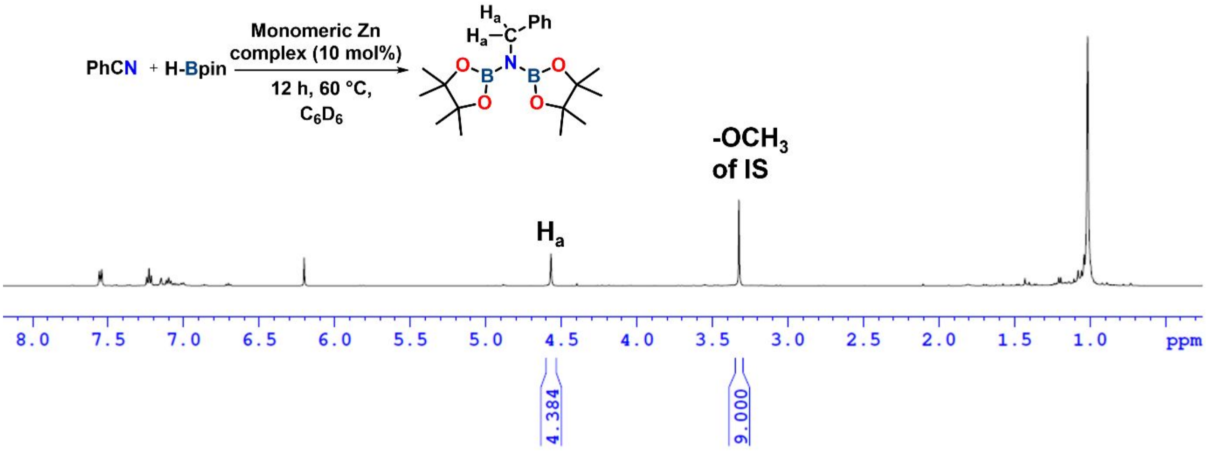

Figure S38. ^{1}H NMR of reaction mixture of monomeric Zn complex **(10 mol%)**, HBpin, and benzonitrile with 0.05 mmol of 1,3,5-trimethoxybenzene as an internal standard.

General procedure for hydroboration of nitriles

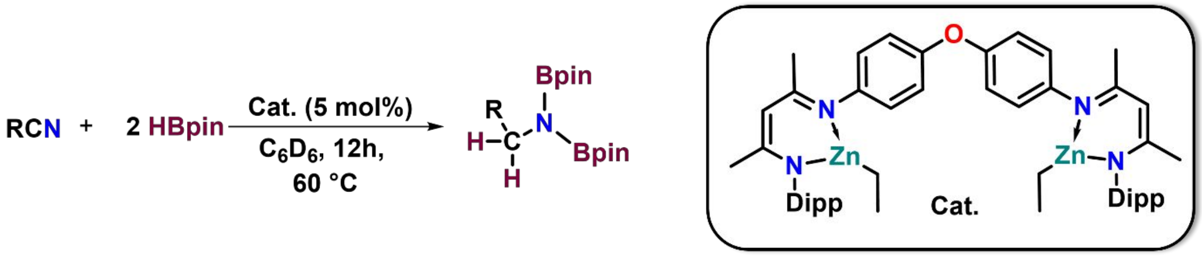

Scheme S4. General procedure for the hydroboration of nitrile.

10 mg (0.01 mmol, i.e. 5 mol%) of complex **1** was dissolved in 0.5 mL of C_6D_6, 116.08 µL (0.8 mmol) of pinacolborane was then added followed by 0.4 mmol of nitrile. This mixture was then transferred to a J Young NMR tube and the reaction was kept in an oil bath at 60 °C. The progress of the reaction was regularly monitored by ^1H and ^{11}B NMR spectroscopy until complete conversion was observed.

1a. N-{B(OCMe$_2$)$_2$} -ethan-1-amine

21 µL of acetonitrile. **^{1}H NMR** (C_6D_6, 500 MHz): 3.36 (q, J = 6.93 Hz, 2H, NCH_2), 1.22 (t, J = 6.98 Hz, 3H, CH_3), 1.02 (s, 24H, OC(CH_3)$_2$). **^{13}C{^{1}H} NMR** (C_6D_6, 126 MHz): 82.12 (OC(CH$_3$)$_2$, 39.03 (NCH$_2$), 24.73 (OC(CH$_3$)$_2$, 19.16 (CH$_2$$CH_3$). **^{11}B{^{1}H} NMR** (C_6D_6, 160 MHz): 26.25 (bs). **LC-MS (ESI):** Calculated for $C_{14}H_{29}B_2O_4N_1$: 297.2282 $[M]^+$, found: 297.2773.

1b. N-{B(OCMe$_2$)$_2$} -propan-1-amine

28 µL propionitrile. **^{1}H NMR** (C_6D_6, 500 MHz): 3.31 (t, J = 7.25 Hz, 2H, NCH_2), 1.67 (m, 2H, CH$_2$CH_2), 1.03 (s, 24H, OC(CH_3)$_2$), 0.90 (t, J = 7.43 Hz, 3H, CH$_2$CH_3). **^{13}C{^{1}H} NMR** (C_6D_6, 126 MHz): 82.09 (OC(CH$_3$)$_2$, 45.95 (NCH$_2$), 26.69 (CH$_2$$CH_2$), 24.69 (OC($CH_3$)$_2$, 11.47 (CH$_2$$CH_3$). **^{11}B{^{1}H} NMR** (C_6D_6, 160 MHz): 26.26 (bs). **LC-MS (ESI):** Calculated for $C_{15}H_{31}B_2O_4N_1$: 310.2361 $[M-H]^+$, found: 310.2496.

1c. N-{B(OCMe$_2$)$_2$} - 2,2-dimethylpropan-1-amine

45 µL Pivalonitrile. **^{1}H NMR** (C_6D_6, 500 MHz): 2.78 (s, 2H, NC*H*$_2$), 1.313 (s, 9H, C(C*H*$_3$)$_3$), 1.10 (s, 12H, OC(C*H*$_3$)$_2$), 1.07 (s, 12H, OC(C*H*$_3$)$_2$). **^{13}C{^{1}H} NMR** (C_6D_6, 126 MHz): 82.99 (O*C*(CH$_3$)$_2$, 52.66 (N*C*H$_2$), 30.45 (CH$_2$*C*H$_2$), 24.96 (OC(*C*H$_3$)$_2$. **^{11}B{^{1}H} NMR** (C_6D_6, 160 MHz): 24.17 (bs). **LC-MS (ESI):** Calculated for $C_{17}H_{35}B_2O_4N_1$: 339.2753 $[M]^+$, found: 339.1499.

1d. N-{B(OCMe$_2$)$_2$} – phenylmethanamine

41 µL benzonitrile. **^{1}H NMR** (C_6D_6, 500 MHz): 7.28 (d, J = 7.56 Hz, 2H, C*H*$_{Aryl}$), 6.99 (t, J = 7.51 Hz, 2H, C*H*$_{Aryl}$), 6.87 (t, J = 13.59 Hz, 1H, C*H*$_{Aryl}$), 4.28 (s, 2H, NC*H*$_2$), 0.78 (s, 24H, OC(C*H*$_3$)$_2$). **^{13}C{^{1}H} NMR** (C_6D_6, 126 MHz): 143.65 (*C*Aryl), 128.30 (*C*Aryl), 127.97 (*C*Aryl), 126.63 (*C*Aryl), 82.48 (O*C*(CH$_3$)$_2$, 47.81 (N*C*H$_2$), 24.71 (OC(*C*H$_3$)$_2$. **^{11}B{^{1}H} NMR** (C_6D_6, 160 MHz): 26.59 (bs). **LC-MS (ESI):** Calculated for $C_{19}H_{31}B_2O_4N_1$: 358.2362 $[M-H]^+$, found: 358.2486.

1e. N-{B(OCMe$_2$)$_2$} – 4-flouro-phenylmethanamine

48 mg 4-flourobenzonitrile. **^{1}H NMR** (C_6D_6, 500 MHz): 7.37 (t, J = 7.28 Hz, 2H, C*H*$_{Aryl}$), 6.86 (t, J = 8.62 Hz, 2H, C*H*$_{Aryl}$), 4.39 (s, 2H, NC*H*$_2$), 1.01 (s, 24H, OC(C*H*$_3$)$_2$). **^{13}C{^{1}H} NMR** (C_6D_6, 126 MHz): 161.18 (*C*Aryl), 139.52 (*C*Aryl), 129.72 (*C*Aryl), 115.04 (*C*Aryl), 82.57 (O*C*(CH$_3$)$_2$, 47.06 (N*C*H$_2$), 24.39 (OC(*C*H$_3$)$_2$. **^{11}B{^{1}H} NMR** (C_6D_6, 160 MHz): 26.49 (bs). **LC-MS (ESI):** Calculated for $C_{19}H_{30}B_2O_4N_1F_1$: 416.1984 $[M+K]^+$, found: 416.2054.

1f. N-{B(OCMe$_2$)$_2$} – 2-chloro-phenylmethanamine

55 mg 2-chlorobenzonitrile. **^{1}H NMR** (C_6D_6, 500 MHz): 7.49 (d, J = 7.72 Hz, 1H, C*H*$_{Aryl}$), 7.12 (d, J = 7.87 Hz, 1H, C*H*$_{Aryl}$), 7.05 (t, J = 7.55 Hz, 1H, C*H*$_{Aryl}$), 6.82 (t, J = 15.25 Hz, 1H, C*H*$_{Aryl}$), 4.74 (s, 2H, NC*H*$_2$), 0.99 (s, 24H, OC(C*H*$_3$)$_2$). **^{13}C{^{1}H} NMR** (C_6D_6, 126 MHz): 140.60 (*C*Aryl), 133.10 (*C*Aryl), 129.42 (*C*Aryl), 127.44 (*C*Aryl), 126.96(*C*Aryl), 126.63(*C*Aryl), 82.66 (O*C*(CH$_3$)$_2$, 45.77 (N*C*H$_2$), 24.60 (OC(*C*H$_3$)$_2$. **^{11}B{^{1}H} NMR** (C_6D_6, 160

MHz): 26.44 (bs). **LC-MS (ESI):** Calculated for $C_{19}H_{30}B_2O_4N_1Cl_1$: 416.1949 $[M+Na]^+$, found: 416.2001.

1g. N-{B(OCMe$_2$)$_2$} – 4-bromo-phenylmethanamine

73 mg 4-bromobenzonitrile. **^{1}H NMR** (C_6D_6, 500 MHz): 7.30 (d, J = 8.49 Hz, 2H, CH_{Aryl}), 7.23 (t, J = 8.34 Hz, 2H, CH_{Aryl}), 4.34 (s, 2H, NCH_2), 0.99 (s, 24H, OC(CH_3)$_2$). **^{13}C{^{1}H} NMR** (C_6D_6, 126 MHz): 142.69 (*C*Aryl), 131.42 (*C*Aryl), 129.89 (*C*Aryl), 120.52 (*C*Aryl), 82.64 (O*C*(CH$_3$)$_2$, 47.17 (N*C*H$_2$), 24.72 (OC(*C*H$_3$)$_2$. **^{11}B{^{1}H} NMR** (C_6D_6, 160 MHz): 26.42 (bs). **LC-MS (ESI):** Calculated for $C_{19}H_{30}B_2O_4N_1Br_1$: 460.1446 $[M+Na]^+$, found: 460.1415.

1h. N-{B(OCMe$_2$)$_2$} – 4-iodo-phenylmethanamine

92 mg 4-iodobenzonitrile. **^{1}H NMR** (C_6D_6, 500 MHz): 7.49 (d, J = 7.83 Hz, 2H, CH_{Aryl}), 7.11 (d, J = 7.91 Hz, 2H, CH_{Aryl}), 4.34 (s, 2H, NCH_2), 0.99 (s, 24H, OC(CH_3)$_2$). **^{13}C{^{1}H} NMR** (C_6D_6, 126 MHz): 143.29 (*C*Aryl), 137.39 (*C*Aryl), 130.13 (*C*Aryl), 91.96 (*C*Aryl), 82.60 (O*C*(CH$_3$)$_2$, 47.22 (N*C*H$_2$), 24.70 (OC(*C*H$_3$)$_2$. **^{11}B{^{1}H} NMR** (C_6D_6, 160 MHz): 26.49 (bs). **LC-MS (ESI):** Calculated for $C_{19}H_{30}B_2O_4N_1I_1$: 508.1305 $[M+Na]^+$, found: 508.1276.

1i. N-{B(OCMe$_2$)$_2$} – 4-nitro-phenylmethanamine

59 mg 4-nitro-benzonitrile. **^{1}H NMR** (C_6D_6, 500 MHz): 7.91 (d, J = 8.5 Hz, 2H, CH_{Aryl}), 7.26 (d, J = 8.5 Hz, 2H, CH_{Aryl}), 4.34 (s, 2H, NCH_2), 1.0 (s, 24H, OC(CH_3)$_2$). **^{13}C{^{1}H} NMR** (C_6D_6, 126 MHz): 150.68 (*C*Aryl), 147.11 (*C*Aryl), 123.43 (*C*Aryl), 82.83 (O*C*(CH$_3$)$_2$, 47.28 (N*C*H$_2$), 24.64 (OC(*C*H$_3$)$_2$. **^{11}B{^{1}H} NMR** (C_6D_6, 160 MHz): 26.44 (bs). **LC-MS (ESI):** Calculated for $C_{19}H_{30}B_2O_6N_2$: 403.2206 $[M-H]^+$, found: 403.2701.

1j. N-{B(OCMe$_2$)$_2$} – 4-cyanobenzylbromidamine

78 mg 4-cyanobenzylbromide. **^{1}H NMR** (C_6D_6, 500 MHz): 7.36 (d, J = 7.70 Hz, 2H, CH_{Aryl}), 7.08 (d, J = 7.65 Hz, 2H, CH_{Aryl}), 4.44 (s, 2H, NCH_2), 4.07 (s, 2H, CH_2Br), 1.0 (s, 24H, OC(CH_3)$_2$). **^{13}C{^{1}H} NMR** (C_6D_6, 126 MHz): 143.93 (*C*Aryl), 136.13 (*C*Aryl), 129.11

(*C*Aryl), 82.59 (O*C*(CH$_3$)$_2$, 47.49 (N*C*H$_2$), 33.70 (*C*H$_2$Br), 24.71 (OC(*C*H$_3$)$_2$. **^{11}B{^{1}H} NMR** (C$_6$D$_6$, 160 MHz): 26.8 (bs). **LC-MS (ESI):** Calculated for $C_{20}H_{32}B_2O_4N_1Br_1$: 474.1603 [M+Na]$^+$, found: 474.1629.

1k. N-{B(OCMe$_2$)$_2$} – 4-cyanopyridinamine

42 mg 4-cyanopyridine. **^{1}H NMR** (C$_6$D$_6$, 500 MHz): 8.49 (d, 2H, C*H*$_{Aryl}$), 7.08 (d, 2H, C*H*$_{Aryl}$), 4.29 (s, 2H, NC*H*$_2$), .092 (s, 24H, OC(C*H*$_3$)$_2$). **^{13}C{^{1}H} NMR** (C$_6$D$_6$, 126 MHz): 151.77 (*C*Aryl), 150.04 (*C*Aryl), 122.38 (*C*Aryl), 82.76 (O*C*(CH$_3$)$_2$, 46.91 (N*C*H$_2$), 24.62 (OC(*C*H$_3$)$_2$. **^{11}B{^{1}H} NMR** (C$_6$D$_6$, 160 MHz): 26.11 (bs). **LC-MS (ESI):** Calculated for $C_{18}H_{30}B_2O_4N_2$: 361.2471 [M-H]$^+$, found: 361.2478.

1l.

52 mg 4-Cyanobenzaldehyde. **^{1}H NMR** (C$_6$D$_6$, 500 MHz): 7.25 (d, J = 8 Hz, 2H, C*H*$_{Aryl}$), 7.07 (d, J = 8 Hz, 2H, C*H*$_{Aryl}$), 4.69 (s, 2H, NC*H*$_2$), 4.25 (s, 2H, OCH$_2$), 0.83 (s, 12H, OC(C*H*$_3$)$_2$), 0.81 (s, 36H, OC(C*H*$_3$)$_2$). **^{11}B{^{1}H} NMR** (C$_6$D$_6$, 160 MHz): 22.65 (bs), 21.75 (bs).

1l′.

52 mg 4-Cyanobenzaldehyde. **^{1}H NMR** (C$_6$D$_6$, 500 MHz): 6.78 (d, J = 8.7 Hz, 2H, C*H*$_{Aryl}$), 6.66 (d, J = 8.20 Hz 2H, C*H*$_{Aryl}$), 4.42 (s, 2H, OC*H*$_2$), 0.79 (s, 12H, OC(C*H*$_3$)$_2$). **^{11}B{^{1}H} NMR** (C$_6$D$_6$, 160 MHz): 22.67 (bs).

1m. 2-(benzhydryloxy)-4,4,5,5-tetramethyl-1,3,2-dioxaborolane

73mg benzophenone and 27.8 µL propionitrile. **^{1}H NMR** (C$_6$D$_6$, 500 MHz): 7.35 (d, J = 7.4 Hz, 4H, C*H*$_{Aryl}$), 7.08 (t, J = 7.64Hz, 4H, C*H*$_{Aryl}$), 7.01 (d, J = 7.5 Hz, 2H, C*H*$_{Aryl}$), 6.31 (s, 1H, OC*H*), 1.11 (s, 12H, OC(C*H*$_3$)$_2$). **^{13}C{^{1}H} NMR** (C$_6$D$_6$, 126 MHz): 142.33 (*C*Aryl), 129.73 (*C*Aryl), 126.97 (*C*Aryl), 118.16 (*C*N), 84.11 (O*C*(CH$_3$)$_2$, 65.28 (O*C*HCN), 24.64 (OC(*C*H$_3$)$_2$. **^{11}B{^{1}H} NMR** (C$_6$D$_6$, 160 MHz): 22.87 (bs). **LC-MS (ESI):** Calculated for $C_{19}H_{23}B_1O_3$:

310.1738 [M]$^+$, found: 310.2486 (for benzophenone); Calculated for $C_{15}H_{31}B_2O_4N_1$: 310.2361 [M-H]$^+$, found: 310.2490 (for propionitrile).

General procedure for hydroboration of carbodiimides

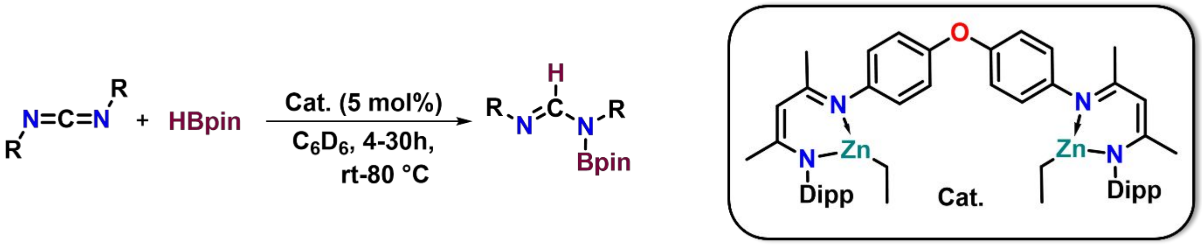

Scheme S5. General procedure for hydroboration of carbodiimide.

6 mg (0.01 mmol, i.e. 5 mol%) of complex **1** was dissolved in 0.5 mL of C_6D_6, 81 μL (0.8 mmol) of pinacolborane was then added followed by 0.14 mmol of carbodiimide. This mixture was transferred to a J Young NMR tube and kept at room temperature for 4 h, except for dipp-carbodiimides which was heated at 60 °C for 30 h. The progress of the reaction was regularly monitored by ^1H and ^{11}B NMR spectroscopy until complete conversion of the starting material was observed.

2a. N,N′-diisopropyl-N-{B(OCMe$_2$)$_2$} – formimidamide

44 μL N,N′-diisopropylcarbodiimide. **^{1}H NMR** (C_6D_6, 500 MHz): 8.14 (s, 1H, NC*H*), 4.87 (m, 1H, C*H*), 3.26 (m, 1H, C*H*), 1.37 (d, J = 7 Hz, 6H, C*H*$_3$), 1.14 (d, J = 6.5 Hz, 6H, C*H*$_3$), 1.02 (s, 12H, OC(C*H*$_3$)$_2$). **^{13}C{^{1}H} NMR** (C_6D_6, 126 MHz): 149.95 (N*C*N), 82.75 (O*C*(CH$_3$)$_2$, 57.20 (CH$_3$*C*H), 43.59 (CH$_3$*C*H), 25.74 (*C*H$_3$CH), 24.58 (OC(*C*H$_3$)$_2$, 21.82 (*C*H$_3$CH). **^{11}B{^{1}H} NMR** (C_6D_6, 160 MHz): 25.28 (bs). **LCMS (ESI):** Calculated for $C_7H_{16}N_2$: 129.1386 [M+H-Bpin]$^+$, found: 129.1371.

2b. N,N′-di-tert-butyl-N-{B(OCMe$_2$)$_2$} – formimidamide

43 μL N,N′-di-tert-butylcarbodiimide. **^{1}H NMR** (C_6D_6, 500 MHz): 8.23 (s, 1H, NC*H*), 1.47 (s, 18H, C*H*$_3$), 1.01 (s, 12H, OC(C*H*$_3$)$_2$). **^{13}C{^{1}H} NMR** (C_6D_6, 126 MHz): 149.24 (N*C*N), 82.20 (O*C*(CH$_3$)$_2$, 55.10 ((CH$_3$)$_3$*C*), 30.63 (*C*H$_3$C), 24.99 (OC(*C*H$_3$)$_2$, 24.54 (*C*H$_3$C). **^{11}B{^{1}H} NMR**

(C_6D_6, 160 MHz): 25.56 (bs). **LCMS (ESI):** Calculated for $C_9H_{20}N_2$: 157.1699 [M+H-Bpin]$^+$, found: 157.1671.

2c. N,N′-dicyclohexyl-N-{B(OCMe$_2$)$_2$} – formimidamide

58 mg N,N′-di-tert-butylcarbodiimide. **^{1}H NMR** (C_6D_6, 500 MHz): 8.20 (s, 1H, NC*H*), 4.51-4.46 (m, 1H, C*H*$_{Cy}$), 2.93 (m, 1H, C*H*$_{Cy}$), 2.08-1.10 (m, 20H, C*H*$_{2Cy}$), 1.04 (s, 12H, OC(C*H*$_3$)$_2$). **^{13}C{^{1}H} NMR** (C_6D_6, 126 MHz): 150.51 (N*C*N), 82.73 (O*C*(CH$_3$)$_2$, 65.34 (*C*H$_{Cy}$), 51.71 (*C*H$_{Cy}$), 36.17 (*C*H$_{2Cy}$), 32.02 (*C*H$_{2Cy}$), 26.87 (*C*H$_{2Cy}$), 26.33 (*C*H$_{2Cy}$), 26.11 (*C*H$_{2Cy}$), 24.60 (OC(*C*H$_3$)$_2$. **^{11}B{^{1}H} NMR** (C_6D_6, 160 MHz): 25.45 (bs). **LCMS (ESI):** Calculated for $C_3H_{24}N_2$: 209.2012 [M+H-Bpin]$^+$, found: 209.1988.

2d. N,N′-bis(2,6-diisopropylphenyl)-N-{B(OCMe$_2$)$_2$} – formimidamide

51 mg 2,6-diisopropylaniline. **^{1}H NMR** (C_6D_6, 500 MHz): 8.35 (s, 1H, NC*H*), 7.27-7.15 (m, 6H, C*H*$_{Aryl}$), 3.40 (m, 2H, C*H*), 3.32 (m, 2H, C*H*), 1.43 (t, J = 14.78 Hz, 12H, CHC*H*$_3$), 1.25 (d, J = 7 Hz, 12H, CHC*H*$_3$), 1.04 (s, 12H, OC(C*H*$_3$)$_2$). **^{13}C{^{1}H} NMR** (C_6D_6, 126 MHz): 152.47 (N*C*N), 148.24 (*C*H$_{Aryl}$), 146.24 (*C*H$_{Aryl}$), 139.66 (*C*H$_{Aryl}$), 134.27 (*C*H$_{Aryl}$), 123.75 (*C*H$_{Aryl}$), 123.19 (*C*H$_{Aryl}$), 83.95 (O*C*(CH$_3$)$_2$, 29.07 (*C*H), 27.79 (*C*H), 25.34 (*C*H$_3$), 24.44 (OC(*C*H$_3$)$_2$, 23.66 (*C*H$_3$). **^{11}B{^{1}H} NMR** (C_6D_6, 160 MHz): 25.48 (bs). **LCMS (ESI):** Calculated for $C_{25}H_{36}N_2$: 365.2951 [M+H-Bpin]$^+$, found: 365.3104.

Gram Scale reaction:

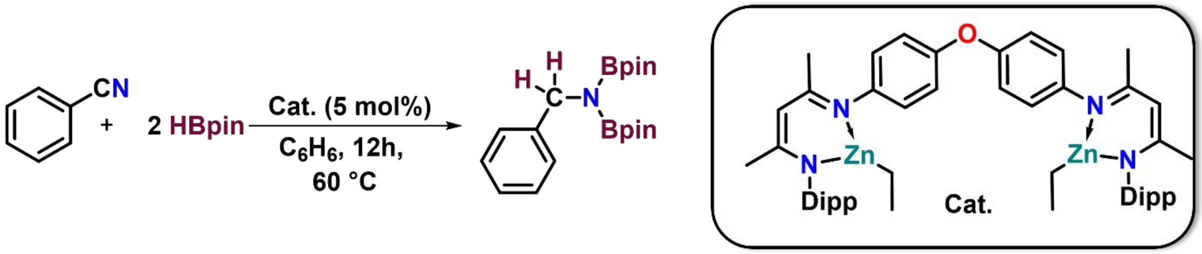

Scheme S6: Gram scale reaction of benzonitrile with HBpin catalyzed by Zn complex.

300 mg (5 mol%) of complex **1** was dissolved in benzene, 4 mL (27.6 mmol) of pinacolborane was then added followed by 1.4 mL (13.8 mmol) of benzonitrile. The resulting reaction mixture was heated at 60 °C for 12 h. After being stirred for 12 h, reaction mixture was allowed to cool to rt and solvent was removed in vaccum. The resulting solid washed with hexane to afford yellow solid as a product.

Yield : 80%

^{1}H NMR (C_6D_6, 500 MHz): 7.28 (d, J = 7.56 Hz, 2H, CH_{Aryl}), 6.99 (t, J = 7.51 Hz, 2H, CH_{Aryl}), 6.87 (t, J = 13.59 Hz, 1H, CH_{Aryl}), 4.28 (s, 2H, NCH_2), 0.78 (s, 24H, OC(CH_3)$_2$). **^{13}C{^{1}H} NMR** (C_6D_6, 126 MHz): 143.65 (*C*Aryl), 128.30 (*C*Aryl), 127.97 (*C*Aryl), 126.63 (*C*Aryl), 82.48 (O*C*(CH$_3$)$_2$, 47.81 (N*C*H$_2$), 24.71 (OC(*C*H$_3$)$_2$. **^{11}B{^{1}H} NMR** (C_6D_6, 160 MHz): 26.59 (bs).

Experimental data to propose mechanistic pathway of hydroboration reaction of benzonitrile.

To further elucidate the reaction mechanism, we performed the controlled experiments. At first, a stoichiometric reaction (1: 2) between the complex **1** and HBpin was carried out. As a result, an Mg-H was obtained which was characterized by ^{1}H, and ^{11}B{^{1}H} NMR spectroscopy.

^{1}H NMR (C_6D_6, 500 MHz): δ 7.15 (bs, 4H, CH_{Aryl}), 7.08 (m, 8H, CH_{Aryl}), 6.94 (d, 2H, J = 7.76 Hz, CH_{Aryl}), 4.85 (s, 2H, C$H_{pentene}$), 3.56 (s, 2H, Zn-*H*), 3.34 (m, 4H, CH_{Dipp}), 1.87 (s, 6H, CH_3), 1.56 (s, 6H, CH_3), 1.17 (d, J = 7 Hz, 12H, CH_{3Dipp}). **^{11}B{^{1}H} NMR** (C_6D_6, 160 MHz): δ 34.65 (Et-Bpin).

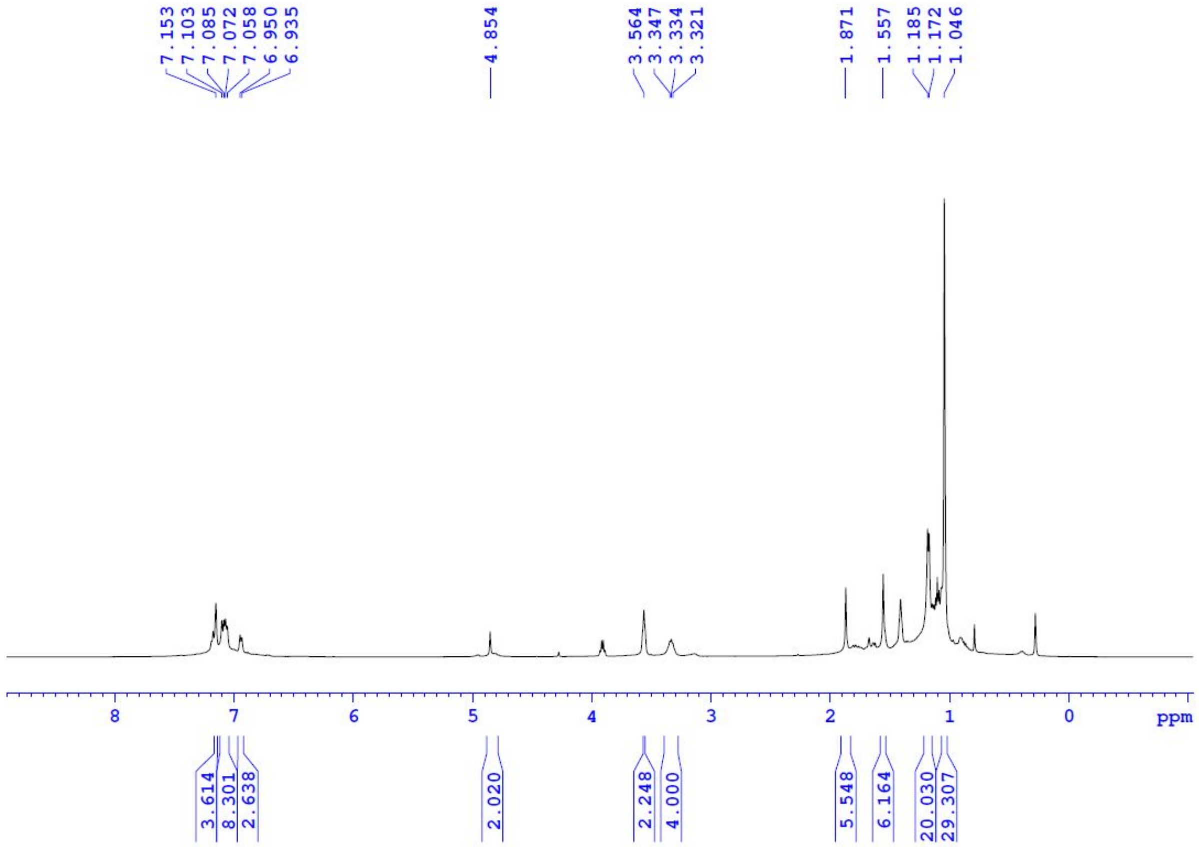

Figure S39. ^{1}H NMR spectrum of Zn complex **1** + HBpin.

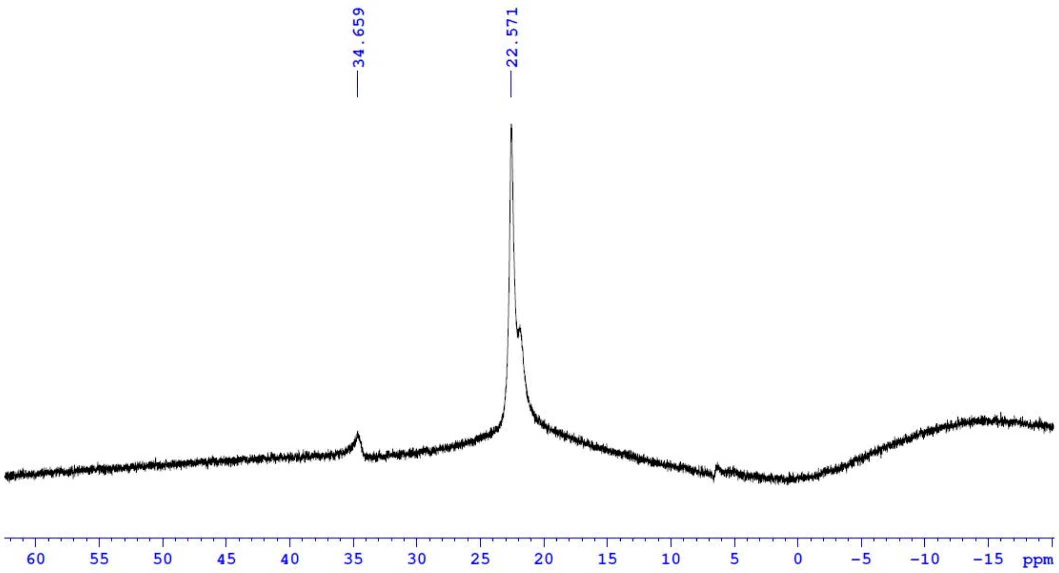

Figure S40. $^{11}B\{^{1}H\}$ NMR spectrum of Zn complex **1** + HBpin.

Next, 1 eq. of benzonitrile was added to the reaction mixture containing Zn-H, followed by heating for 12 hours at 60 °C. Upon recording the ^{1}H NMR spectrum, the Zn-H peak at 3.5 ppm had disappeared, and a new peak at 4.3 ppm corresponding to $PCH_2N(Bpin)_2$ was observed, along with unreacted benzonitrile. Subsequently, an additional eq. of HBpin was added to the same NMR tube and the mixture was heated again for 12 hours at 60 °C. The ^{1}H NMR spectrum revealed complete consumption of the remaining benzonitrile, with only product peaks detected.

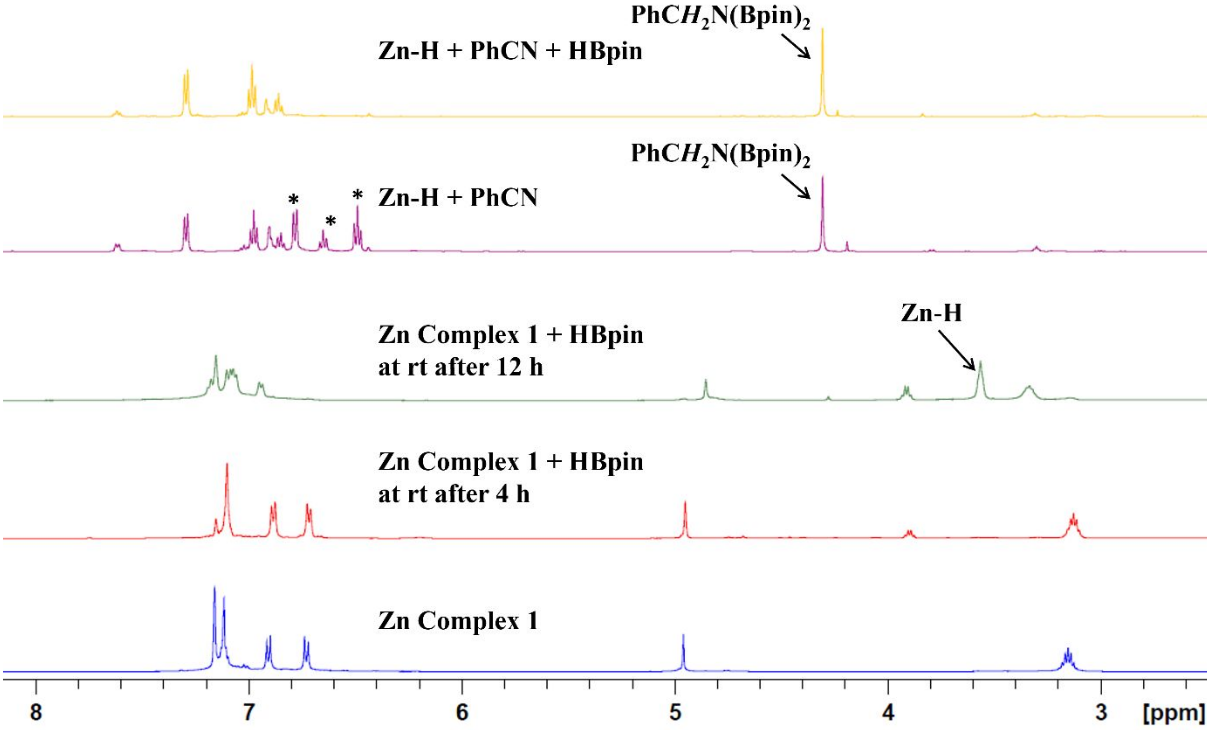

Figure S41. ^{1}H NMR (500 MHz) stack plot of the stoichiometric reaction of Zn complex 1 with HBpin in C_6D_6. Stacked spectra from bottom to top: Zn complex **1**; Zn complex **1 +** HBpin at rt after 4 h; Zn complex **1 +** HBpin at rt after 12 h; Zn-H + PhCN (* = unreacted PhCN); Zn-H + PhCN + 1 eq. HBpin.

NMR spectra for hydroboration of nitriles

In ^{11}B NMR we observed a peak at 34 ppm for Et-Bpin and another peak at 22 ppm for $B(OR)_3$.

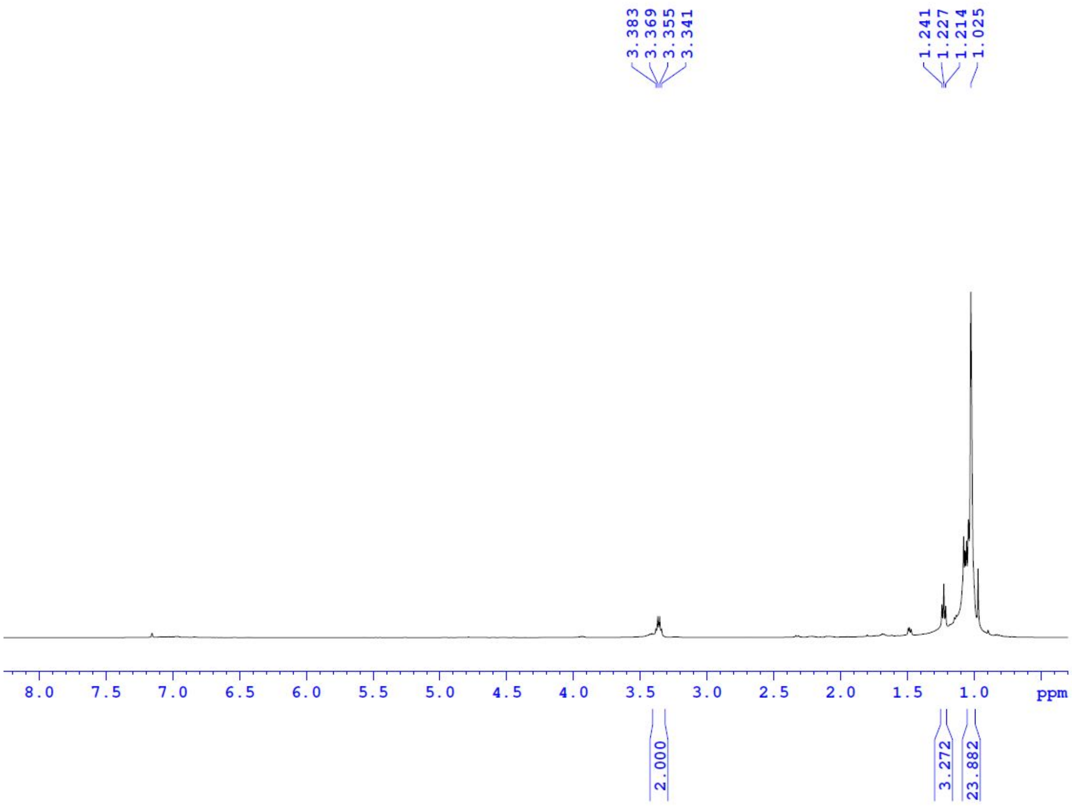

Figure S42. ^{1}H NMR spectrum of **1a.**

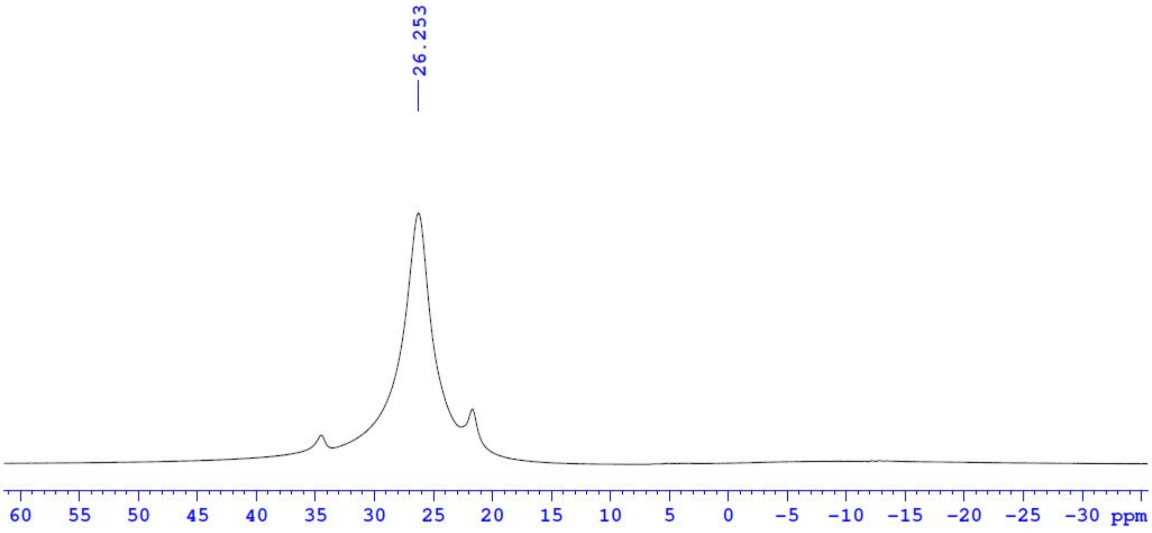

Figure S43. $^{11}B\{^{1}H\}$ NMR spectrum of **1a.**

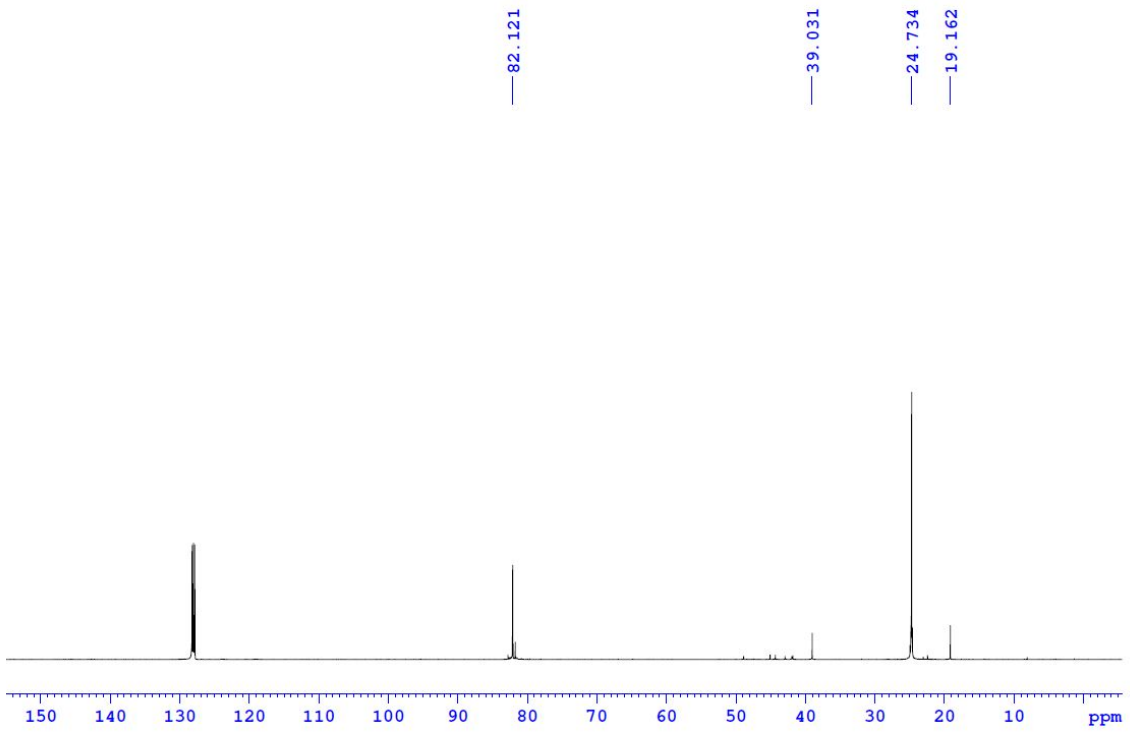

Figure S44. $^{13}C\{^{1}H\}$ NMR spectrum of **1a.**

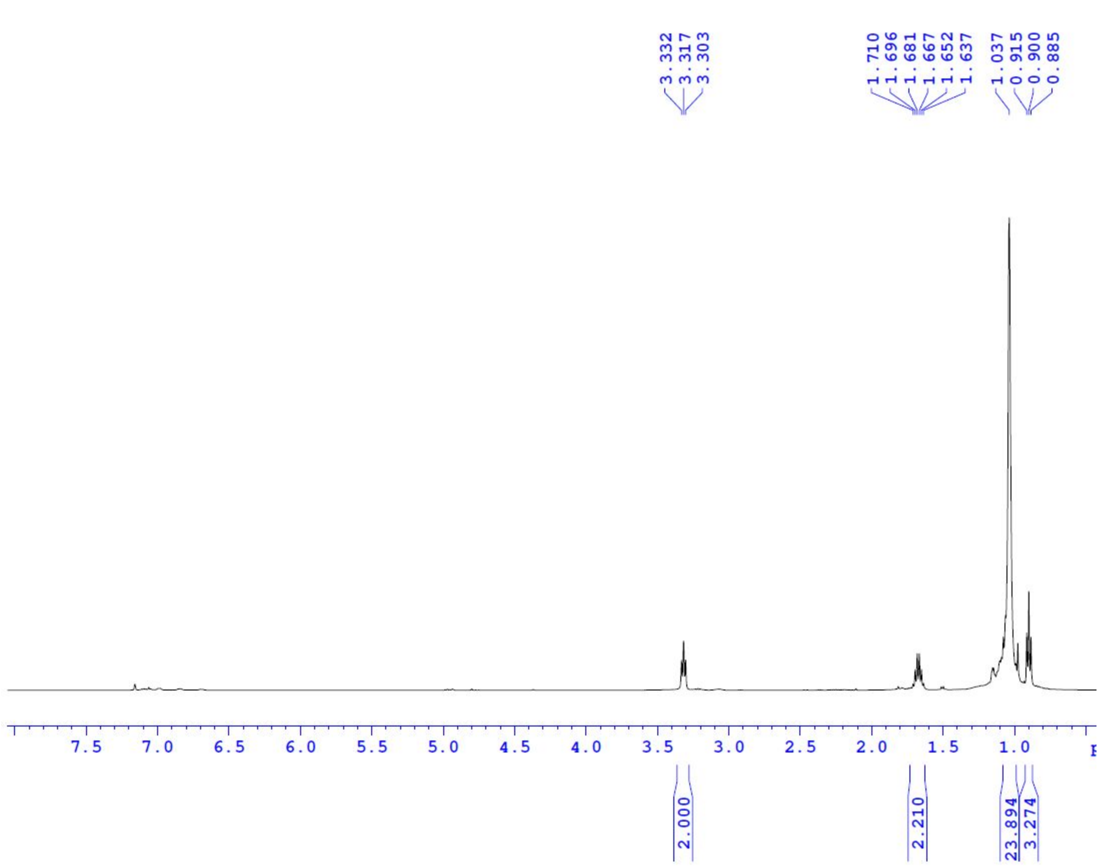

Figure S45. ^{1}H NMR spectrum of **1b.**

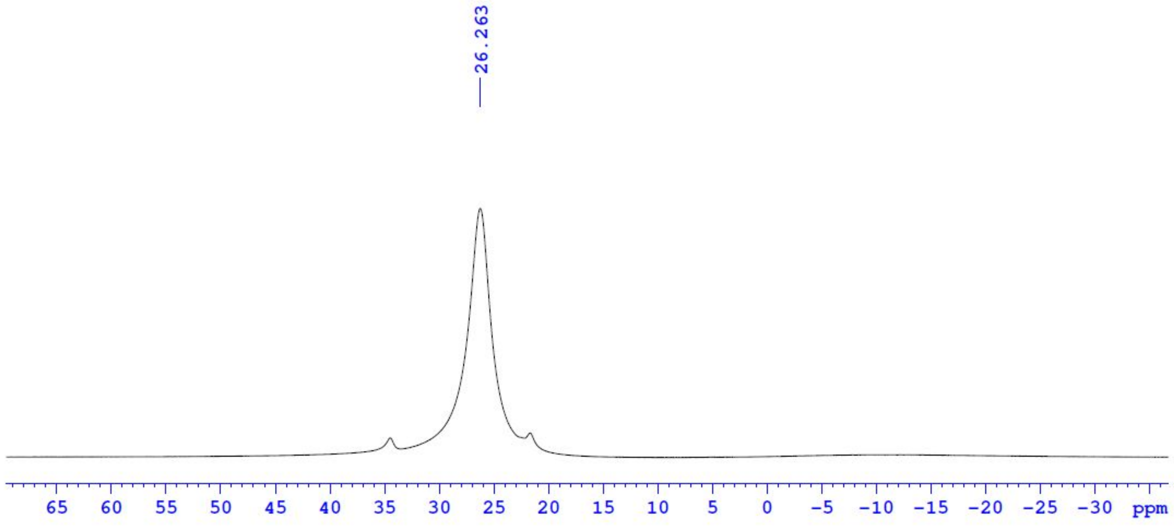

Figure S46. $^{11}B\{^{1}H\}$ NMR spectrum of **1b.**

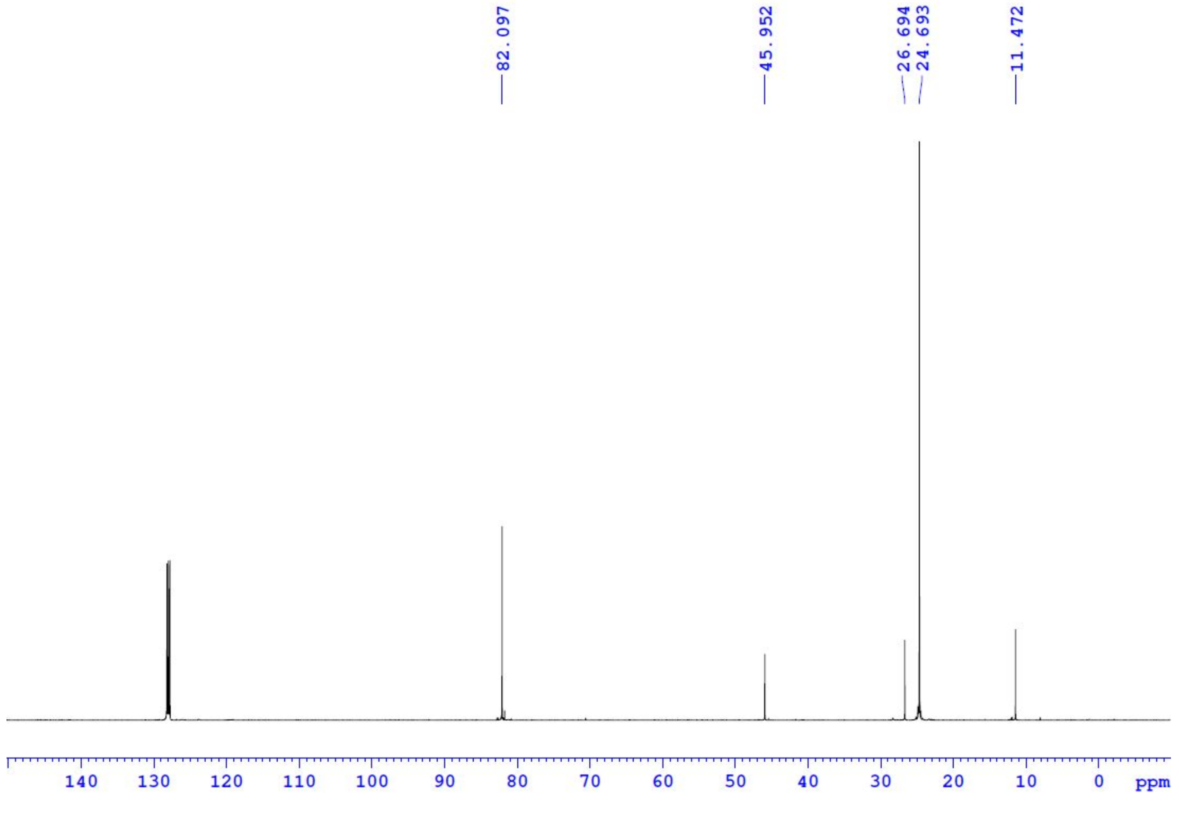

Figure S47. $^{13}C\{^{1}H\}$ NMR spectrum of **1b.**

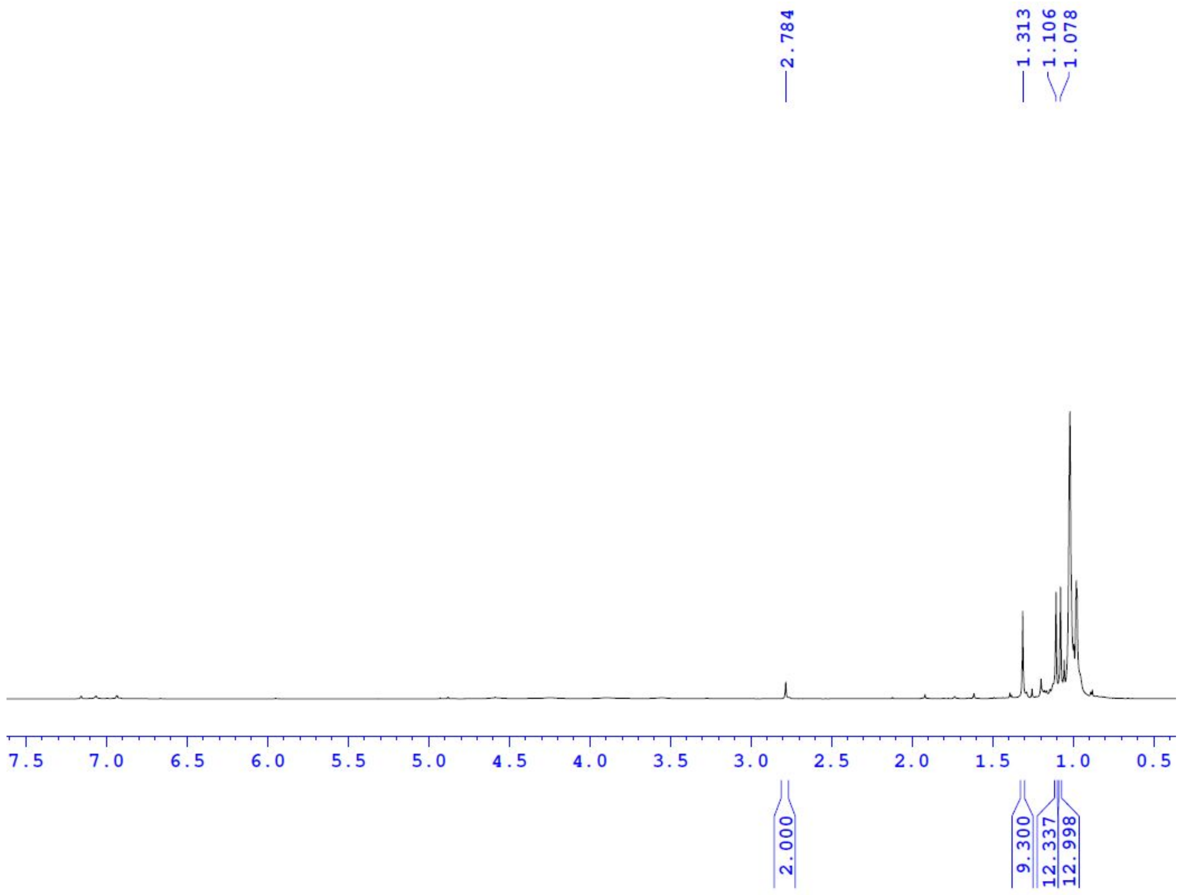

Figure S48. ^{1}H NMR spectrum of **1c.**

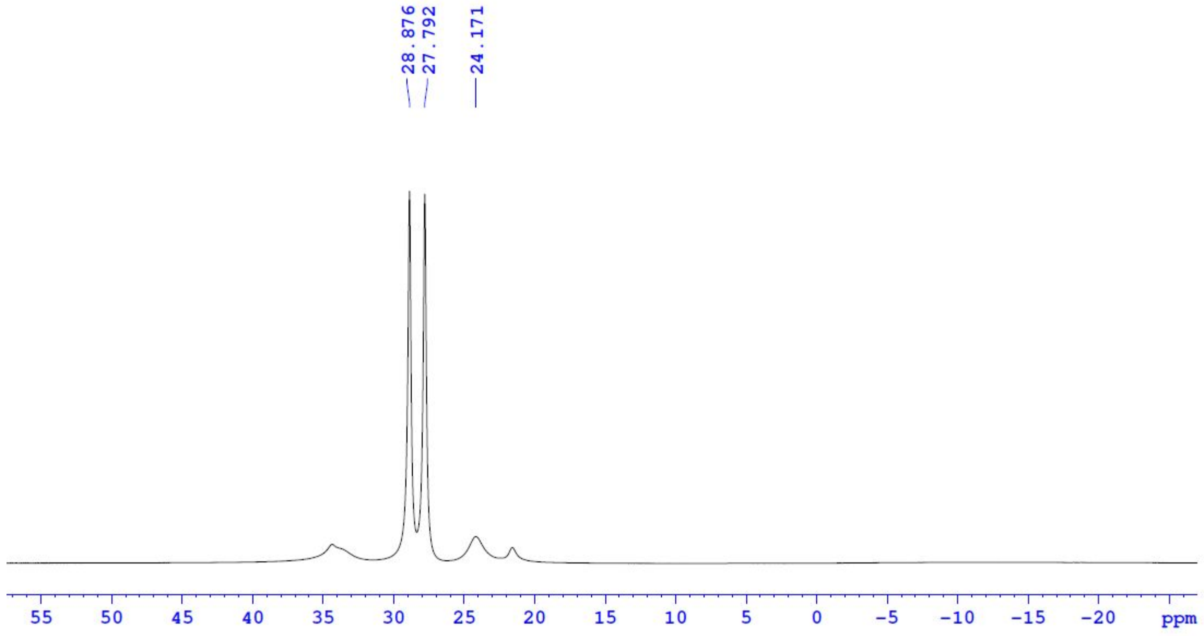

Figure S49. $^{11}B\{^{1}H\}$ NMR spectrum of **1c.**

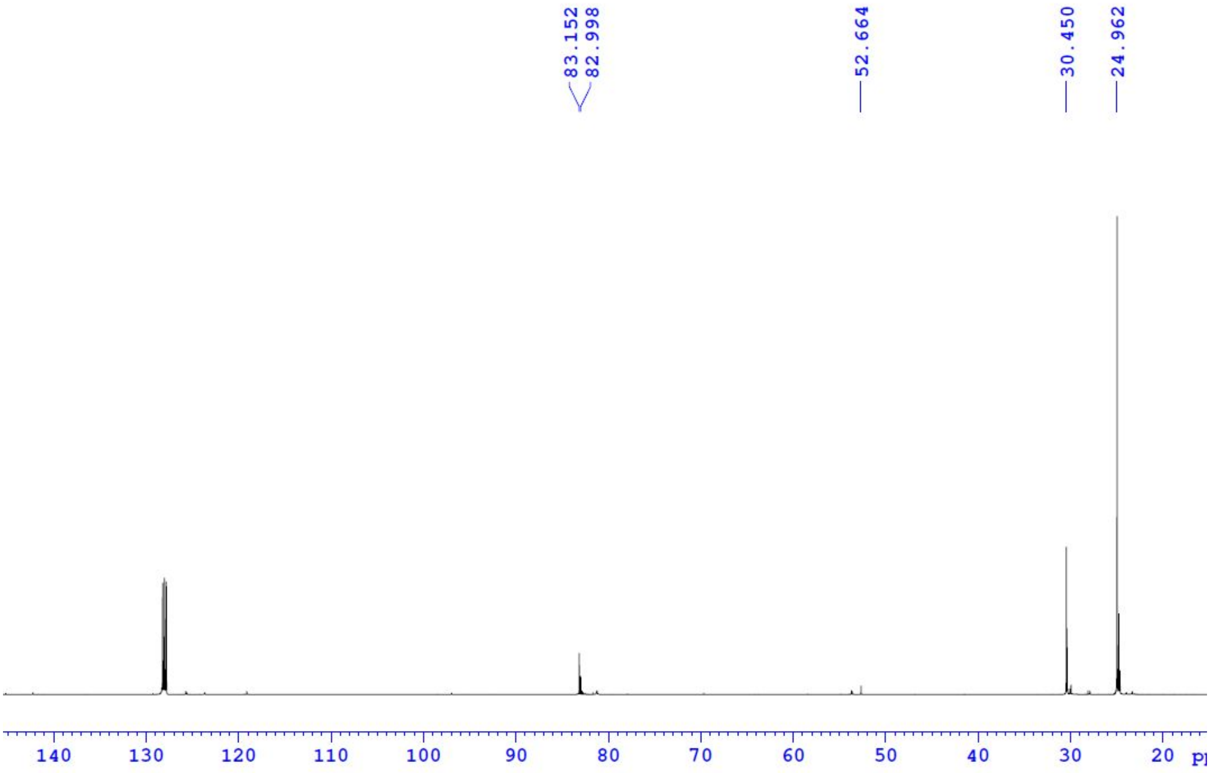

Figure S50. $^{13}C\{^{1}H\}$ NMR spectrum of **1c.**

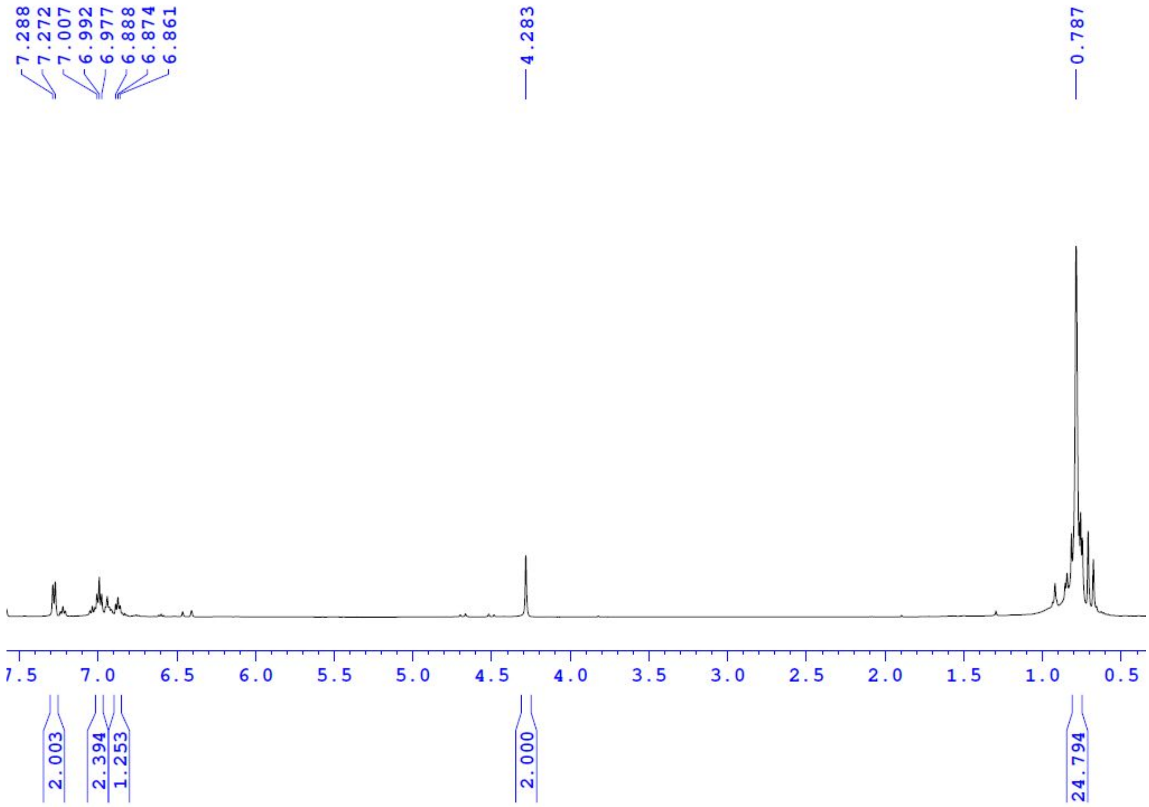

Figure S51. ^{1}H NMR spectrum of **1d.**

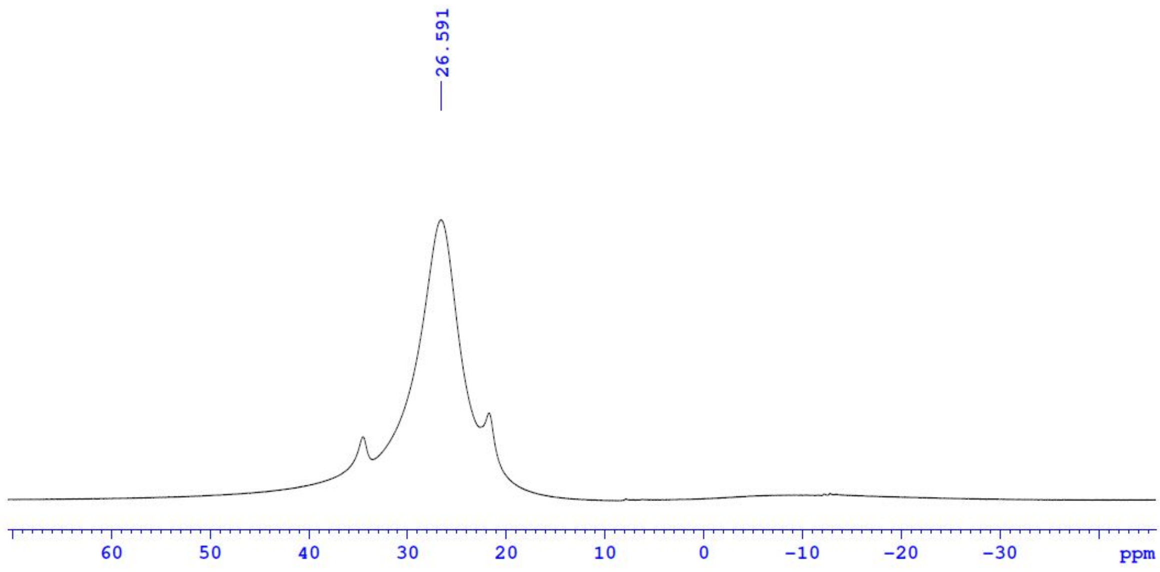

Figure S52. $^{11}B\{^1H\}$ NMR spectrum of **1d.**

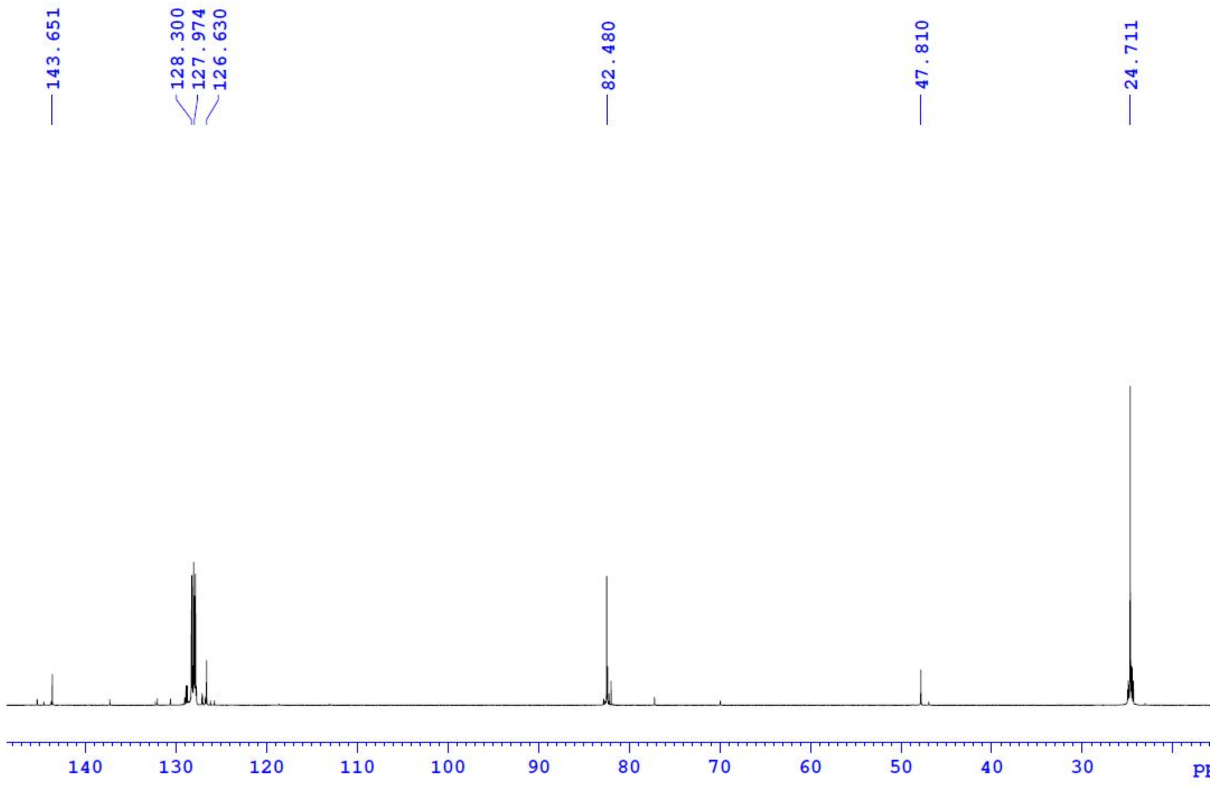

Figure S53. $^{13}C\{^1H\}$ NMR spectrum of **1d.**

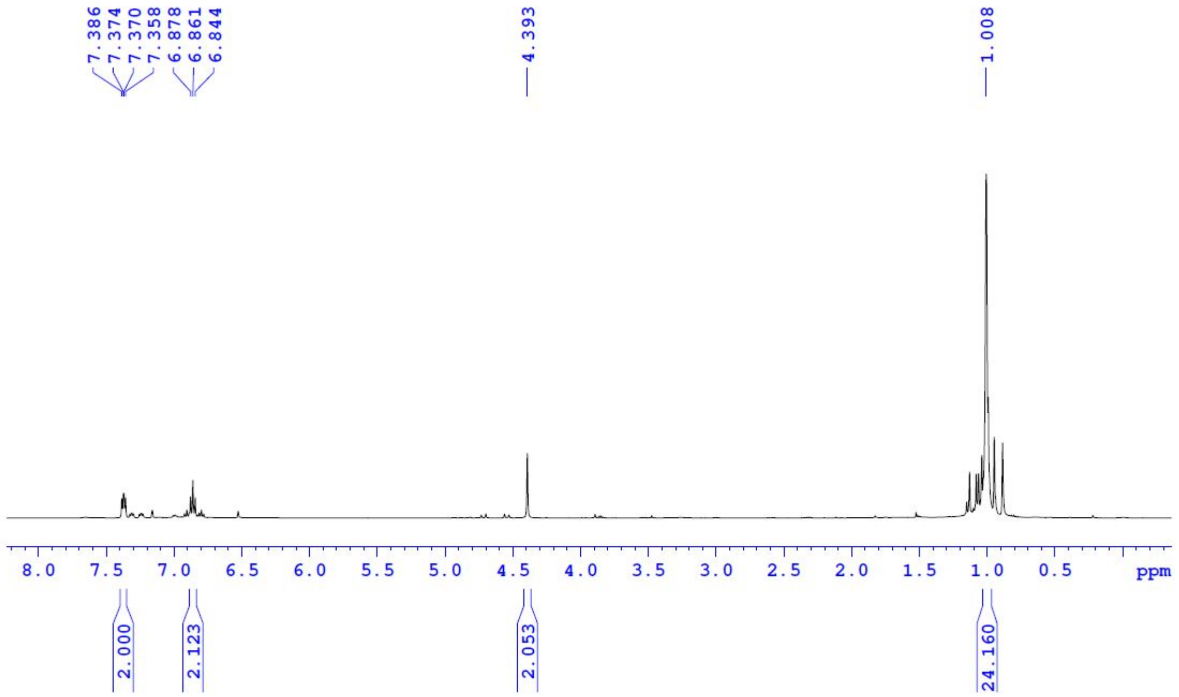

Figure S54. ^{1}H NMR spectrum of **1e.**

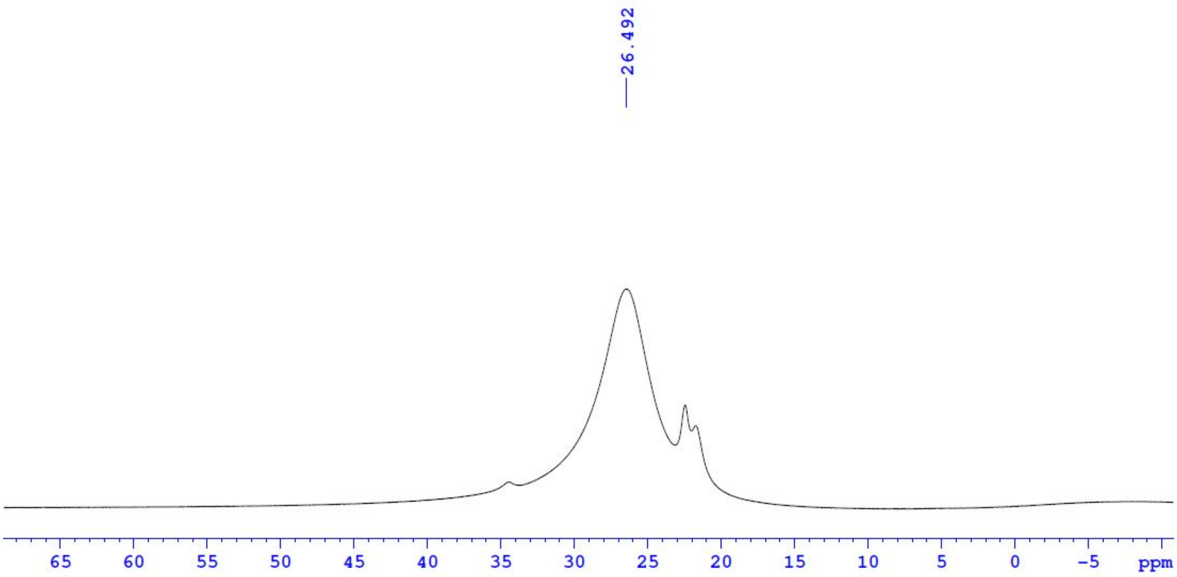

Figure S55. $^{11}B\{^{1}H\}$ NMR spectrum of **1e.**

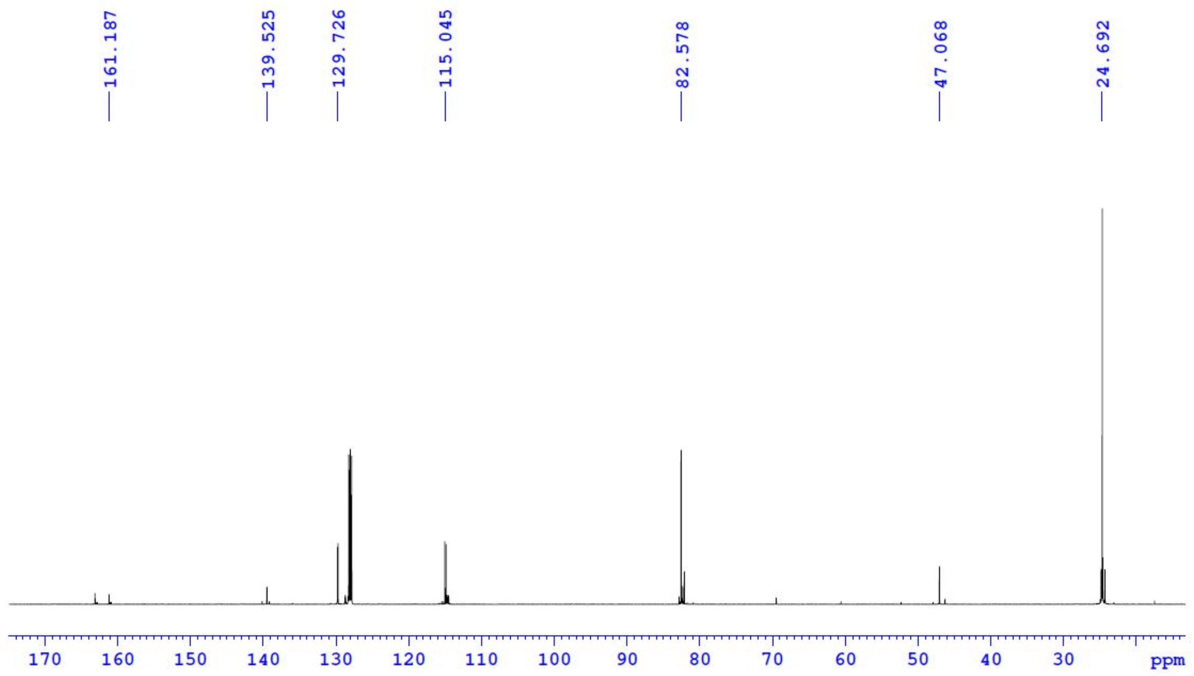

Figure S56. $^{13}C\{^1H\}$ NMR spectrum of **1e.**

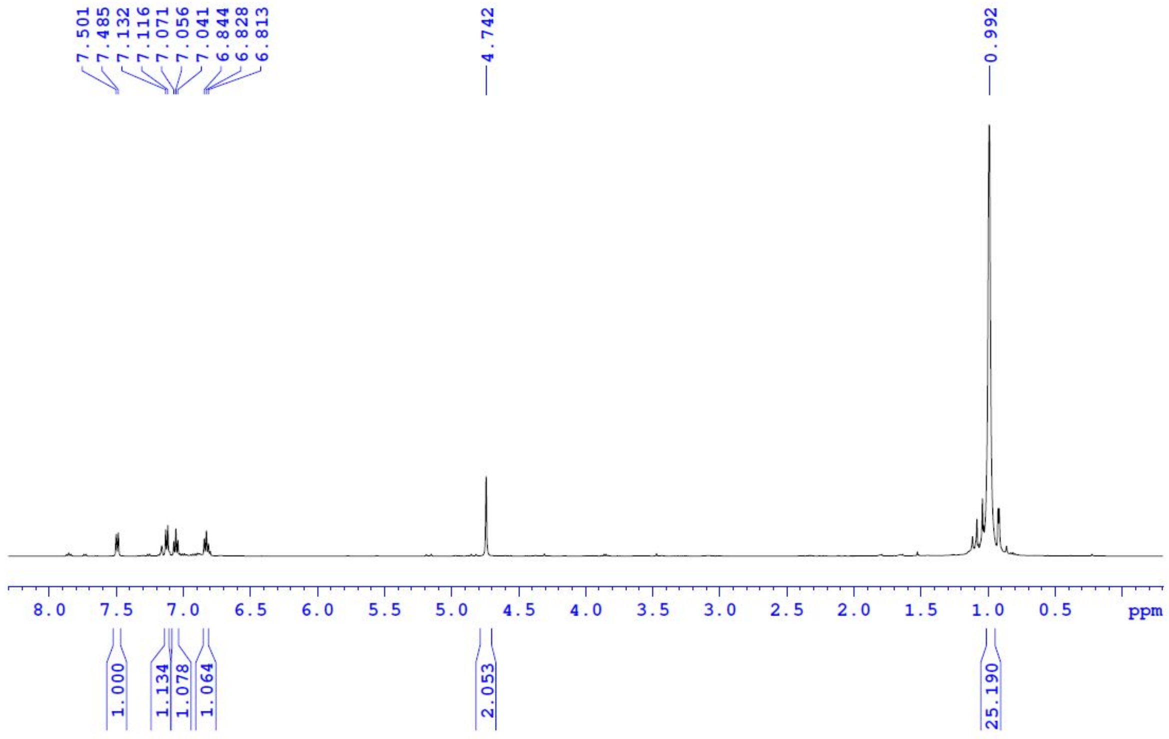

Figure S57. ^1H NMR spectrum of **1f.**

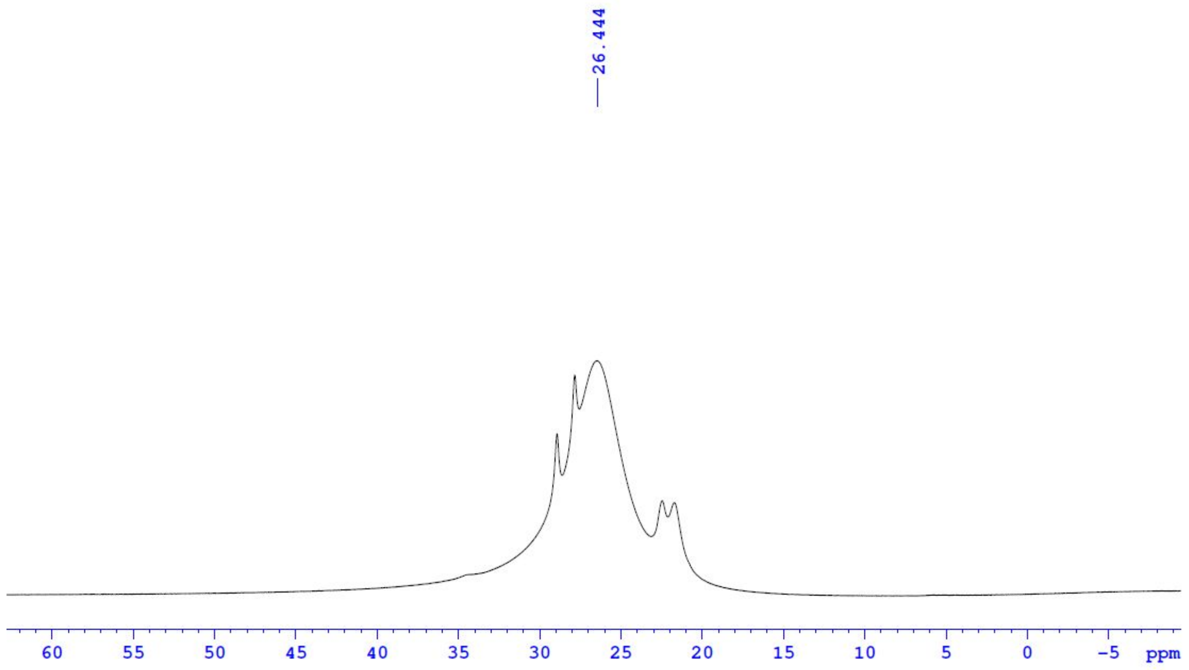

Figure S58. $^{11}B\{^{1}H\}$ NMR spectrum of **1f.**

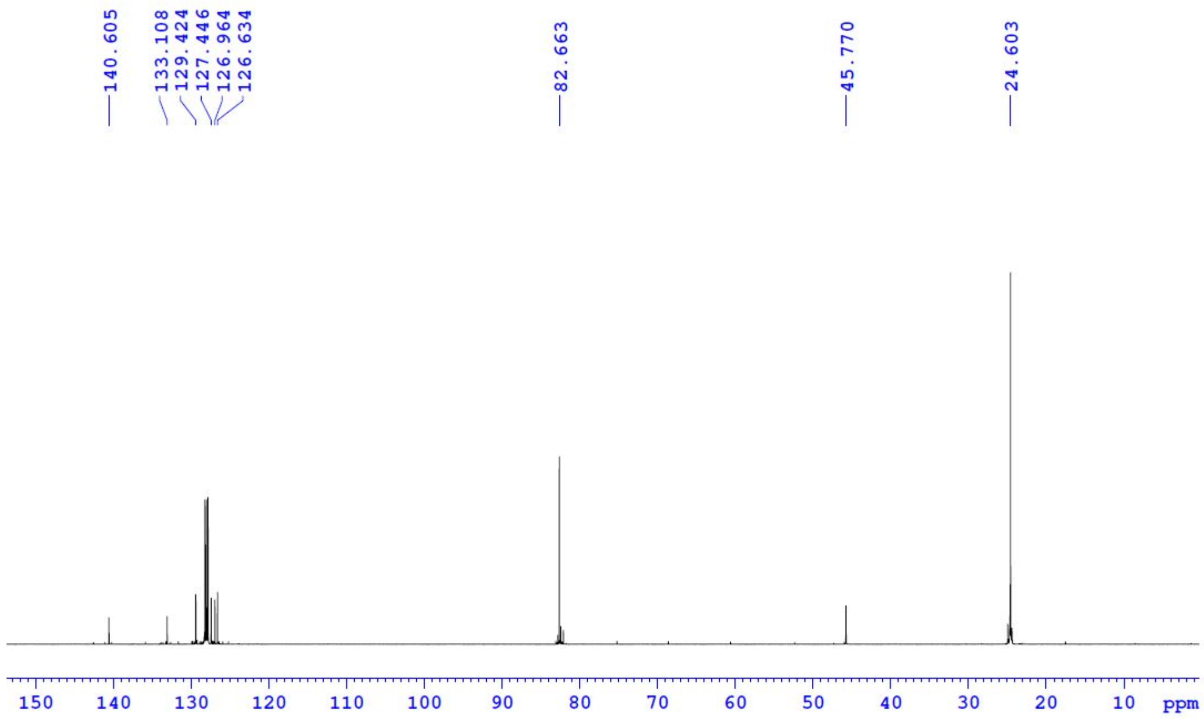

Figure S59. $^{13}C\{^{1}H\}$ NMR spectrum of **1f.**

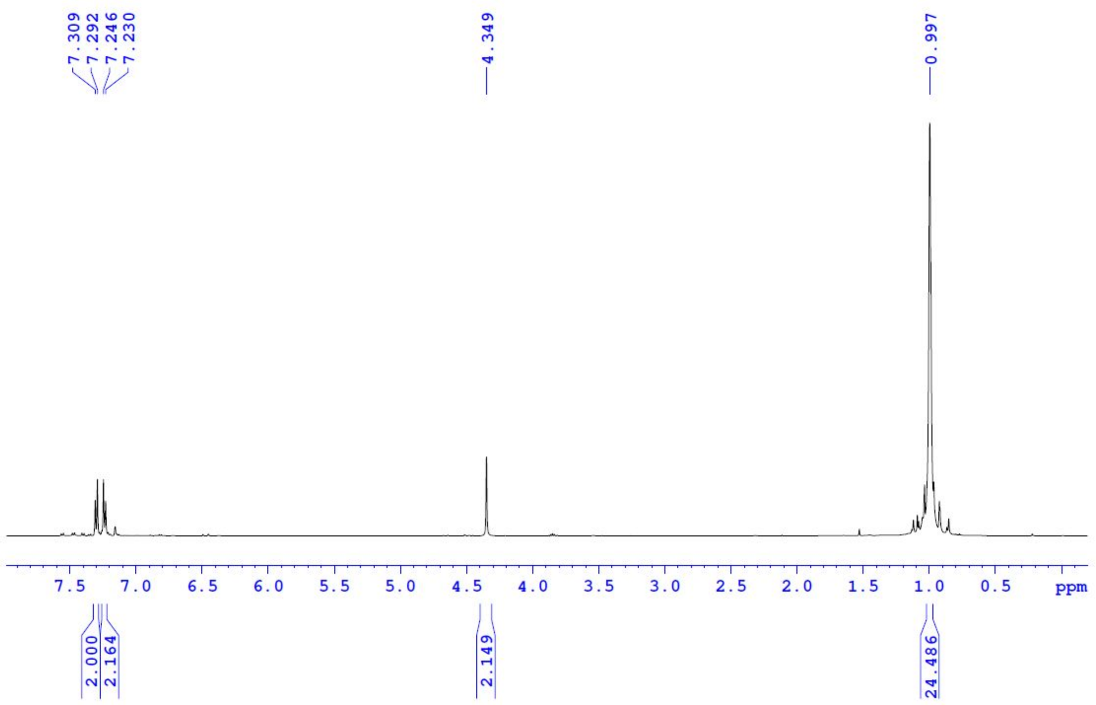

Figure S60. ^{1}H NMR spectrum of **1g.**

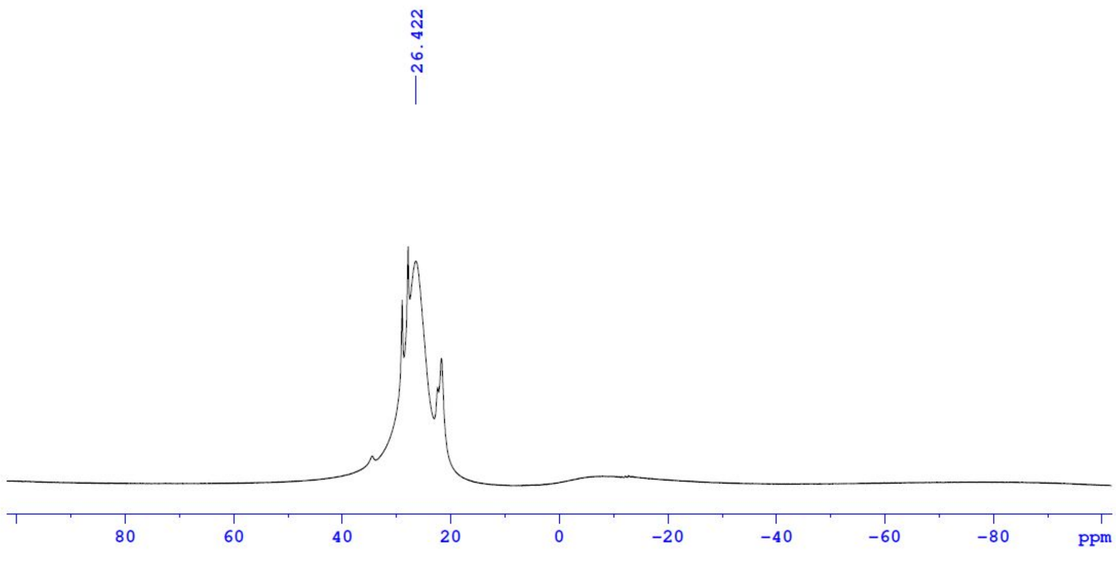

Figure S61. $^{11}B\{^{1}H\}$ NMR spectrum of **1g.**

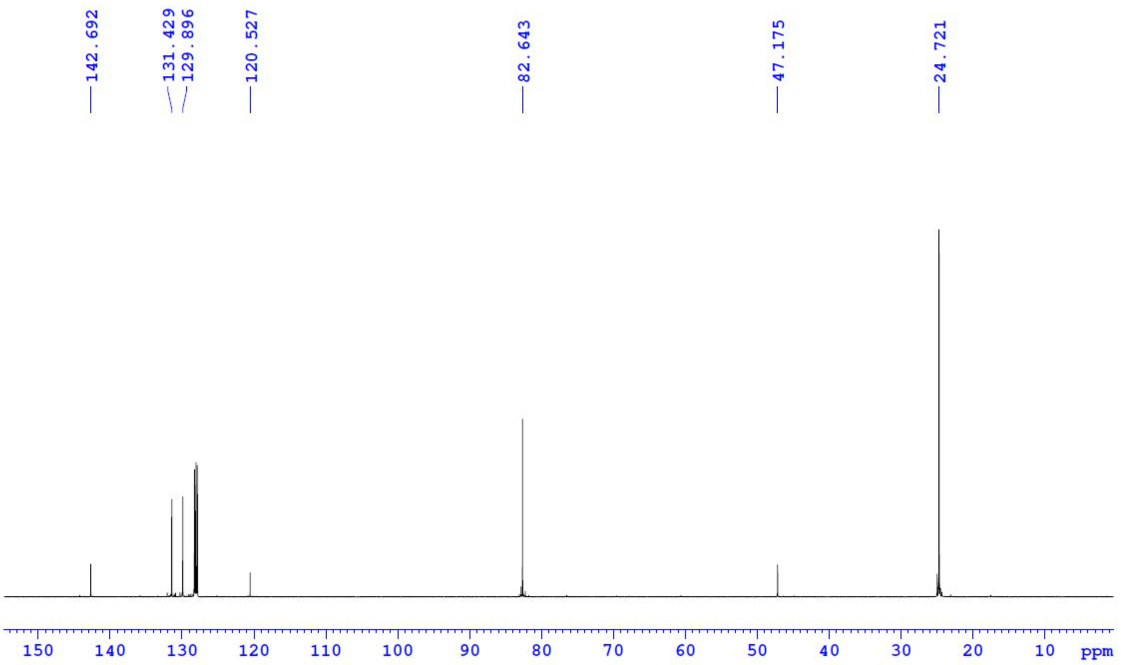

Figure S62. $^{13}C\{^{1}H\}$ NMR spectrum of **1g.**

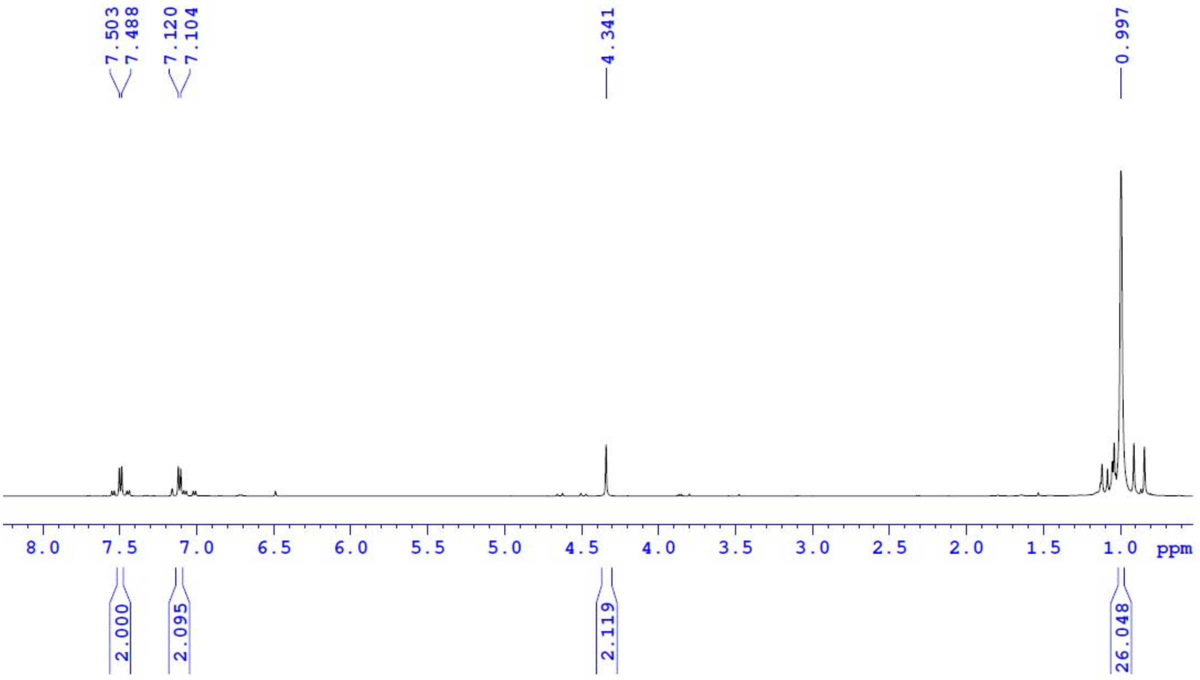

Figure S63. ^{1}H NMR spectrum of **1h.**

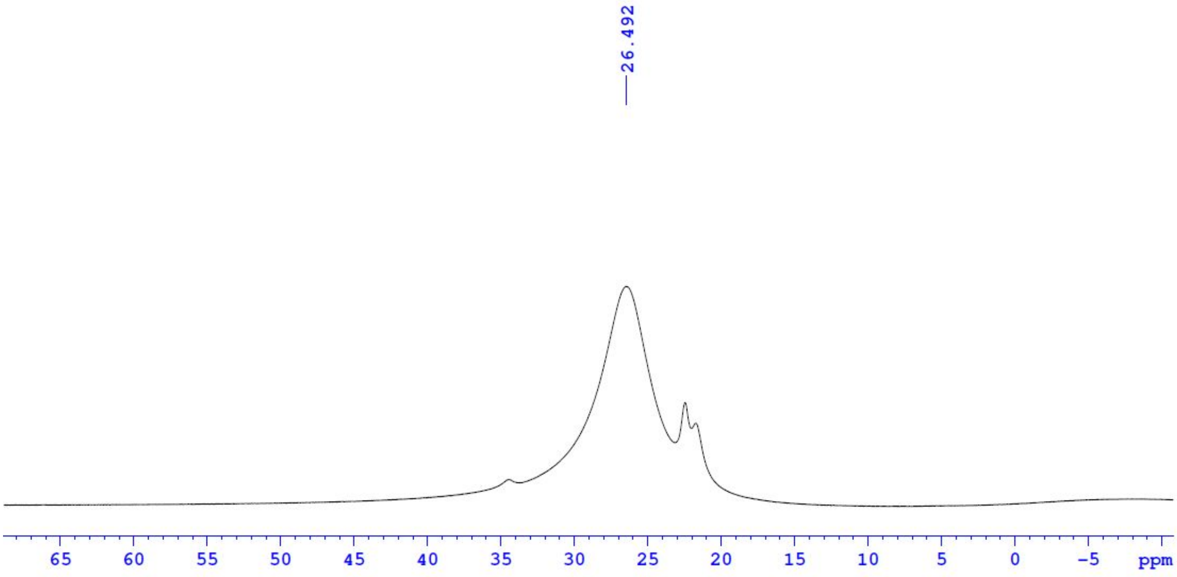

Figure S64. $^{11}B\{^{1}H\}$ NMR spectrum of **1h.**

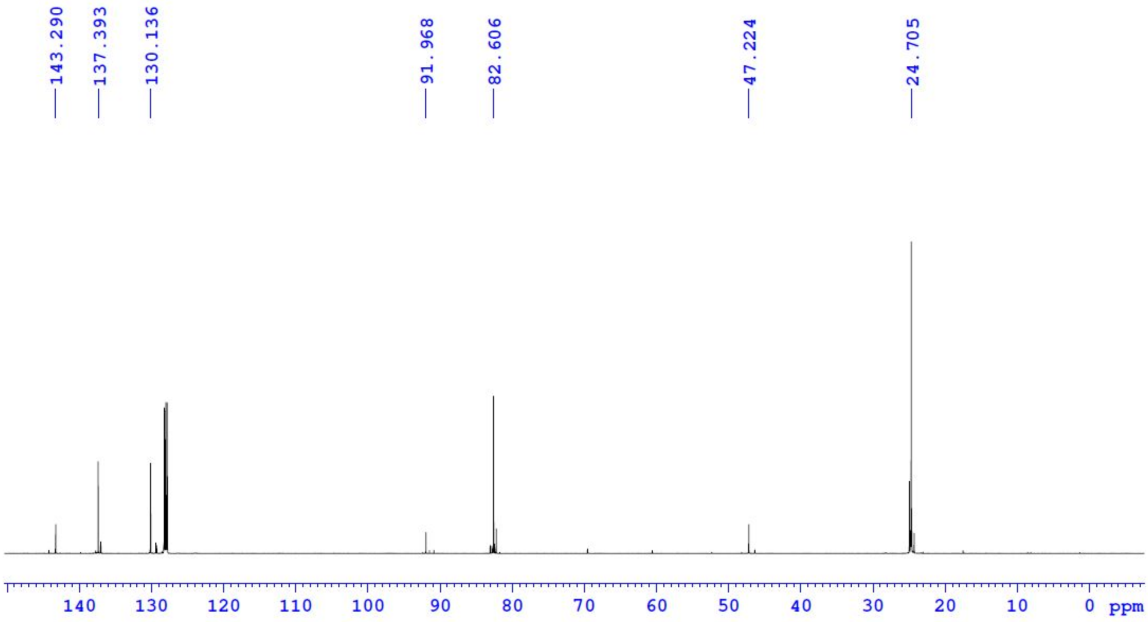

Figure S65. $^{13}C\{^{1}H\}$ NMR spectrum of **1h.**

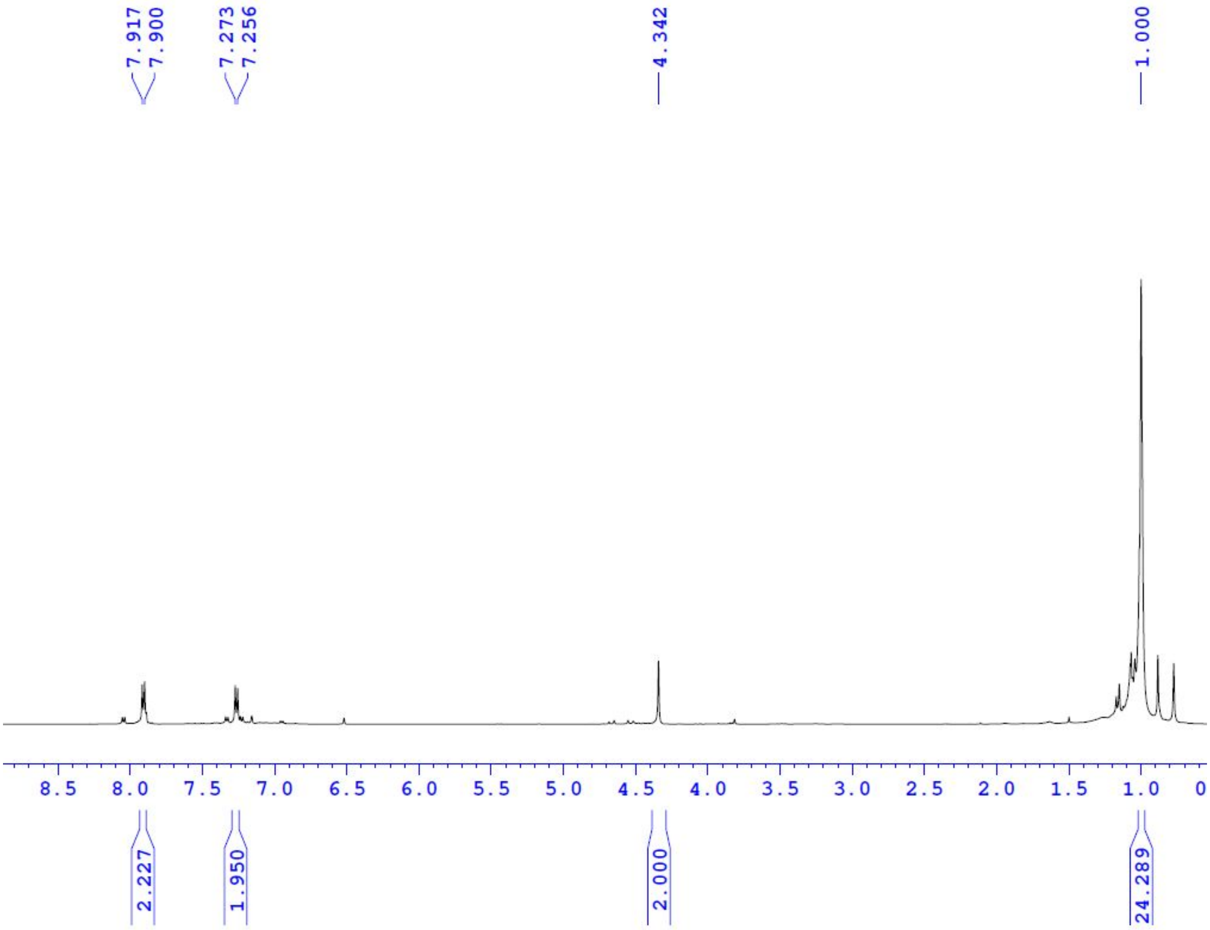

Figure S66. ^{1}H NMR spectrum of **1i.**

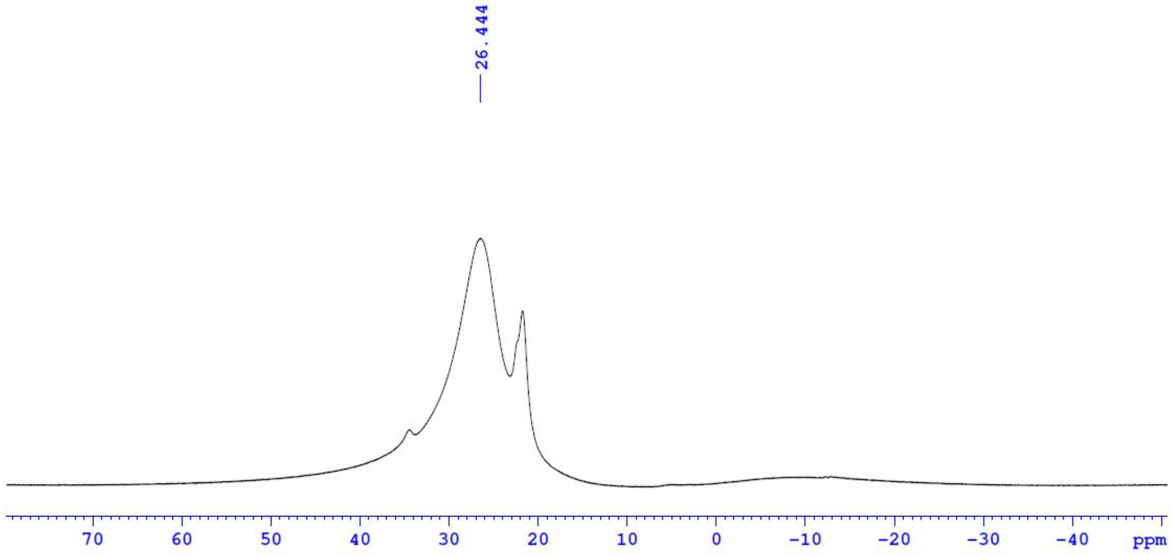

Figure S67. $^{11}B\{^{1}H\}$ NMR spectrum of **1i.**

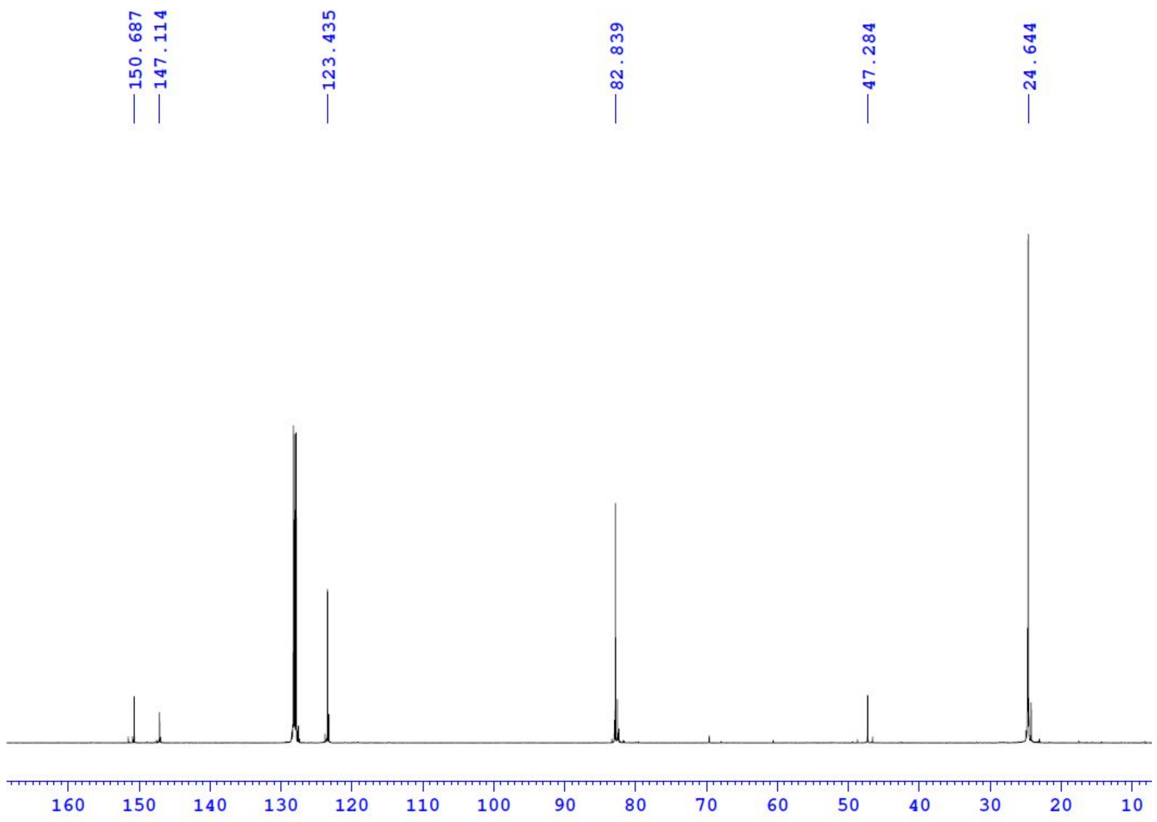

Figure S68. $^{13}C\{^{1}H\}$ NMR spectrum of **1i.**

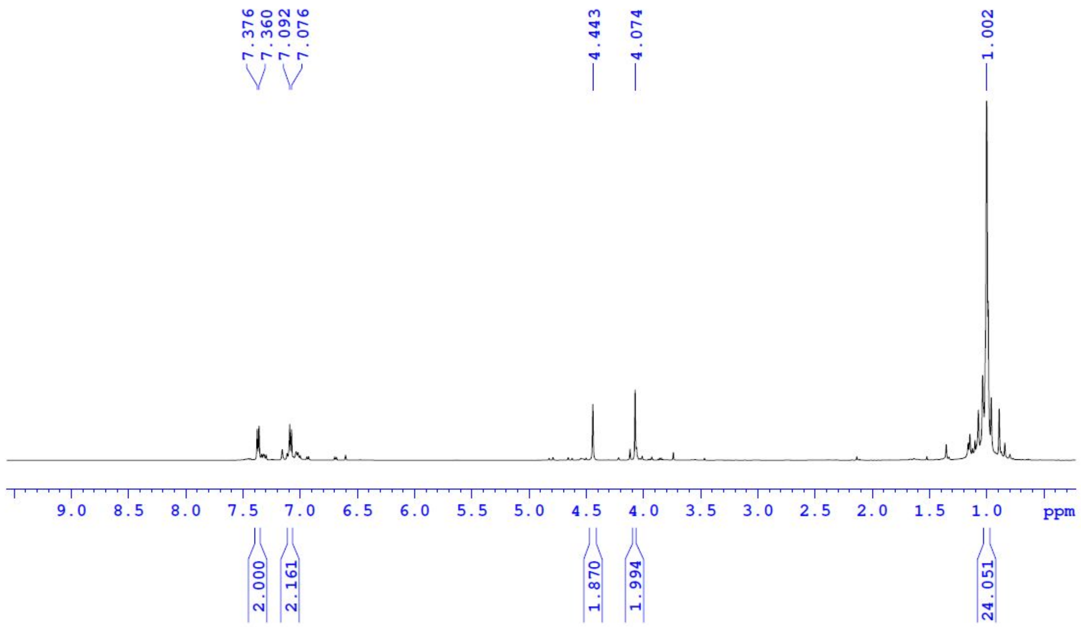

Figure S69. ^{1}H NMR spectrum of **1j.**

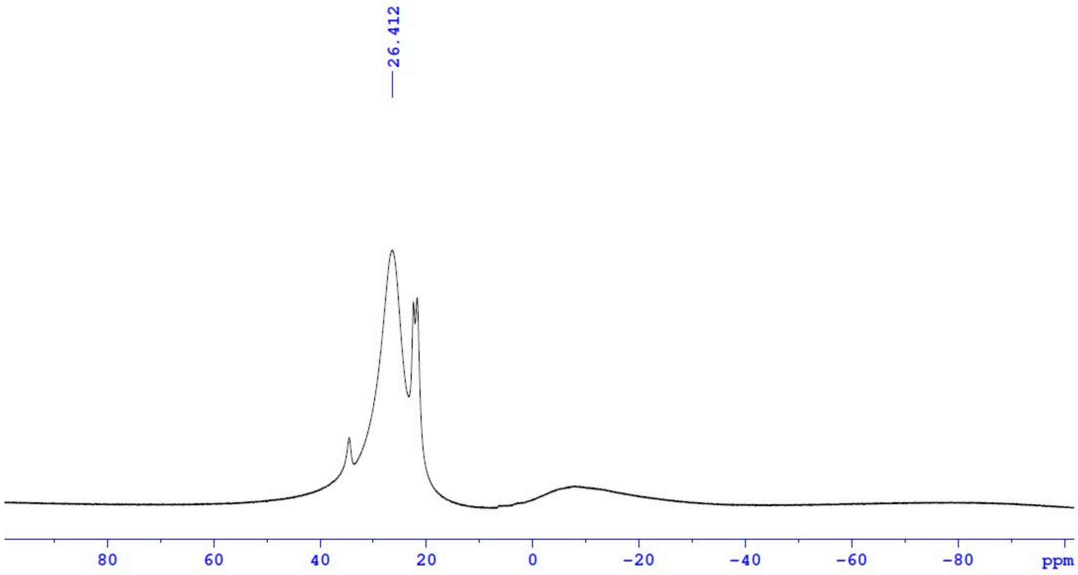

Figure S70. $^{11}B\{^{1}H\}$ NMR spectrum of **1j.**

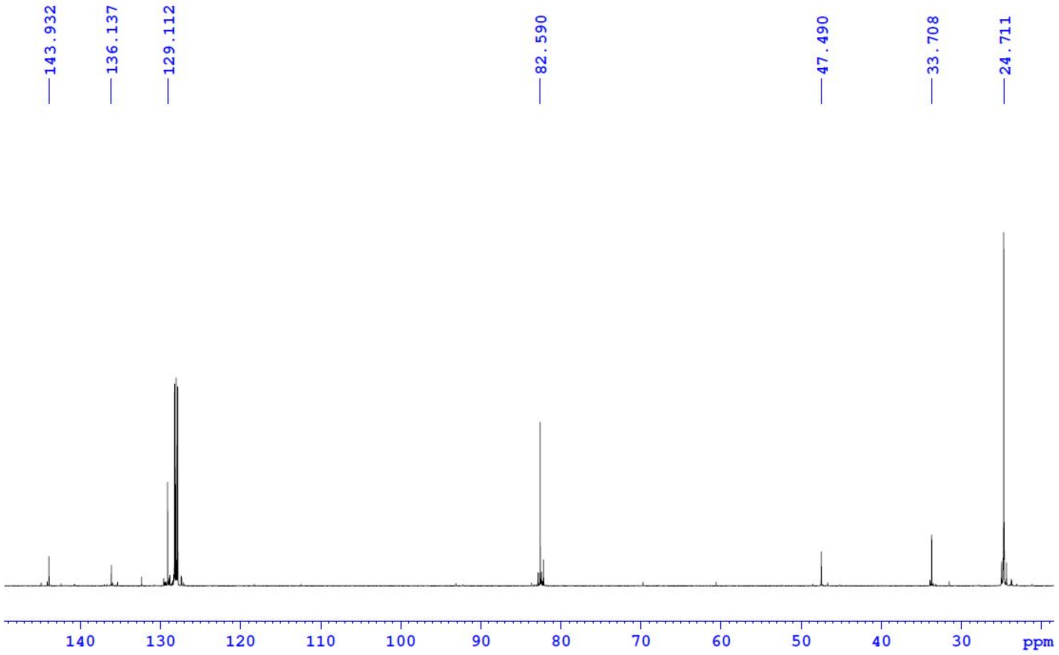

Figure S71. $^{13}C\{^{1}H\}$ NMR spectrum of **1j.**

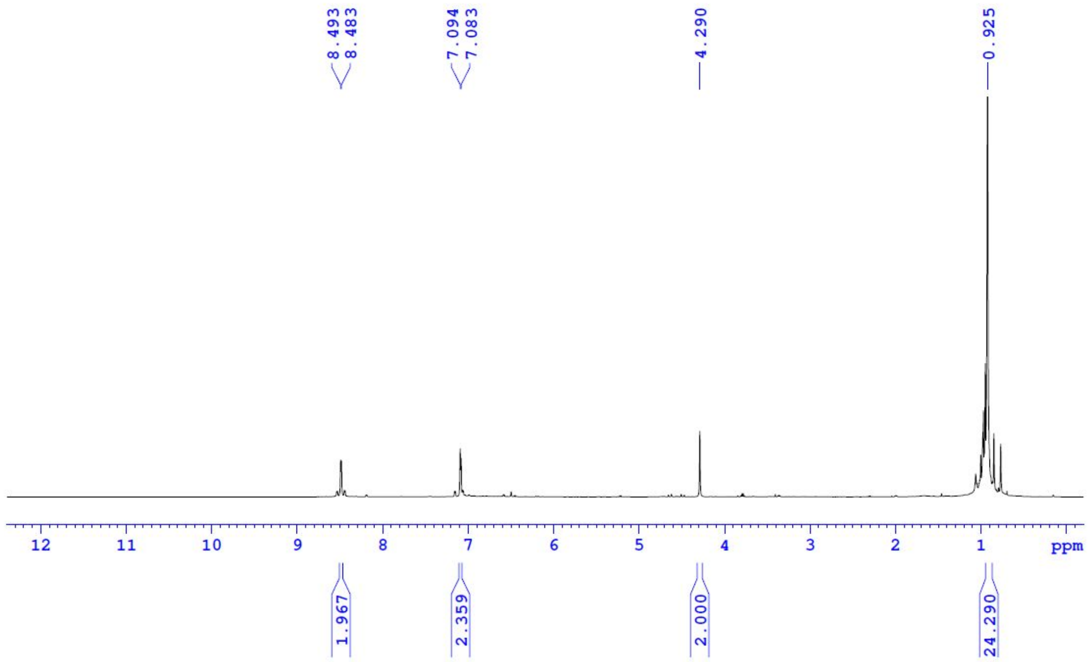

Figure S72. ^{1}H NMR spectrum of **1k.**

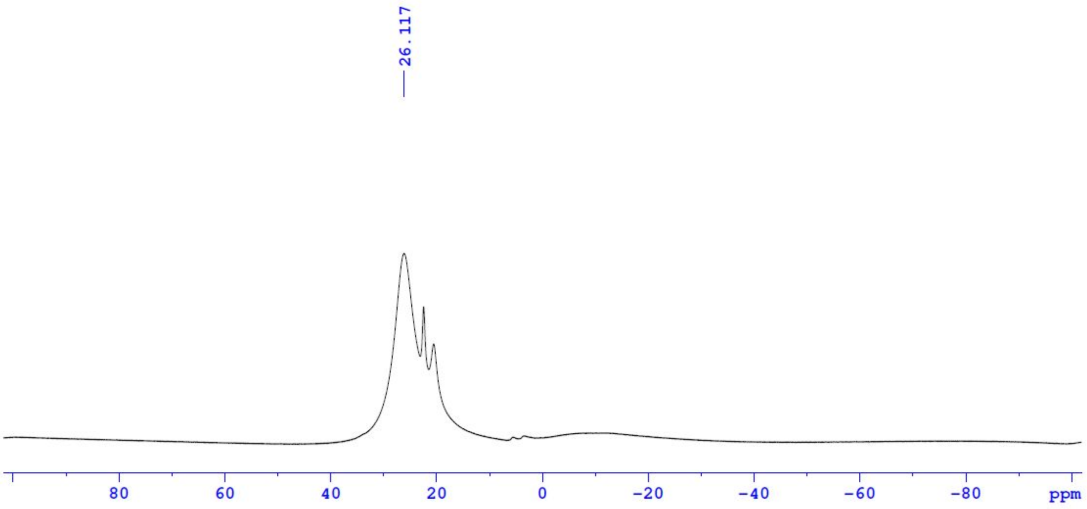

Figure S73. $^{11}B\{^{1}H\}$ NMR spectrum of **1k.**

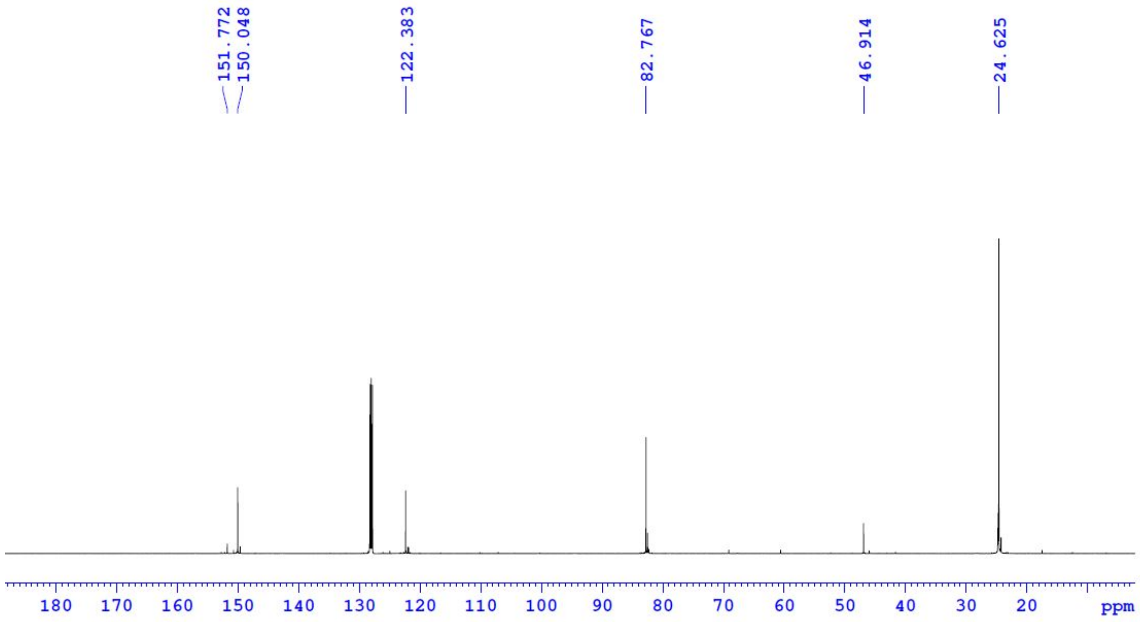

Figure S74. $^{13}C\{^1H\}$ NMR spectrum of **1k.**

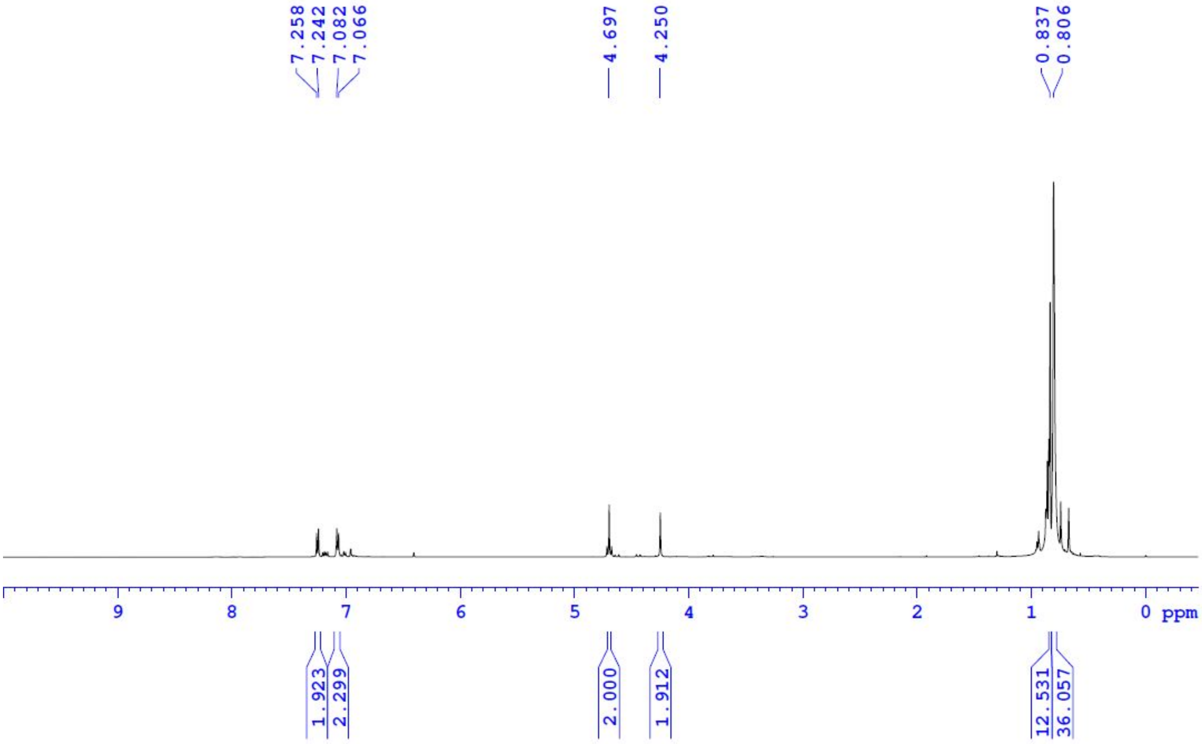

Figure S75. ^1H NMR spectrum of **1l.**

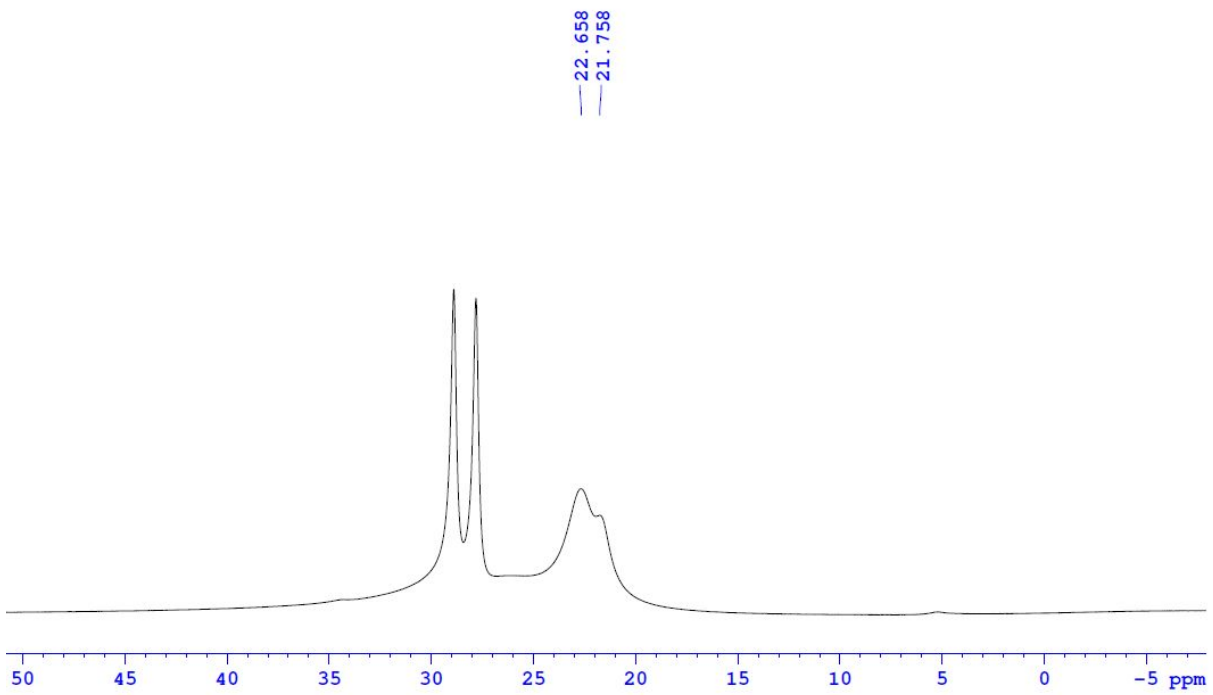

Figure S76. $^{11}B\{^{1}H\}$ NMR spectrum of **1l.**

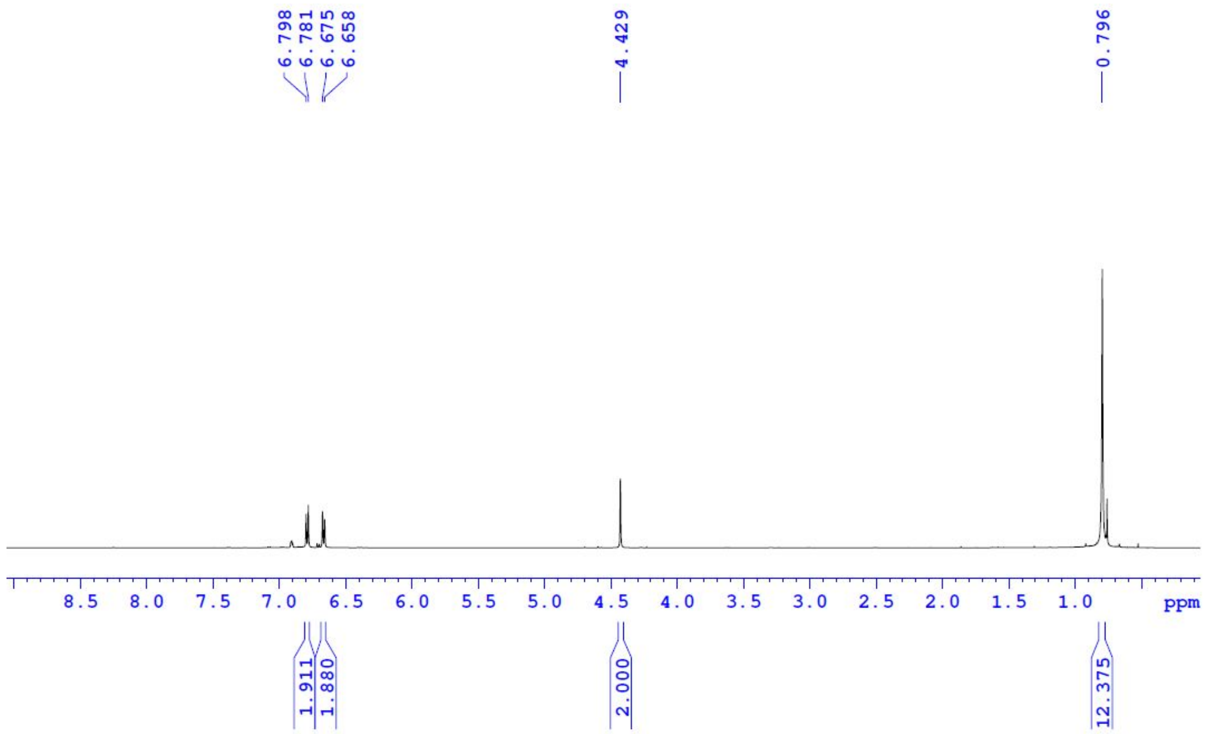

Figure S77. ^{1}H NMR spectrum of **1l′.**

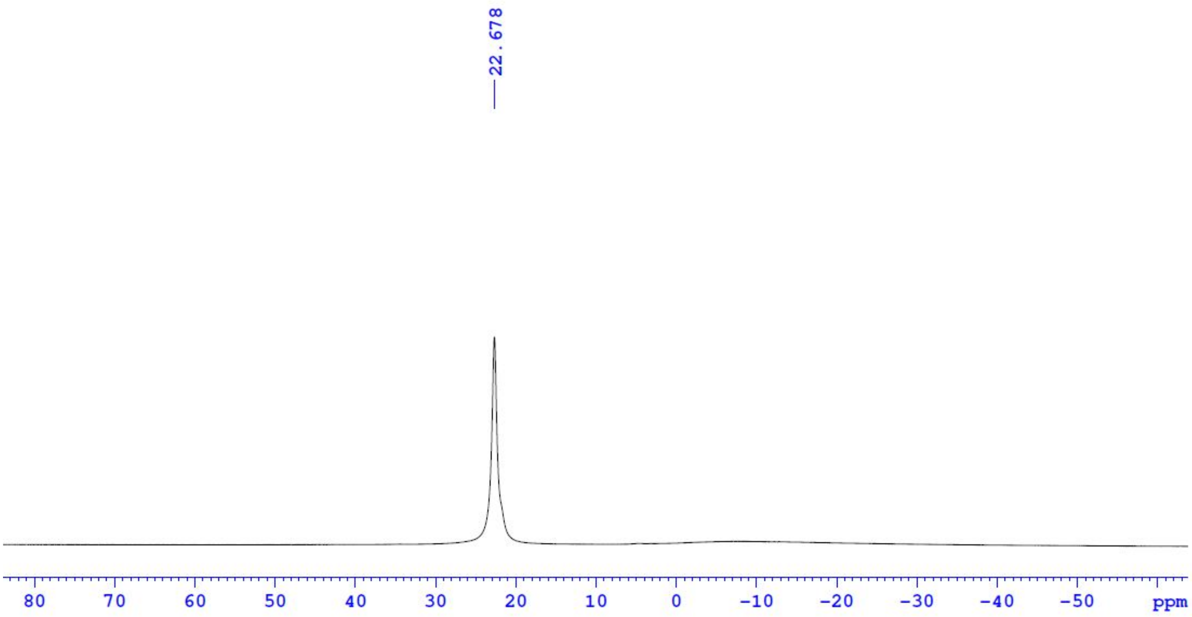

Figure S78. $^{11}B\{^{1}H\}$ NMR spectrum of **1l′**.

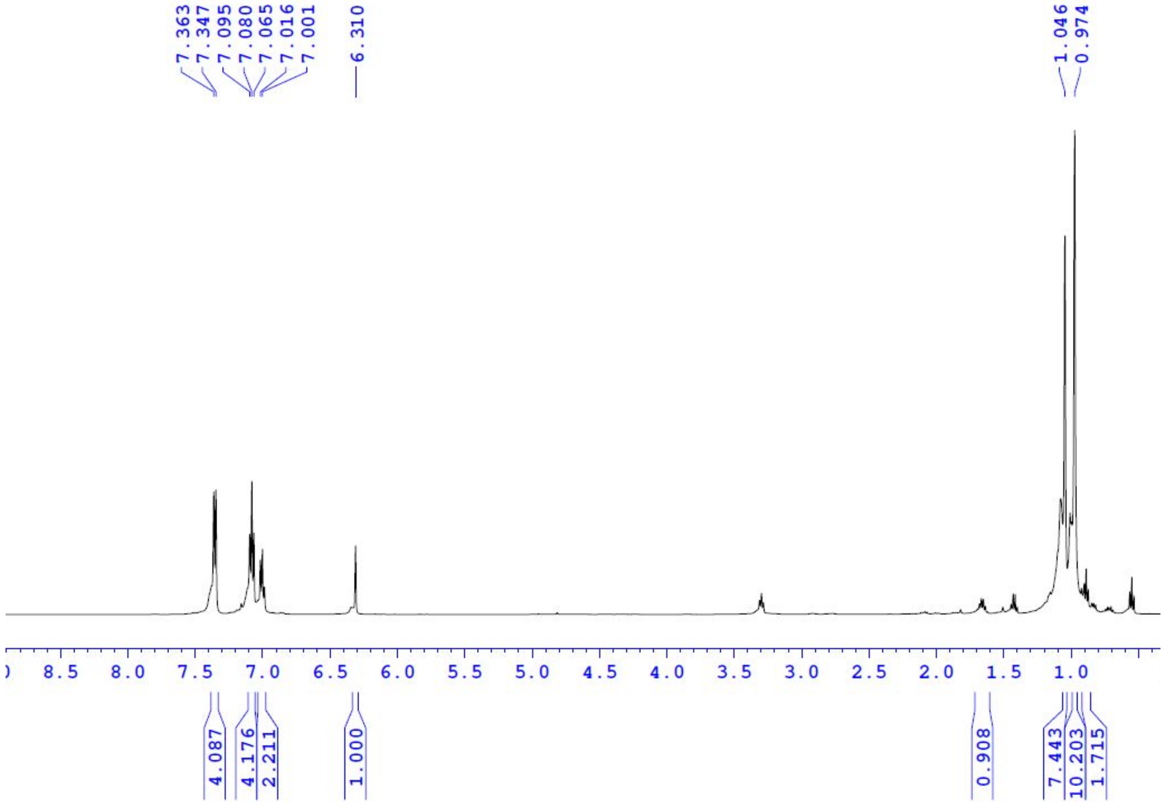

Figure S79. ^{1}H NMR spectrum of **1m.**

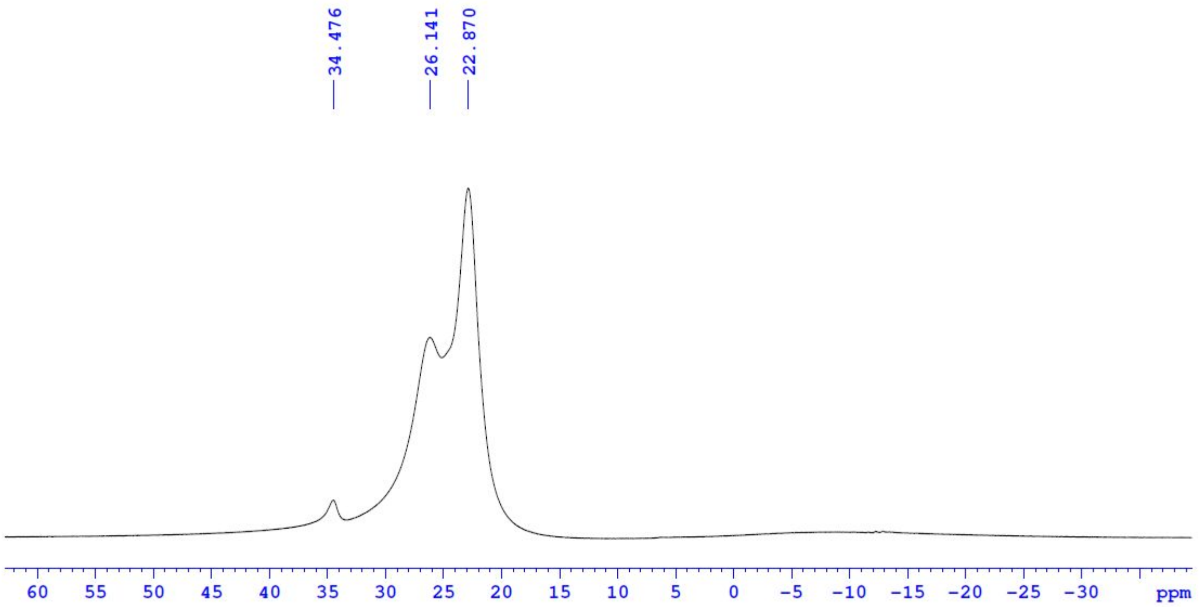

Figure S80. $^{11}B\{^{1}H\}$ NMR spectrum of **1m.**

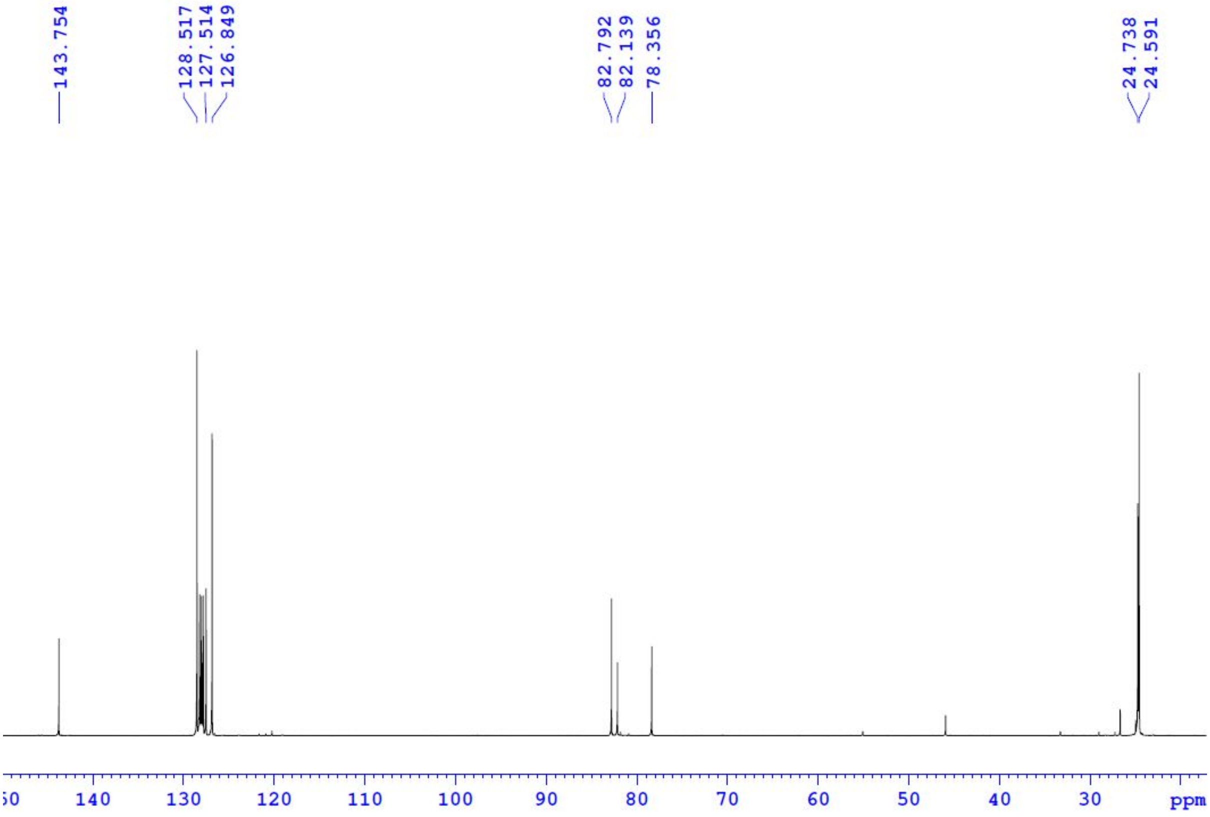

Figure S81. $^{13}C\{^{1}H\}$ NMR spectrum of **1m.**

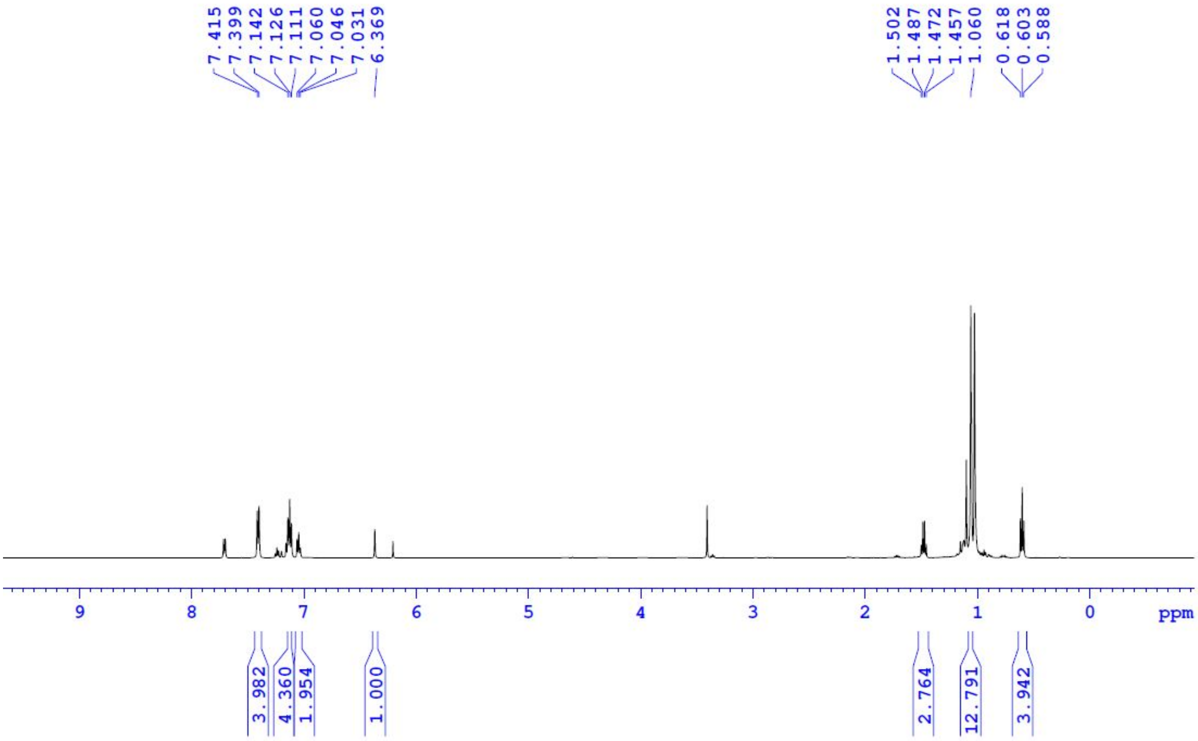

Figure S82. ^{1}H NMR spectrum of **1m-H1.**

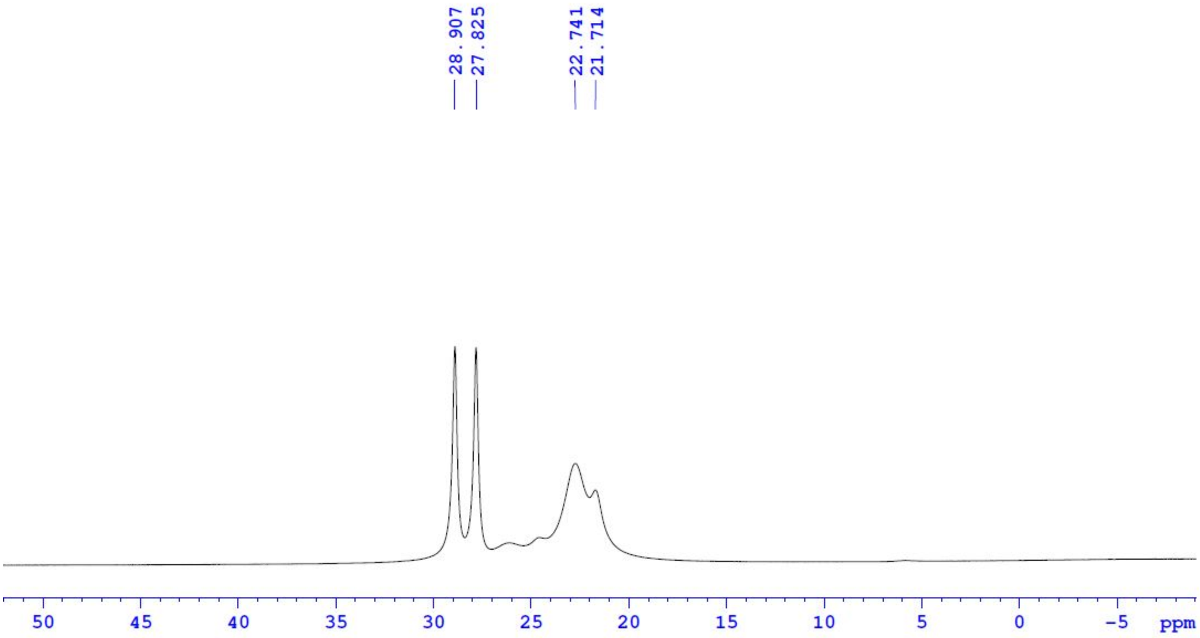

Figure S83. $^{11}B\{^{1}H\}$ NMR spectrum of **1m-H1.**

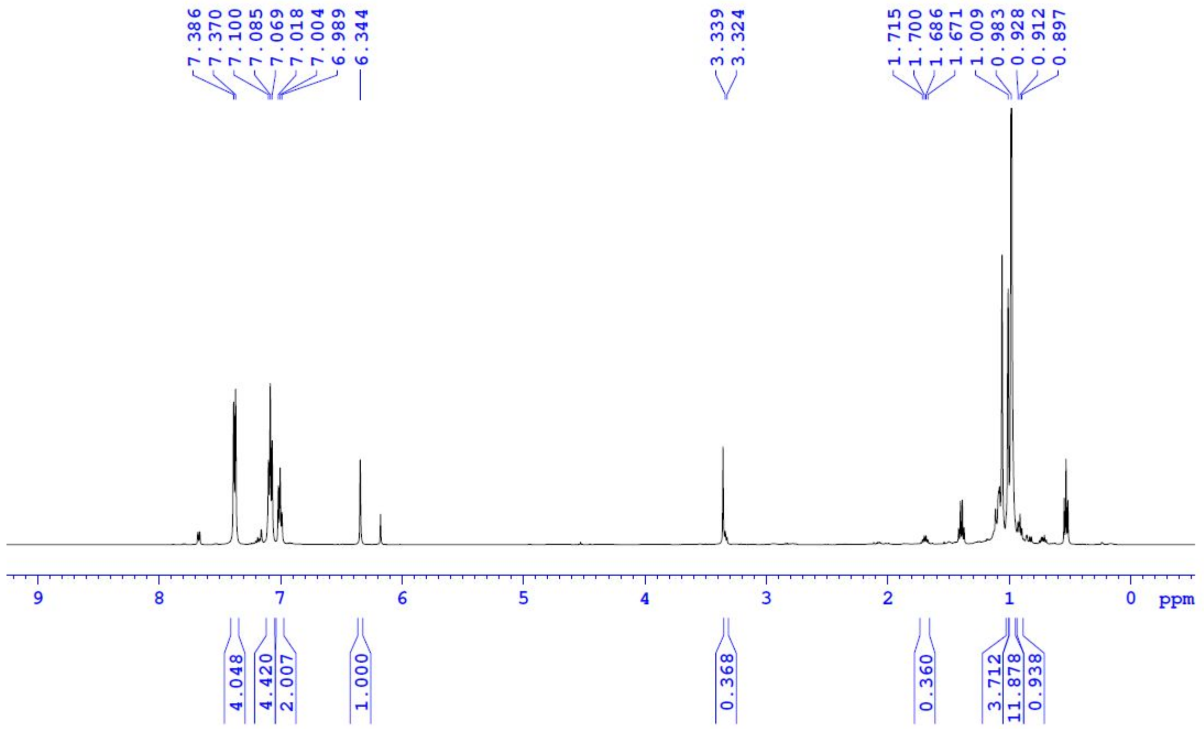

Figure S84. ^{1}H NMR spectrum of **1m-H3.**

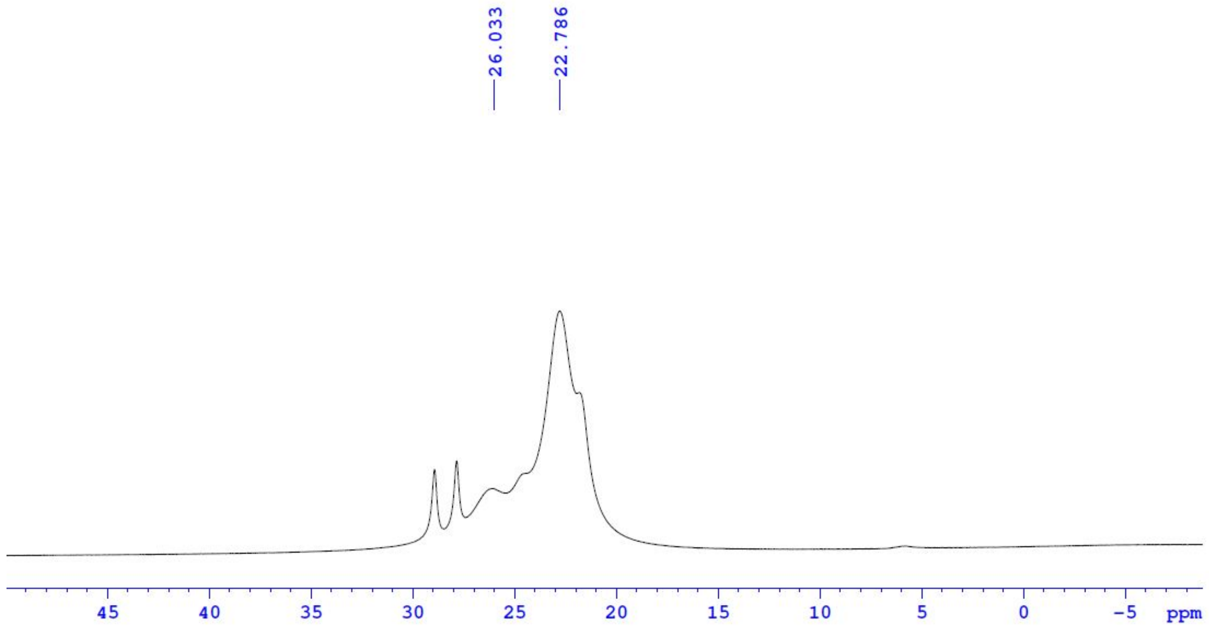

Figure S85. ^{11}B{^{1}H} NMR spectrum of **1m-H3.**

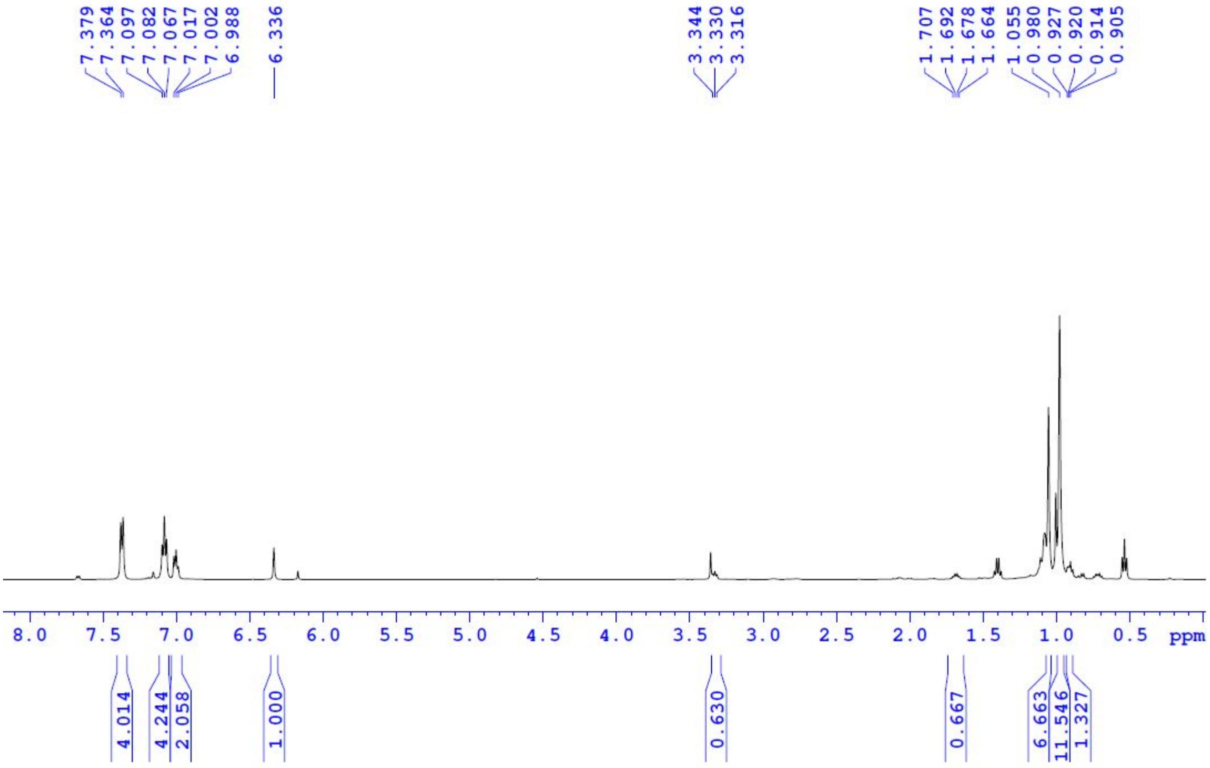

Figure S86. ^{1}H NMR spectrum of **1m-H5.**

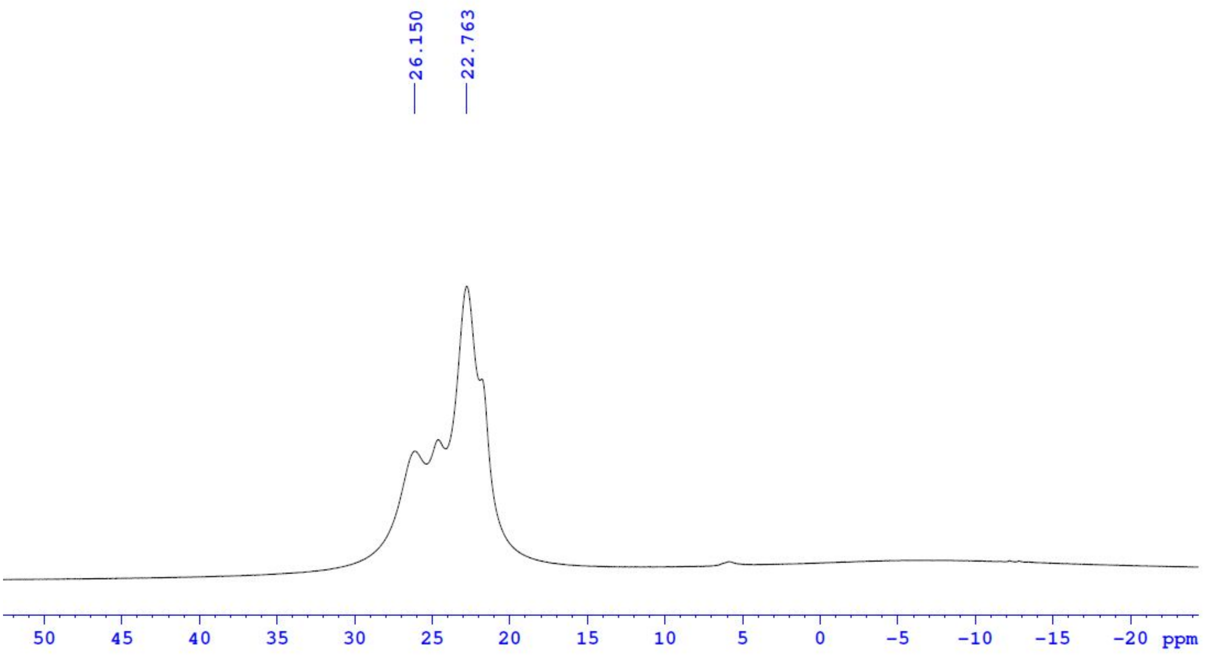

Figure S87. ^{11}B{^{1}H} NMR spectrum of **1m-H5.**

NMR spectra for hydroboration of carbodiimide

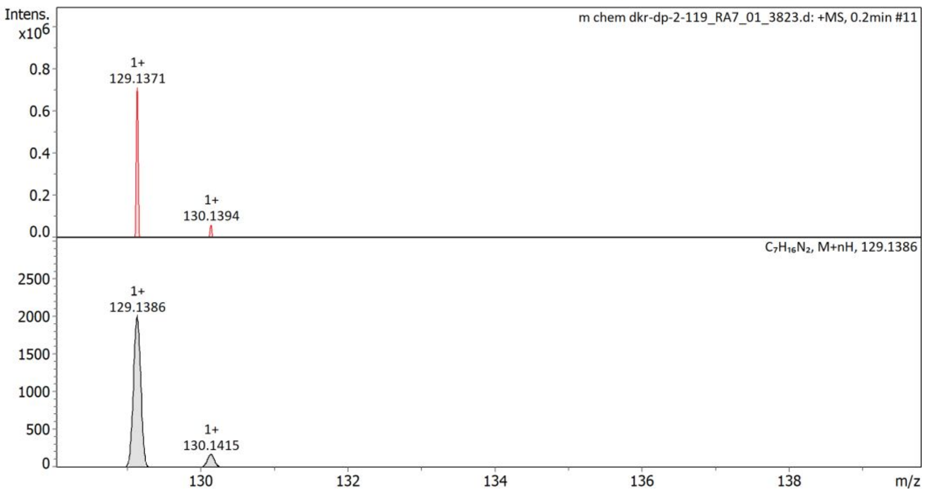

Figure S88. High-resolution mass spectrum of **2a**. Calculated isotope pattern for [M+H-Bpin]$^+$.

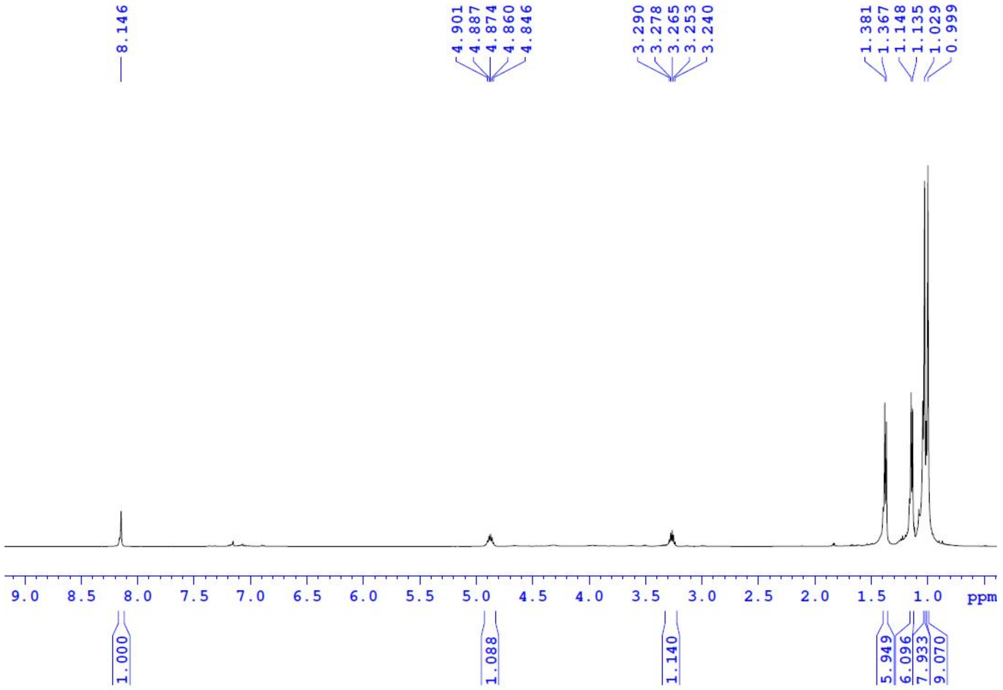

Figure S89. ^{1}H NMR spectrum of **2a.**

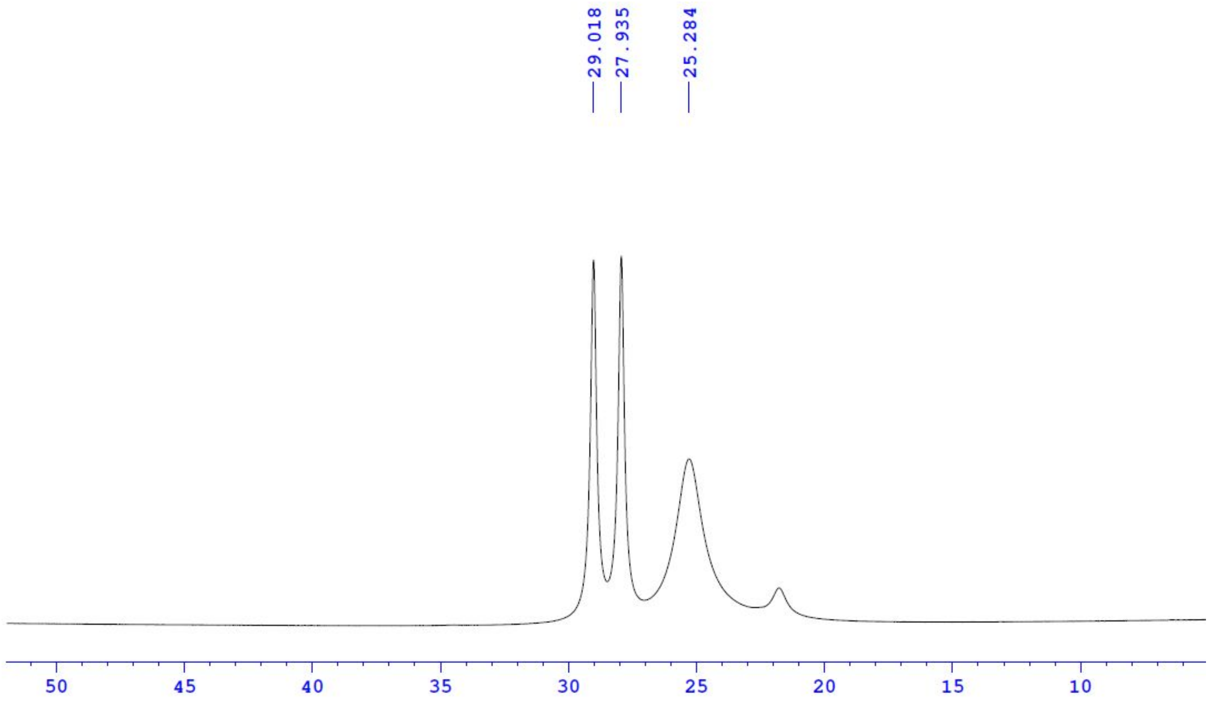

Figure S90. $^{11}B\{^1H\}$ NMR spectrum of **2a.**

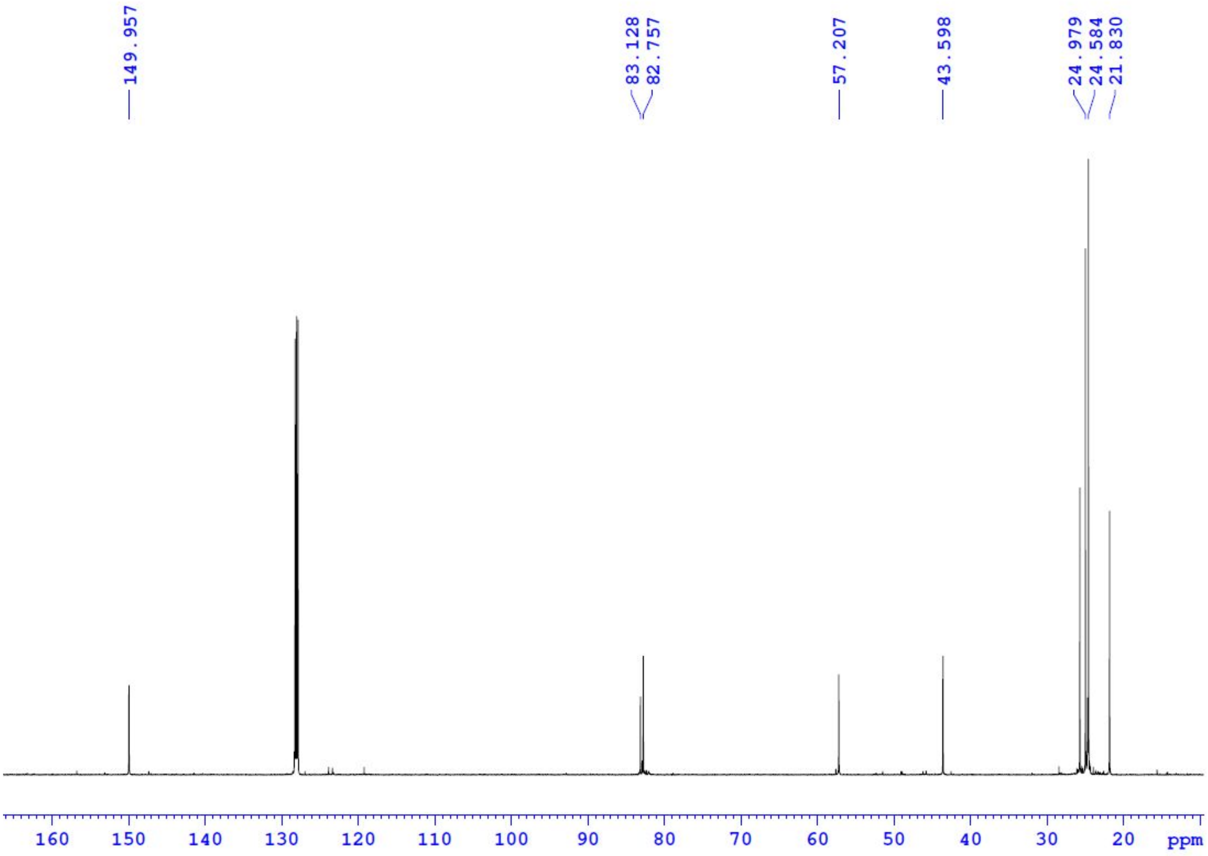

Figure S91. $^{13}C\{^1H\}$ NMR spectrum of **2a.**

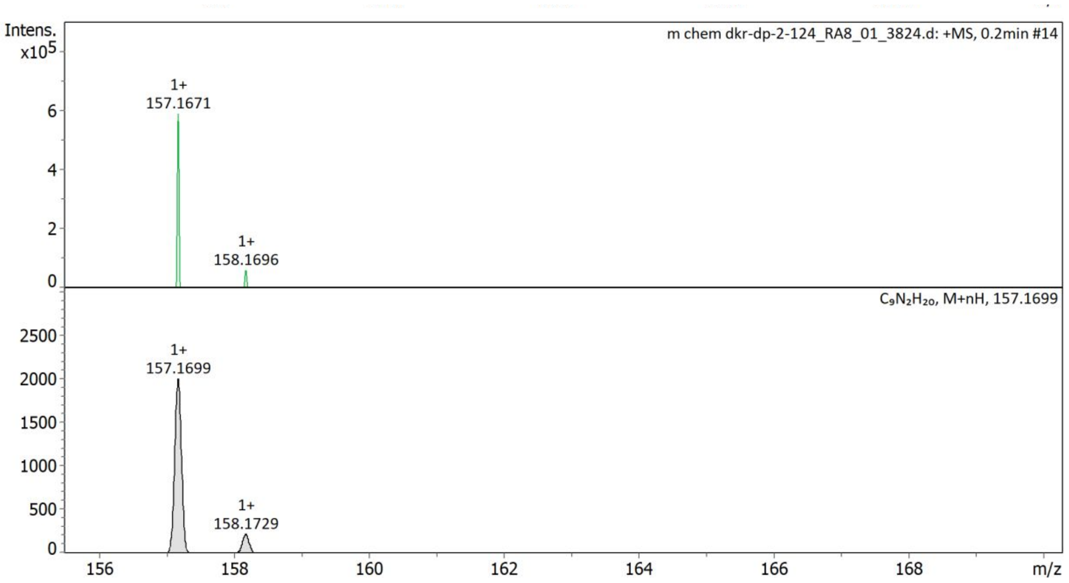

Figure S92. High-resolution mass spectrum of **2b**. Calculated isotope pattern for [M+H-Bpin]$^+$.

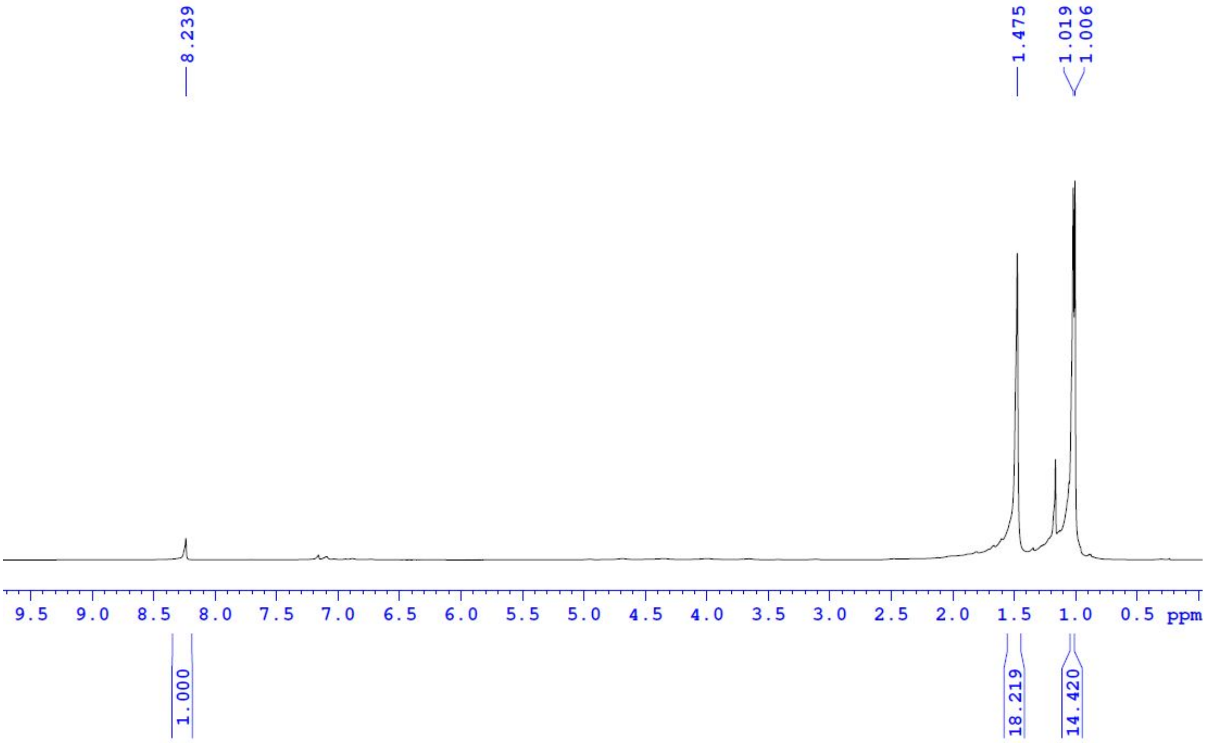

Figure S93. ^{1}H NMR spectrum of **2b.**

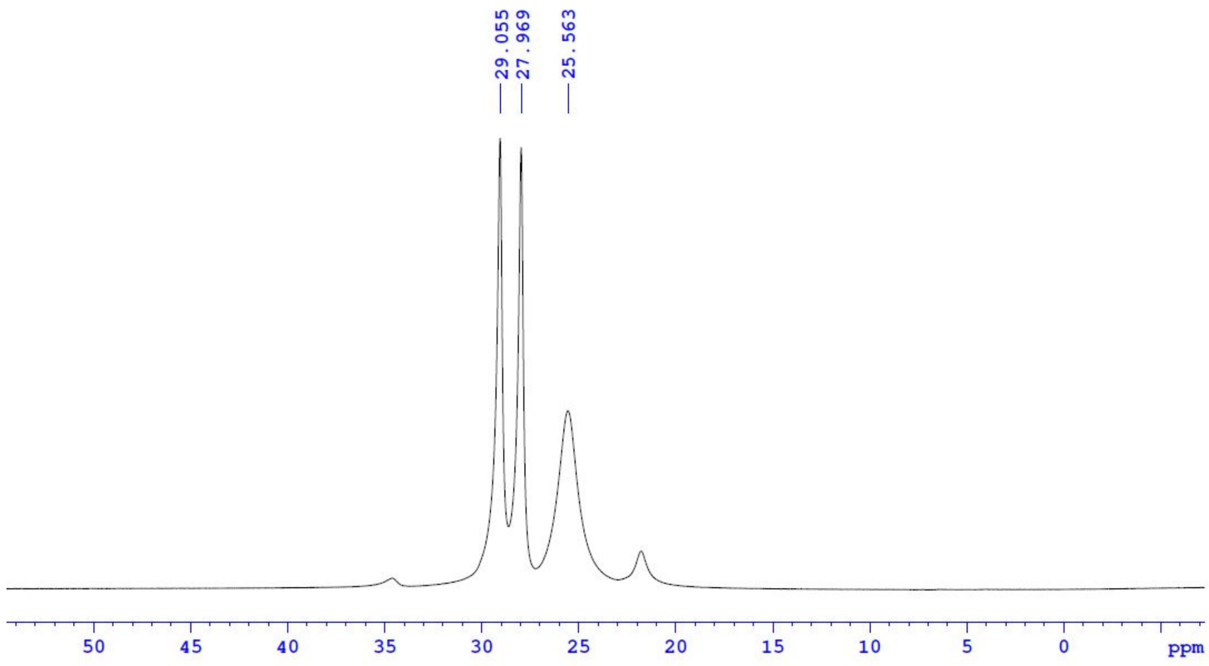

Figure S94. $^{11}B\{^{1}H\}$ NMR spectrum of **2b.**

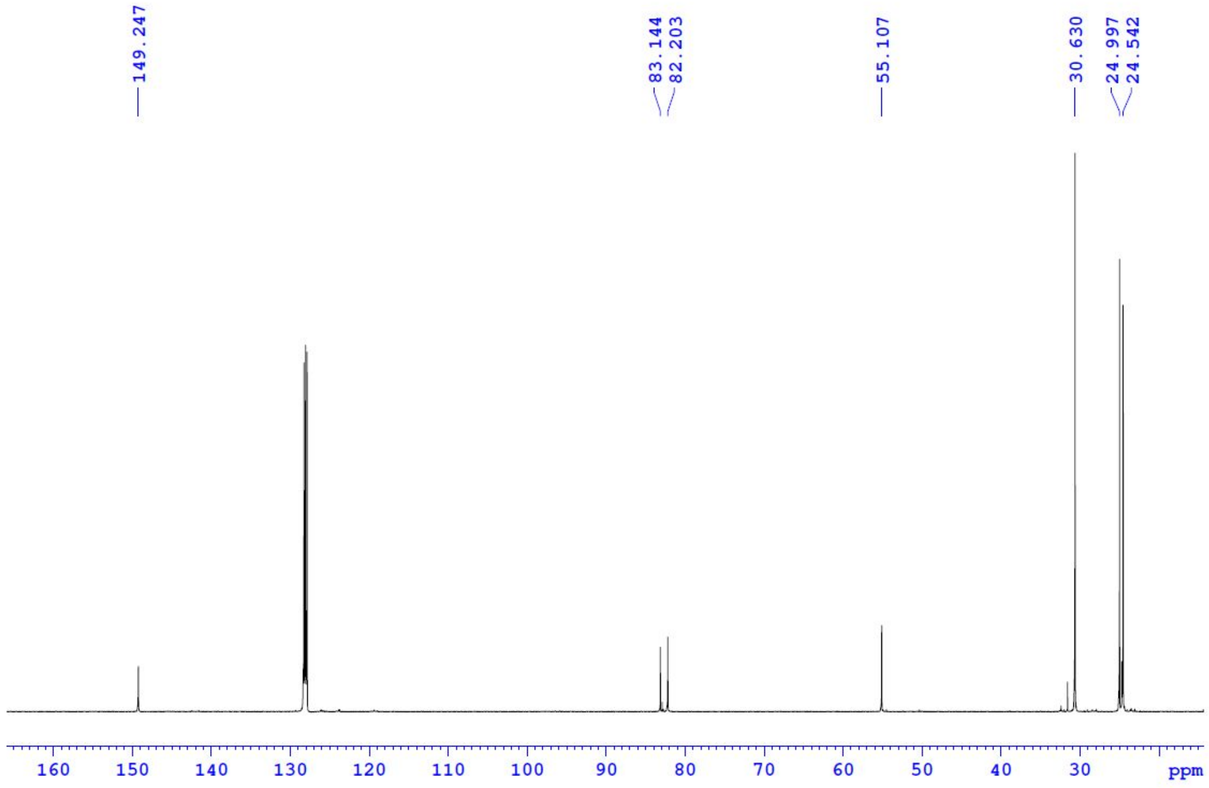

Figure S95. $^{13}C\{^{1}H\}$ NMR spectrum of **2b.**

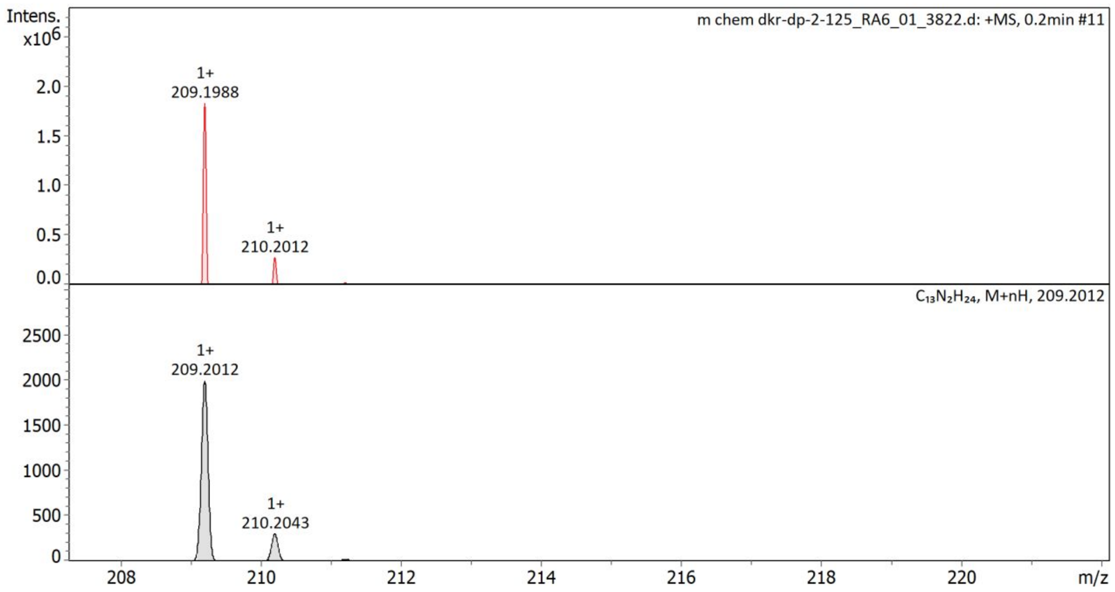

Figure S96. High-resolution mass spectrum of **2c**. Calculated isotope pattern for $[M+H-Bpin]^+$.

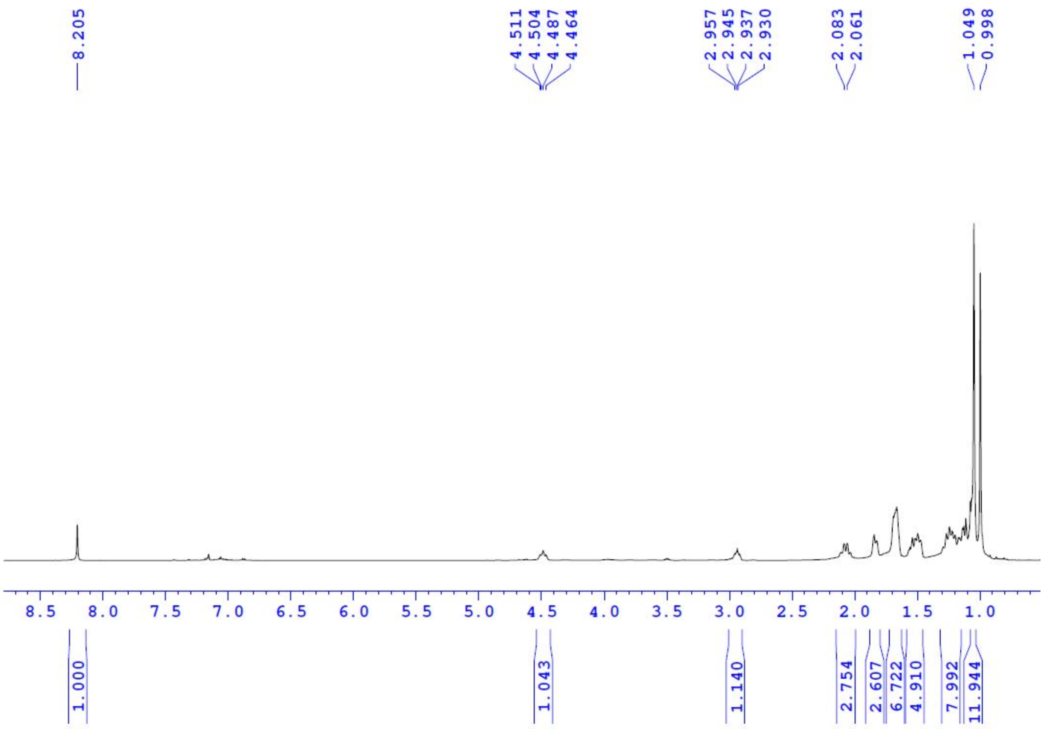

Figure S97. ^1H NMR spectrum of **2c.**

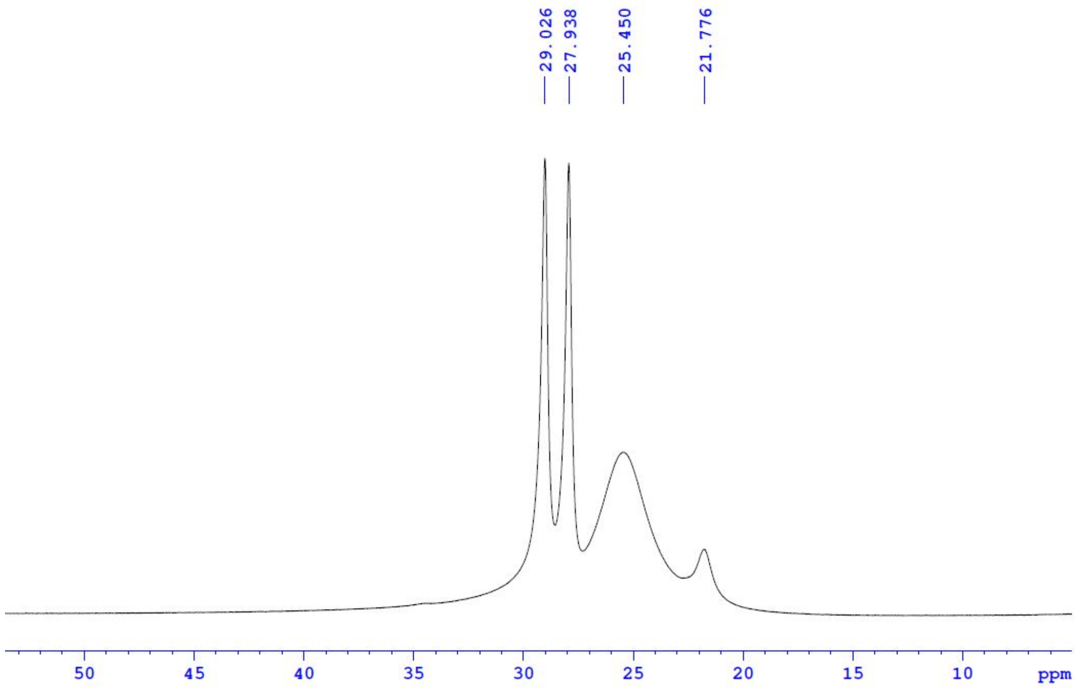

Figure S98. $^{11}B\{^1H\}$ NMR spectrum of **2c.**

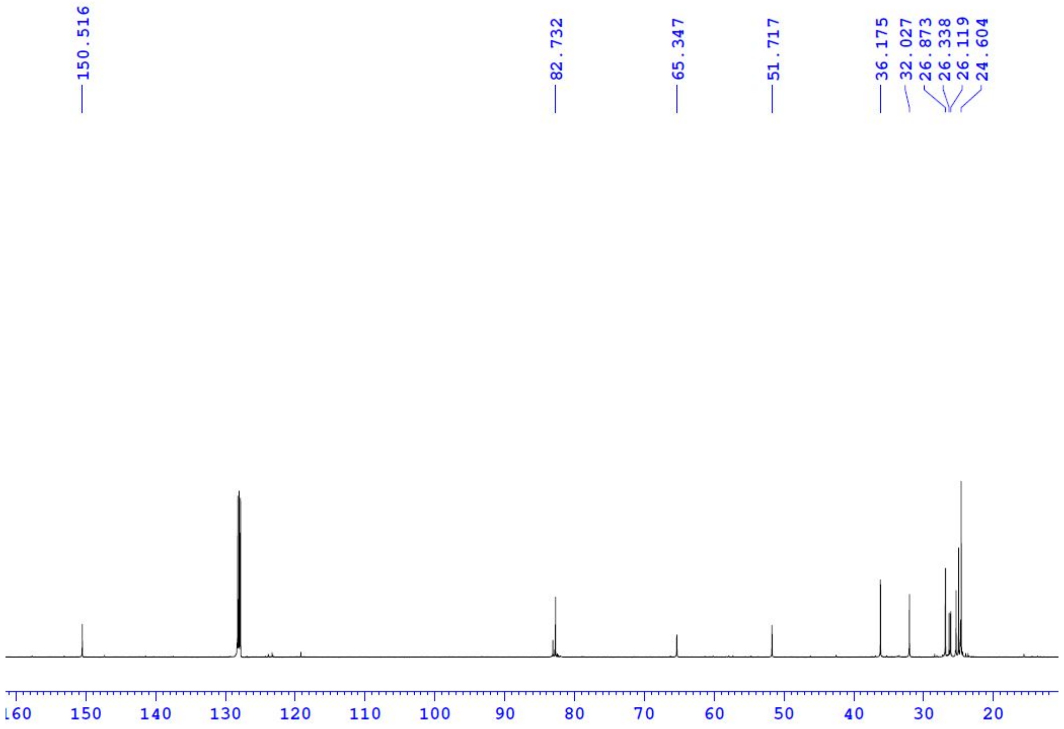

Figure S99. $^{13}C\{^1H\}$ NMR spectrum of **2c.**

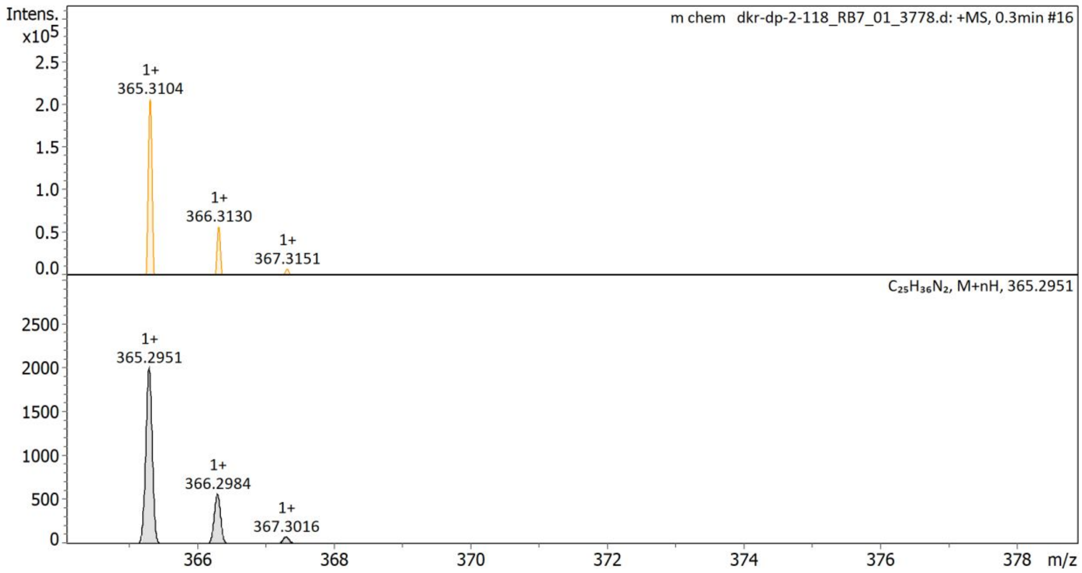

Figure S100. High-resolution mass spectrum of **2c**. Calculated isotope pattern for [M+H-Bpin]$^+$.

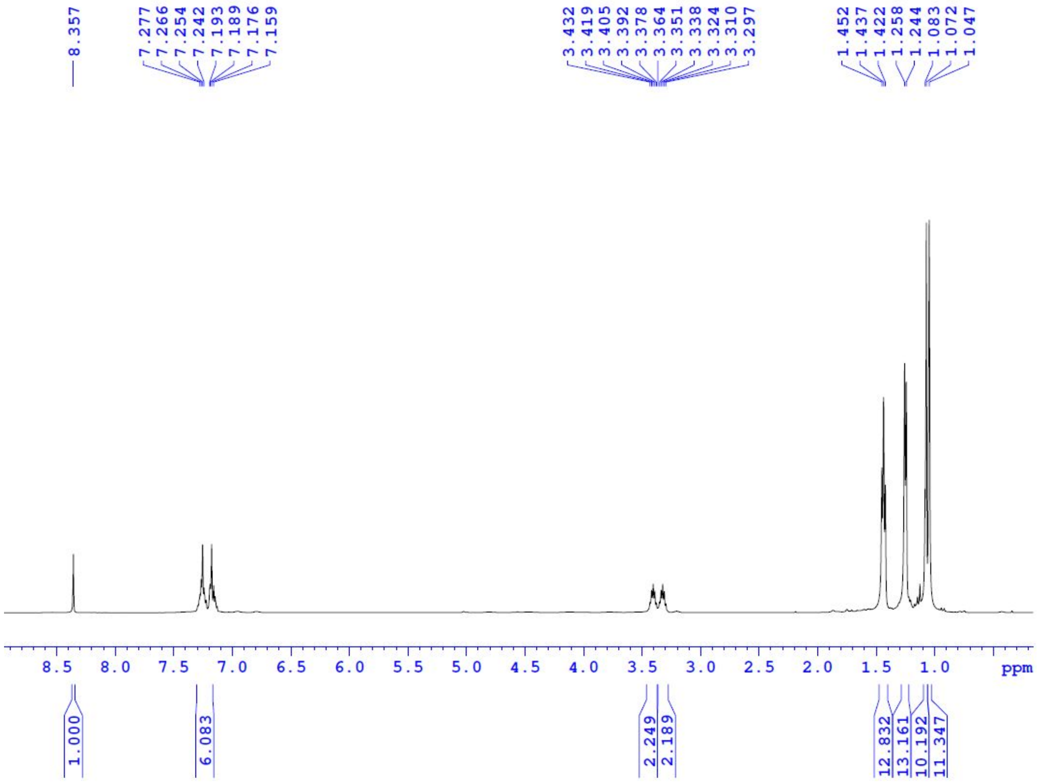

Figure S101. ^{1}H NMR spectrum of **2d.**

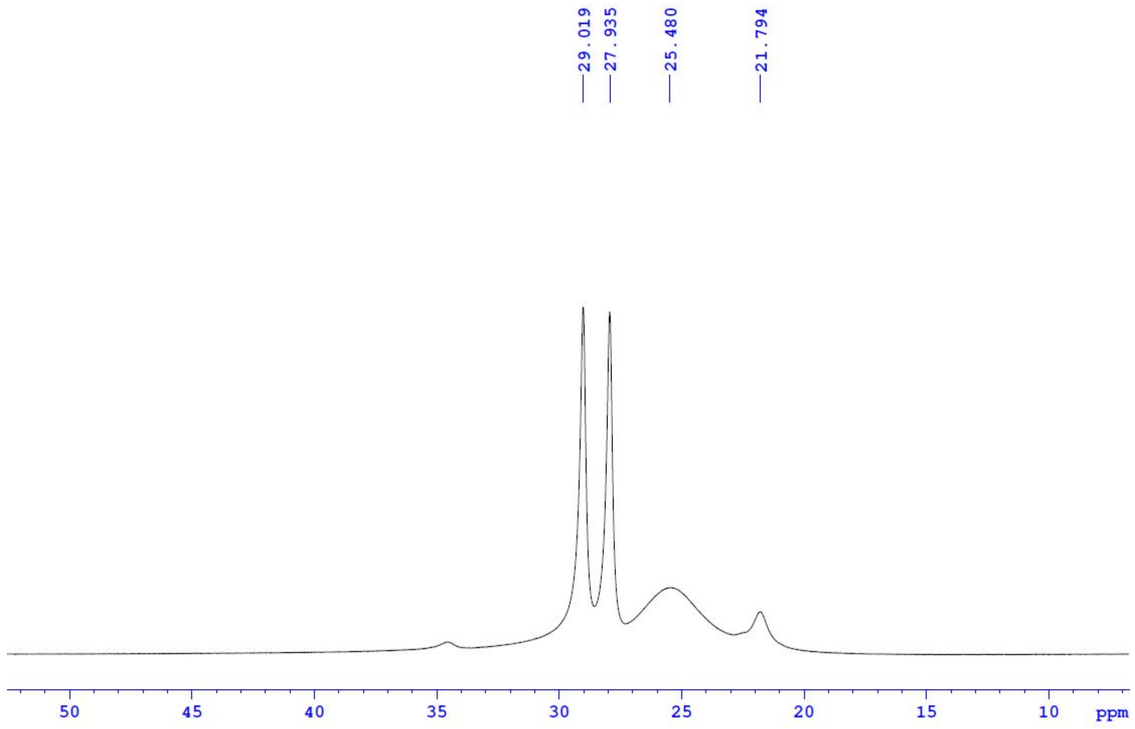

Figure S102. $^{11}B\{^{1}H\}$ NMR spectrum of **2d.**

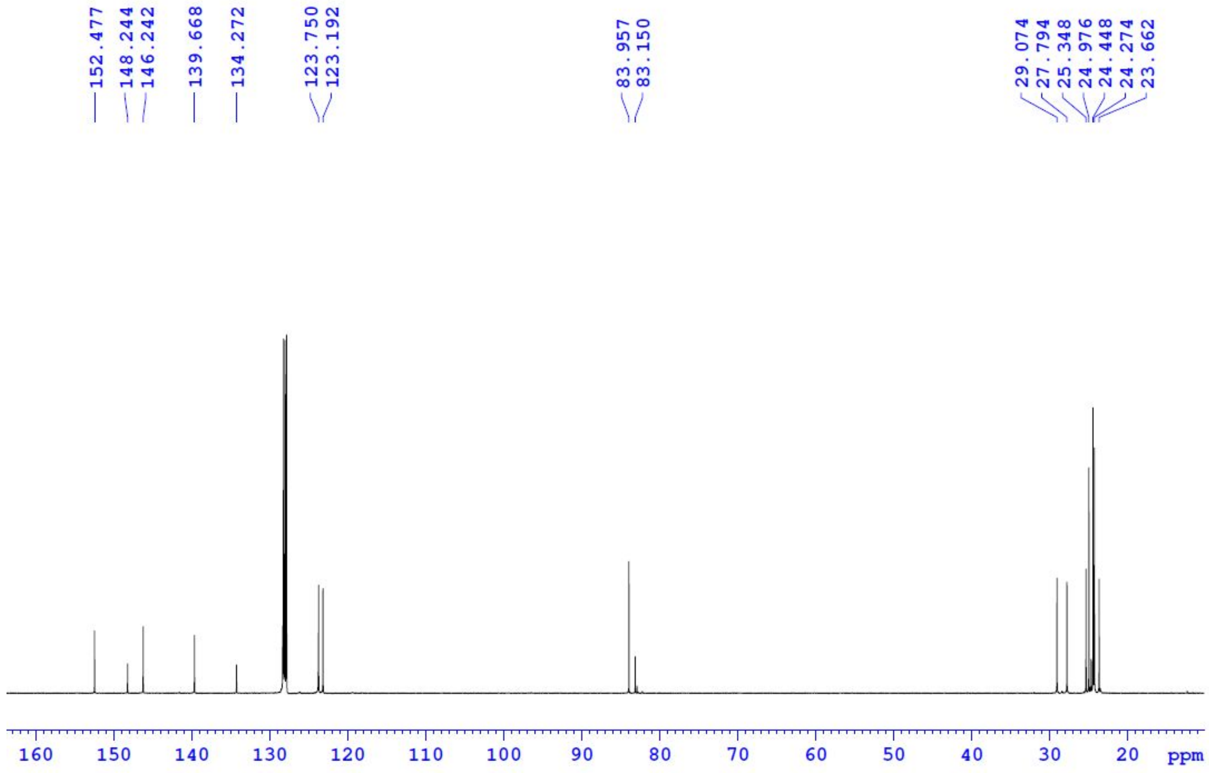

Figure S103. $^{13}C\{^{1}H\}$ NMR spectrum of **2d.**

NMR for gram scale reaction

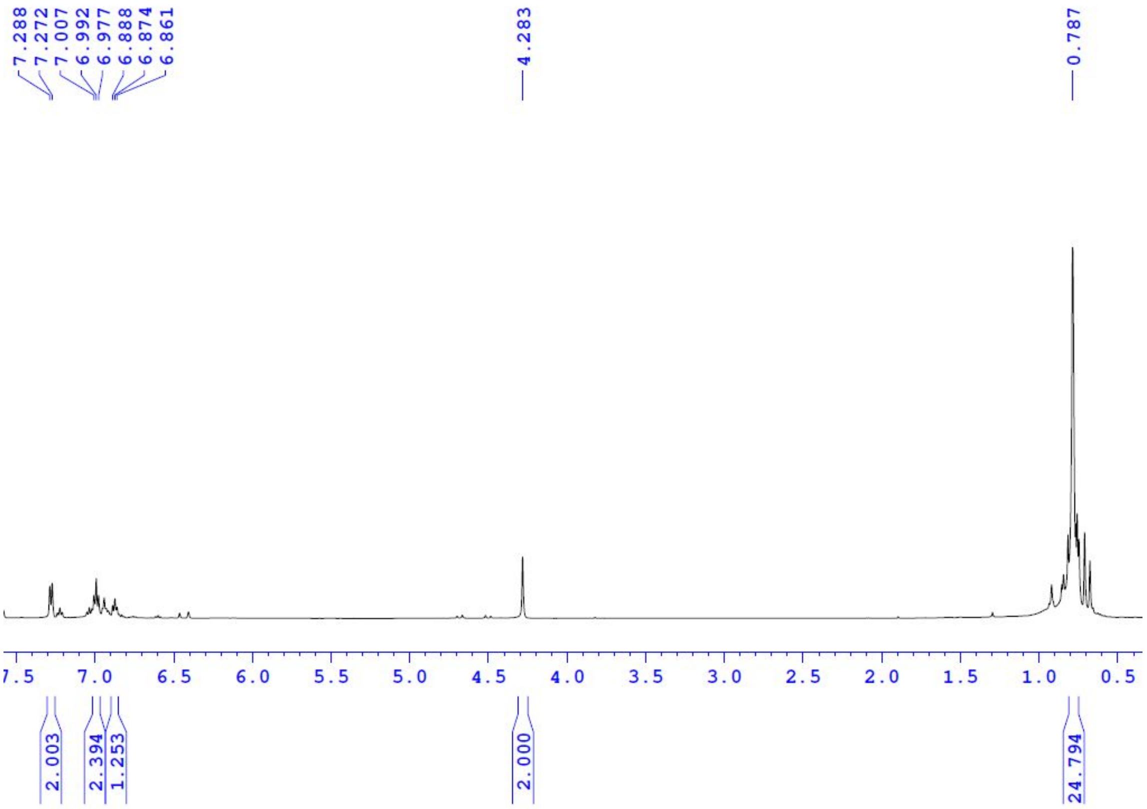

Figure S104. ^{1}H NMR spectrum of gram scale reaction of benzonitrile with HBpin.

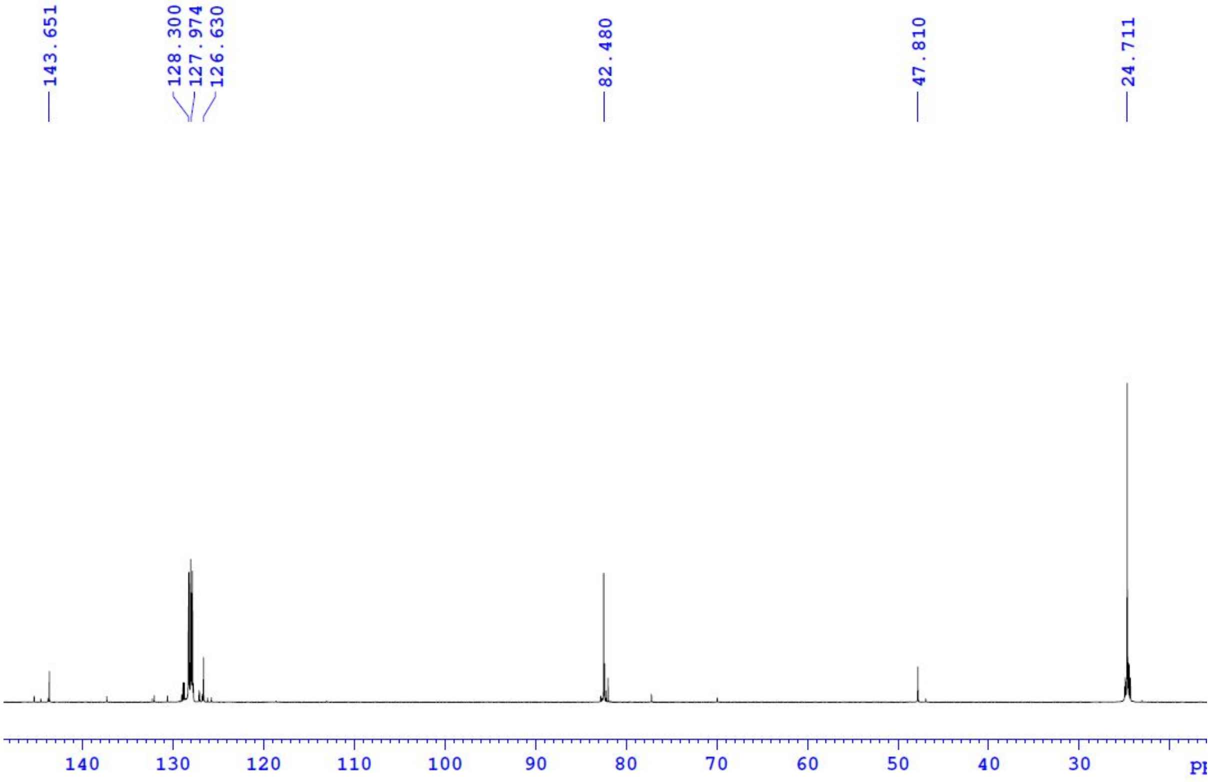

Figure S105. $^{13}C\{^{1}H\}$ NMR spectrum of gram scale reaction of benzonitrile with HBpin.

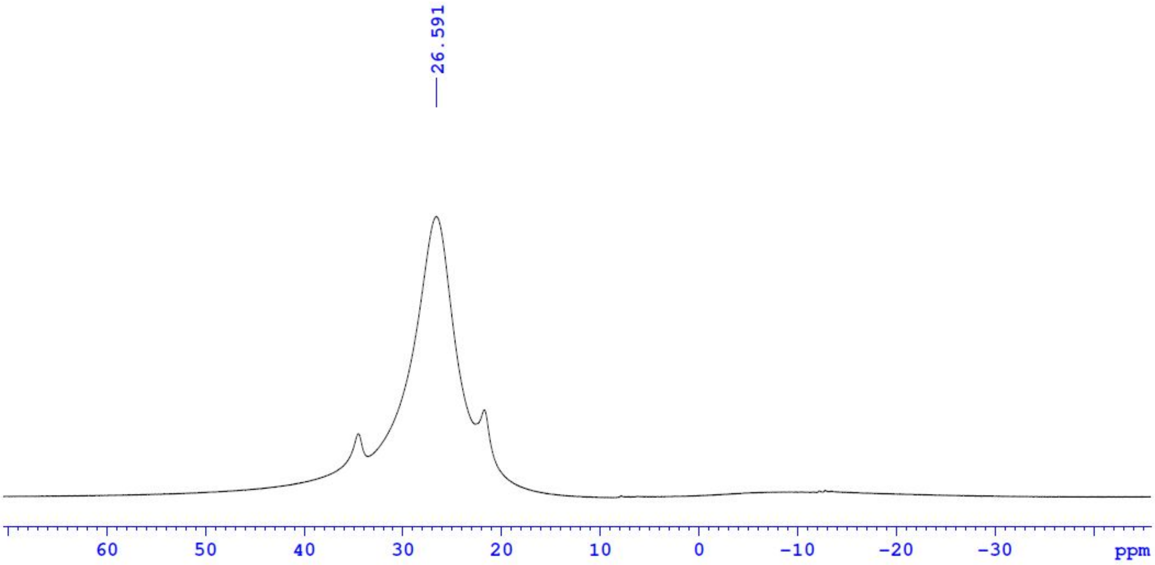

Figure S106. $^{11}B\{^{1}H\}$ NMR spectrum of gram scale reaction of benzonitrile with HBpin.

Crystallographic Details

Single crystal X-ray structural studies of crystals were collected on a CCDAgilent Technologies (Oxford Diffraction) SuperNova diffractometer. Data for both the compounds were collected at 293 K using a graphite-monochromated MoKα radiation (λα =0.71073 Å). The strategy for the data collection was evaluated by using the CrysAlisPro CCD software. The data were collected by the standard 'phi-omega scan techniques and were scaled and reduced using CrysAlisPro RED software. Using Olex2[2], the structure was solved with the SHELXT[3] structure solution program using Intrinsic Phasing and refined with the SHELXL[4] refinement package using Least Squares minimisation. The structures were solved by direct methods and refined by full-matrix least squares, based on *F*2, using SHELXL Crystal structures were refined using Olex2-1.0 software. All non-hydrogen atoms were refined anisotropically. All efforts to grow good quality crystals of **L1** to obtain better SCXRD data were not successful; however, the connectivity (Figure S107) and the spectroscopic data verified its identity. Therefore, we refrain from discussing its structural parameters.

Table S1. Crystallographic data of ligand **L3.**

**Chemical Compound**	**L3**
**CCDC**	2360759
**Empirical formula**	$C_{44}H_{56}N_4$
**Formula weight (g·mol⁻¹)**	640.92
**Temperature (K)**	293 (2)
**Radiation, λ (Å)**	MoK_α,<br>0.7107
**Crystal system**	Triclinic
**Space group**	P-1
Unit cell dimensions	
***a* (Å)**	9.8960(6)
***b* (Å)**	9.9489(8)
***c* (Å)**	11.4928(7)

**α (°)**	106.668(6)
**β (°)**	94.515(5)
**γ (°)**	113.850(7)
**Volume (Å^3)**	966.62(13)
Z	1
**Calculated density (g·cm^{-3})**	1.101
**Absorbtion coefficient (mm^{-1})**	0.064
***F*(000)**	348.0
**Theta range for collection**	6.612 to 49.986
**Reflections collected**	7083
**Independent reflections**	3376
**Refinement method**	Full-matrix least-squares on F^2
**Data /restraints/parameters**	3376/0/223
**Goodness-of-fit on F^2**	1.049
**Final R indices [I>2 σ (I)]**	R_1 = 0.0643, wR^2 = 0.1805
**R indices (all data)**	R_1 = 0.0800, wR^2 = 0.1904
**Maximum/minimum residual electron density (e·Å^{-3})**	0.31 / –0.22
**Index ranges**	-11 ≤ h ≤ 10, -11 ≤ k ≤ 11, -13 ≤ l ≤ 13

Molecular Diagram of Ligand L1

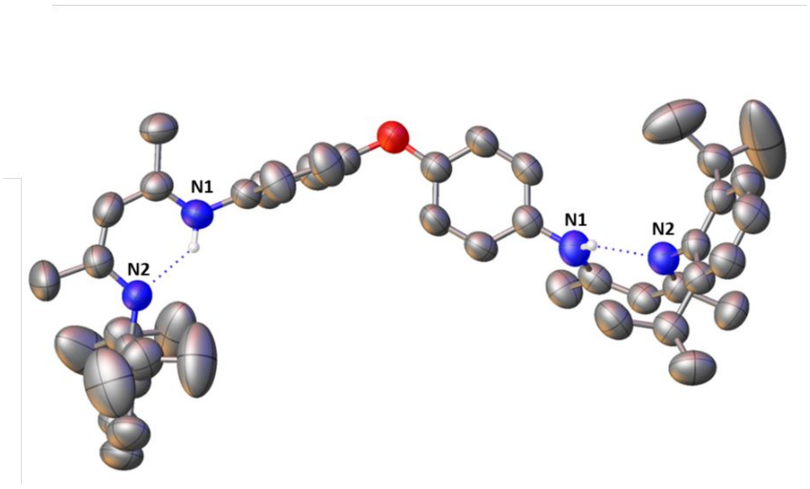

Figure S107. The molecular structure of ligand **L1**.

Computational Details

All DFT calculations were conducted using the Gaussian 16, Revision C.1 software.[5] Geometry optimizations were performed at the PBE0[6,7] level of theory, incorporating Grimme's D3 dispersion corrections[8] with Becke-Johnson (BJ) damping function.[9] The def2-SVP[10] basis set was employed for all atoms. Harmonic vibrational frequency calculations were carried out at the same level of theory to confirm whether the optimized structures were energy minima (indicated by only real frequencies) or transition states (indicated by a single imaginary frequency). These calculations also provided Gibbs free energy corrections at room temperature and 1 atm. Single-point energy calculations were executed using the PBE0-D3(BJ) functional and the def2-TZVPP[10] basis set, offering triple-zeta quality electronic energies for free energy calculations. Solvent effects were included in the single-point energy calculations using the solvation model based on density (SMD)[11] variation of the integral equation formalism of the Polarizable Continuum Model (IEFPCM).[12] The chosen solvent is benzene, with a dielectric constant of $\varepsilon = 2.2706$. Thus, the level of theory for geometry optimizations is PBE0-D3(BJ)/def2-TZVPP+SMD(Benzene). Additionally, a concentration correction of $\Delta G^{0\rightarrow *} = RT \ln(24.46) = 1.89$ kcal mol^{-1} was applied to the free energies of all calculated species, adjusting the 1 atm gas-phase values (ΔG^0) to a condensed phase standard state concentration of 1 M (ΔG^*). This adjustment ensures a more accurate depiction of associative and dissociative steps.[13-15] The free energies of all computed species throughout the mechanism are presented in Tables S2 and S3. To confirm the connectivity of the transition state structures, intrinsic reaction coordinate (IRC) calculations were performed.[16] Charge analyses using Hirshfeld,[17] Mulliken,[18,19] and Löwdin[20] population methods were conducted with the Multiwfn 3.8 program.[21] Visualizations of the 3D structures were generated using CYLview.[22] Additional calculations for **TS3** and intermediate **F**, incorporating Dipp substituents (**TS3$^{\mathbf{MDipp}}$**

and F^{MDipp}), were conducted at the ωB97X-D[23]/def2-TZVPP and DLPNO-CCSD(T)[24]/def2-TZVPP levels of theory.

Table S2. Computed free energies (G_{tot}) of the species involved in the proposed mechanistic pathway of the hydroboration of PhCN to **1d** using $\mathbf{1^{MMe}}$ as the pre-catalyst (all values are given in Hartree, Eh). G_{tot} was obtained as the sum of the electronic energy (E) at the PBE0-D3(BJ)/def2 TZVPP+SMD(Benzene) level; the thermal correction to Gibbs free energy (G_{corr}) at the PBE0-D3(BJ)/def2-SVP level; and the concentration correction (G_{conc} = 0.003012 Eh).

Species	E (Eh)	G_{corr} (Eh)	G_{tot} (Eh)
PhCN	-324.239377	0.069889	-324.166476
HBpin	-254.417479	0.050916	-254.363551
EtBpin	-332.997990	0.102659	-332.892319
**1d**	-833.207281	0.223998	-832.980271
$\mathbf{1^{MMe}}$	-2242.117908	0.214477	-2241.900419
$\mathbf{A^{MMe}}$	-2163.556625	0.162233	-2163.391381
$\mathbf{C^{MMe}}$	-2487.802452	0.249180	-2487.550260
$\mathbf{TS1^{MMe}}$	-2487.768383	0.249917	-2487.515454
$\mathbf{D^{MMe}}$	-2487.825783	0.255452	-2487.567319
$\mathbf{TS2^{MMe}}$	-2742.264577	0.330354	-2741.931211
$\mathbf{E^{MMe}}$	-2742.288144	0.332216	-2741.952916
$\mathbf{F^{MMe}}$	-2742.288012	0.333282	-2741.951718
$\mathbf{TS3^{MMe}}$	-2742.212841	0.332839	-2741.876990
$\mathbf{G^{MMe}}$	-2742.326249	0.334415	-2741.988823
$\mathbf{TS4^{MMe}}$	-2996.760197	0.410869	-2996.346316
$\mathbf{H^{MMe}}$	-2996.775848	0.409085	-2996.363751

Table S3. Computed free energies (G_{tot}) of the species involved in the proposed mechanistic pathway of the hydroboration of PhCN to **1d** using $\mathbf{1^{MPh}}$ as the pre-catalyst (all values are given in Hartree, Eh). Species already mentioned in Table S2 are not shown here. The monomeric systems investigated with Dipp substituents, $\mathbf{F^{MDipp}}$ and $\mathbf{TS3^{MDipp}}$, are also shown. G_{tot} was obtained as the sum of the electronic energy (E) at the PBE0-D3(BJ)/def2 TZVPP+SMD(Benzene) level; the thermal correction to Gibbs free energy (G_{corr}) at the PBE0-D3(BJ)/def2-SVP level; and the concentration correction (G_{conc} = 0.003012 Eh).

Species	E (Eh)	G_{corr} (Eh)	G_{tot} (Eh)
$\mathbf{1^{MPh}}$	-2625.318408	0.309358	-2625.006039
$\mathbf{A^{MPh}}$	-2546.754599	0.255590	-2546.495997
$\mathbf{C^{MPh}}$	-2870.994715	0.346418	-2870.645285
$\mathbf{TS1^{MPh}}$	-2870.969619	0.346820	-2870.619787
$\mathbf{D^{MPh}}$	-2871.023662	0.350503	-2870.670147
$\mathbf{TS2^{MPh}}$	-3125.466810	0.428239	-3125.035559
$\mathbf{E^{MPh}}$	-3125.479151	0.430943	-3125.045196
$\mathbf{F^{MPh}}$	-3125.488417	0.432318	-3125.053087
$\mathbf{TS3^{MPh}}$	-3125.409849	0.430718	-3124.976120
$\mathbf{G^{MPh}}$	-3125.516478	0.431843	-3125.081624
$\mathbf{TS4^{MPh}}$	-3379.956166	0.508392	-3379.444762
$\mathbf{H^{MPh}}$	-3379.977600	0.506799	-3379.467790
$\mathbf{F^{MDipp}}$	-3596.876427	0.753156	-3596.120259
$\mathbf{TS3^{MDipp}}$	-3596.811880	0.752371	-3596.056497

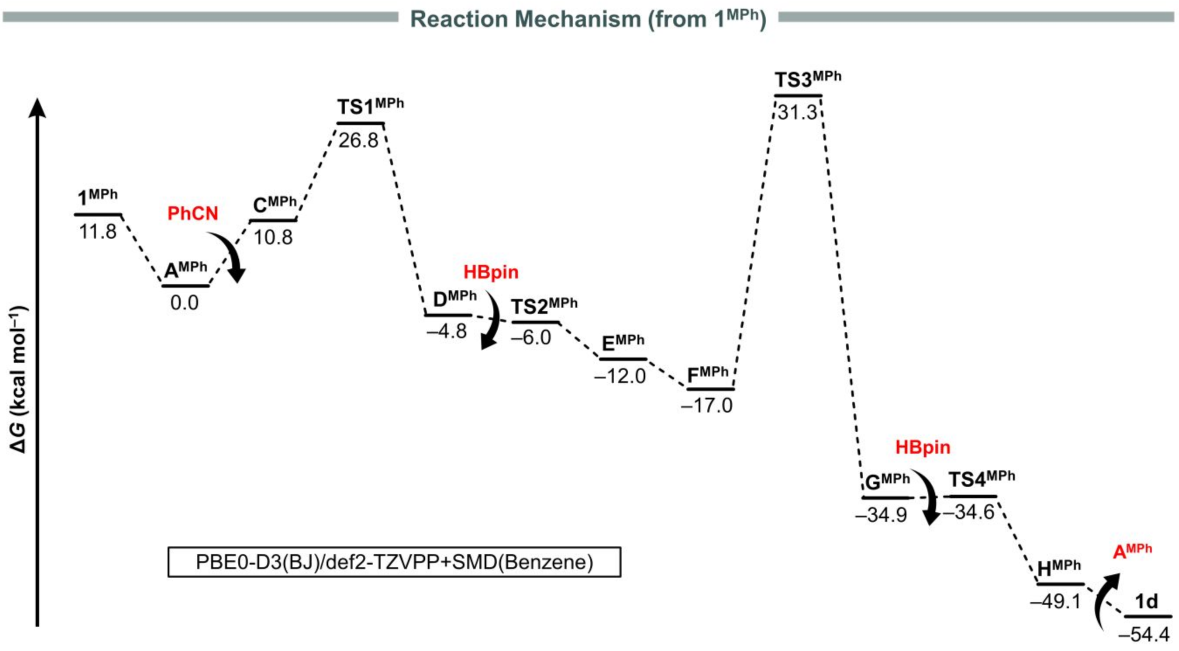

Figure S108. Computed Gibbs free energies in kcal mol^{-1} for the hydroboration of PhCN to **1d** using **1**$^{\mathbf{MPh}}$ as the pre-catalyst. Energies are at the PBE0-D3(BJ)/def2-TZVPP+SMD(Benzene) level of theory, from optimized structures at PBE0-D3(BJ)/def2-SVP.

Cartesian Coordinates

All cartesian coordinates are given in Å.

**PhCN**			
N	-3.201953000	0.000171000	0.000031000
C	-2.042514000	-0.000335000	-0.000016000
C	-0.609702000	-0.000580000	-0.000031000
C	0.091236000	-1.215782000	-0.000010000
C	1.481899000	-1.208725000	0.000003000
C	2.177030000	0.000504000	0.000021000
C	1.481037000	1.209302000	-0.000004000
C	0.090420000	1.215280000	-0.000002000
H	-0.462220000	-2.156467000	-0.000003000
H	2.027681000	-2.154681000	-0.000011000
H	3.269479000	0.000861000	0.000049000
H	2.026167000	2.155633000	-0.000011000
H	-0.463870000	2.155473000	-0.000007000

**HBpin**			
B	1.223751000	0.000050000	0.000001000
O	0.480069000	-1.139087000	-0.000104000
C	-0.891102000	-0.771896000	0.000111000
C	-0.891175000	0.771825000	-0.000111000
O	0.479990000	1.139126000	0.000105000
H	-1.382044000	-1.195480000	0.890693000
H	-1.382421000	-1.195754000	-0.890126000
H	-1.382511000	1.195656000	0.890127000
H	-1.382126000	1.195378000	-0.890700000
H	2.423535000	0.000062000	-0.000005000

EtBpin

B	0.199248000	-0.289374000	-0.001973000
O	-0.233177000	1.008656000	-0.043848000
C	-1.646762000	1.016032000	0.039489000
C	-2.054474000	-0.469357000	-0.037769000
O	-0.835921000	-1.185202000	0.045085000
H	-2.065499000	1.615768000	-0.783813000
H	-1.953212000	1.486450000	0.988804000
H	-2.559506000	-0.715747000	-0.986961000
H	-2.721145000	-0.770380000	0.785628000
C	1.707170000	-0.708376000	-0.010753000
H	1.868887000	-1.386765000	0.846220000
C	2.701137000	0.446104000	0.009440000
H	1.865675000	-1.349143000	-0.897321000
H	2.574507000	1.068815000	0.908375000
H	2.560507000	1.110521000	-0.856833000
H	3.743903000	0.093296000	-0.006570000

1d

N	0.676768000	-0.142089000	0.635417000
C	-0.482537000	0.219050000	1.459450000
C	-1.715965000	0.499877000	0.635216000
C	-1.901358000	1.754307000	0.041798000
C	-3.018339000	2.005335000	-0.751893000
C	-3.966918000	1.004784000	-0.963570000
C	-3.790706000	-0.247279000	-0.377043000
C	-2.671936000	-0.497374000	0.416435000
H	-1.152412000	2.533268000	0.206536000
H	-3.152507000	2.990474000	-1.206002000

H	-4.844637000	1.202341000	-1.583943000
H	-4.531109000	-1.035284000	-0.536964000
H	-2.523559000	-1.481169000	0.868174000
H	-0.224670000	1.109124000	2.049391000
H	-0.689122000	-0.601724000	2.160127000
B	1.561948000	0.897236000	0.232917000
O	1.356546000	2.209838000	0.605947000
C	2.478125000	2.954439000	0.171833000
C	3.206344000	2.017610000	-0.806550000
O	2.688845000	0.733358000	-0.527151000
H	3.106113000	3.212563000	1.042483000
H	2.149110000	3.893522000	-0.299217000
H	4.298700000	2.023688000	-0.668789000
H	2.996241000	2.277705000	-1.859002000
B	0.781513000	-1.498848000	0.213506000
O	-0.074691000	-2.469122000	0.689630000
C	0.190325000	-3.655561000	-0.032873000
C	1.542442000	-3.393805000	-0.716321000
O	1.698365000	-1.990059000	-0.676218000
H	1.569275000	-3.747186000	-1.758664000
H	2.376989000	-3.872706000	-0.174248000
H	-0.617185000	-3.823983000	-0.766937000
H	0.214710000	-4.518363000	0.650460000

1

C	-8.046652000	-0.861393000	-0.380672000
C	-6.284979000	-2.658823000	-0.070284000
N	-7.270848000	0.199002000	-0.192934000
Zn	-5.390604000	0.113241000	0.415930000

C	-7.776932000	1.511460000	-0.368747000
C	-3.983982000	1.362700000	1.008886000
C	-2.595113000	1.030374000	0.463805000
C	-9.506499000	-0.652720000	-0.684310000
C	-6.052151000	-4.143915000	-0.142703000
H	-6.988864000	-4.689224000	-0.307613000
H	-5.581183000	-4.506606000	0.783410000
H	-5.354408000	-4.385917000	-0.958777000
H	-10.021003000	-0.244069000	0.199653000
H	-9.994028000	-1.593267000	-0.967078000
H	-9.642706000	0.082369000	-1.489436000
C	-7.590150000	-2.191266000	-0.309908000
H	-8.341353000	-2.958523000	-0.492813000
H	-2.262437000	0.028781000	0.783237000
H	-1.822177000	1.748012000	0.795200000
H	-2.571435000	1.028397000	-0.639440000
H	-3.953363000	1.373806000	2.113357000
H	-4.272635000	2.388534000	0.718128000
N	-5.263491000	-1.862590000	0.214953000
C	-3.955361000	-2.367819000	0.382147000
C	-3.432546000	-2.578923000	1.662429000
C	-3.117998000	-2.551509000	-0.726372000
C	-1.790515000	-2.928102000	-0.562071000
C	-1.279236000	-3.115677000	0.723588000
C	-2.101143000	-2.947580000	1.835213000
O	0.016318000	-3.503888000	0.922194000
C	1.029489000	-2.743202000	0.413298000
C	2.248888000	-3.381426000	0.183574000
C	3.334361000	-2.653899000	-0.289247000

C	3.223485000	-1.282453000	-0.552790000
C	1.998033000	-0.654663000	-0.299335000
C	0.905057000	-1.372091000	0.176472000
N	4.364822000	-0.553168000	-0.949357000
Zn	5.746631000	-0.400877000	0.474248000
N	6.992685000	0.679520000	-0.618778000
C	6.705526000	1.152514000	-1.825046000
C	5.534071000	0.831975000	-2.536350000
C	4.444851000	0.034942000	-2.135686000
C	5.658492000	-1.112409000	2.310559000
C	4.346554000	-0.814776000	3.034488000
H	-4.078052000	-2.421925000	2.529448000
H	-1.678018000	-3.097860000	2.829992000
H	-3.519169000	-2.373147000	-1.726924000
H	-1.136161000	-3.060666000	-1.426179000
H	4.293270000	-3.148384000	-0.460571000
H	2.328483000	-4.450546000	0.388168000
H	1.909224000	0.423640000	-0.449477000
H	-0.038485000	-0.862289000	0.379987000
H	4.311957000	-1.244454000	4.052250000
H	3.476712000	-1.216335000	2.488411000
H	4.172979000	0.269175000	3.146207000
H	6.515105000	-0.732186000	2.894960000
H	5.808066000	-2.206278000	2.252414000
H	5.458240000	1.264190000	-3.533532000
C	3.332216000	-0.148876000	-3.135492000
C	7.670291000	2.100898000	-2.486468000
H	8.702611000	1.731147000	-2.422418000
H	7.654788000	3.072170000	-1.966735000

H	7.408780000	2.267141000	-3.538380000
H	3.660521000	0.138340000	-4.141858000
H	2.457370000	0.465382000	-2.873520000
H	2.990893000	-1.194348000	-3.149028000
C	8.216552000	1.004542000	0.017493000
C	9.295656000	0.095710000	-0.042151000
C	8.311795000	2.195149000	0.770780000
C	9.508087000	2.476597000	1.434944000
C	10.588344000	1.604691000	1.366607000
C	10.472664000	0.426504000	0.637984000
C	9.264604000	-1.223610000	-0.801802000
C	9.285723000	-1.059045000	-2.322074000
H	8.329095000	-0.670420000	-2.701320000
H	9.455690000	-2.034144000	-2.805748000
H	10.090194000	-0.380161000	-2.642975000
C	8.154154000	-2.187139000	-0.378544000
H	10.213759000	-1.714860000	-0.529062000
H	8.386689000	-3.205629000	-0.727576000
H	7.188266000	-1.913861000	-0.832349000
H	8.038184000	-2.224738000	0.714916000
C	7.112994000	3.112927000	0.920658000
C	6.342625000	2.760225000	2.195210000
H	5.432871000	3.373726000	2.291458000
H	6.965938000	2.928344000	3.087866000
H	6.044101000	1.700650000	2.201159000
C	7.474280000	4.594526000	0.881786000
H	6.438756000	2.916986000	0.073318000
H	6.561618000	5.210083000	0.880307000
H	8.057023000	4.845914000	-0.017929000

H	8.066272000	4.898741000	1.759192000
H	11.518167000	1.837720000	1.890563000
H	11.315798000	-0.268824000	0.594648000
H	9.593740000	3.393644000	2.022079000
C	-8.424425000	2.157840000	0.706418000
C	-8.869165000	3.470675000	0.532098000
C	-8.677054000	4.138892000	-0.671236000
C	-8.018713000	3.496805000	-1.713796000
C	-7.550419000	2.184373000	-1.589659000
C	-6.829701000	1.575178000	-2.785082000
H	-9.030747000	5.165150000	-0.793924000
H	-7.852688000	4.025372000	-2.656789000
C	-8.564867000	1.470509000	2.051447000
H	-9.370368000	3.981946000	1.357048000
H	-8.473887000	0.387326000	1.880145000
C	-9.914595000	1.715393000	2.719128000
C	-7.407748000	1.877115000	2.966760000
H	-10.750145000	1.454507000	2.051295000
H	-10.042542000	2.767473000	3.018366000
H	-10.005240000	1.107475000	3.632419000
H	-6.434536000	1.655351000	2.502533000
H	-7.458045000	1.343984000	3.929428000
H	-7.433506000	2.959032000	3.172597000
C	-7.627074000	0.479102000	-3.493166000
H	-6.744418000	2.409155000	-3.501781000
C	-5.397991000	1.110098000	-2.509628000
H	-4.851260000	0.993206000	-3.458601000
H	-5.379460000	0.124953000	-2.016546000
H	-4.843849000	1.830769000	-1.889839000

H	-7.675935000	-0.440908000	-2.892129000
H	-7.145133000	0.218555000	-4.448940000
H	-8.654123000	0.806863000	-3.713898000

**B**			
C	-7.109058000	0.187552000	-0.888716000
C	-5.852127000	2.395116000	-1.074235000
Zn	-4.471833000	0.209447000	0.206268000
N	-4.667331000	1.962881000	-0.679720000
C	-5.990261000	3.816394000	-1.546374000
H	-7.041109000	4.086778000	-1.703878000
H	-5.447756000	3.958997000	-2.493594000
H	-5.539783000	4.510196000	-0.820727000
C	-7.004665000	1.582043000	-1.074583000
H	-7.933036000	2.086090000	-1.340232000
N	-2.562221000	-0.320242000	0.784649000
N	-4.142464000	0.304731000	2.257970000
C	-2.885286000	-0.184975000	2.104752000
C	-1.401587000	-0.643254000	0.289405000
C	-1.188564000	-0.645658000	-1.186767000
C	-0.266390000	-0.977706000	1.201166000
C	-4.749795000	0.648939000	3.355937000
C	-6.138910000	1.195394000	3.289099000
C	-4.065663000	0.496241000	4.674829000
H	-6.462112000	1.317366000	2.244335000
H	-6.213889000	2.180887000	3.783258000
H	-6.871117000	0.540113000	3.798023000
H	-3.783541000	-0.558247000	4.857569000
H	-4.698799000	0.829560000	5.508697000

H	-3.122862000	1.075430000	4.708009000
H	-2.074359000	-0.260732000	-1.707234000
H	-0.961601000	-1.653977000	-1.579557000
H	-0.331797000	-0.007553000	-1.465584000
H	-0.020124000	-0.126992000	1.866216000
H	0.637987000	-1.248922000	0.639455000
H	-0.525800000	-1.821046000	1.868138000
H	-2.172599000	-0.319902000	2.921522000
C	7.250966000	0.062228000	1.583109000
C	8.471758000	-0.522963000	2.242538000
C	6.595098000	1.107241000	2.261385000
C	5.435487000	1.810621000	1.887737000
N	4.732494000	1.547212000	0.791948000
Zn	5.137068000	0.067874000	-0.465917000
C	4.191626000	-0.658286000	-2.040431000
C	2.729402000	-1.049852000	-1.844460000
H	4.769641000	-1.523415000	-2.412480000
H	4.261124000	0.102636000	-2.839243000
H	2.604007000	-1.818275000	-1.061770000
H	2.268395000	-1.461707000	-2.760718000
H	2.115370000	-0.187036000	-1.540807000
H	8.807805000	0.097323000	3.082138000
H	9.294909000	-0.639916000	1.524703000
H	8.245415000	-1.531321000	2.624092000
H	7.047130000	1.411842000	3.204531000
C	3.596876000	2.317896000	0.450998000
C	2.322342000	1.751156000	0.530403000
C	3.713403000	3.620130000	-0.054314000
C	1.187949000	2.453992000	0.132272000

H	2.221051000	0.731813000	0.908072000
C	2.589339000	4.337309000	-0.443919000
H	4.705972000	4.065553000	-0.155789000
C	1.321154000	3.757555000	-0.351946000
H	2.673084000	5.350480000	-0.841509000
O	0.268824000	4.520975000	-0.765432000
H	0.205161000	1.984426000	0.198870000
C	-0.982044000	3.957960000	-0.754383000
C	-1.716895000	3.901243000	0.428428000
C	-1.503225000	3.436702000	-1.935536000
C	-2.961865000	3.282714000	0.436078000
H	-1.288670000	4.315147000	1.343543000
C	-3.490576000	2.732697000	-0.739445000
H	-3.528969000	3.184696000	1.364365000
C	-2.755776000	2.830265000	-1.927057000
H	-3.159196000	2.383568000	-2.838856000
H	-0.905484000	3.490066000	-2.847430000
C	4.974644000	2.906925000	2.812635000
H	5.127967000	3.897511000	2.358692000
H	3.895900000	2.818470000	3.010163000
H	5.520704000	2.875544000	3.763040000
C	-8.481549000	-0.409075000	-1.058553000
H	-8.451021000	-1.353712000	-1.615796000
H	-8.893842000	-0.643432000	-0.064171000
H	-9.163169000	0.287295000	-1.561100000
C	7.568234000	-1.462176000	-0.226169000
C	7.330007000	-2.809043000	0.125314000
C	8.009431000	-3.814698000	-0.566547000
C	8.902138000	-3.506450000	-1.586211000

C	9.113566000	-2.176801000	-1.932541000
C	8.455419000	-1.132515000	-1.274246000
C	8.747527000	0.289630000	-1.733239000
H	9.426769000	-4.303177000	-2.118549000
H	9.805808000	-1.931424000	-2.742978000
C	6.300157000	-3.164394000	1.181481000
H	7.831673000	-4.860407000	-0.305562000
H	6.149844000	-2.273843000	1.809952000
C	6.741831000	-4.300225000	2.099415000
C	4.956128000	-3.473689000	0.518702000
H	7.723189000	-4.094925000	2.554413000
H	6.816678000	-5.259286000	1.563310000
H	6.011918000	-4.441148000	2.911397000
H	4.618793000	-2.633441000	-0.106696000
H	4.179164000	-3.675591000	1.273128000
H	5.035873000	-4.356279000	-0.135775000
C	9.564472000	1.104093000	-0.729289000
H	9.387032000	0.160967000	-2.622703000
C	7.525213000	1.080303000	-2.203442000
H	7.845846000	1.951926000	-2.795575000
H	6.947400000	1.476017000	-1.353068000
H	6.861362000	0.471351000	-2.834794000
H	8.968212000	1.374535000	0.154615000
H	9.904744000	2.043879000	-1.192618000
H	10.456018000	0.553158000	-0.393600000
N	6.842365000	-0.433882000	0.423553000
C	-6.245896000	-2.025344000	-0.637813000
C	-6.563090000	-2.761997000	0.522935000
C	-6.699267000	-4.150566000	0.427972000

C	-6.521472000	-4.809756000	-0.779827000
C	-6.179782000	-4.076007000	-1.910779000
C	-6.025763000	-2.686728000	-1.871247000
C	-5.602373000	-2.000382000	-3.164359000
H	-6.634393000	-5.894642000	-0.840602000
H	-6.014800000	-4.592449000	-2.860594000
C	-6.749282000	-2.091655000	1.869064000
H	-6.950561000	-4.723348000	1.324457000
H	-6.592352000	-1.014546000	1.720596000
C	-5.702651000	-2.570689000	2.874673000
C	-8.163784000	-2.286533000	2.414505000
H	-4.682094000	-2.382634000	2.509164000
H	-5.803060000	-3.649760000	3.073622000
H	-5.818825000	-2.040666000	3.832680000
H	-8.927744000	-1.940687000	1.702203000
H	-8.297009000	-1.726826000	3.353702000
H	-8.369955000	-3.347283000	2.629277000
C	-4.301160000	-1.212118000	-3.040656000
H	-5.390617000	-2.835659000	-3.852832000
C	-6.689670000	-1.164478000	-3.840957000
H	-6.372649000	-0.901417000	-4.862685000
H	-6.877318000	-0.220760000	-3.308491000
H	-7.637825000	-1.717642000	-3.917926000
H	-4.445699000	-0.283461000	-2.469287000
H	-3.927743000	-0.931778000	-4.038382000
H	-3.522849000	-1.809653000	-2.545494000
N	-6.096829000	-0.613282000	-0.565414000

1^{MMe}			
C	1.284450000	1.594880000	0.041660000
C	1.936179000	-0.868439000	0.087190000
N	-0.027011000	1.460950000	-0.093620000
Zn	-0.891015000	-0.305556000	-0.181949000
N	0.731097000	-1.406761000	-0.038169000
C	-0.863154000	2.636612000	-0.174129000
C	0.587909000	-2.844208000	-0.064559000
C	-2.806063000	-0.778591000	-0.349046000
C	-3.703139000	-0.227357000	0.758997000
C	1.876703000	2.978160000	0.111823000
C	3.134513000	-1.774422000	0.198480000
H	-0.815241000	3.259679000	0.737774000
H	-1.910368000	2.329483000	-0.304690000
H	-0.604427000	3.289854000	-1.027290000
H	-0.475794000	-3.101323000	-0.167793000
H	0.954416000	-3.328729000	0.858861000
H	1.122522000	-3.313060000	-0.910876000
H	4.063320000	-1.200314000	0.294939000
H	3.219268000	-2.425616000	-0.686025000
H	3.045359000	-2.439451000	1.072248000
H	2.966040000	2.942417000	0.228730000
H	1.454958000	3.544314000	0.957509000
H	1.644364000	3.552435000	-0.799178000
C	2.188862000	0.517184000	0.125786000
H	3.236458000	0.796226000	0.234497000
H	-3.406937000	-0.599319000	1.754312000
H	-4.767003000	-0.497638000	0.626649000
H	-3.664902000	0.873890000	0.817230000

H	-2.908754000	-1.877950000	-0.393010000
H	-3.168995000	-0.420455000	-1.330116000

A^{MMe}

C	1.274556000	1.103215000	0.000075000
C	-1.274562000	1.103231000	0.000160000
N	1.484850000	-0.206028000	0.000109000
Zn	-0.000021000	-1.485226000	-0.000018000
N	-1.484844000	-0.206003000	0.000213000
C	2.837573000	-0.715045000	-0.000029000
C	-2.837550000	-0.715061000	-0.000139000
C	2.458242000	2.034523000	-0.000133000
C	-2.458208000	2.034588000	-0.000049000
H	3.413111000	-0.400000000	0.889364000
H	3.413498000	-0.398673000	-0.888693000
H	2.811404000	-1.813719000	-0.000827000
H	-3.412750000	-0.400089000	-0.889799000
H	-3.413872000	-0.398612000	0.888229000
H	-2.811357000	-1.813732000	0.000781000
H	-2.142157000	3.084162000	0.000267000
H	-3.094027000	1.863214000	0.883072000
H	-3.093210000	1.863490000	-0.883839000
H	2.142235000	3.084112000	0.000532000
H	3.093078000	1.863668000	-0.884085000
H	3.094219000	1.862859000	0.882819000
C	0.000012000	1.704267000	0.000132000
H	0.000021000	2.793794000	0.000184000
H	0.000029000	-3.047788000	0.000174000

C^{MMe}

C	1.141739000	-1.278668000	1.273155000
C	1.143048000	1.279622000	1.272059000
N	1.748097000	-1.490135000	0.120870000
Zn	2.187966000	-0.001145000	-1.129117000
N	1.749583000	1.489441000	0.119577000
C	2.009196000	-2.839433000	-0.318717000
C	2.012307000	2.838047000	-0.321104000
C	0.732569000	-2.458197000	2.119027000
C	0.735272000	2.460299000	2.116993000
H	2.610813000	-3.420161000	0.403972000
H	2.570988000	-2.809513000	-1.262848000
H	1.082267000	-3.413812000	-0.505576000
H	2.573792000	2.806681000	-1.265373000
H	2.614892000	3.418530000	0.400983000
H	1.086094000	3.413526000	-0.508135000
H	0.232726000	2.142089000	3.038247000
H	0.055812000	3.125915000	1.560741000
H	1.612224000	3.066516000	2.394357000
H	0.230275000	-2.138657000	3.039957000
H	1.608811000	-3.065158000	2.396996000
H	0.052424000	-3.123524000	1.563262000
C	0.833261000	0.000856000	1.790968000
H	0.360130000	0.001516000	2.773628000
H	3.162558000	-0.001665000	-2.361229000
N	-0.081443000	-0.000282000	-1.814793000
C	-1.143695000	0.000032000	-1.353852000
C	-2.445238000	0.000320000	-0.762536000
C	-2.561912000	0.000995000	0.636406000

C	-3.827669000	0.001238000	1.212332000
C	-4.967068000	0.000816000	0.407402000
C	-4.847266000	0.000138000	-0.982802000
C	-3.589285000	-0.000120000	-1.574697000
H	-1.650491000	0.001286000	1.241555000
H	-3.925307000	0.001767000	2.300119000
H	-5.958321000	0.001009000	0.866855000
H	-5.740946000	-0.000201000	-1.610377000
H	-3.482911000	-0.000648000	-2.660993000

TS1^{MMe}

C	-3.052557000	-1.282801000	0.091097000
C	-3.052552000	1.282806000	0.091096000
N	-1.749252000	-1.517399000	0.068302000
Zn	-0.535050000	-0.000002000	0.056893000
N	-1.749248000	1.517400000	0.068301000
C	-1.248158000	-2.869509000	0.018087000
C	-1.248149000	2.869508000	0.018085000
C	-3.997785000	-2.455477000	0.087802000
C	-3.997777000	2.455485000	0.087800000
H	-1.568485000	-3.476833000	0.884010000
H	-0.149488000	-2.853380000	0.018496000
H	-1.564753000	-3.406046000	-0.894548000
H	-0.149478000	2.853376000	0.018495000
H	-1.568474000	3.476834000	0.884007000
H	-1.564742000	3.406045000	-0.894550000
H	-5.043242000	2.127792000	0.116292000
H	-3.851923000	3.072930000	-0.812795000
H	-3.814821000	3.110948000	0.954056000

H	-5.043249000	-2.127781000	0.116294000
H	-3.814831000	-3.110940000	0.954059000
H	-3.851933000	-3.072923000	-0.812793000
C	-3.642397000	0.000003000	0.114739000
H	-4.731546000	0.000005000	0.136442000
N	0.905375000	-0.000006000	-1.564588000
C	1.620344000	-0.000005000	-0.600163000
C	3.020646000	-0.000002000	-0.172973000
C	3.994918000	-0.000002000	-1.182679000
C	5.342188000	0.000000000	-0.841043000
C	5.725760000	0.000002000	0.501643000
C	4.756418000	0.000001000	1.503544000
C	3.405376000	-0.000001000	1.167343000
H	3.672648000	-0.000003000	-2.225980000
H	6.100670000	0.000000000	-1.627373000
H	6.785834000	0.000003000	0.766598000
H	5.054422000	0.000003000	2.554509000
H	2.623628000	-0.000001000	1.931835000
H	0.925501000	-0.000004000	0.915795000

D^{MMe}

C	-3.308249000	1.276503000	0.003136000
C	-3.306842000	-1.277977000	0.003305000
N	-1.999768000	1.493243000	0.017783000
Zn	-0.755716000	0.000776000	0.025213000
N	-1.998198000	-1.493416000	0.018167000
C	-1.484326000	2.844109000	0.027310000
C	-1.481385000	-2.843710000	0.027481000
C	-4.240829000	2.458232000	-0.004409000

C	-4.238276000	-2.460639000	-0.004624000
H	-1.788444000	3.422542000	-0.863661000
H	-0.386005000	2.812120000	0.040856000
H	-1.808426000	3.416542000	0.914920000
H	-0.383118000	-2.810569000	0.042734000
H	-1.783518000	-3.421729000	-0.864454000
H	-1.806279000	-3.417238000	0.914082000
H	-5.287621000	-2.144465000	-0.018414000
H	-4.077372000	-3.092688000	0.883116000
H	-4.055708000	-3.098706000	-0.883827000
H	-5.289863000	2.141004000	-0.017837000
H	-4.059234000	3.096526000	-0.883642000
H	-4.080235000	3.090382000	0.883316000
C	-3.905072000	-0.001024000	-0.004886000
H	-4.994438000	-0.001658000	-0.017225000
N	1.102089000	0.000760000	0.237061000
C	2.021552000	0.000373000	-0.626750000
C	3.469016000	0.000228000	-0.281209000
C	3.876462000	0.000267000	1.058655000
C	5.227781000	0.000165000	1.381941000
C	6.191681000	0.000048000	0.369061000
C	5.795232000	-0.000003000	-0.967247000
C	4.438529000	0.000086000	-1.288371000
H	3.092217000	0.000390000	1.820166000
H	5.539926000	0.000174000	2.429671000
H	7.254428000	-0.000032000	0.624434000
H	6.546432000	-0.000106000	-1.761150000
H	4.119504000	0.000041000	-2.335720000
H	1.858735000	0.000143000	-1.735198000

$TS2^{MMe}$

C	-2.639195000	-1.705944000	-0.910340000
C	-1.881082000	-1.581117000	1.531441000
N	-1.837008000	-0.798917000	-1.444262000
Zn	-0.655593000	0.240019000	-0.299301000
N	-0.956330000	-0.633978000	1.428743000
C	-1.879377000	-0.531038000	-2.863763000
C	-0.146664000	-0.277944000	2.570103000
C	-3.595266000	-2.446989000	-1.807291000
C	-2.114105000	-2.225135000	2.872630000
H	-1.665105000	-1.427473000	-3.472810000
H	-1.123367000	0.225262000	-3.115818000
H	-2.858231000	-0.136703000	-3.190599000
H	0.604640000	0.464090000	2.263350000
H	0.405607000	-1.138424000	2.988599000
H	-0.734790000	0.164591000	3.395519000
H	-2.925269000	-2.961008000	2.829249000
H	-2.366905000	-1.470489000	3.634089000
H	-1.203254000	-2.735500000	3.224674000
H	-4.207161000	-3.158692000	-1.241343000
H	-3.050644000	-3.001502000	-2.587952000
H	-4.267068000	-1.746373000	-2.327965000
C	-2.668036000	-2.048772000	0.460708000
H	-3.393580000	-2.817258000	0.725136000
N	0.951329000	1.231212000	-0.698497000
C	2.208147000	1.149658000	-0.690541000
C	3.018611000	-0.065446000	-0.420920000
C	2.433107000	-1.292877000	-0.080575000
C	3.225430000	-2.406725000	0.171267000

C	4.616758000	-2.313451000	0.086702000
C	5.210313000	-1.098062000	-0.249289000
C	4.413672000	0.017170000	-0.499263000
H	1.345129000	-1.369482000	-0.006448000
H	2.756767000	-3.357804000	0.436022000
H	5.237545000	-3.190806000	0.284217000
H	6.298068000	-1.019200000	-0.316447000
H	4.874128000	0.974341000	-0.761852000
H	2.823765000	2.053141000	-0.895141000
H	-0.203471000	3.123806000	-2.083634000
B	-0.239796000	2.973553000	-0.887101000
O	-1.371692000	2.313188000	-0.265903000
C	-1.559003000	2.924849000	1.003229000
C	-0.239298000	3.651603000	1.260850000
O	0.258037000	3.935683000	-0.025121000
H	-1.795799000	2.161931000	1.761367000
H	-2.407012000	3.629051000	0.943123000
H	0.471327000	3.005722000	1.811483000
H	-0.369324000	4.582575000	1.834336000

E^{MMe}

C	-0.907480000	-2.961635000	-0.439835000
C	0.462172000	-2.030526000	1.517243000
N	-1.446882000	-1.839698000	-0.894110000
Zn	-0.996794000	-0.152655000	-0.038084000
N	0.117821000	-0.752192000	1.451836000
C	-2.335636000	-1.859005000	-2.033681000
C	0.737082000	0.210464000	2.332174000
C	-1.241658000	-4.257431000	-1.129898000

C	1.454886000	-2.459865000	2.564452000
H	-1.836351000	-2.212099000	-2.954254000
H	-2.704263000	-0.842196000	-2.227035000
H	-3.218935000	-2.501810000	-1.871893000
H	0.315516000	1.206969000	2.139865000
H	1.827922000	0.286094000	2.170664000
H	0.572383000	-0.015411000	3.400614000
H	1.631145000	-3.541146000	2.531694000
H	1.108454000	-2.192850000	3.575036000
H	2.417495000	-1.945268000	2.411958000
H	-0.720293000	-5.104128000	-0.668901000
H	-0.968953000	-4.217581000	-2.196320000
H	-2.325034000	-4.452779000	-1.088696000
C	-0.021689000	-3.039973000	0.657141000
H	0.342682000	-4.042406000	0.879042000
N	-0.369337000	1.543889000	-0.927103000
C	0.755725000	2.120716000	-1.018218000
C	2.046911000	1.419693000	-0.912361000
C	2.138134000	0.025049000	-1.036731000
C	3.354357000	-0.619459000	-0.846393000
C	4.498670000	0.119559000	-0.535795000
C	4.423698000	1.508007000	-0.432401000
C	3.204894000	2.153841000	-0.626582000
H	1.248789000	-0.554087000	-1.295034000
H	3.410372000	-1.706225000	-0.943714000
H	5.453942000	-0.389114000	-0.384739000
H	5.318659000	2.089603000	-0.199461000
H	3.139636000	3.242231000	-0.541152000
H	0.802066000	3.219259000	-1.137561000

H	-2.245970000	2.311472000	-1.994553000
B	-1.757804000	2.250339000	-0.870245000
O	-2.491636000	1.258014000	0.058495000
C	-2.939090000	1.993292000	1.177087000
C	-2.030315000	3.220763000	1.196992000
O	-1.773380000	3.493077000	-0.145356000
H	-2.877423000	1.385991000	2.095325000
H	-3.993561000	2.287740000	1.026636000
H	-1.096891000	2.994118000	1.759367000
H	-2.503591000	4.087928000	1.688216000

F^{MMe}

C	-1.007327000	2.701143000	0.808017000
C	-1.894227000	1.768246000	-1.407181000
N	-0.512387000	1.590027000	1.341058000
Zn	-0.731673000	-0.099092000	0.395359000
N	-1.576806000	0.491693000	-1.259946000
C	0.160809000	1.628060000	2.618726000
C	-1.816875000	-0.455554000	-2.327521000
C	-0.874832000	3.990875000	1.573905000
C	-2.556187000	2.208573000	-2.685811000
H	1.028368000	2.312293000	2.623679000
H	0.536703000	0.624627000	2.862679000
H	-0.506811000	1.936547000	3.443264000
H	-1.464511000	-1.450021000	-2.016603000
H	-1.281679000	-0.185193000	-3.255479000
H	-2.888743000	-0.541702000	-2.582708000
H	-2.785788000	3.280218000	-2.671863000
H	-3.491528000	1.652145000	-2.854995000

H	-1.907064000	2.003218000	-3.551864000
H	-1.334814000	4.826787000	1.034534000
H	0.184649000	4.234457000	1.752713000
H	-1.351049000	3.910165000	2.563830000
C	-1.649137000	2.774076000	-0.444525000
H	-1.994985000	3.768582000	-0.724196000
N	0.608670000	-1.598284000	0.435220000
C	1.839686000	-1.847095000	0.266688000
C	2.866528000	-0.866967000	-0.119601000
C	2.577269000	0.495008000	-0.295114000
C	3.581513000	1.383240000	-0.658725000
C	4.887629000	0.927510000	-0.854359000
C	5.186270000	-0.422714000	-0.681480000
C	4.180269000	-1.312775000	-0.314467000
H	1.560824000	0.862362000	-0.137232000
H	3.345100000	2.441486000	-0.792345000
H	5.674190000	1.629332000	-1.142066000
H	6.206254000	-0.783224000	-0.832800000
H	4.409276000	-2.373284000	-0.176872000
H	2.209263000	-2.878309000	0.415929000
H	-0.090876000	-3.439247000	1.672344000
B	-0.463567000	-2.648705000	0.803830000
O	-1.616524000	-1.755127000	1.274724000
C	-2.813500000	-2.289946000	0.747933000
C	-2.355988000	-3.490757000	-0.105835000
O	-1.015219000	-3.245847000	-0.397745000
H	-3.328666000	-1.523899000	0.140563000
H	-3.495743000	-2.590836000	1.561077000
H	-2.942031000	-3.590491000	-1.035342000

H	-2.476110000	-4.431532000	0.469768000

TS3^{MMe}

C	1.735075000	-2.592715000	0.220210000
C	3.344260000	-0.642021000	-0.143420000
N	0.587662000	-2.013731000	-0.123366000
Zn	0.555271000	-0.082530000	-0.405960000
N	2.483304000	0.285419000	-0.524187000
C	-0.598741000	-2.824379000	-0.275708000
C	2.958901000	1.563433000	-1.009575000
C	1.731549000	-4.060349000	0.562144000
C	4.816651000	-0.325086000	-0.152310000
H	-0.436593000	-3.694464000	-0.935454000
H	-1.401607000	-2.223207000	-0.720853000
H	-0.982880000	-3.210705000	0.686980000
H	2.106353000	2.234316000	-1.182698000
H	3.516898000	1.464631000	-1.958609000
H	3.629637000	2.065944000	-0.289628000
H	5.412124000	-1.174232000	0.202437000
H	5.036544000	0.547012000	0.483604000
H	5.152604000	-0.065626000	-1.168803000
H	2.716433000	-4.388543000	0.913925000
H	1.463536000	-4.667511000	-0.317514000
H	0.987128000	-4.285662000	1.341596000
C	2.984109000	-1.947150000	0.265019000
H	3.815168000	-2.575023000	0.584494000
N	-0.719805000	1.199375000	-1.142113000
C	-2.019821000	1.388895000	-0.977015000
C	-2.993297000	0.417579000	-0.413554000

C	-2.662081000	-0.373024000	0.692779000
C	-3.591770000	-1.271023000	1.207213000
C	-4.845740000	-1.401569000	0.608196000
C	-5.182009000	-0.612539000	-0.491763000
C	-4.264738000	0.310838000	-0.986792000
H	-1.685714000	-0.243253000	1.167111000
H	-3.339230000	-1.872474000	2.083651000
H	-5.570543000	-2.114177000	1.009435000
H	-6.165867000	-0.709853000	-0.956441000
H	-4.527972000	0.949836000	-1.834536000
H	-2.523879000	2.207167000	-1.531146000
H	-1.788255000	2.567949000	0.297253000
B	-0.555772000	2.227776000	-0.030564000
O	0.005472000	1.483518000	1.141712000
C	0.865137000	2.366623000	1.825574000
C	0.708319000	3.712421000	1.089895000
O	0.272558000	3.380840000	-0.197817000
H	1.901215000	1.987253000	1.765091000
H	0.589698000	2.439576000	2.891921000
H	1.654071000	4.277110000	1.039780000
H	-0.035332000	4.349068000	1.611162000

G^MMe^

C	2.020541000	-2.362738000	0.645062000
C	3.485678000	-0.492458000	-0.292678000
N	0.826546000	-1.787598000	0.548158000
Zn	0.668212000	-0.012033000	-0.221320000
N	2.542955000	0.393991000	-0.569580000
C	-0.347403000	-2.485959000	1.027357000

C	2.889182000	1.693917000	-1.095658000
C	2.109838000	-3.748609000	1.227922000
C	4.924208000	-0.137545000	-0.559924000
H	-0.547912000	-3.414815000	0.464583000
H	-1.230317000	-1.845463000	0.912651000
H	-0.269989000	-2.757622000	2.095065000
H	1.966429000	2.252511000	-1.300050000
H	3.466236000	1.632944000	-2.035622000
H	3.481375000	2.292925000	-0.380741000
H	5.598061000	-0.957292000	-0.285113000
H	5.216717000	0.759400000	0.008733000
H	5.079655000	0.099205000	-1.624516000
H	3.142101000	-4.117046000	1.226048000
H	1.485013000	-4.455699000	0.660023000
H	1.738291000	-3.763164000	2.264854000
C	3.236401000	-1.771576000	0.248613000
H	4.121127000	-2.388209000	0.403450000
N	-0.768962000	1.143182000	-0.677773000
C	-1.901047000	0.766326000	-1.489408000
C	-2.699080000	-0.368100000	-0.889463000
C	-3.302322000	-0.206083000	0.365029000
C	-4.004662000	-1.250352000	0.958436000
C	-4.111210000	-2.482894000	0.308621000
C	-3.516562000	-2.655691000	-0.938591000
C	-2.817060000	-1.602613000	-1.531402000
H	-3.209668000	0.757886000	0.873073000
H	-4.473783000	-1.106032000	1.935098000
H	-4.658870000	-3.304876000	0.776012000
H	-3.593686000	-3.616610000	-1.453784000

H	-2.347046000	-1.742772000	-2.509498000
H	-1.584520000	0.465311000	-2.505341000
H	-2.571169000	1.634521000	-1.621770000
B	-0.751176000	2.348032000	0.014001000
O	0.302896000	2.681713000	0.871164000
C	0.115468000	4.018146000	1.273712000
C	-1.359153000	4.310442000	0.953415000
O	-1.711501000	3.351224000	-0.016790000
H	0.792628000	4.682125000	0.703988000
H	0.350866000	4.137359000	2.343588000
H	-1.515651000	5.329250000	0.563791000
H	-1.998215000	4.191189000	1.848173000

TS4^{MMe}

C	-2.613196000	-2.091383000	-1.112409000
C	-3.652187000	0.194542000	-0.632970000
N	-1.682355000	-2.115064000	-0.171903000
Zn	-1.371846000	-0.518121000	0.922076000
N	-2.903253000	0.576007000	0.395724000
C	-0.897605000	-3.311453000	0.044820000
C	-3.248813000	1.769261000	1.134347000
C	-2.793292000	-3.299758000	-1.993103000
C	-4.787316000	1.082544000	-1.071748000
H	-1.507713000	-4.144681000	0.439743000
H	-0.100572000	-3.096346000	0.769139000
H	-0.411425000	-3.674072000	-0.878116000
H	-2.479053000	1.962336000	1.891866000
H	-4.222216000	1.675511000	1.649865000
H	-3.309422000	2.667065000	0.493042000

H	-5.307824000	0.664705000	-1.941043000
H	-4.422851000	2.088394000	-1.334785000
H	-5.519995000	1.214235000	-0.259764000
H	-3.592066000	-3.144922000	-2.727460000
H	-3.041105000	-4.188822000	-1.391909000
H	-1.862777000	-3.533710000	-2.534279000
C	-3.482900000	-1.004381000	-1.351728000
H	-4.175320000	-1.144532000	-2.181000000
N	0.632893000	0.331470000	0.091210000
C	1.121509000	-0.390341000	-1.091788000
C	2.607819000	-0.240645000	-1.310144000
C	3.137777000	0.953064000	-1.814747000
C	4.511612000	1.101830000	-1.989954000
C	5.377605000	0.057430000	-1.666218000
C	4.859373000	-1.136774000	-1.168284000
C	3.484216000	-1.282313000	-0.991144000
H	2.456983000	1.769069000	-2.069763000
H	4.910292000	2.039113000	-2.386536000
H	6.455369000	0.173964000	-1.804516000
H	5.530558000	-1.961381000	-0.914596000
H	3.075209000	-2.209857000	-0.584145000
H	0.576431000	-0.011906000	-1.968413000
H	0.876980000	-1.454859000	-0.986715000
B	0.235313000	1.687563000	-0.077219000
O	0.166871000	2.306934000	-1.307304000
C	-0.473069000	3.553270000	-1.112185000
C	-0.404322000	3.785185000	0.404182000
O	-0.180853000	2.500762000	0.947769000
H	-1.515568000	3.487720000	-1.471588000

H	0.038553000	4.337216000	-1.691008000
H	-1.332094000	4.213768000	0.813847000
H	0.431169000	4.450667000	0.683064000
H	-0.428217000	-0.439247000	2.233026000
B	1.040345000	-0.200102000	1.430108000
O	1.577201000	-1.500631000	1.459778000
C	2.505096000	-1.541688000	2.521753000
C	2.240428000	-0.252129000	3.310173000
O	1.659440000	0.617257000	2.369505000
H	3.157023000	0.196088000	3.724016000
H	1.538495000	-0.435473000	4.146560000
H	3.530812000	-1.563104000	2.108903000
H	2.358771000	-2.449632000	3.129545000

H^{MMe}

C	-2.304707000	1.936221000	-0.950842000
C	-3.255055000	0.161775000	0.617877000
N	-1.625149000	1.150678000	-1.774910000
Zn	-1.448769000	-0.778709000	-1.426816000
N	-2.725915000	-0.913595000	0.062499000
C	-0.954306000	1.725735000	-2.918159000
C	-2.994271000	-2.216389000	0.626385000
C	-2.295449000	3.423148000	-1.186940000
C	-4.145807000	0.001479000	1.821814000
H	-1.657809000	2.198487000	-3.627681000
H	-0.424681000	0.933000000	-3.464595000
H	-0.208223000	2.492596000	-2.641449000
H	-2.560255000	-2.985913000	-0.026489000
H	-4.074316000	-2.427607000	0.717682000

H	-2.529781000	-2.335124000	1.620071000
H	-4.494564000	0.971363000	2.196323000
H	-3.610882000	-0.520307000	2.630930000
H	-5.028704000	-0.610988000	1.579385000
H	-2.920898000	3.949505000	-0.456208000
H	-2.657939000	3.672264000	-2.196271000
H	-1.266733000	3.810978000	-1.110366000
C	-3.039645000	1.480943000	0.161651000
H	-3.555384000	2.257387000	0.726621000
N	0.834747000	-0.343809000	0.101350000
C	1.668002000	0.291968000	-0.929956000
C	3.070479000	0.597376000	-0.462533000
C	3.393923000	1.866011000	0.031451000
C	4.681732000	2.146196000	0.485365000
C	5.665511000	1.159394000	0.451032000
C	5.353531000	-0.107614000	-0.041310000
C	4.066278000	-0.386370000	-0.495551000
H	2.616928000	2.633047000	0.067243000
H	4.919221000	3.142826000	0.866256000
H	6.676173000	1.378208000	0.804643000
H	6.121412000	-0.884704000	-0.075968000
H	3.815733000	-1.379024000	-0.876400000
H	1.178092000	1.223841000	-1.238036000
H	1.699459000	-0.377906000	-1.800684000
B	0.247898000	0.518970000	1.081581000
O	0.330393000	1.886030000	0.964401000
C	-0.314387000	2.462031000	2.081637000
C	-0.795510000	1.265243000	2.929265000
O	-0.400904000	0.112628000	2.213290000

H	-1.154627000	3.084478000	1.736416000
H	0.397469000	3.105012000	2.624626000
H	-1.889564000	1.265558000	3.053161000
H	-0.336660000	1.247908000	3.931461000
H	-0.763459000	-1.921634000	-2.255747000
B	0.868204000	-1.771268000	0.184671000
O	1.686390000	-2.532267000	-0.615081000
C	1.269981000	-3.876458000	-0.466710000
C	0.451297000	-3.877323000	0.832804000
O	0.095783000	-2.522312000	1.030507000
H	1.040684000	-4.230702000	1.696962000
H	-0.456820000	-4.496046000	0.762412000
H	2.146046000	-4.541125000	-0.424813000
H	0.653976000	-4.158354000	-1.337460000

1^{MPh}

C	1.124291000	2.073988000	0.066114000
C	-1.413958000	1.980952000	0.121209000
N	1.363164000	0.769653000	0.041946000
Zn	-0.048384000	-0.620142000	0.112091000
N	-1.559781000	0.660328000	0.091706000
C	0.083297000	-2.583956000	0.185609000
C	1.378601000	-3.165138000	-0.378516000
C	2.289201000	3.027218000	0.042958000
C	-2.634949000	2.863315000	0.179153000
H	-2.366225000	3.876819000	0.500992000
H	-3.384244000	2.450127000	0.869991000
H	-3.119989000	2.936977000	-0.805885000
H	1.955357000	4.070137000	0.094900000

H	2.883116000	2.888443000	-0.873060000
H	2.969802000	2.825580000	0.883997000
C	-0.166785000	2.631492000	0.122001000
H	-0.205636000	3.719324000	0.166649000
H	1.515048000	-2.912565000	-1.443264000
H	1.417095000	-4.267708000	-0.307693000
H	2.269143000	-2.778772000	0.144100000
H	-0.789165000	-3.010926000	-0.340381000
H	-0.040158000	-2.897410000	1.238714000
C	-2.835008000	0.061559000	0.010136000
C	-3.287534000	-0.745206000	1.062278000
C	-3.628818000	0.180684000	-1.138776000
C	-4.516456000	-1.394210000	0.978201000
H	-2.658683000	-0.854483000	1.949008000
C	-4.858258000	-0.468074000	-1.218023000
H	-3.261248000	0.773675000	-1.979431000
C	-5.310297000	-1.255316000	-0.159547000
H	-4.855116000	-2.016525000	1.810099000
H	-5.464089000	-0.365993000	-2.121861000
H	-6.272787000	-1.767313000	-0.225777000
C	2.685218000	0.272438000	-0.015449000
C	3.358532000	-0.080856000	1.159998000
C	3.305848000	0.043453000	-1.249168000
C	4.630897000	-0.644496000	1.101085000
H	2.862399000	0.081219000	2.119824000
C	4.578446000	-0.519569000	-1.304126000
H	2.767963000	0.299623000	-2.165040000
C	5.246443000	-0.865776000	-0.130304000
H	5.142999000	-0.919306000	2.026479000

H	5.048574000	-0.697562000	-2.274383000
H	6.241432000	-1.313991000	-0.175276000

A^{MPh}

C	1.270355000	1.694422000	-0.177534000
C	-1.270371000	1.694338000	-0.177581000
N	1.466679000	0.383811000	-0.081677000
Zn	0.000068000	-0.938715000	-0.074678000
C	2.775529000	-0.140943000	0.026348000
C	2.462813000	2.612402000	-0.240976000
C	-2.462866000	2.612262000	-0.241296000
H	-2.160014000	3.637124000	-0.486242000
H	-2.998798000	2.627280000	0.719552000
H	-3.184087000	2.257942000	-0.992944000
H	2.998325000	2.627676000	0.720107000
H	2.159975000	3.637195000	-0.486236000
H	3.184379000	2.258001000	-0.992249000
C	-0.000022000	2.294974000	-0.240851000
H	-0.000063000	3.379225000	-0.345343000
N	-1.466669000	0.383734000	-0.081571000
C	-2.775535000	-0.140982000	0.026406000
C	-3.476128000	-0.084206000	1.237741000
C	-3.362218000	-0.788630000	-1.066937000
C	-4.632781000	-1.348279000	-0.956452000
C	-5.332384000	-1.274969000	0.247181000
C	-4.746981000	-0.643494000	1.343965000
H	-3.004645000	0.393919000	2.099800000
H	-5.281081000	-0.592740000	2.296029000
H	-2.804902000	-0.848045000	-2.004589000

H	-5.078267000	-1.849296000	-1.819334000
C	3.476158000	-0.084097000	1.237660000
C	4.746980000	-0.643449000	1.343886000
C	5.332323000	-1.275028000	0.247135000
C	4.632698000	-1.348381000	-0.956481000
C	3.362155000	-0.788700000	-1.066970000
H	6.327294000	-1.717300000	0.333277000
H	5.078147000	-1.849475000	-1.819339000
H	5.281113000	-0.592651000	2.295933000
H	3.004716000	0.394094000	2.099701000
H	2.804789000	-0.848138000	-2.004589000
H	0.000042000	-2.492395000	-0.075245000
H	-6.327375000	-1.717208000	0.333308000

C^{MPh}

C	1.223928000	-0.631866000	-1.238453000
C	-0.804404000	-1.430980000	0.091673000
N	2.059026000	-0.449047000	-0.227042000
Zn	1.475243000	-0.548988000	1.688201000
N	-0.300575000	-1.403760000	1.305488000
C	-1.035070000	-1.899834000	2.445942000
C	1.684913000	-0.369758000	-2.651304000
H	-0.330241000	-2.364729000	3.152027000
H	-1.803213000	-2.642662000	2.179017000
H	-1.536013000	-1.081276000	2.994261000
H	0.826991000	-0.271618000	-3.328175000
H	2.317087000	-1.190225000	-3.022550000
H	2.293525000	0.545010000	-2.701259000
C	-0.115528000	-1.040359000	-1.083935000

H	-0.692934000	-1.123322000	-2.005106000
H	2.306870000	-0.634835000	3.012043000
N	0.415148000	1.542394000	1.431467000
C	-0.493156000	2.013842000	0.888897000
C	-1.631353000	2.568620000	0.226201000
C	-2.136627000	1.934721000	-0.919622000
C	-3.260799000	2.459608000	-1.547004000
C	-3.874300000	3.606938000	-1.044269000
C	-3.366978000	4.237860000	0.092620000
C	-2.246343000	3.723429000	0.734168000
H	-1.642173000	1.032926000	-1.288194000
H	-3.662625000	1.964407000	-2.433320000
H	-4.757703000	4.013936000	-1.541864000
H	-3.849702000	5.136177000	0.483041000
H	-1.842379000	4.205249000	1.626359000
C	-2.222964000	-1.855015000	-0.140436000
C	-2.519172000	-2.947361000	-0.962608000
C	-3.277615000	-1.123311000	0.420144000
C	-3.842637000	-3.306907000	-1.212142000
H	-1.698804000	-3.519290000	-1.402819000
C	-4.599756000	-1.473220000	0.159323000
H	-3.056320000	-0.263797000	1.057242000
C	-4.885773000	-2.568922000	-0.655095000
H	-4.060252000	-4.168696000	-1.847778000
H	-5.412131000	-0.886487000	0.594984000
H	-5.923102000	-2.847870000	-0.855068000
C	3.404924000	-0.095016000	-0.441887000
C	4.315055000	-0.981575000	-1.033388000
C	3.869878000	1.146254000	0.013687000

C	5.650534000	-0.620913000	-1.192836000
H	3.966157000	-1.967602000	-1.348664000
C	5.206100000	1.501878000	-0.146962000
H	3.162745000	1.824227000	0.497061000
C	6.102393000	0.623601000	-0.755469000
H	6.347762000	-1.325270000	-1.653729000
H	5.551106000	2.475177000	0.210914000
H	7.151380000	0.902825000	-0.877330000

TS1^{MPh}

C	1.283402000	-2.945078000	-0.169361000
C	-1.279818000	-2.946558000	-0.169294000
N	1.504491000	-1.643310000	-0.035103000
Zn	0.000321000	-0.395828000	-0.020529000
N	-1.502401000	-1.645046000	-0.035026000
C	2.454997000	-3.887098000	-0.280103000
C	-2.450351000	-3.889907000	-0.279961000
H	-2.137020000	-4.843777000	-0.721296000
H	-3.251219000	-3.452334000	-0.893026000
H	-2.884346000	-4.101277000	0.709135000
H	3.255520000	-3.448459000	-0.892851000
H	2.142778000	-4.841153000	-0.721832000
H	2.888998000	-4.098364000	0.709013000
C	0.002126000	-3.528910000	-0.244824000
H	0.002747000	-4.608157000	-0.393658000
N	-0.000618000	1.054916000	-1.619031000
C	-0.001050000	1.750264000	-0.637665000
C	-0.001999000	3.142893000	-0.189533000
C	-0.002770000	4.132306000	-1.184177000

C	-0.003677000	5.473849000	-0.821422000
C	-0.003821000	5.835699000	0.527337000
C	-0.003056000	4.850941000	1.514074000
C	-0.002148000	3.505548000	1.156780000
H	-0.002638000	3.826233000	-2.232315000
H	-0.004278000	6.244865000	-1.595433000
H	-0.004534000	6.891423000	0.809096000
H	-0.003170000	5.132326000	2.569519000
H	-0.001540000	2.713194000	1.910043000
H	-0.000503000	1.043972000	0.850062000
C	-2.798106000	-1.101468000	0.080524000
C	-3.614926000	-1.371140000	1.186543000
C	-3.248546000	-0.201057000	-0.894447000
C	-4.869045000	-0.776185000	1.296961000
H	-3.248011000	-2.037439000	1.970350000
C	-4.502414000	0.392683000	-0.776817000
H	-2.596171000	0.027423000	-1.740529000
C	-5.320698000	0.104903000	0.315051000
H	-5.494753000	-0.995284000	2.165818000
H	-4.841563000	1.089486000	-1.547226000
H	-6.303065000	0.573680000	0.406192000
C	2.799562000	-1.098210000	0.080471000
C	3.616783000	-1.367104000	1.186385000
C	3.248846000	-0.197062000	-0.894354000
C	4.870182000	-0.770641000	1.296838000
H	3.250733000	-2.033995000	1.970094000
C	4.501997000	0.398181000	-0.776690000
H	2.596141000	0.030782000	-1.740354000
C	5.320699000	0.111189000	0.315072000

H	5.496210000	-0.989143000	2.165614000
H	4.840247000	1.095544000	-1.546986000
H	6.302498000	0.581147000	0.406244000

D^{MPh}

C	-3.113357000	1.282074000	-0.353498000
C	-3.118031000	-1.271529000	-0.353894000
N	-1.817356000	1.484759000	-0.137640000
Zn	-0.541508000	0.000544000	-0.100675000
N	-1.822781000	-1.479023000	-0.138059000
C	-4.043873000	2.458447000	-0.502087000
C	-4.052989000	-2.444327000	-0.502971000
H	-4.942574000	-2.152034000	-1.074749000
H	-4.392532000	-2.809281000	0.478339000
H	-3.557984000	-3.286677000	-1.005999000
H	-4.935479000	2.169390000	-1.072341000
H	-3.546142000	3.298401000	-1.006463000
H	-4.380383000	2.825596000	0.479443000
C	-3.694804000	0.006358000	-0.486619000
H	-4.761084000	0.008340000	-0.711409000
N	1.305584000	-0.002666000	0.126035000
C	2.241286000	-0.003189000	-0.719018000
C	3.680537000	-0.005553000	-0.345957000
C	4.062611000	-0.007339000	1.001325000
C	5.407820000	-0.009513000	1.348979000
C	6.390221000	-0.009935000	0.354068000
C	6.018780000	-0.008176000	-0.989392000
C	4.668248000	-0.005993000	-1.335237000
H	3.265387000	-0.006938000	1.749014000

H	5.700514000	-0.010896000	2.402253000
H	7.448049000	-0.011644000	0.629123000
H	6.784501000	-0.008502000	-1.769290000
H	4.368651000	-0.004594000	-2.388339000
H	2.099684000	-0.001896000	-1.830366000
C	-1.277360000	2.770278000	0.080223000
C	-1.673148000	3.564612000	1.164605000
C	-0.252537000	3.230429000	-0.756803000
C	-1.079354000	4.804877000	1.383173000
H	-2.435848000	3.190629000	1.850805000
C	0.340249000	4.469236000	-0.532617000
H	0.076407000	2.599032000	-1.585424000
C	-0.073948000	5.265586000	0.534341000
H	-1.397441000	5.411165000	2.234887000
H	1.137405000	4.812318000	-1.196254000
H	0.394913000	6.235864000	0.711857000
C	-1.287404000	-2.766449000	0.079841000
C	-1.686992000	-3.559981000	1.163427000
C	-0.263198000	-3.229646000	-0.756279000
C	-1.097576000	-4.802324000	1.382046000
H	-2.449143000	-3.183799000	1.849025000
C	0.325201000	-4.470525000	-0.532042000
H	0.068793000	-2.598968000	-1.584231000
C	-0.092805000	-5.266001000	0.534086000
H	-1.418605000	-5.407932000	2.233140000
H	1.121927000	-4.815939000	-1.194985000
H	0.372621000	-6.237924000	0.711646000

TS2^{MPh}

C	-1.809501000	-1.548857000	-2.006576000
C	0.724248000	-1.534383000	-2.368615000
N	-1.855635000	-0.987332000	-0.805765000
Zn	-0.219901000	-0.524250000	0.171811000
N	1.105602000	-1.028803000	-1.199978000
C	-3.087032000	-1.965346000	-2.690196000
C	1.757846000	-1.839370000	-3.423022000
H	1.291761000	-1.898594000	-4.414080000
H	2.256546000	-2.800944000	-3.227529000
H	2.542639000	-1.069644000	-3.438243000
H	-2.887832000	-2.760587000	-3.419420000
H	-3.542182000	-1.121834000	-3.231517000
H	-3.829455000	-2.318466000	-1.961138000
C	-0.614917000	-1.798050000	-2.713955000
H	-0.753209000	-2.246129000	-3.697807000
N	0.040518000	0.807743000	1.543650000
C	0.242423000	2.043492000	1.671378000
C	0.250226000	3.043122000	0.572996000
C	-0.276043000	2.753693000	-0.693814000
C	-0.216651000	3.694970000	-1.715098000
C	0.369889000	4.941876000	-1.486939000
C	0.885362000	5.245347000	-0.227889000
C	0.817741000	4.302924000	0.796034000
H	-0.749528000	1.784161000	-0.871467000
H	-0.634849000	3.458886000	-2.696656000
H	0.417772000	5.679853000	-2.291353000
H	1.340161000	6.221520000	-0.042960000
H	1.217813000	4.537004000	1.787052000

H	0.464619000	2.473313000	2.671859000
H	-0.969715000	-0.436969000	3.586999000
B	0.038708000	-0.735719000	2.995534000
O	0.015644000	-1.759869000	1.968986000
C	1.235123000	-2.486142000	2.096274000
C	2.149808000	-1.516926000	2.836129000
O	1.264034000	-0.765055000	3.636517000
H	1.610837000	-2.774730000	1.104288000
H	1.048725000	-3.401678000	2.684392000
H	2.687833000	-0.859501000	2.127301000
H	2.893880000	-2.027049000	3.466909000
C	-3.064017000	-0.647121000	-0.162505000
C	-3.323178000	-1.171410000	1.111208000
C	-3.962202000	0.276155000	-0.714335000
C	-4.473527000	-0.801499000	1.801865000
H	-2.602567000	-1.864404000	1.552269000
C	-5.112187000	0.640346000	-0.018825000
H	-3.739755000	0.725970000	-1.684397000
C	-5.376247000	0.100293000	1.239189000
H	-4.661194000	-1.218877000	2.793972000
H	-5.801666000	1.364466000	-0.459887000
H	-6.275867000	0.392511000	1.785331000
C	2.457716000	-0.801710000	-0.871404000
C	3.385085000	-1.848347000	-0.763611000
C	2.865739000	0.497999000	-0.539151000
C	4.688451000	-1.597228000	-0.341721000
H	3.069677000	-2.868892000	-0.992157000
C	4.169222000	0.743131000	-0.116883000
H	2.143401000	1.313722000	-0.611135000

C	5.087424000	-0.301588000	-0.014875000
H	5.396119000	-2.425758000	-0.257620000
H	4.465987000	1.763090000	0.138802000
H	6.107927000	-0.108431000	0.322861000

E^MPh^

C	0.289525000	-2.584677000	0.020639000
C	-1.714140000	-1.147872000	-0.679373000
N	1.236409000	-1.647892000	-0.001727000
Zn	0.809501000	0.154487000	-0.631712000
N	-1.116368000	-0.005283000	-0.963012000
C	-1.860218000	1.165356000	-1.371026000
C	0.630688000	-4.008399000	0.385043000
H	-1.255318000	1.753919000	-2.077082000
H	-2.081278000	1.823408000	-0.511612000
H	-2.814253000	0.917211000	-1.859923000
H	-0.045994000	-4.697119000	-0.137102000
H	0.512689000	-4.187879000	1.464623000
H	1.668768000	-4.252844000	0.123293000
C	-1.059978000	-2.341436000	-0.293127000
H	-1.721669000	-3.200564000	-0.183185000
N	1.404769000	1.901803000	0.147755000
C	0.840390000	2.753088000	0.897786000
C	-0.273721000	2.427252000	1.805715000
C	-0.547369000	1.104480000	2.186115000
C	-1.654258000	0.814630000	2.975067000
C	-2.503160000	1.841100000	3.397086000
C	-2.228550000	3.161127000	3.041761000
C	-1.114462000	3.452224000	2.257740000

H	0.124126000	0.301401000	1.873154000
H	-1.855866000	-0.219049000	3.265662000
H	-3.374323000	1.610699000	4.015197000
H	-2.884433000	3.967269000	3.378407000
H	-0.896879000	4.485827000	1.974300000
H	1.147763000	3.814291000	0.855102000
H	3.601419000	2.064824000	-0.483323000
B	2.476144000	2.196424000	-0.948561000
O	2.094378000	1.081766000	-1.954402000
C	1.792065000	1.716937000	-3.178698000
C	1.449212000	3.149893000	-2.775209000
O	2.269995000	3.416486000	-1.681192000
H	0.969296000	1.196687000	-3.696785000
H	2.681882000	1.695489000	-3.833273000
H	0.371329000	3.225291000	-2.510376000
H	1.635809000	3.871352000	-3.588737000
C	-3.206221000	-1.244794000	-0.736291000
C	-3.834768000	-2.094554000	-1.652014000
C	-3.989273000	-0.506335000	0.159850000
C	-5.224788000	-2.196894000	-1.679144000
H	-3.225304000	-2.672728000	-2.350847000
C	-5.376709000	-0.618360000	0.138423000
H	-3.501672000	0.151824000	0.883614000
C	-5.998343000	-1.460863000	-0.783470000
H	-5.706012000	-2.857366000	-2.404467000
H	-5.977393000	-0.043435000	0.847379000
H	-7.087548000	-1.544414000	-0.802431000
C	2.546023000	-1.853408000	0.474299000
C	3.624013000	-1.427063000	-0.315661000

C	2.809051000	-2.373796000	1.750346000
C	4.930277000	-1.555436000	0.145533000
H	3.420976000	-0.972620000	-1.288481000
C	4.119092000	-2.503144000	2.203678000
H	1.977343000	-2.652872000	2.399671000
C	5.187035000	-2.102244000	1.402345000
H	5.755673000	-1.215209000	-0.484155000
H	4.304636000	-2.908435000	3.201541000
H	6.213365000	-2.198948000	1.763164000

F^MPh

C	-1.058365000	-2.099230000	1.442488000
C	1.307577000	-2.548267000	0.547465000
N	-1.099418000	-0.783064000	1.295531000
Zn	0.416281000	0.179974000	0.481937000
N	1.679758000	-1.302276000	0.264044000
C	-2.229488000	-2.844236000	2.031840000
C	2.244458000	-3.698292000	0.273609000
H	1.669409000	-4.609215000	0.063497000
H	2.885507000	-3.907297000	1.143802000
H	2.906280000	-3.484003000	-0.576373000
H	-1.872118000	-3.713526000	2.599367000
H	-2.895596000	-3.219703000	1.239454000
H	-2.828217000	-2.202195000	2.690899000
C	0.052506000	-2.894723000	1.083492000
H	-0.081014000	-3.961825000	1.263936000
N	-0.197391000	1.559087000	-0.844934000
C	-1.186904000	1.667656000	-1.628143000
C	-1.984402000	0.522783000	-2.097378000

C	-1.499339000	-0.791008000	-2.022737000
C	-2.308186000	-1.860014000	-2.389250000
C	-3.610786000	-1.630639000	-2.838977000
C	-4.094382000	-0.326308000	-2.937331000
C	-3.280817000	0.744494000	-2.576925000
H	-0.477711000	-0.974912000	-1.682618000
H	-1.918982000	-2.878819000	-2.325586000
H	-4.246588000	-2.472232000	-3.124719000
H	-5.109955000	-0.143748000	-3.295969000
H	-3.659280000	1.768225000	-2.640445000
H	-1.521181000	2.665858000	-1.965214000
H	-0.040218000	3.704417000	0.027648000
B	0.629769000	2.697046000	-0.208368000
O	1.103654000	2.006964000	1.094368000
C	2.470772000	2.334244000	1.274874000
C	2.821244000	3.207393000	0.053381000
O	1.868579000	2.898242000	-0.914467000
H	3.080427000	1.415446000	1.304187000
H	2.612195000	2.870979000	2.228173000
H	3.835304000	2.993668000	-0.323378000
H	2.779464000	4.281166000	0.330784000
C	2.976704000	-0.948556000	-0.156068000
C	4.123777000	-1.294129000	0.574579000
C	3.116245000	-0.115505000	-1.275583000
C	5.377871000	-0.839477000	0.175512000
H	4.022895000	-1.894585000	1.480343000
C	4.371071000	0.341040000	-1.665356000
H	2.226177000	0.200609000	-1.823173000
C	5.509779000	-0.022228000	-0.946914000

H	6.259654000	-1.114221000	0.759738000
H	4.451507000	0.999747000	-2.532984000
H	6.493869000	0.340895000	-1.251295000
C	-2.253986000	0.000183000	1.472647000
C	-2.135602000	1.214697000	2.165665000
C	-3.481763000	-0.316059000	0.871746000
C	-3.226299000	2.069328000	2.286073000
H	-1.166729000	1.491098000	2.589134000
C	-4.569586000	0.543286000	0.997680000
H	-3.568319000	-1.216431000	0.262112000
C	-4.452151000	1.735794000	1.709955000
H	-3.111861000	3.010925000	2.828096000
H	-5.514172000	0.283401000	0.513421000
H	-5.306225000	2.410577000	1.801114000

TS3^{MPh}

C	0.339948000	3.073687000	-0.038131000
C	-2.151860000	2.549335000	-0.228698000
N	0.832508000	1.835898000	0.031962000
Zn	-0.354012000	0.265290000	-0.136056000
N	-2.097148000	1.225228000	-0.195112000
C	1.291993000	4.243415000	-0.049586000
C	-3.476625000	3.249552000	-0.405410000
H	-3.322485000	4.267926000	-0.782637000
H	-4.021032000	3.322749000	0.547988000
H	-4.125164000	2.698628000	-1.100841000
H	0.761879000	5.175034000	-0.278772000
H	2.087651000	4.089753000	-0.793897000
H	1.794476000	4.359479000	0.921909000

C	-1.022871000	3.389717000	-0.145769000
H	-1.240504000	4.454539000	-0.215762000
N	-0.090533000	-1.443267000	-1.057687000
C	0.862109000	-2.360048000	-1.043391000
C	2.251894000	-2.224935000	-0.540878000
C	2.517933000	-1.569613000	0.667045000
C	3.814933000	-1.532777000	1.163375000
C	4.858928000	-2.108926000	0.438206000
C	4.602710000	-2.746958000	-0.774170000
C	3.295548000	-2.828445000	-1.248458000
H	1.688257000	-1.144164000	1.236742000
H	4.016478000	-1.044766000	2.118405000
H	5.879627000	-2.064944000	0.825948000
H	5.419791000	-3.200838000	-1.339802000
H	3.078019000	-3.362761000	-2.177775000
H	0.739821000	-3.269319000	-1.668802000
H	0.095644000	-3.274362000	0.268329000
B	-0.747527000	-2.294717000	0.033752000
O	-0.753215000	-1.411772000	1.244620000
C	-1.841188000	-1.821051000	2.045944000
C	-2.589159000	-2.886111000	1.202262000
O	-2.072274000	-2.795975000	-0.093134000
H	-2.478170000	-0.950237000	2.267558000
H	-1.479635000	-2.237477000	3.002556000
H	-3.675642000	-2.701129000	1.180600000
H	-2.424374000	-3.895513000	1.630084000
C	2.235360000	1.660072000	0.066171000
C	2.904901000	1.196770000	-1.073267000
C	2.974530000	1.932532000	1.223377000

C	4.289282000	1.053714000	-1.068573000
H	2.320829000	0.957370000	-1.964733000
C	4.360835000	1.796513000	1.221802000
H	2.447721000	2.259211000	2.123131000
C	5.024384000	1.364983000	0.074342000
H	4.797171000	0.690854000	-1.965035000
H	4.926810000	2.024330000	2.128712000
H	6.111020000	1.254518000	0.075843000
C	-3.285354000	0.459866000	-0.241416000
C	-3.495245000	-0.428946000	-1.299816000
C	-4.236987000	0.529247000	0.786491000
C	-4.649869000	-1.204500000	-1.348252000
H	-2.728223000	-0.525665000	-2.070160000
C	-5.392272000	-0.245058000	0.732690000
H	-4.055636000	1.190795000	1.636808000
C	-5.606850000	-1.111866000	-0.339681000
H	-4.791552000	-1.901470000	-2.176955000
H	-6.125902000	-0.177829000	1.539995000
H	-6.510812000	-1.723854000	-0.379812000

G^{MPh}

C	-2.191187000	-0.954790000	0.332490000
C	-0.274215000	-2.176520000	-0.836240000
N	-1.528863000	0.022987000	0.922754000
Zn	0.339879000	0.316506000	0.461550000
N	0.725883000	-1.379304000	-0.466369000
C	-2.178477000	0.946452000	1.829775000
C	0.000658000	-3.387142000	-1.692602000
H	-2.578107000	1.825419000	1.297066000

H	-1.438208000	1.319758000	2.552739000
H	-3.005524000	0.480447000	2.387494000
H	-0.898874000	-3.664615000	-2.256490000
H	0.288197000	-4.254268000	-1.078748000
H	0.825703000	-3.200567000	-2.394253000
C	-1.619591000	-1.945664000	-0.500433000
H	-2.336688000	-2.643993000	-0.930748000
N	1.519834000	1.795870000	0.580909000
C	1.295343000	3.000797000	-0.178464000
C	-0.040675000	2.920748000	-0.879146000
C	-1.129798000	3.697070000	-0.475617000
C	-2.384945000	3.533539000	-1.063830000
C	-2.573577000	2.576177000	-2.059059000
C	-1.495653000	1.792797000	-2.473584000
C	-0.242384000	1.971655000	-1.893320000
H	-0.990872000	4.435836000	0.318414000
H	-3.223217000	4.153648000	-0.735705000
H	-3.556193000	2.444919000	-2.518905000
H	-1.631172000	1.041855000	-3.255338000
H	0.600511000	1.365085000	-2.237196000
H	2.089306000	3.165273000	-0.934612000
H	1.300403000	3.902574000	0.462680000
B	2.743680000	1.408000000	1.116929000
O	2.820534000	0.231082000	1.867843000
C	4.187055000	-0.079071000	2.023218000
C	4.915087000	1.239629000	1.708875000
O	3.984952000	2.002932000	0.970806000
H	4.469742000	-0.878150000	1.316194000
H	4.385491000	-0.439083000	3.045633000

H	5.832127000	1.081884000	1.118305000
H	5.195669000	1.782848000	2.630009000
C	2.075649000	-1.690479000	-0.709591000
C	2.882009000	-0.766115000	-1.389585000
C	2.670615000	-2.861885000	-0.219078000
C	4.233460000	-1.020818000	-1.602450000
H	2.436962000	0.166311000	-1.739633000
C	4.020902000	-3.118886000	-0.442793000
H	2.067088000	-3.562391000	0.361343000
C	4.809723000	-2.203973000	-1.140139000
H	4.840348000	-0.284183000	-2.134198000
H	4.464057000	-4.039459000	-0.054660000
H	5.869642000	-2.405629000	-1.310105000
C	-3.672791000	-1.061263000	0.508384000
C	-4.511810000	-0.043045000	0.039396000
C	-4.240025000	-2.189015000	1.110540000
C	-5.893238000	-0.152835000	0.171899000
H	-4.070570000	0.831911000	-0.443664000
C	-5.622768000	-2.291681000	1.254130000
H	-3.586047000	-2.985817000	1.473034000
C	-6.452053000	-1.275033000	0.783884000
H	-6.539436000	0.642926000	-0.206604000
H	-6.054689000	-3.172863000	1.734643000
H	-7.536164000	-1.357770000	0.891922000

TS4^{MPh}

C	0.262909000	3.341539000	-1.087134000
C	2.620352000	2.357804000	-1.000966000
N	-0.365851000	2.465221000	-0.316922000

Zn	0.607379000	0.949216000	0.529794000
N	2.399539000	1.297067000	-0.223524000
C	-0.491371000	4.524580000	-1.637843000
C	4.022722000	2.667816000	-1.466077000
H	4.094498000	3.714050000	-1.786431000
H	4.315699000	2.034337000	-2.316383000
H	4.754632000	2.484989000	-0.666574000
H	0.197147000	5.267749000	-2.056874000
H	-1.100182000	4.996133000	-0.852405000
H	-1.189125000	4.215478000	-2.430221000
C	1.634365000	3.273330000	-1.404653000
H	1.999286000	4.091324000	-2.024169000
N	-0.284431000	-1.034367000	-0.108753000
C	-1.153054000	-0.931907000	-1.293491000
C	-2.274323000	-1.942623000	-1.327215000
C	-2.019653000	-3.291286000	-1.602255000
C	-3.056680000	-4.221704000	-1.611494000
C	-4.365312000	-3.816715000	-1.350302000
C	-4.629440000	-2.474460000	-1.081465000
C	-3.590346000	-1.545541000	-1.068387000
H	-0.994161000	-3.604951000	-1.811294000
H	-2.842471000	-5.271336000	-1.828629000
H	-5.178647000	-4.546676000	-1.358263000
H	-5.652294000	-2.147707000	-0.876687000
H	-3.788877000	-0.497051000	-0.833986000
H	-0.519535000	-1.049827000	-2.184770000
H	-1.587484000	0.075031000	-1.324243000
B	0.816833000	-1.947710000	-0.168367000
O	1.171396000	-2.631975000	-1.308251000

C	2.331043000	-3.389431000	-1.008214000
C	2.514075000	-3.248809000	0.514590000
O	1.635930000	-2.207606000	0.891575000
H	3.188599000	-2.978503000	-1.565306000
H	2.183373000	-4.433648000	-1.325091000
H	3.543858000	-2.982997000	0.792009000
H	2.232946000	-4.170378000	1.052165000
H	0.082050000	0.321390000	1.937578000
B	-0.939714000	-0.803951000	1.229343000
O	-2.169216000	-0.136581000	1.201129000
C	-2.929965000	-0.601499000	2.289677000
C	-1.936445000	-1.418626000	3.129285000
O	-0.920496000	-1.777550000	2.223802000
H	-2.384987000	-2.322702000	3.569882000
H	-1.515366000	-0.809076000	3.952034000
H	-3.760086000	-1.226631000	1.911677000
H	-3.363649000	0.246853000	2.844518000
C	-1.735602000	2.633380000	-0.005176000
C	-2.108384000	2.946343000	1.306211000
C	-2.734179000	2.419454000	-0.961461000
C	-3.452123000	3.047343000	1.652121000
H	-1.326008000	3.091021000	2.054306000
C	-4.078711000	2.516224000	-0.611373000
H	-2.449594000	2.156561000	-1.982983000
C	-4.444336000	2.828548000	0.696768000
H	-3.727461000	3.293141000	2.680851000
H	-4.846914000	2.337870000	-1.368021000
H	-5.499174000	2.899966000	0.971282000
C	3.461686000	0.429938000	0.110826000

C	3.831291000	0.270122000	1.452020000
C	4.140307000	-0.308604000	-0.868048000
C	4.891441000	-0.562046000	1.798596000
H	3.276314000	0.814132000	2.219447000
C	5.209058000	-1.131258000	-0.519417000
H	3.819184000	-0.231935000	-1.909090000
C	5.597231000	-1.254995000	0.814321000
H	5.171545000	-0.666686000	2.849436000
H	5.738644000	-1.686374000	-1.298119000
H	6.436194000	-1.898973000	1.087339000

H^{MPh}

C	-1.176837000	2.403782000	1.440248000
C	-2.860263000	0.502632000	1.691591000
N	-0.813219000	2.192184000	0.178234000
Zn	-1.802308000	0.911110000	-1.004644000
N	-2.846922000	0.054455000	0.451201000
C	-0.582901000	3.551855000	2.221932000
C	-3.710481000	-0.206822000	2.713896000
H	-3.708650000	0.321562000	3.674974000
H	-3.348752000	-1.234167000	2.872309000
H	-4.746673000	-0.299980000	2.355207000
H	-1.265266000	3.851678000	3.027358000
H	-0.389653000	4.416791000	1.572413000
H	0.378685000	3.277897000	2.681631000
C	-2.132006000	1.630863000	2.127650000
H	-2.333143000	1.945713000	3.151725000
N	1.673376000	-0.373835000	-0.189892000
C	3.081138000	-0.017800000	-0.401011000

C	4.016540000	-1.154117000	-0.069685000
C	4.673478000	-1.200166000	1.163827000
C	5.507802000	-2.270252000	1.485495000
C	5.694010000	-3.308981000	0.575407000
C	5.042108000	-3.272009000	-0.657650000
C	4.209543000	-2.202398000	-0.977554000
H	4.516654000	-0.389055000	1.879454000
H	6.016992000	-2.291764000	2.452377000
H	6.349240000	-4.147145000	0.825126000
H	5.187460000	-4.081996000	-1.376945000
H	3.692676000	-2.171138000	-1.940369000
H	3.325553000	0.854012000	0.219539000
H	3.208012000	0.281317000	-1.450061000
B	1.186727000	-0.385135000	1.152271000
O	1.852913000	0.261236000	2.168866000
C	1.084850000	0.104451000	3.348842000
C	0.052540000	-0.977808000	3.001382000
O	0.077397000	-1.060734000	1.588507000
H	0.596159000	1.061743000	3.594833000
H	1.738815000	-0.179275000	4.187367000
H	-0.957628000	-0.712012000	3.342912000
H	0.322339000	-1.960643000	3.424822000
H	-2.149587000	1.072965000	-2.537343000
B	0.911547000	-0.791350000	-1.305766000
O	1.448283000	-1.064807000	-2.536543000
C	0.366640000	-1.306517000	-3.426351000
C	-0.814537000	-1.612124000	-2.509114000
O	-0.468805000	-0.947294000	-1.292729000
H	-0.927192000	-2.688258000	-2.299503000

H	-1.767946000	-1.211234000	-2.880377000
H	0.615477000	-2.137871000	-4.101782000
H	0.183285000	-0.400832000	-4.028430000
C	-3.482967000	-1.153398000	0.097573000
C	-4.714792000	-1.141808000	-0.567062000
C	-2.840024000	-2.377831000	0.329531000
C	-5.305921000	-2.337214000	-0.971583000
H	-5.196835000	-0.182172000	-0.766811000
C	-3.438132000	-3.568503000	-0.075755000
H	-1.856089000	-2.362990000	0.803207000
C	-4.673269000	-3.554965000	-0.724946000
H	-6.269270000	-2.315155000	-1.487213000
H	-2.929202000	-4.517938000	0.110644000
H	-5.137959000	-4.490590000	-1.044529000
C	0.222414000	2.924381000	-0.434515000
C	1.515407000	2.993981000	0.105555000
C	-0.021837000	3.545632000	-1.668995000
C	2.523847000	3.687246000	-0.558736000
H	1.734504000	2.468819000	1.036445000
C	0.990888000	4.235017000	-2.329305000
H	-1.020030000	3.471421000	-2.105304000
C	2.269291000	4.314292000	-1.777515000
H	3.525376000	3.725080000	-0.122158000
H	0.776496000	4.715576000	-3.287126000
H	3.063900000	4.853425000	-2.298103000

F^{MDipp}

C	0.862102000	-2.759256000	0.874529000
C	-1.448469000	-2.003232000	1.538393000

N	1.248996000	-1.681545000	0.204164000
Zn	-0.093578000	-0.448401000	-0.479488000
N	-1.607612000	-1.003635000	0.698393000
C	1.850942000	-3.878448000	1.105269000
C	-2.364759000	-2.199155000	2.720706000
H	-2.910347000	-3.136833000	2.647276000
H	-3.065311000	-1.374588000	2.814149000
H	-1.756504000	-2.242738000	3.623665000
H	2.304760000	-3.801692000	2.092385000
H	2.647070000	-3.846459000	0.366077000
H	1.340376000	-4.836731000	1.043434000
C	-0.382457000	-2.907962000	1.468034000
H	-0.493597000	-3.789889000	2.084150000
N	0.364842000	1.197527000	-1.502698000
C	0.825319000	2.373203000	-1.527682000
C	1.794778000	2.949362000	-0.599818000
C	2.832752000	2.174133000	-0.089917000
C	3.804605000	2.750245000	0.707849000
C	3.735160000	4.095408000	1.029701000
C	2.701182000	4.873326000	0.530413000
C	1.749311000	4.311297000	-0.297711000
H	2.888128000	1.129822000	-0.353589000
H	4.620946000	2.144009000	1.070962000
H	4.490776000	4.542655000	1.658819000
H	2.649987000	5.924224000	0.774573000
H	0.955887000	4.919389000	-0.707015000
H	0.519257000	3.040719000	-2.339058000
H	-0.106921000	0.935613000	-3.719018000
B	-0.571418000	0.723196000	-2.633834000

O	-0.737897000	-0.762157000	-2.346695000
C	-2.037459000	-1.081401000	-2.769592000
C	-2.830603000	0.253373000	-2.753704000
O	-1.922825000	1.251435000	-2.403687000
H	-2.460675000	-1.836881000	-2.103768000
H	-1.995748000	-1.487323000	-3.790938000
H	-3.643770000	0.247874000	-2.019933000
H	-3.255432000	0.452313000	-3.750208000
C	2.635091000	-1.410827000	0.125592000
C	3.309815000	-1.003880000	1.292804000
C	3.310654000	-1.490293000	-1.097458000
C	4.654749000	-0.679530000	1.201436000
C	4.660919000	-1.166976000	-1.139380000
C	5.331245000	-0.764647000	-0.002072000
H	5.189348000	-0.359174000	2.081953000
H	5.196326000	-1.235070000	-2.073999000
H	6.381531000	-0.516573000	-0.050400000
C	-2.739747000	-0.165654000	0.708835000
C	-2.475733000	1.200458000	0.793042000
C	-4.067155000	-0.600862000	0.539422000
C	-3.470030000	2.157752000	0.721169000
H	-1.441220000	1.501677000	0.904931000
C	-5.059844000	0.372436000	0.500824000
C	-4.781200000	1.720708000	0.590111000
H	-6.087634000	0.064513000	0.378074000
H	-5.586944000	2.438907000	0.536496000
C	-3.152040000	3.629630000	0.780013000
H	-4.046632000	4.177962000	0.463729000
C	-2.006035000	3.984859000	-0.168513000

H	-1.052418000	3.672402000	0.250901000
H	-2.136719000	3.481050000	-1.123972000
H	-1.971638000	5.060672000	-0.331346000
C	-2.819030000	4.044144000	2.216066000
H	-2.607590000	5.110472000	2.262610000
H	-3.653716000	3.826876000	2.879668000
H	-1.945703000	3.502662000	2.573818000
C	-4.476802000	-2.046350000	0.344596000
H	-3.581362000	-2.673847000	0.370616000
C	-5.157959000	-2.257734000	-1.013430000
H	-5.386815000	-3.313471000	-1.147769000
H	-6.089148000	-1.699468000	-1.070746000
H	-4.523092000	-1.936374000	-1.833557000
C	-5.450468000	-2.507853000	1.437499000
H	-5.091601000	-2.267533000	2.433717000
H	-6.417299000	-2.027403000	1.308286000
H	-5.599278000	-3.583966000	1.373032000
C	2.574849000	-0.863344000	2.610525000
H	1.730778000	-1.556404000	2.617022000
C	1.994640000	0.548225000	2.740546000
H	1.425386000	0.633637000	3.664160000
H	2.786904000	1.292917000	2.748548000
H	1.330494000	0.761098000	1.902396000
C	3.448259000	-1.192287000	3.822431000
H	3.977543000	-2.131288000	3.674604000
H	4.177168000	-0.408149000	4.011013000
H	2.824359000	-1.284603000	4.709230000
C	2.602196000	-1.937040000	-2.353795000
H	1.535496000	-2.043021000	-2.133458000

C	3.134750000	-3.295834000	-2.816667000
H	2.990743000	-4.050504000	-2.046121000
H	2.607653000	-3.616691000	-3.712206000
H	4.197096000	-3.237785000	-3.044618000
C	2.743636000	-0.896022000	-3.464897000
H	2.376102000	0.068638000	-3.123353000
H	3.779066000	-0.788810000	-3.780234000
H	2.150998000	-1.194926000	-4.325892000

TS3^{MDipp}

C	0.964978000	-2.107379000	1.796585000
C	-1.535022000	-1.703510000	1.865861000
N	1.325354000	-1.155775000	0.936620000
Zn	-0.001054000	0.204241000	0.345876000
N	-1.610414000	-0.826096000	0.872933000
C	1.976886000	-3.144083000	2.212987000
C	-2.802655000	-2.174498000	2.529686000
H	-2.595514000	-2.943339000	3.283391000
H	-3.515596000	-2.572792000	1.793832000
H	-3.306988000	-1.324351000	3.014023000
H	1.625870000	-3.702818000	3.089000000
H	2.961140000	-2.707242000	2.425814000
H	2.123751000	-3.859839000	1.387874000
C	-0.327899000	-2.259581000	2.327195000
H	-0.426174000	-3.006653000	3.113406000
N	0.039623000	2.158079000	0.378985000
C	0.847026000	3.105954000	-0.068587000
C	2.247855000	2.969008000	-0.529156000
C	2.688469000	1.798853000	-1.153765000

C	3.987451000	1.711798000	-1.640364000
C	4.859686000	2.790615000	-1.491763000
C	4.426008000	3.965956000	-0.875858000
C	3.117302000	4.060137000	-0.412389000
H	1.991245000	0.972582000	-1.296430000
H	4.320155000	0.794521000	-2.129033000
H	5.883508000	2.718133000	-1.866783000
H	5.108295000	4.811960000	-0.766216000
H	2.760390000	4.983401000	0.052777000
H	0.573155000	4.166615000	0.103315000
H	-0.050816000	3.255954000	-1.595030000
B	-0.736581000	2.358686000	-0.919098000
O	-0.589777000	1.069415000	-1.649095000
C	-1.590766000	1.098001000	-2.640147000
C	-2.653219000	2.103507000	-2.120117000
O	-2.128033000	2.671796000	-0.950613000
H	-2.002985000	0.090092000	-2.782937000
H	-1.153831000	1.433587000	-3.598327000
H	-3.604329000	1.600590000	-1.883679000
H	-2.859107000	2.875813000	-2.885659000
C	2.661170000	-1.168611000	0.459568000
C	3.633236000	-0.376164000	1.106664000
C	2.988783000	-1.969080000	-0.654118000
C	4.958555000	-0.480296000	0.676393000
C	4.325039000	-2.023676000	-1.061778000
C	5.308085000	-1.307521000	-0.386208000
H	5.731439000	0.112706000	1.167966000
H	4.603580000	-2.646691000	-1.914597000
H	6.351089000	-1.376354000	-0.704658000

C	-2.878835000	-0.552567000	0.303651000
C	-3.651839000	0.489349000	0.815107000
C	-3.333490000	-1.318382000	-0.791525000
C	-4.918428000	0.780573000	0.307965000
H	-3.220076000	1.092740000	1.615255000
C	-4.615018000	-1.035975000	-1.275986000
C	-5.398277000	-0.020036000	-0.732667000
H	-5.009088000	-1.609536000	-2.117494000
H	-6.389051000	0.179106000	-1.151477000
C	-5.753036000	1.915543000	0.861706000
H	-6.438742000	2.220693000	0.051740000
C	-4.915731000	3.132608000	1.246673000
H	-4.330417000	2.942661000	2.160647000
H	-4.201514000	3.399972000	0.453839000
H	-5.565855000	3.996529000	1.454192000
C	-6.610499000	1.432697000	2.033192000
H	-7.255887000	2.241702000	2.411031000
H	-7.252979000	0.588205000	1.741087000
H	-5.971313000	1.093874000	2.864786000
C	-2.441379000	-2.369949000	-1.430773000
H	-1.415090000	-1.966472000	-1.374323000
C	-2.765597000	-2.622973000	-2.900440000
H	-1.990256000	-3.259306000	-3.354037000
H	-3.725139000	-3.150677000	-3.020246000
H	-2.821311000	-1.691913000	-3.483649000
C	-2.438921000	-3.701997000	-0.676071000
H	-2.033364000	-3.606159000	0.339001000
H	-3.458624000	-4.113804000	-0.605994000
H	-1.814637000	-4.438520000	-1.207081000

C	3.231373000	0.587572000	2.210246000
H	2.237528000	0.973997000	1.924478000
C	4.172598000	1.782482000	2.322117000
H	3.737204000	2.540994000	2.990140000
H	5.146369000	1.495948000	2.751217000
H	4.353585000	2.253842000	1.346454000
C	3.079971000	-0.086090000	3.576015000
H	2.270261000	-0.826968000	3.590587000
H	4.015312000	-0.588757000	3.871007000
H	2.846749000	0.667067000	4.344787000
C	1.904886000	-2.714118000	-1.411925000
H	1.067603000	-2.872669000	-0.714754000
C	2.345985000	-4.085163000	-1.913754000
H	2.766703000	-4.698066000	-1.101799000
H	1.488310000	-4.627740000	-2.340324000
H	3.106825000	-4.010793000	-2.706742000
C	1.373264000	-1.851923000	-2.560067000
H	0.956785000	-0.897085000	-2.204689000
H	2.176299000	-1.618796000	-3.277730000
H	0.572649000	-2.376202000	-3.105779000

References

1. Chamberlain, B. M.; Cheng, M.; Moore, D. R.; Ovitt, T. M.; Lobkovsky, E. B.; Coates, G. W. Polymerization of Lactide with Zinc and Magnesium β-Diiminate Complexes: Stereocontrol and Mechanism. J. Am. Chem. Soc. 2001, 123, 14, 3229–3238, DOI: 10.1021/ja003851f.
2. Dolomanov, O. V.; Bourhis, L. J.; Gildea, R. J.; Howard, J. A. K.; Puschmann, H. OLEX2 : A Complete Structure Solution, Refinement and Analysis Program. *J. Appl. Crystallogr.* **2009**, *42*, 339–341, DOI: 10.1107/S0021889808042726.
3. Sheldrick, G. M.; SHELXT – Integrated Space-Group and Crystal-Structure Determination. *Acta Crystallogr.* **2015**, *A71*, 3–8, DOI: 10.1107/S2053273314026370.
4. Sheldrick, G. M.; Crystal Structure Refinement with SHELXL. *Acta Crystallogr.* **2015**, *C71*, 3–8, DOI: 10.1107/S2053229614024218.
5. Frisch, M. J.; Trucks, G. W.; Schlegel, H. B.; Scuseria, G. E.; Robb, M. A.; Cheeseman, J. R.; Scalmani, G.; Barone, V.; Mennucci, B.; Petersson, G. A.; Nakatsuji, H.; Caricato, M.; Li, X.; Hratchian, H. P.; Izmaylov, A. F.; Bloino, J.; Zheng, G.; Sonnenberg, J. L.; Hada, M.; Ehara, M.; Toyota, K.; Fukuda, R.; Hasegawa, J.; Ishida, M.; Nakajima, T.; Honda, Y.; Kitao, O.; Nakai, H.; Vreven, T.; Montgomery Jr., J. A.; Peralta, J. E.; Ogliaro, F.; Bearpark, M.; Heyd, J. J.; Brothers, E.; Kudin, K. N.; Staroverov, V. N.; Kobayashi, R.; Normand, J.; Raghavachari, K.; Rendell, A.; Burant, J. C.; Iyengar, S. S.; Tomasi, J.; Cossi, M.; Rega, N.; Millam, J. M.; Klene, M.; Knox, J. E.; Cross, J. B.; Bakken, V.; Adamo, C.; Jaramillo, J.; Gomperts, R.; Stratmann, R. E.; Yazyev, O.; Austin, A. J.; Cammi, R.; Pomelli, C.; Ochterski, J. W.; Martin, R. L.; Morokuma, K.; Zakrzewski, V. G.; Voth, G. A.; Salvador, P.; Dannenberg, J. J.; Dapprich, S.; Daniels, A. D.; Farkas, Ö.; Foresman, J. B.; Ortiz, J. V; Cioslowski, J.; Fox, D. J. *Gaussian 16, Revision C.01*; Gaussian, Inc.: Wallingford CT, 2016.
6. Adamo, C.; Barone, V. Toward Reliable Density Functional Methods without Adjustable Parameters: The PBE0 Model. *J. Chem. Phys.* **1999**, *110*, 6158–6170, DOI: 10.1063/1.478522.
7. Ernzerhof, M.; Scuseria, G. E. Assessment of the Perdew–Burke–Ernzerhof Exchange-Correlation Functional. *J. Chem. Phys.* **1999**, *110*, 5029–5036, DOI: 10.1063/1.478401.
8. Grimme, S.; Antony, J.; Ehrlich, S.; Krieg, H. A Consistent and Accurate Ab Initio Parametrization of Density Functional Dispersion Correction (DFT-D) for the 94 Elements H-Pu. *J. Chem. Phys.* **2010**, *132*, 154104, DOI: 10.1063/1.3382344.
9. Grimme, S.; Ehrlich, S.; Goerigk, L. Effect of the Damping Function in Dispersion Corrected Density Functional Theory. *J. Comput. Chem.* **2011**, *32*, 1456–1465, DOI: 10.1002/jcc.21759.

10. Weigend, F.; Ahlrichs, R. Balanced Basis Sets of Split Valence, Triple Zeta Valence and Quadruple Zeta Valence Quality for H to Rn: Design and Assessment of Accuracy. *Phys. Chem. Chem. Phys.* **2005**, *7*, 3297–3305, DOI: 10.1039/b508541a.

11. Marenich, A. V.; Cramer, C. J.; Truhlar, D. G. Universal Solvation Model Based on Solute Electron Density and on a Continuum Model of the Solvent Defined by the Bulk Dielectric Constant and Atomic Surface Tensions. *J. Phys. Chem. B* **2009**, *113*, 6378–6396, DOI: 10.1021/jp810292n.

12. Cancès, E.; Mennucci, B.; Tomasi, J. A New Integral Equation Formalism for the Polarizable Continuum Model: Theoretical Background and Applications to Isotropic and Anisotropic Dielectrics. *J. Chem. Phys.* **1997**, *107*, 3032–3041, DOI: 10.1063/1.474659.

13. Kelly, C. P.; Cramer, C. J.; Truhlar, D. G. SM6: A Density Functional Theory Continuum Solvation Model for Calculating Aqueous Solvation Free Energies of Neutrals, Ions, and Solute−Water Clusters. *J. Chem. Theory Comput.* **2005**, *1*, 1133–1152, DOI: 10.1021/ct050164b.

14. Sparta, M.; Riplinger, C.; Neese, F. Mechanism of Olefin Asymmetric Hydrogenation Catalyzed by Iridium Phosphino-Oxazoline: A Pair Natural Orbital Coupled Cluster Study. *J. Chem. Theory Comput.* **2014**, *10*, 1099–1108, DOI: 10.1021/ct400917j.

15. Fantuzzi, F.; Nascimento, M. A. C.; Ginovska, B.; Bullock, R. M.; Raugei, S. Splitting of Multiple Hydrogen Molecules by Bioinspired Diniobium Metal Complexes: A DFT Study. *Dalton Trans.* **2021**, *50*, 840–849, DOI: 10.1039/D0DT03411H.

16. Ishida, K.; Morokuma, K.; Komornicki, A. The Intrinsic Reaction Coordinate. An ab initio Calculation for HNC→HCN and $H^- + CH_4 \rightarrow CH_4 + H^-$. *J. Chem. Phys.* **1977**, *66*, 2153–2156, DOI: 10.1063/1.434152.

17. Hirshfeld, F. L. Bonded-Atom Fragments for Describing Molecular Charge Densities. *Theor. Chim. Acta* **1977**, *44*, 129–138, DOI: 10.1007/BF00549096.

18. Mulliken, R. S. Electronic Population Analysis on LCAO–MO Molecular Wave Functions. I. *J. Chem. Phys.* **1955**, *23*, 1833–1840, DOI: 10.1063/1.1740588.

19. Bickelhaupt, F. M.; van Eikema Hommes, N. J. R.; Fonseca Guerra, C.; Baerends, E. J. The Carbon−Lithium Electron Pair Bond in $(CH_3Li)_n$ (n = 1, 2, 4). *Organometallics* **1996**, *15*, 2923–2931, DOI: 10.1021/om950966x.

20. Löwdin, P. On the Non-Orthogonality Problem Connected with the Use of Atomic Wave Functions in the Theory of Molecules and Crystals. *J. Chem. Phys.* **1950**, *18*, 365–375, DOI: 10.1063/1.1747632.

21. Lu, T.; Chen, F. Multiwfn: A Multifunctional Wavefunction Analyzer. *J. Comput. Chem.* **2012**, *33*, 580–592, DOI: 10.1002/jcc.22885.

22. CYLview, 1.0b; Legault, C. Y., Université de Sherbrooke, 2009 (http://www.cylview.org).

23. Chai, J.-D.; Head-Gordon, M. Long-Range Corrected Hybrid Density Functionals with Damped Atom–Atom Dispersion Corrections. *Phys. Chem. Chem. Phys.* **2008**, *10*, 6615. DOI: 10.1039/b810189b.

24. Guo, Y.; Riplinger, C.; Becker, U.; Liakos, D. G.; Minenkov, Y.; Cavallo, L.; Neese, F. Communication: An Improved Linear Scaling Perturbative Triples Correction for the Domain Based Local Pair-Natural Orbital Based Singles and Doubles Coupled Cluster Method [DLPNO-CCSD(T)]. *J. Chem. Phys.* **2018**, *148*, 011101. DOI: 10.1063/1.5011798.
